# Synthesis of Enantiostructured Triacylglycerols Possessing a Saturated Fatty Acid, a Polyunsaturated Fatty Acid and an Active Drug Intended as Novel Prodrugs

**DOI:** 10.3390/molecules29235745

**Published:** 2024-12-05

**Authors:** Lena Rós Jónsdóttir, Gudmundur G. Haraldsson

**Affiliations:** Science Institute, Chemistry Department, University of Iceland, Dunhaga 3, 107 Reykjavik, Iceland; lrj@hi.is

**Keywords:** asymmetric synthesis, enantiostructured triacylglycerols, lipase, n-3 PUFAs, acylglycerol prodrugs

## Abstract

This report describes the asymmetric synthesis of a focused library of enantiopure structured triacylglycerols (TAGs) comprised of a single saturated fatty acid (C6, C8, C10, C12, C14 or C16), a pure bioactive n-3 polyunsaturated fatty acid (EPA or DHA) and a potent drug (ibuprofen or naproxen) intended as a novel type of prodrug. One of the terminal *sn*-1 or *sn*-3 positions of the glycerol backbone is occupied with a saturated fatty, the remaining one with a PUFA, and the drug entity is present in the *sn*-2 position. This was accomplished by a six-step chemoenzymatic approach starting from enantiopure (*R*)- and (*S*)-solketals. The highly regioselective immobilized *Candida antarctica* lipase (CAL-B) played a crucial role in the regiocontrol of the synthesis. All combinations, a total of 48 such prodrug TAGs, were prepared, isolated and fully characterized, along with 60 acylglycerol intermediates, obtained in very high to excellent yields.

## 1. Introduction

Fish oil and marine fat are the main sources of the long-chain omega-3 polyunsaturated fatty acids (n-3 PUFAs), of which eicosapentaenoic acid (EPA) and docosahexaenoic acid (DHA) are largely predominant [1]. Algal oil extracted from marine microalgae biomass grown in contained fermentation vessels has also become a major sustainable vegetarian source of EPA and DHA for human consumption [2,3]. EPA and DHA are attributed to numerous beneficial effects on human health and prevention of various health disorders, including inflammation, cardiovascular and brain diseases, and many more [1,4,5,6]. They act in membranes, cell signaling, as precursors to potent lipid mediators and regulate gene expression via receptors. EPA and DHA are indeed precursors to various prostanoids and leukotrienes [7] as well as the more recently established highly potent resolvins, protectins and maresins that exhibit potent anti-inflammatory and pro-resolving actions, currently known as the specialized pro-resolving mediators (SPMs) [8,9,10].

The term structured lipids refers to acylglycerol lipids constituting certain types of fatty acids placed at pre-determined positions of the glycerol backbone [11,12,13]. Structured triacylglycerols (TAGs) carrying long-chain bioactive PUFAs such as EPA and DHA at the 2-position and saturated medium chain (C6:0, C8:0 and C10:0) fatty acids (MCFAs) at the terminal 1,3-positions have acquired a growing interest of scientists because of their properties and nutritional value [14]. In such MLM (medium-long-medium)-type TAGs, the MCFAs located at the end-positions undergo rapid hydrolysis by pancreatic lipase in the digestive tract. They are rapidly absorbed into the intestines and rapidly carried to the liver, where they act as a quick but only moderate source of energy. The remaining 2-monoacylglycerols constituting the n-3 PUFAs are absorbed through the intestinal wall, accumulate as TAGs in the adipose tissue and as phospholipids in cell membranes as a source of bioactive PUFAs and essential fatty acids [15,16].

Natural TAGs differ significantly in animals and plants from species to species, where the fatty acids by no means happen to be randomly distributed. Classical examples of naturally structured TAGs include cocoa butter [13,17], used in chocolate manufacturing, and TAGs in human milk [12,18]. Generally, in fish oil TAGs, the mid position of the glycerol backbone is more enriched with the n-3 PUFAs, especially DHA, compared to the terminal positions. Notably, in the TAG oil of marine mammals, including whales and seals, this is the other way around, with the mid position less enriched with these PUFAs than the outer positions [19].

We have reported an efficient two-step chemoenzymatic synthesis of regiopure MLM-type structured TAGs constituting pure EPA or DHA in the 2-position and pure short- and medium-chain saturated fatty acids in the 1,3-positions. This was accomplished with the aid of a highly regioselective immobilized *Candida antarctica* lipase (CAL-B) that was observed to act exclusively at the end-positions of glycerol [20,21]. We have also reported on a multi-step asymmetric synthesis of what we name enantiostructured TAGs, also involving the highly regioselective lipase. Glycerol is prochiral, causing a TAG molecule to become chiral when the two fatty acyl groups occupying the terminal 1,3-positions are different, regardless of the acyl group accommodating the 2-position. Accordingly, the two enantiotopic terminal carbons of the glycerol backbone in TAGs are distinguishable by a stereospecific numbering indicated by a prefix, *sn*-, with the *pro-S* hydroxycarbon group of glycerol referring to the *sn*-1 position, the *pro-R* group to the *sn*-3 position, and the central carbon to the *sn*-2 position [22,23]. Numerous tailor-made enantiostructured TAGs have been synthesized with a variety of pre-determined fatty acyl groups occupying the individual stereospecific positions of the glycerol backbone [24,25,26,27]. There are multiple reports on the stereospecific positioning of fatty acids in animal and plant TAGs [28,29].

**Figure 1:**
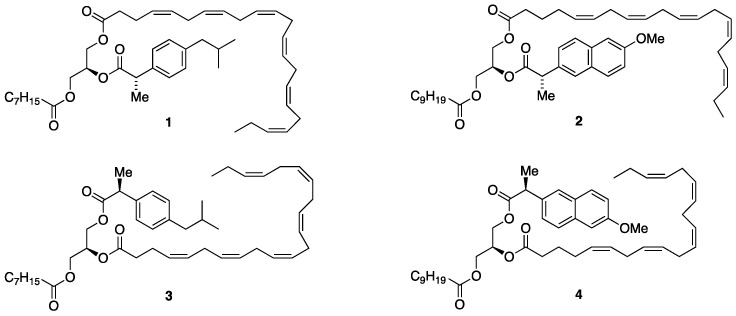
The structure of TAG prodrugs **1** and **2** belong to the first category prodrugs, and TAG products **3** and **4** belong to the second category prodrugs.

The current report describes a further extension of the concept of structured and enantiostructured TAGs, namely our synthesis of enantiostructured TAGs intended as prodrugs possessing a pure saturated fatty acid (SFA), EPA or DHA and a potent drug all attached as carboxylic esters to pre-determined stereospecific positions of the glycerol framework of the TAG molecule. To demonstrate this novel concept, we have selected the well-known non-steroidal anti-inflammatory drugs (NSAIDs) (*S*)-ibuprofen and (*S*)-naproxen and chosen to follow procedures already established for the synthesis of the enantiostructured TAGs previously described. This is illustrated in Figure 1.

Two categories of such prodrug designs are shown in Figure 1. Structures **1** and **2** belong to the first category, where the active drugs are located in the *sn*-2 position along with EPA or DHA in one of the terminal positions and an SFA in the other. Structure **1** contains (*S*)-ibuprofen esterified in the *sn*-2 position, DHA in the *sn*-1 position and caprylic acid (C8:0) in the remaining *sn*-3 position. Likewise, structure **2** contains (*S*)-naproxen in the *sn*-2 position, EPA in the *sn*-1 position and capric acid (C10:0) in the *sn*-3 position. In the second prodrug category, the location of the n-3 PUFAs and the drug has been interchanged with the drugs located in the *sn*-1 position and the PUFAs in the *sn*-2 position, as shown in structures **3** and **4**. The current paper reports the synthesis of the first category prodrugs with all combinations of medium- and longer-chain SFAs ranging from C6:0 to C16:0, EPA and DHA, (*S*)-ibuprofen and (*S*)-naproxen for both (*R*)- and (*S*)-TAG diastereomers. The synthesis of all the corresponding diasteromers of the second category of prodrugs is also underway to be reported.

## 2. Results and Discussion

A prodrug is a compound that undergoes an intra- or extra-cellular bioconversion within the human body to liberate an active drug. Prodrugs are designed to improve the bioavailability of a drug and how it is absorbed, distributed, metabolized and excreted (ADME) [30,31,32]. Lipid-based drug carriers and prodrugs offer advantages such as increased absorption through the intestines and they enhance drug availability and targeting [33,34]. The current report describes the preparation of enantiostructured TAGs constituting even carbon number SFAs ranging from C6:0 to C16:0, EPA and DHA, and an active drug attached to the glycerol backbone, where the benign effects of the n-3 PUFAs, assumed benefits of structured and enantiostructured TAGs, and the pharmaceutical properties of the drug are combined in a single molecule. It is our belief that this approach may offer an interesting and novel form of a prodrug.

Two regioisomeric prodrug forms are proposed, and their general structures are displayed in Figure 1. In the first form (represented by structures **1** and **2**), the active drug is attached as an ester to the *sn*-2 position of the TAG with the PUFA at the *sn*-3 and the SFA at the *sn*-1 positions (first category TAG prodrugs). The current report describes the synthesis of TAG prodrugs that belong to this category. All corresponding diastereomers where the acyl groups at the end-positions have been interchanged (the n-3 PUFAs at the *sn*-1 and the SFAs at the *sn*-3 positions) were also synthesized. In the second form (represented by **3** and **4**), the location of the drug and the PUFA has been reversed such that the drug is at the *sn*-3 position with the PUFA at the *sn*-2 position (second category TAG prodrugs). Their synthesis will be addressed in a separate report that is underway to be completed.

A prodrug design of the type described may offer good opportunities for controlling the site-specific release of not only the drug but also the bioactive n-3 PUFA as a combination of the regio- and enantio-specific location of the SFA and its length, the n-3 PUFA and the drug within the TAG backbone and their consequent release. This may result in higher bioavailability of the drug with less drug needed and, therefore, fewer harmful side effects. As far as we know, there are currently no reports on acylglycerol-based prodrugs possessing n-3 PUFAs and active drugs attached to the glycerol backbone.

Serving as precursors to the specialized pro-resolving mediators, EPA and DHA may be regarded as anti-inflammatory prodrugs [35]. It will be of interest to find out how EPA and DHA may act on their own or perhaps through some interactive or synergistic effects with the drugs. The non-steroidal anti-inflammatory drugs were an obvious choice to demonstrate this concept, and they offer the prerequisite of a carboxyl group to allow an ester bond formation to the glycerol backbone.

By varying the location of the three counterparts present in the designed prodrugs within the glycerol skeleton, better control may be gained on when (timing) and where (site) these counterparts will be released from the prodrug molecule. In the current prodrug design, the saturated fatty acids, located at the terminal positions only, will be most accessible for cleavage at an early stage in the digestive tract by pancreatic lipase. The SFAs are generally significantly better substrates to lipase than the n-3 PUFAs [36] and will undergo a cleavage ahead of the other counterparts present to deliver a diacylglycerol (DAG) derivative. The DAG delivery rate may depend both on the length of the SFAs ranging from C6:0 to C16:0 as well as their location at the *sn*-1 or the *sn*-3 positions by the pancreatic lipase fatty acid selectivity and its enantioselectivity.

Varying the location of the drug and the bioactive n-3 PUFAs between the terminal and mid positions within the glycerol framework will presumably influence their timing and site of release. In the case of the first category prodrugs with the drug located at the *sn*-2 position and the n-3 PUFAs at the terminal positions, it is anticipated that the release of the PUFA from the DAG will take place prior to the drug to form a 2-monoacylglycerol (2-MAG) with the drug still attached. The rate of release of the n-3 PUFA will assumingly depend on its stereospecific location between the *sn*-1 and *sn*-3 positions within the DAGs, and it is anticipated that the release of EPA will occur faster than DHA by the fact that lipase prefers EPA over DHA [36]. In the case of the second category of prodrugs, the situation is different and perhaps more complicated when the active drug is located at a terminal position. However, it is anticipated that all parameters discussed above may enable some fine-tuning of the prodrug in terms of the release of both the PUFA and the drug.

EPA and DHA are not only regarded as prodrugs, but they are also available in their ethyl ester form as prescription drugs registered as an adjuvant therapy to treat hypertriglyceridemia [37] both as a mixture of EPA and DHA [38,39] as well as virtually pure EPA [40,41,42]. This may enable further development of our prodrug concept based on the enantiostructured TAGs into a codrug formula. A codrug constitutes two drug components that display activity against the same disease. When released, they may offer various beneficial effects, including therapeutic synergy [43,44,45,46]. One embodiment of such a codrug might involve a potent statin drug in combination with EPA and DHA in such a molecule without or, if needed, with a suitable linker to the glycerol moiety.

### 2.1. Synthetic Strategies

**Figure 2:**
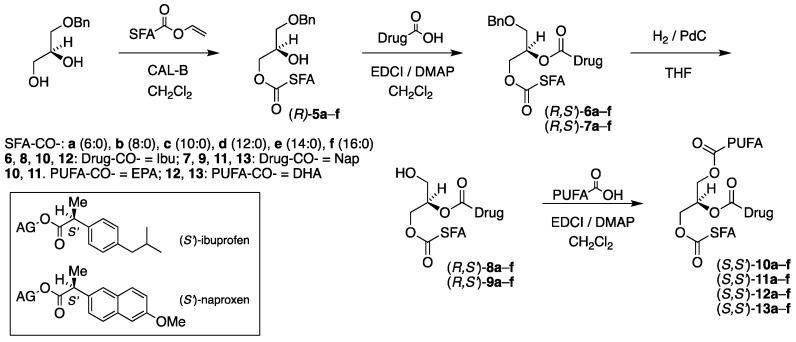
Chemoenzymatic synthesis of the first category TAG prodrug diastereomer series (*S*,*S′*)-**10a**–**f**–**13a**–**f**, starting from 1-*O*-benzyl-*sn*-glycerol. In the scheme, SFA-CO-, PUFA-CO- and Drug-CO- refer to the corresponding saturated fatty acyl, polyunsaturated fatty acyl and drug acyl group substituents, respectively. In box: (*S′*)-ibuprofen and (*S′*)-naproxen attached as esters to acylglycerols (AG).

A four-step chemoenzymatic approach was designed for the synthesis of the first category of TAG prodrugs, which is depicted in Figure 2. It is based on the use of 1-*O*-benzyl-*sn*-glycerol (prepared in two steps from (*R*)-solketal [24]) as a chiral precursor, of which the *sn*-1 position is protected as a benzyl ether. The first step involves a lipase-promoted regioselective acylation of the *sn*-3 hydroxyl group of the diol with a saturated fatty acid. The second step involves an incorporation of the drug into the remaining *sn*-2 position by use of a chemical coupling agent. This is followed by the removal of the benzyl protective group, and in the final step, the second unsaturated fatty acid is introduced to the *sn*-1 position of the glycerol backbone brought about by the same chemical coupling agent to complete the synthesis.

As can be noticed in Figure 2, all combinations of the six saturated fatty acids caproic, caprylic, capric, lauric, myristic and palmitic acids (C6:0, C8:0, C10:0, C12:0, C14:0 and C16:0, respectively) are located at the *sn*-3 position, EPA and DHA at the remaining *sn*-1 terminal position and (*S*)-ibuprofen (Ibu) and (*S*)-naproxen (Nap) at the *sn*-2 position of the glycerol moiety were under the scope to be synthesized. This results in a focused library of a total of 24 targeted enantiostructured TAG prodrugs (*S*,*S′*)-**10a**–**f**–**13a**–**f**. This synthetic task also involves a total of 30 enantiopure acylglycerol intermediates.

**Figure 3:**
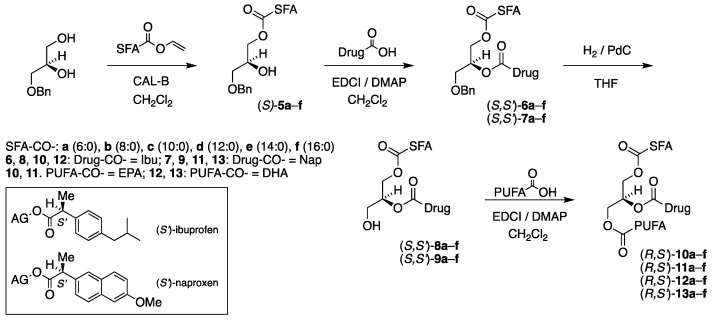
Chemoenzymatic synthesis of the first category TAG prodrug diastereomer series (*R*,*S′*)-**10a**–**f**–**13a**–**f**, starting from 3-*O*-benzyl-*sn*-glycerol. In the scheme, SFA-CO-, PUFA-CO- and Drug-CO- refer to the corresponding saturated fatty acyl, polyunsaturated fatty acyl and drug acyl group substituents, respectively. In box: (*S′*)-ibuprofen and (*S′*)-naproxen attached as esters to acylglycerols (AG).

We were equally interested in the corresponding TAG prodrug products (*R*,*S′*)-**10a**–**f**–**13a**–**f**, where the enantiospecific location of the SFAs and the PUFAs has been interchanged with the drugs still located at the *sn*-2 position. This results in an identical number of target products and intermediates. It should be noted that the resulting TAG prodrug products are diastereomeric to those shown in Figure 2, and the synthetic route is identical, this time starting from 3-*O*-benzyl-*sn*-gycerol (prepared in two steps from (*S*)-solketal [26]) as a chiral precursor. This is illustrated in Figure 3.

### 2.2. The Enzymatic Coupling of the SFAs

The first step involved an enzymatic coupling of the saturated fatty acids activated as vinyl esters to the terminal position of the benzyl-protected glycerols. As anticipated and previously described [20,21], the immobilized *Candida antarctica* lipase B (CAL-B) acylated the protected glycerol exclusively at the primary alcohol position. The reaction was performed in dry dichloromethane at r.t., and it took the lipase only 90 min to complete the reaction, as was confirmed by TLC monitoring and ^1^H NMR spectroscopy. There were no indications of any acylation taking place at the 2-position of the glycerol backbone.

All the products were obtained as colorless oils in excellent yields (>94%) in all cases except one (87%) after purification by silica gel chromatography. Table 1a,b show the identity, yields and specific optical activity of the resulting benzyl-protected *sn*-3-MAG intermediates (*R*)-**5a**–**f** derived from 1-*O*-benzyl-*sn*-glycerol (Table 1a) and the corresponding *sn*-1-MAGs (*S*)-**5a**–**f** derived from 3-*O*-benzyl-*sn*-glycerol, in accordance with the reaction schemes in Figure 2 and Figure 3.

The use of the saturated fatty acids activated as vinyl esters secures a fast irreversible reaction that is crucial for maintaining the excellent regioselectivity of the lipase [20,21]. Another important parameter offered by the lipase is the mildness under which the lipase acts, especially the low temperatures, to prevent acyl migration [27,47], which is detrimental to the regiocontrol provided by the lipase.

All partially acylated glycerol intermediates possessing an acyl group adjacent to a free hydroxyl group are prone to undergo such spontaneous thermodynamically controlled acyl migration. Because silica gel is known to promote acyl migration, special care had to be taken when it came to purification by means of chromatography, which required the use of silica gel impregnated with 4% boric acid, which is known to suppress acyl migration [27,48].

The glyceryl proton region of the ^1^H NMR spectra (δ 5.40–3.45 ppm) is ideally suited to confirm the structure and evaluate the purity of all partial and intermediate acylglycerol derivatives involved in the synthesis as well as the final triacylglycerol products. Quite characteristic patterns of peaks are provided for each of the individual acylglycerols. This is also of great use to accurately detect the level of undesired acyl migration-related byproducts, as we have demonstrated in detail in numerous previous reports [20,21,24,25,26,27]. In the current work, we benefited from the spectral details obtained from the ^1^H NMR and the 2D-NMR ^1^H-^1^H-COSY spectroscopy, enabling a full assignment of the ^1^H NMR data to establish the regiopurity of all intermediates and products involved. Making sure that no acyl migration has taken place enables us to conclude about full control of the stereochemistry of all the products.

The structures of the benzyl-protected MAGs were confirmed by the characteristic pattern for the glyceryl proton region of their ^1^H-NMR spectra. Figure S1 in the Supplementary Materials presents a comparison of the glyceryl region of the benzyl-protected glycerol starting material and the benzyl-protected *sn*-3-MAG (*R*)-**5a**. The characteristic pattern of peaks for the two types of glycerols is clearly evident. Upon acylation the two protons belonging to the *sn*-3 position have undergone a dramatic down-field shift that is also affecting the proton belonging to the *sn*-2 position of the glycerol backbone. No sign of acyl migration was observed in the case of the benzyl-protected *sn*-3-MAG derivatives. Acyl migration side reactions would distort the peak pattern and give additional peaks into their glyceryl proton region.

### 2.3. The Coupling of the Active Drugs

The second step involved a chemical coupling of the drugs into the *sn*-2 position of the benzyl-protected *sn*-3-MAGs (*R*)-**5a**–**f** and (*S*)-**5a**–**f** by use of 1-ethyl-3-(3-dimethylaminopropyl)carbodiimide (EDCI) as a coupling agent in the presence of 4-dimethylaminopyridine (DMAP) serving both as a catalyst and a base. The reactions were performed under conditions identical to those previously described in our syntheses of structured and enantiostructured TAGs using 10% excess of the drugs in dichloromethane at room temperature under which no acyl migration took place [20,21,24,25,26,27].

Table 2a,b outline the yields and optical activity of all intermediate products (*R*,*S′*)-**6a**–**f** and (*S*,*S′*)-**6a**–**f** obtained from (*R*)-**5a**–**f** and (*S*)-**5a**–**f** acylated with (*S*)-ibuprofen, respectively. This is in full accordance with the schemes shown in Figure 2 and Figure 3 with *R* and *S* referring to the absolute configuration of the glycerol moiety and *S′* to that of the drugs.

As can be noticed from Table 2a, the yields obtained for the (*R*,*S′*)-**6a**–**f** series were excellent (91–98%) in all cases except one (80%). The diastereomeric (*S*,*S′*)-**6a**–**f** series was accomplished in 81–95% yields, as can be noticed from Table 2b.

Similarly, the yields and optical activity of all the intermediate products (*R*,*S′*)-**7a**–**f** and (*S*,*S′*)-**7a**–**f** obtained from the corresponding acylation of the (*R*)-**5a**–**f** and (*S*)-**5a**–**f** with (*S*)-naproxen are outlined in Table 3a,b, respectively. As may be noticed from Table 3a, excellent yields were accomplished for the (*R*,*S′*)-**7a**–**f** series (90–98%).

Excellent yields were also obtained in most cases for the corresponding diastereomeric (*S*,*S′*)-**7a**–**f** series, but (*S*,*S′*)-**7e** and **7f** were afforded in lower 75 and 77% yields, respectively. Because these lower yields have not been optimized, we believe they may be significantly improved rather than being associated with diastereomeric issues. Solid products were obtained for all naproxen products constituting the longer-chain SFAs C10:0 to C16:0. They all afforded white, thin, needle-like crystals from hexane.

Figure S2 in the Supplementary Materials provides a comparison of the glyceryl proton region of the ^1^H NMR spectrum of (*R*,*S′*)-**6a**, which is typical for a benzyl-protected glycerol possessing a drug in the 2-position and an SFA in a terminal position, and the precursor (*R*)-**5a**. As can be noticed, the proton belonging to the *sn*-2 position has undergone a dramatic down-field shift upon acylation into that position by the drug. This is also significantly affecting the two protons belonging to the *sn*-1 position. Evidently, there are no signs of an acyl migration taking place. The dramatic change in the peak pattern for the benzyl protons is also noteworthy from the figure from a singlet in the precursor to a typical AB-quartet that is interfering with one of the protons belonging to the *sn*-3 position.

### 2.4. The Removal of the Benzyl Protective Group

The third step of the first category of prodrug synthesis involved the removal of the benzyl protective group. All compounds of the ibuprofen and naproxen series (*R*,*S′*)-**6a**–**f**, (*S*,*S′*)-**7a**–**f**, (*R*,*S′*)-**7a**–**f** and (*R*,*S′*)-**7a**–**f** were subjected to catalytic hydrogenolysis by use of a Pd/C catalyst in a mixture of THF and n-hexane under atmospheric pressure at r.t. Catalytic amount of perchloric acid was used to initiate the reaction by following a procedure previously described [26]. It was noted that the reaction involving the starting material as liquids proceeded significantly faster than those in the solid form and required 15 min as compared with 35 min.

The yields and specific optical rotation values are revealed in Table 4a,b for the ibuprofen adducts (*R*,*S′*)-**8a**–**f** and (*S*,*S′*)-**8a**–**f** obtained from the deprotection reaction, respectively. All products were obtained as liquids, and as can be noticed, in very high to excellent yields (84–98%).

Table 5a,b similarly display the corresponding results for the naproxen adducts (*R*,*S′*)-**9a**–**f** and (*S*,*S′*)-**9a**–**f**, respectively. Again, very high to excellent yields were obtained for all products (84–98%). As before, the products having SFAs of a chain length from C10:0 to C16:0 were all obtained as crystalline material after crystallization from n-hexane.

No acyl migration was observed to take place despite the use of the perchloric acid, but special care had to be taken to neutralize the reaction immediately after the reaction had proceeded to completion by the use of sodium bicarbonate. This was evident from the glyceryl proton region of the ^1^H NMR spectra of the products showing no signs of acyl migration that are easily detected in the glyceryl proton region. Shortly after the reactions described herein were performed, we discovered that the use of the perchloric acid to initiate the reaction was not necessary when running the reaction in a pure THF as a solvent instead of mixing it with n-hexane [25].

As can be noticed in Figure S3 of the Supplementary Materials providing a comparison between the glyceryl proton region of the product (*R*,*S′*)-**9d** and its precursor (*R*,*S′*)-**7d**, it is evident that the removal of the benzyl protective moiety has resulted in a slight down-field shift of the protons belonging to the *sn*-1 carbon with the two doublets of doublets merging closer together to give a multiplet. Only minor changes occurred to the protons belonging to the *sn*-3 carbon, whereas the protons belonging to the *sn*-2 carbon underwent a slight up-field shift.

### 2.5. The Coupling of the PUFA

The fourth and last step of the prodrug synthesis involved a chemical coupling of EPA and DHA into the open end-position of the diacylglycerols possessing the drug and the SFA obtained from the previous step. Previously described procedures involving approximately 5–10% excess of EPA and DHA using EDCI as a coupling agent in the presence of DMAP in dichloromethane at r.t. were followed under which conditions no acyl migration took place [20,21,26].

All products were obtained as yellowish to yellow oils in very high to excellent yields, with a few exceptions. The reactions involving DHA were observed to require longer reaction time than those of EPA and afforded somewhat lower yields. Table 6, Table 7, Table 8 and Table 9 outline the yields and the specific optical activity of the products in accordance with the reaction schemes in Figure 2 and Figure 3. The TAG prodrug products (*S*,*S′*)-**10a**–**f** and (*R*,*S′*)-**10a**–**f** possessing an SFA, EPA and ibuprofen are shown in Table 6a and Table 6b, respectively.

Similarly, the corresponding TAG prodrug products (*S*,*S′*)-**11a**–**f** and (*R*,*S′*)-**10a**–**f** possessing an SFA, EPA and naproxen are shown in Table 7a and Table 7b, respectively.

Table 8a and Table 8b outline the TAG prodrug products (*S*,*S′*)-**12a**–**f** and (*R*,*S′*)-**12a**–**f** possessing an SFA, DHA and ibuprofen, respectively.

Finally, the TAG prodrug products (*S*,*S′*)-**13a**–**f** and (*R*,*S′*)-**13a**–**f** possessing an SFA, DHA and naproxen are outlined in Table 9a and Table 9b, respectively.

As may be noticed from Figure S4 of the Supplementary Materials providing a comparison of the glyceryl proton region of the product (*R*,*S′*)-**11c** and the precursor (*R*,*S′*)-**9c**, changes anticipated for TAGs have taken place with a significant down-field shift of the protons belonging to the *sn*-1 position upon acylation into that position. They now resonate as two well-dispersed doublets of doublets, with one of them merging with one of the peaks from the protons belonging to the *sn*-3 position.

## 3. Materials and Methods

### 3.1. General Information

The ^1^H- and ^13^C-NMR spectra were recorded on a 400 MHz Bruker Avance NEO 400 spectrometer (Bruker Switzerland AG, Faellanden, Switzerland). Chemical shifts (δ) are reported in parts per million (ppm) from tetramethylsilane with the solvent resonance used as an internal standard. In all cases, the solvent was deuterochloroform, which had been filtered through the aluminum oxide to rid of acid contamination. The coupling constants (*J*) are given in Hertz (Hz). The following abbreviations are used to describe the multiplicity: s, singlet; d, doublet; t, triplet; q, quartet; dd, doublet of doublets; dt, doublet of triplets; AB q, AB-quartet; and m, multiplet. For ^13^C-NMR, the number of carbon nuclei contributing to each signal is indicated in parentheses after the chemical shift value. Infrared spectra were recorded on a Nicolet Avatar FT-IR (E.S.P.) spectrometer (Thermo Scientific, Madison, WI, USA) using sodium chloride windows (NaCl) for liquid compounds or potassium bromide pellets (KBr) for solids. The following abbreviations are used to describe the peaks: s, strong; vs, very strong; m, medium; w, weak; and br, broad. The high-resolution mass spectra (HMRS) were recorded on a Bruker OTOF-Q Compact ESI mass spectrometer (Bruker Daltonic, Bremen, Germany). The optical activity was measured on an Autopol V automatic Polarimeter from Rudolph Research Analytical (Hacketstown, NJ, USA) using a 40T-2.5-100-0.7 TempTrol polarimetric cell with 2.5 mm inside diameter, 100 mm optical length and 0.7 mL volume with c (concentration) referring to g sample/100mL. Melting points were determined using a Büchi m-560 melting point apparatus (Uster, Switzerland). TLC monitoring was done on silica plates from SiliCycle (Québec, QC, Canada) and the plates were developed in 4% PMA solution in methanol. Boric acid-impregnated silica gel was prepared by dissolving 4 g of boric acid in 100 mL methanol and then adding 55 g of silica and swirling the resulting slurry for a few minutes. The methanol was then evaporated off, and the silica was dried in vacuo for 6 h at 40 °C.

All chemicals and solvents were used without further purification unless otherwise stated. All solvents used, deuterated chloroform (99.8% D), diethyl ether (≥99.8%), ethyl acetate (≥99.7%), dichloromethane (99.8%), ethanol (≥99.8%), hexane (>99%), methanol (99.9%) and tetrahydrofuran (99.9%), were from Sigma-Aldrich (Steinheim, Germany). Tetrahydrofuran was dried over natrium wire in the presence of benzophenone under a dry nitrogen atmosphere prior to use. Dichloromethane was stored over molecular sieves under nitrogen after being taken to use. All of the following chemicals were obtained from Sigma-Aldrich: boric acid (≥99.5%), hydrochloric acid (37%), magnesium sulfate (≥99.5%), phosphomolybdic acid, sodium bicarbonate (≥99.0%), sodium hydride (60% dispersion in mineral oil), sodium sulfate (≥99%), (*R*)-solketal (98%, 98% ee), (*S*)-solketal (98%, 99% ee), (*S*)-ibuprofen (99%), vinyl dodecanoate (≥99%), palladium on carbon catalyst, perchloric acid (>70%), benzyl bromide (98%), EDCI (1-ethyl-3-(3-dimethylaminopropyl)carbodiimide, >99%) and DMAP (4-dimethylaminopyridine, >99%). Vinyl hexanoate (>99%), vinyl octanoate (>99%), vinyl decanoate (>99%), vinyl tetradecanoate (>99%) and vinyl hexadecanoate (>96%) were purchased from TCI Europe (Zwinderecht, Belgium). The immobilized *Candida antarctica* lipase B (CAL-B, Novozym 435) was obtained as a gift from Novozymes Denmark (Bagsvaerd, Denmark). EPA (98%) and DHA (≥95%) were obtained as ethyl esters from Pronova Biopharma (Sandefjord, Norway) and were hydrolyzed to their corresponding free acids [49]. (*S*)-Naproxen was acquired from Prof. Thorsteinn Loftsson at the Faculty of Pharmaceutical Sciences at the University of Iceland (Reykjavik, Iceland). The silica gel for the chromatography (40–63 μm, 0.060–0.300, F60) was obtained from SiliCycle. The TLC plates were dipped into a methanol solution of phosphomolybdic acid (PMA) to develop the spots.

### 3.2. The Enzymatic Coupling of the SFAs: Synthesis of (R)-***5a*** and (S)-***5a***

For synthesis of (*R*)-**5b**–**f** and (*S*)-**5b**–**f** see Supplementary Materials.

#### 3.2.1. Synthesis of 1-*O*-Benzyl-3-hexanoyl-*sn*-glycerol, (*R*)-**5a**

Immobilized CAL-B (18 mg) was added to a solution of 1-*O*-benzyl-*sn*-glycerol (150 mg, 0.823 mmol) and vinyl hexanoate (134 mg, 0.940 mmol) in CH_2_Cl_2_ (3 mL). The resulting mixture was stirred at room temperature for approx. 90 min when TLC monitoring indicated a complete reaction. The lipase preparation was separated by filtration, and the solvent was removed in vacuo on a rotary evaporator. The concentrate was applied to a 4% boric acid-impregnated flash silica gel chromatography using petroleum ether/ethyl acetate (7:3) as the eluent. This afforded the product (*R*)-**5a** as a colorless liquid in 94% yield (216 mg, 0.770 mmol). ${\left[\alpha \right]}_{D}^{20}$ = −2.29 (c. 10.0, CH_2_Cl_2_). IR (NaCl, ν_max_/cm^−1^): 3459 (br), 2957 (vs), 2930 (vs), 2861 (vs), 1737 (vs). ^1^H NMR (400 MHz, CDCl_3_) δ_H_: 7.38–7.28 (m, 5H, Ph-H), 4.56 (s, 2H, PhCH_2_), 4.19 (dd, *J* = 11.5, 4.5 Hz, 1H, CH_2_ *sn*-3), 4.14 (dd, *J* = 11.5, 6.0 Hz, 1H, CH_2_ *sn*-3), 4.05–4.00 (m, 1H, CH *sn*-2), 3.56 (dd, *J* = 9.6, 4.3 Hz, 1H, CH_2_ *sn*-1), 3.50 (dd, *J* = 9.6, 6.1 Hz, 1H, CH_2_ *sn*-1), 2.48 (bs, 1H, OH), 2.32 (t, *J* = 7.6 Hz, 2H, CH_2_COO), 1.66–1.58 (m, 2H, C*H*_2_CH_2_COO), 1.38–1.24 (m, 4H, CH_2_), 0.89 (t, *J* = 6.9 Hz, 3H, CH_3_) ppm. ^13^C{H} NMR (101 MHz, CDCl_3_) δ_C_: 174.1, 137.8, 128.6 (2), 128.0, 127.9 (2), 73.6, 71.0, 69.1, 65.5, 34.2, 31.4, 24.7, 22.4, 14.0 ppm. HRMS (ESI) *m*/*z*: [M + Na]^+^ calcd for C_16_H_24_O_4_Na 303.1567; found, 303.1557.

#### 3.2.2. Synthesis of 3-*O*-Benzyl-1-hexanoyl-*sn*-glycerol, (*S*)-**5a**

The same procedure was followed as described for (*R*)-**5a** using immobilized CAL-B (6 mg), 3-*O*-benzyl-*sn*-glycerol (50 mg, 0.274 mmol), vinyl hexanoate (44 mg, 0.309 mmol) and CH_2_Cl_2_ (3 mL). Purification on a 4% boric acid-impregnated flash silica gel chromatography using pet. ether/ethyl acetate (7:3) as the eluent afforded the product (*S*)-**5a** as a colorless liquid in 95% yield (73 mg, 0.260 mmol). Spectroscopic data identical to those for (*R*)-**5a** were obtained. ${\left[\alpha \right]}_{D}^{20}$ = +2.01 (c. 3.6, CH_2_Cl_2_). HRMS (ESI) *m*/*z*: [M + Na]^+^ calcd for C_16_H_24_O_4_Na 303.1567; found, 303.1565.

### 3.3. The Coupling of the Active Drugs: Synthesis of (R,S′)-***6a***, (S,S′)-***6a***, (R,S′)-***7a*** and (S,S′)-***7a***

For synthesis of (*R*,*S′*)-**6b**–(*R*,*S′*)-**6f**, (*S*,*S′*)-**6b**–(*S*,*S′*)-**6f**, (*R*,*S′*)-**7b**–(*R*,*S′*)-**7f** and (*S*,*S′*)-**7b**–(*S*,*S′*)-**7f**, see the full experimental details in the Supplementary Materials.

#### 3.3.1. Synthesis of 1-*O*-Benzyl-3-hexanoyl-2-[(*S*)-2-(4-isobutylphenyl)propanoyl]-*sn*-glycerol, (*R*,*S′*)-**6a**

To a solution of 1-*O*-benzyl-3-hexanoyl-*sn*-glycerol (*R*)-**5a** (93 mg, 0.332 mmol) and (*S*)-ibuprofen (83 mg, 0.401 mmol) in CH_2_Cl_2_ (3 mL) were added DMAP (36 mg, 0.292 mmol) and EDCI (68 mg, 0.352 mmol). The solution was stirred on a magnetic stirrer at room temperature for 12 h. The reaction was disconnected by passing the reaction mixture through a short column packed with silica gel by use of Et_2_O/CH_2_Cl_2_ (1:9). The solvent was removed in vacuo on a rotary evaporator. The concentrate was applied to a silica gel chromatography using petroleum ether/ethyl acetate (7:3) as the eluent, which afforded the product (*R*,*S′*)-**6a** as a pale-yellow oil in 80% yield (152 mg, 0.267 mmol). ${\left[\alpha \right]}_{D}^{20}$ = −0.77 (c. 9.7, CH_2_Cl_2_). IR (NaCl, ν_max_/cm^−1^): 3028 (s), 2956 (vs), 2932 (vs), 2869 (vs), 1740 (vs), 1162 (br s). ^1^H NMR (400 MHz, CDCl_3_) δ_H_: 7.34–7.25 (m, 3H, Ph-H), 7.21 (m, 2H, Ibu-2,6 and 2H, Ph-H), 7.07 (d, *J* = 8.1 Hz, 2H, Ibu-3,5), 5.27–5.21 (m, 1H, CH *sn*-2), 4.42–4.34 (m, 1H, CH_2_ *sn*-3 and 2H, PhCH_2_), 4.20 (dd, *J* = 11.9, 6.6 Hz, 1H, CH_2_ *sn*-3), 3.72 (q, *J* = 7.2 Hz, 1H, CHCH_3_), 3.55–3.46 (m, 2H, CH_2_ *sn*-1), 2.43 (d, *J* = 7.2 Hz, 2H, C*H*_2_CH(CH_3_)_2_), 2.26 (t, *J* = 7.5 Hz, 2H, CH_2_COO), 1.83 (nonet, *J* = 6.7 Hz, 1H, C*H*(CH_3_)_2_), 1.64–1.54 (m, 2H, C*H*_2_CH_2_COO), 1.50 (d, *J* = 7.2 Hz, 3H, CHCH_3_), 1.36–1.25 (m, 4H, CH_2_), 0.90 (t, *J* = 6.8 Hz, 3H, CH_2_C*H*_3_), 0.89 (d, *J* = 6.6 Hz, 6H, CH(CH_3_)_2_) ppm. ^13^C{H} NMR (101 MHz, CDCl_3_) δ_C_: 174.0 (Ibu), 173.4 (SFA), 140.5, 137.7, 137.5, 129.3 (2), 128.3 (2), 127.6 (2), 127.5 (2), 127.2, 73.3, 70.6, 68.2, 62.6, 45.1, 45.0, 34.0, 31.2, 30.1, 24.5, 22.4 (2), 22.3, 18.5, 13.9 ppm. HRMS (ESI) *m*/*z*: [M + Na]^+^ calcd for C_29_H_40_O_5_Na 491.2768; found, 491.2764.

#### 3.3.2. Synthesis of 3-*O*-Benzyl-1-hexanoyl-2-[(*S*)-2-(4-isobutylphenyl)propanoyl]-*sn*-glycerol, (*S*,*S′*)-**6a**

The same procedure was followed as described for (*R*,*S′*)-**6a** using 3-*O*-benzyl-1-hexanoyl-*sn*-glycerol (*S*)-**5a** (36 mg, 0.128 mmol), (*S*)-ibuprofen (30 mg, 0.145 mmol), CH_2_Cl_2_ (2 mL), DMAP (14 mg, 0.115 mmol) and EDCI (26 mg, 0.136 mmol). Purification on a silica gel chromatography using pet. ether/ethyl acetate (4:1) as the eluent afforded the product (*S*,*S′*)-**6a** as a pale-yellow oil in 93% yield (56 mg, 0.119 mmol). ${\left[\alpha \right]}_{D}^{20}$ = +22.2 (c. 5.6, CH_2_Cl_2_). IR (NaCl, ν_max_/cm^−1^): 3024 (s), 2957 (vs), 2934 (vs), 2865 (vs), 1743 (vs), 1162 (br s). ^1^H NMR (400 MHz, CDCl_3_) δ_H_: 7.37–7.26 (m, 5H, Ph-H), 7.19 (d, *J* = 8.1 Hz, 2H, Ibu-2,6), 7.06 (d, *J* = 8.1 Hz, 2H, Ibu-3,5), 5.26–5.20 (m, 1H, CH *sn*-2), 4.51 (AB q, *J* = 12.1 Hz, 2H, PhCH_2_), 4.24 (dd, *J* = 11.6, 3.9 Hz, 1H, CH_2_ *sn*-1), 4.12 (dd, *J* = 11.9, 6.8 Hz, 1H, CH_2_ *sn*-1), 3.72 (q, *J* = 7.2 Hz, 1H, C*H*CH_3_), 3.63–3.55 (m, 2H, CH_2_ *sn*-3), 2.43 (d, *J* = 7.2 Hz, 2H, CH_2_CH(CH_3_)_2_), 2.11 (t, *J* = 7.6 Hz, 2H, CH_2_COO), 1.83 (nonet, *J* = 6.7 Hz, 1H, CH(CH_3_)_2_), 1.48 (m, 2H, CH_2_CH_2_COO and 3H, CHCH_3_), 1.33–1.20 (m, 4H, CH_2_), 0.90 (t, *J* = 6.9 Hz, 3H, CH_2_CH_3_), 0.88 (d, *J* = 6.6 Hz, 6H, CH(CH_3_)_2_) ppm. ^13^C{H} NMR (101 MHz, CDCl_3_) δ_C_: 174.2 (Ibu), 173.4 (SFA), 140.6, 137.9, 137.6, 129.4 (2), 128.6 (2), 127.9 (2), 127.7 (2), 127.3, 73.5, 70.6, 68.5, 62.7, 45.3, 45.2, 34.0, 31.4, 30.1, 24.6, 22.5 (2), 22.4, 18.4, 14.1 ppm. HRMS (ESI) *m*/*z*: [M + Na]^+^ calcd for C_29_H_40_O_5_Na 491.2768; found, 491.2764.

#### 3.3.3. Synthesis of 1-*O*-Benzyl-3-hexanoyl-2-[(*S*)-2-(6-methoxynaphthalen-2-yl)propanoyl]-*sn*-glycerol, (*R*,*S′*)-**7a**

To a solution of 1-*O*-benzyl-3-hexanoyl-*sn*-glycerol (*R*)-**5a** (93 mg, 0.332 mmol) and (*S*)-naproxen (92 mg, 0.401 mmol) in CH_2_Cl_2_ (3 mL) were added DMAP (36 mg, 0.292 mmol) and EDCI (68 mg, 0.352 mmol). The solution was stirred on a magnetic stirrer at room temperature for 12 h. The reaction was disconnected by passing the reaction mixture through a short column packed with silica gel by use of Et_2_O/CH_2_Cl_2_ (1:9). The solvent was removed in vacuo on a rotary evaporator. The concentrate was applied to a silica gel chromatography using petroleum ether/ethyl acetate (7:3) as the eluent, which afforded the product (*R*,*S′*)-**7a** as a clear oil in 90% yield (147 mg, 0.299 mmol). ${\left[\alpha \right]}_{D}^{20}$ = −3.9 (c. 10.0, CH_2_Cl_2_). IR (NaCl, ν_max_/cm^−1^): 3062 (s), 3031 (s), 2957 (vs), 2935 (vs), 2871 (vs), 1739 (vs), 1634 (vs), 1174 (br s). ^1^H NMR (400 MHz, CDCl_3_) δ_H_: 7.70–7.65 (m, 3H, H-1,4,8 Nap), 7.41 (dd, *J* = 8.5, 1.8 Hz, 1H, H-3 Nap), 7.24–7.21 (m, 3H, Ph-H), 7.14 (dd, *J* = 8.9, 2.6 Hz, 1H, H-7 Nap), 7.12–7.09 (m, 2H, Ph-H and 1H, Nap-5), 5.27 (dtd, *J* = 6.6, 5.0, 3.7 Hz, 1H, CH *sn*-2), 4.39–4.27 (m, 1H, CH_2_ *sn*-3 and 2H, PhCH_2_), 4.21 (dd, *J* = 11.9, 6.6 Hz, 1H, CH_2_ *sn*-3), 3.91 (s, 3H, OCH_3_), 3.89 (q, *J* = 7.2, 1H, CHCH_3_), 3.54–3.44 (m, 2H, CH_2_ *sn*-1), 2.22 (t, *J* = 7.5 Hz, 2H, CH_2_COO), 1.64–1.54 (m, 2H, CH_2_CH_2_COO and 3H, CHC*H*_3_), 1.33–1.21 (m, 4H, CH_2_), 0.89 (t, *J* = 6.9 Hz, 3H, CH_2_C*H*_3_) ppm. ^13^C{H} NMR (101 MHz, CDCl_3_) δ_C_: 173.9 (Nap), 173.4 (SFA), 157.6, 137.7, 135.4, 133.68, 129.2, 128.9, 128.2 (2), 127.6, 127.4 (2), 127.1, 126.2, 126.0, 118.9, 105.6, 73.2, 70.7, 68.2, 62.6, 55.3, 45.4, 34.0, 31.2, 24.58, 22.3, 18.5, 13.9 ppm. HRMS (ESI) *m*/*z*: [M + Na]^+^ calcd for C_30_H_36_O_6_Na 515.2404; found, 515.2396.

#### 3.3.4. Synthesis of 3-*O*-Benzyl-1-hexanoyl-2-[(*S*)-2-(6-methoxynaphthalen-2-yl)propanoyl]-*sn*-glycerol, (*S*,*S′*)-**7a**

To a solution of 3-*O*-benzyl-1-hexanoyl-*sn*-glycerol (*S*)-**5a** (36 mg, 0.128 mmol) and (*S*)-naproxen (34 mg, 0.147 mmol) in CH_2_Cl_2_ (2.3 mL) were added DMAP (15 mg, 0.121 mmol) and EDCI (28 mg, 0.145 mmol). The solution was stirred on a magnetic stirrer at room temperature for 16 h. The reaction was disconnected by passing the reaction mixture through a short column packed with silica gel by use of Et_2_O/CH_2_Cl_2_ (1:9). The solvent was removed in vacuo on a rotary evaporator. The concentrate was applied to a silica gel chromatography using petroleum ether/ethyl acetate (8.5:1.5) as the eluent, which afforded the product (*S*,*S′*)-**7a** as a clear oil in 97% yield (58 mg, 0.124 mmol). ${\left[\alpha \right]}_{D}^{20}$ = +20.8 (c. 3.4, CH_2_Cl_2_). IR (NaCl, ν_max_/cm^−1^): 3058 (s), 3028 (s), 2956 (vs), 2932 (vs), 2855 (vs), 1742 (vs), 1635 (vs), 1170 (br s). ^1^H NMR (400 MHz, CDCl_3_) δ_H_: 7.73–7.63 (m, 3H, H-1,4,8 Nap), 7.41 (dd, *J* = 8.6, 1.8 Hz, 1H, H-3 Nap), 7.36–7.26 (m, 5H, Ph-H), 7.14 (dd, *J* = 8.9, 2.5 Hz, 1H, H-7 Nap), 7.10 (d, *J* = 2.5 Hz, 1H, Nap-5) 5.26–5.20 (m, 1H, CH *sn*-2), 4.51 (AB q, *J* = 12.1 Hz, 2H, PhCH_2_), 4.23 (dd, *J* = 11.9, 3.7 Hz, 1H, CH_2_ *sn*-1), 4.13 (dd, *J* = 11.9, 6.9 Hz, 1H, CH_2_ *sn*-1), 3.91 (s, 3H, OCH_3_), 3.72 (q, *J* = 7.1, 1H, C*H*CH_3_), 3.61–3.59 (m, 2H, CH_2_ *sn*-3), 1.94–1.87 (m, 2H, CH_2_COO), 1.58 (d, *J* = 7.2 Hz, 3H, CHC*H*_3_), 1.40–1.31 (m, 2H, C*H*_2_CH_2_COO), 1.23–1.15 (m, 2H, C*H*_2_CH_2_CH_3_), 1.13–1.03 (m, 2H, C*H*_2_CH_3_), 0.85 (t, *J* = 7.2 Hz, 3H, CH_2_C*H*_3_) ppm. ^13^C{H} NMR (101 MHz, CDCl_3_) δ_C_: 174.1 (Nap), 173.4 (SFA), 157.8, 137.8, 135.6, 133.8, 129.4, 129.1, 128.5 (2), 127.9, 127.7 (2), 127.2, 126.4, 126.1, 119.1, 105.7, 73.4, 70.7, 68.5, 62.6, 55.4, 45.6, 33.8, 31.3, 24.4, 22.4, 18.5, 14.0 ppm. HRMS (ESI) *m*/*z*: [M + Na]^+^ calcd for C_30_H_36_O_6_Na 515.2404; found, 515.2405.

### 3.4. The Removal of the Benzyl Protective Group: Synthesis of (R,S′)-***8a***, (S,S′)-***8a***, (R,S′)-***9a*** and (S,S′)-***9a***

For synthesis of (*R*,*S′*)-**8b**–(*R*,*S′*)-**8f**, (*S*,*S′*)-**8b**–(*S*,*S′*)-**8f**, (*R*,*S′*)-**9b**–(*R*,*S′*)-**9f** and (*S*,*S′*)-**9b**–(*S*,*S′*)-**9f**, see the full experimental details in the Supplementary Materials.

#### 3.4.1. Synthesis of 3-Hexanoyl-2-[(*S*)-2-(4-isobutylphenyl)propanoyl]-*sn*-glycerol, (*R*,*S′*)-**8a**

Pd/C catalyst (26 mg) was placed into a 25 mL flame-dried two-necked round-bottom flask equipped with a magnetic stirrer under a nitrogen atmosphere at room temperature, and the flask was sealed with a septum. A solution of 1-*O*-benzyl-3-hexanoyl-2-[(*S*)-2-(4-isobutylphenyl)propanoyl]-*sn*-glycerol, (*R*,*S′*)-**6a** (116 mg, 0.236 mmol) dissolved in dry THF (7 mL) was added with a syringe, followed by n-hexane (11.2 mL). A balloon filled with hydrogen gas was then mounted on a syringe and stuck through the septum. The mixture was stirred while the hydrogen gas was blown through the flask to replace the nitrogen atmosphere with hydrogen. Then, a tiny drop of perchloric acid was added, and the solution was stirred vigorously at room temperature while being monitored with TLC. When the reaction came to completion according to the TLC (approximately 15 min), the flask was promptly opened, and the acid was neutralized by adding NaHCO_3_ (s). Then, the solution was filtered, and the solvent was removed in vacuo on a rotary evaporator. The crude product was applied to a 4% boric acid-impregnated flash silica gel chromatography using petroleum ether/ethyl acetate (7:3) as the eluent, which afforded the product (*R*,*S′*)-**8a** as a pale-yellow oil in 84% yield (75 mg, 0.198 mmol). ${\left[\alpha \right]}_{D}^{20}$ = +23.9 (c. 4.0, CH_2_Cl_2_). IR (NaCl, ν_max_/cm^−1^): 3500 (br), 2956 (vs), 2932 (vs), 2870 (vs), 1739 (vs), 1165 (br s). ^1^H NMR (400 MHz, CDCl_3_) δ_H_: 7.19 (d, *J* = 8.2 Hz, 2H, Ibu-2,6), 7.10 (d, *J* = 8.1 Hz, 2H, Ibu-3,5), 5.07–5.02 (m, 1H, CH *sn*-2), 4.30 (dd, *J* = 11.9, 4.4 Hz, 1H, CH_2_ *sn*-3), 4.21 (dd, *J* = 11.9, 5.9 Hz, 1H, CH_2_ *sn*-3), 3.73 (q, *J* = 7.1 Hz, 1H, C*H*CH_3_), 3.61–3.56 (m, 2H, CH_2_ *sn*-1), 2.44 (d, *J* = 7.1 Hz, 2H, C*H*_2_CH(CH_3_)_2_), 2.29 (t, *J* = 7.6 Hz, 2H, CH_2_COO), 1.84 (nonet, *J* = 6.7 Hz, 1H, C*H*(CH_3_)_2_), 1.67–1.57 (m, 2H, C*H*_2_CH_2_COO), 1.50 (d, *J* = 7.2 Hz, 3H, CHC*H*_3_), 1.34–1.27 (m, 4H, CH_2_), 0.90 (t, *J* = 6.1 Hz, 3H, CH_2_C*H*_3_), 0.88 (d, *J* = 6.6 Hz, 6H, CH(C*H*_3_)_2_) ppm. ^13^C{H} NMR (101 MHz, CDCl_3_) δ_C_: 174.3 (Ibu), 173.8 (SFA), 140.9, 137.8, 129.6 (2), 127.1 (2), 72.7, 62.1, 61.5, 45.3, 45.1, 34.2, 31.4, 30.3, 24.7, 22.5 (2), 22.4, 18.4, 14.1 ppm. HRMS (ESI) *m*/*z*: [M + Na]^+^ calcd for C_22_H_34_O_5_Na 401.2298; found, 401.2297.

#### 3.4.2. Synthesis of 1-Hexanoyl-2-[(*S*)-2-(4-isobutylphenyl)propanoyl]-*sn*-glycerol, (*S*,*S′*)-**8a**

The same procedure was followed as described for (*R*,*S′*)-**8a** using Pd/C catalyst (13 mg), 3-*O*-benzyl-1-hexanoyl-2-[(*S*)-2-(4-isobutylphenyl)propanoyl]-*sn*-glycerol (*S*,*S′*)-**6a** (30 mg, 0.064 mmol), THF (4 mL) and n-hexane (5.6 mL). Purification on 4% boric acid-impregnated flash silica gel chromatography using pet. ether/ethyl acetate (3:2) as the eluent afforded the product (*S*,*S′*)-**8a** as a pale-yellow oil in 95% yield (23 mg, 0.061 mmol). ${\left[\alpha \right]}_{D}^{20}$ = +5.65 (c. 2.3, CH_2_Cl_2_). IR (NaCl, ν_max_/cm^−1^): 3474 (br), 2956 (vs), 2870 (vs), 1740 (vs), 1513 (vs), 1165 (br s). ^1^H NMR (400 MHz, CDCl_3_) δ_H_: 7.19 (d, *J* = 8.2 Hz, 2H, Ibu-2,6), 7.10 (d, *J* = 8.1 Hz, 2H, Ibu-3,5), 5.07–5.02 (m, 1H, CH *sn*-2), 4.30 (dd, *J* = 11.9, 4.4 Hz, 1H, CH_2_ *sn*-1), 4.14 (dd, *J* = 11.9, 6.0 Hz, 1H, CH_2_ *sn*-1), 3.72–3.63 (m, 2H, CH_2_ *sn*-3 and 1H, CHCH_3_), 2.44 (d, *J* = 7.1 Hz, 2H, CH_2_CH(CH_3_)_2_), 2.18 (t, *J* = 7.6 Hz, 2H, CH_2_COO), 1.91 (t, *J* = 6.5 Hz, 1H, OH), 1.84 (nonet, *J* = 6.8 Hz, 1H, CH(CH_3_)_2_), 1.55 (m, 2H, CH_2_CH_2_COO), 1.50 (d, *J* = 7.2 Hz, 3H, CHCH_3_), 1.34–1.27 (m, 4H, CH_2_), 0.90 (t, *J* = 6.1 Hz, 3H, CH_2_CH_3_), 0.88 (d, *J* = 6.6 Hz, 6H, CH(CH_3_)_2_) ppm. ^13^C{H} NMR (101 MHz, CDCl_3_) δ_C_: 174.5 (Ibu), 173.8 (SFA), 140.8, 137.4, 129.5 (2), 127.2 (2), 72.7, 62.0, 61.7, 45.2, 45.2, 34.1, 31.4, 30.3, 24.6, 22.5 (2), 22.4, 18.4, 14.0 ppm. HRMS (ESI) *m*/*z*: [M + Na]^+^ calcd for C_22_H_34_O_5_Na 401.2298; found, 401.2295.

#### 3.4.3. Synthesis of 3-Hexanoyl-2-[(*S*)-2-(6-methoxynaphthalen-2-yl)propanoyl]-*sn*-glycerol, (*R*,*S′*)-**9a**

The Pd/C catalyst (24 mg) was placed into a 25 mL flame-dried two-necked round-bottom flask equipped with a magnetic stirrer under a nitrogen atmosphere at room temperature, and the flask was sealed with a septum. A solution of 1-*O*-benzyl-3-hexanoyl-2-[(*S*)-2-(6-methoxynaphthalen-2-yl)propanoyl]-*sn*-glycerol, (*R*,*S′*)-**7a** (107 mg, 0.217 mmol) dissolved in dry THF (6.4 mL) was added with a syringe, followed by n-hexane (10.3 mL). A balloon filled with hydrogen gas was then mounted on a syringe and stuck through the septum. The mixture was stirred while the hydrogen gas was blown through the flask to replace the nitrogen atmosphere with hydrogen. Then, a tiny drop of perchloric acid was added, and the solution was stirred vigorously at room temperature while being monitored with TLC. When the reaction came to completion according to the TLC (approximately 15 min), the flask was promptly opened, and the acid was neutralized by adding NaHCO_3_ (s). Then, the solution was filtered, and the solvent was removed in vacuo on a rotary evaporator. The crude product was applied to a 4% boric acid-impregnated flash silica gel chromatography using petroleum ether/ethyl acetate (1:1) as the eluent, which afforded the product (*R*,*S′*)-**9a** as a pale-yellow oil in 98% yield (86 mg, 0.213 mmol). ${\left[\alpha \right]}_{D}^{20}$ = +5.63 (c. 1.6, CH_2_Cl_2_). IR (NaCl, ν_max_/cm^−1^): 3358 (br), 2926 (vs), 2856 (vs), 1739 (vs), 1632 (vs). 1606 (vs). ^1^H NMR (400 MHz, CDCl_3_) δ_H_: 7.71–7.66 (m, 3H, Nap-1,4,8), 7.38 (dd, *J* = 8.5, 1.9 Hz, 1H, Nap-3), 7.14 (dd, *J* = 8.9, 2.5 Hz, 1H, Nap-7), 7.10 (d, *J* = 2.5 Hz, 1H, Nap-5), 5.14–5.01 (m, 1H, CH *sn*-2), 4.31 (dd, *J* = 11.9, 4.3 Hz, 1H, CH_2_ *sn*-3), 4.27–4.19 (m, 1H, CH_2_ *sn*-3), 3.91 (s, 3H, OCH_3_), 3.91–3.82 (m, 1H, C*H*CH_3_), 3.61–3.57 (m, 2H, CH_2_ *sn*-1), 2.27–2.24 (m, 2H, CH_2_COO), 2.23–2.20 (bs, 1H, OH), 1.58 (d, *J* = 7.2 Hz, 3H, CHC*H*_3_), 1.56 (quint, *J* = 7.2 Hz, 2H, C*H*_2_CH_2_COO), 1.38–1.22 (m, 4H, CH_2_), 0.89 (t, *J* = 7.0 Hz, 3H, CH_2_C*H*_3_) ppm. ^13^C{H} NMR (101 MHz, CDCl_3_) δ_C_: 174.3 (Nap), 173.8 (SFA), 157.9, 135.6, 133.9, 129.4, 129.0, 127.4, 126.1, 126.0, 119.3, 105.8, 72.8, 62.1, 61.5, 55.5, 45.6, 34.2, 31.1, 24.7, 22.4, 18.5, 14.0 ppm. HRMS (ESI) *m*/*z*: [M + Na]^+^ calcd for C_23_H_30_O_6_Na 425.1935; found, 425.1934.

#### 3.4.4. Synthesis of 1-Hexanoyl-2-[(*S*)-2-(6-methoxynaphthalen-2-yl)propanoyl]-*sn*-glycerol, (*S*,*S′*)-**9a**

The same procedure was followed as described for (*R*,*S′*)-**9a** using Pd/C catalyst (3 mg), 3-*O*-benzyl-1-hexanoyl-2-[(*S*)-2-(6-methoxynaphthalen-2-yl)propanoyl]-*sn*-glycerol, (*S*,*S′*)-**7a** (14 mg, 0.028 mmol), THF (1 mL) and n-hexane (1.5 mL). Purification on 4% boric acid-impregnated flash silica gel chromatography using pet. ether/ethyl acetate (3:2) as the eluent afforded the product (*S*,*S′*)-**9a** as a pale-yellow oil in 97% yield (11 mg, 0.027 mmol). ${\left[\alpha \right]}_{D}^{20}$ = +8.60 (c. 1.0, CH_2_Cl_2_). IR (NaCl, ν_max_/cm^−1^): 3421 (br), 3060 (s), 2925 (vs), 2856 (vs), 1739 (vs), 1634 (s), 1607 (vs), 1162 (br s). ^1^H NMR (400 MHz, CDCl_3_) δ_H_: 7.73–7.65 (m, 3H, Nap-1,4,8), 7.39 (dd, *J* = 8.5, 1.9 Hz, 1H, Nap-3), 7.14 (dd, *J* = 8.9, 2.6 Hz, 1H, Nap-7), 7.10 (d, *J* = 2.6 Hz, 1H, Nap-5), 5.08 (m, 1H, CH *sn*-2), 4.19 (dd, *J* = 11.9, 4.4 Hz, 1H, CH_2_ *sn*-1), 4.27–4.13 (dd, *J* = 11.9, 6.1, Hz, 1H, CH_2_ *sn*-1), 3.91 (s, 3H, OCH_3_), 3.96–3.84 (m, 1H, CHCH_3_), 3.74–3.69 (m, 2H, CH_2_ *sn*-3), 2.23–2.20 (bs, 1H, OH), 2.05–1.89 (m, 2H, CH_2_COO), 1.59 (d, *J* = 7.1 Hz, 3H, CHCH_3_), 1.44–1.33 (m, 2H, CH_2_CH_2_COO), 1.38–1.06 (m, 4H, CH_2_), 0.85 (t, *J* = 7.2 Hz, 3H, CH_2_CH_3_) ppm. ^13^C{H} NMR (101 MHz, CDCl_3_) δ_C_: 174.5 (Nap), 173.7 (SFA), 157.9, 135.4, 133.9, 129.4, 129.1, 127.3, 126.2, 126.1, 119.2, 105.7, 72.8, 61.9, 61.7, 55.5, 45.6, 33.9, 31.3, 29.9, 24.5, 18.5, 14.0 ppm. HRMS (ESI) *m*/*z*: [M + Na]^+^ calcd for C_23_H_30_O_6_Na 425.1935; found, 425.1932.

### 3.5. Coupling of EPA: Synthesis of (S,S′)-***10a***, (R,S′)-***10a***, (S,S′)-***11a*** and (R,S′)-***11a***

For synthesis of (*S*,*S′*)-**10b**–(*S*,*S′*)-**10f**, (*R*,*S′*)-**10b**–(*R*,*S′*)-**10f**, (*S*,*S′*)-**11b**–(*S*,*S′*)-**11f** and (*R*,*S′*)-**11b**–(*R*,*S′*)-**11f**, see the full experimental details in the Supplementary Materials.

#### 3.5.1. Synthesis of 1-[5*Z*,8*Z*,11*Z*,14*Z*,17*Z*)-Eicosa-5,8,11,14,17-pentaenoyl]-3-hexanoyl-2-[(*S*)-2-(4-isobutylphenyl)propanoyl]-*sn*-glycerol, (*S*,*S′*)-**10a**

To a solution of 3-hexanoyl-2-[(*S*)-2-(4-isobutylphenyl)propanoyl]-*sn*-glycerol (*R*,*S′*)-**8a** (45 mg, 0.118 mmol) and EPA as a free acid (33 mg, 0.108 mmol) in CH_2_Cl_2_ (4 mL) were added DMAP (13 mg, 0.106 mmol) and EDCI (28 mg, 0.143 mmol). The solution was stirred on a magnetic stirrer at room temperature for 12 h. The reaction was disconnected by passing the reaction mixture through a short column packed with silica gel by use of Et_2_O/CH_2_Cl_2_ (1:9). The solvent was removed in vacuo on a rotary evaporator. The residue was applied to a silica gel chromatography using petroleum ether/ethyl acetate (8.5:1.5) as the eluent, which afforded the product (*S*,*S′*)-**10a** as a yellow oil in 90% yield (70 mg, 0.106 mmol). ${\left[\alpha \right]}_{D}^{20}$ = +11.7 (c. 6.0, CH_2_Cl_2_). IR (NaCl, ν_max_/cm^−1^): 3013 (s), 2959 (vs), 2933 (vs), 2871 (vs), 1743 (vs), 1656 (s). ^1^H NMR (400 MHz, CDCl_3_) δ_H_: 7.18 (d, *J* = 8.1 Hz, 2H, Ibu-2,6), 7.07 (d, *J* = 8.1 Hz, 2H, Ibu-3,5), 5.44–5.28 (m, 10H, =CH), 5.35–5.32 (m, 1H, CH *sn*-2), 4.30 (dd, *J* = 11.9, 4.2 Hz, 1H, CH_2_ *sn*-1/3), 4.19 (dd, *J* = 11.9, 4.5 Hz, 1H, CH_2_ *sn*-1/3), 4.13 (dd, J = 11.9, 6.0 Hz, 1H, CH_2_ *sn*-1/3), 4.07 (dd, *J* = 11.9, 6.4 Hz, 1H, CH_2_ *sn*-1/3), 3.70 (q, *J* = 7.1 Hz, 1H, CHCH_3_), 2.86–2.78 (m, 8H, =CHCH_2_CH=), 2.43 (d, *J* = 7.2 Hz, 2H, CH_2_CH(CH_3_)_2_), 2.28 (t, *J* = 7.2 Hz, 2H, CH_2_COO EPA), 2.18 (t, *J* = 7.5 Hz, 2H, CH_2_COO), 2.10–2.05 (m, 4H, CH_2_CH_2_CH= and =CHCH_2_CH3), 1.84 (nonet, *J* = 6.8 Hz, 1H, CH(CH_3_)_2_), 1.63–1.48 (m, 4H, CH_2_CH_2_COO SFA and CH_2_CH_2_COO EPA), 1.49 (d, *J* = 7.2 Hz, 3H, CHCH_3_), 1.32–1.29 (m, 4H, CH_2_), 0.97 (t, *J* = 7.5 Hz, 3H, CH_3_ EPA), 0.90 (t, *J* = 7.2 Hz, 3H, CH_3_ SFA), 0.89 (d, *J* = 6.6 Hz, 6H, CH(CH_3_)_2_) ppm. ^13^C{H} NMR (101 MHz, CDCl_3_) δ_C_: 173.9 (Ibu), 173.4 (SFA), 173.0 (EPA), 140.7, 137.4, 132.2, 129.4 (2), 129.0, 128.71, 128.4, 128.3, 128.3, 128.2, 128.0, 128.3, 127.3 (2), 127.2, 69.3, 62.2, 62.1, 45.2, 45.2, 34.1, 33.4, 31.4, 30.3, 26.6, 25.8 (2), 25.7 (2), 24.7, 24.7, 22.5 (2), 22.4, 20.7, 18.5, 14.4, 14.0 ppm. HRMS (ESI) *m*/*z*: [M + Na]^+^ calcd for C_42_H_62_O_6_Na 685.4439; found, 685.4439.

#### 3.5.2. Synthesis of 3-[5*Z*,8*Z*,11*Z*,14*Z*,17*Z*)-Eicosa-5,8,11,14,17-pentaenoyl]-1-hexanoyl-2-[(*S*)-2-(4-isobutylphenyl)propanoyl]-*sn*-glycerol, (*R*,*S′*)-**10a**

The same procedure was followed as described for (*S*,*S′*)-**10a** using 1-hexanoyl-[(*S*)-2-(4-isobutylphenyl)propanoyl]-*sn*-glycerol (*S*,*S′*)-**8a** (19 mg, 0.050 mmol), EPA (16 mg, 0.053 mmol), CH_2_Cl_2_ (3 mL), DMAP (7 mg, 0.054 mmol) and EDCI (14 mg, 0.073 mmol). Purification on a silica gel chromatography using pet. ether/ethyl acetate (8.5:1.5) as the eluent afforded the product (*R*,*S′*)-**10a** as a pale-yellow oil in 74% yield (25 mg, 0.037 mmol). ${\left[\alpha \right]}_{D}^{20}$ = +6.50 (c. 2.0, CH_2_Cl_2_). ^1^H NMR (400 MHz, CDCl_3_) δ_H_: 7.18 (dd, *J* = 8.1, 1.7 Hz, 2H, Ibu-2,6), 7.07 (dd, *J* = 8.1, 3.8 Hz, 2H, Ibu-3,5), 5.45–5.29 (m, 10H, =CH), 5.28–5.22 (m, 1H, CH *sn*-2), 4.30 (dd, *J* = 11.9, 4.3 Hz, 1H, CH_2_ *sn*-1/3), 4.21 (dd, *J* = 11.9, 4.3 Hz, 1H, CH_2_ *sn*-1/3), 4.15 (dd, J = 11.9, 6.4 Hz, 1H, CH_2_ *sn*-1/3), 4.06 (dd, *J* = 11.9, 6.3 Hz, 1H, CH_2_ *sn*-1/3), 3.70 (q, *J* = 7.1 Hz, 1H, CHCH_3_), 2.87–2.77 (m, 8H, =CHCH_2_CH=), 2.44 (d, *J* = 7.2 Hz, 2H, CH_2_CH(CH_3_)_2_), 2.33–2.22 (m, 2H, CH_2_COO EPA), 2.18–2.04 (m, 6H, CH_2_COO SFA, CH_2_CH_2_CH= and =CHCH_2_CH_3_), 1.84 (nonet, *J* = 6.8 Hz, 1H, C*H*(CH_3_)_2_), 1.74–1.58 (m, 2H, CH_2_CH_2_COO EPA), 1.57–1.50 (m, 2H, CH_2_CH_2_COO SFA), 1.49 (d, *J* = 7.2 Hz, 3H, CHC*H*_3_), 1.33–1.20 (m, 4H, CH_2_), 0.97 (t, *J* = 7.5 Hz, 3H, CH_3_ EPA), 0.89 (d, *J* = 6.6 Hz, 6H, CH(C*H*_3_)_2_), 0.88 (t, *J* = 6.5 Hz, 3H, CH_3_ SFA) ppm. ^13^C{H} NMR (101 MHz, CDCl_3_) δ_C_: 173.9 (Ibu), 173.3 (SFA), 173.1 (EPA), 140.7, 137.4, 132.2, 129.4 (2), 129.1, 129.0, 128.7, 128.4, 128.4, 128.3, 128.2, 128.0, 127.3 (2), 127.2, 69.4, 62.3, 62.1, 45.2, 45.2, 34.0, 33.5, 31.4, 30.3, 26.7, 25.8 (2), 25.7 (2), 24.8, 24.6, 22.5 (2), 22.4, 20.7, 18.5, 14.4, 14.0 ppm. HRMS (ESI) *m*/*z*: [M + Na]^+^ calcd for C_42_H_62_O_6_Na 685.4439; found, 685.4430.

#### 3.5.3. Synthesis of 1-[5*Z*,8*Z*,11*Z*,14*Z*,17*Z*)-Eicosa-5,8,11,14,17-pentaenoyl]-3-hexanoyl-2[(*S*)-2-(6-methoxynaphthalen-2-yl)propanoyl]-*sn*-glycerol, (*S*,*S′*)-**11a**

To a solution of 3-hexanoyl-2-[(*S*)-2-(6-methoxynaphthalen-2-yl)propanoyl]-*sn*-glycerol (*R*,*S′*)-**9a** (35 mg, 0.087 mmol) and EPA as a free acid (28 mg, 0.093 mmol) in CH_2_Cl_2_ (4 mL) were added DMAP (11 mg, 0.094 mmol) and EDCI (24 mg, 0.127 mmol). The solution was stirred on a magnetic stirrer at room temperature for 12 h. The reaction was disconnected by passing the reaction mixture through a short column packed with silica gel by use of Et_2_O/CH_2_Cl_2_ (1:9). The solvent was removed in vacuo on a rotary evaporator. The residue was applied to a silica gel chromatography using petroleum ether/ethyl acetate (7:3) as the eluent, which afforded the product (*S*,*S′*)-**11a** as a yellow oil in 91% yield (54 mg, 0.079 mmol). ${\left[\alpha \right]}_{D}^{20}$ = +8.12 (c. 5.0, CH_2_Cl_2_). IR (NaCl, ν_max_/cm^−1^): 3012 (s), 2960 (vs), 2933 (vs), 2872 (vs), 1735 (vs), 1634 (vs), 1607 (vs). ^1^H NMR (400 MHz, CDCl_3_) δ_H_: 7.72–7.65 (m, 3H, Nap-1,4,8), 7.38 (dd, *J* = 8.5, 1.9 Hz, 1H, Nap-3), 7.14 (dd, *J* = 8.9, 2.5 Hz, 1H, Nap-7), 7.10 (d, *J* = 2.5 Hz, 1H, Nap-5), 5.46–5.31 (m, 10H, =CH), 5.33–5.19 (m, 1H, CH *sn*-2), 4.30 (dd, *J* = 11.9, 4.2 Hz, 1H, CH_2_ *sn*-1/3), 4.20–4.10 (m, 2H, CH_2_ *sn*-1/3), 4.07 (dd, *J* = 11.9, 6.4 Hz, 1H, CH_2_ *sn*-1/3), 3.90 (s, 3H, OCH_3_), 3.85 (q, *J* = 7.5 Hz, 1H, CHCH_3_), 2.87–2.71 (m, 8H, =CHCH_2_CH=), 2.30–2.19 (m, 2H, CH_2_COO EPA), 2.12–2.04 (m, 2H, CH_2_COO SFA), 2.00 (td, *J* = 7.6, 5.6 Hz, 2H, CH_2_CH_2_CH=), 1.96–1.90 (m, 2H, =CHCH_2_CH_3_), 1.62–1.52 (m, 5H, CH_2_CH_2_COO SFA and CHCH_3_), 1.46 (quint, *J* = 7.3 Hz, 2H, CH_2_CH_2_COO EPA), 1.35–1.19 (m, 4H, CH_2_), 0.97 (t, *J* = 7.5 Hz, 3H, CH_3_ EPA), 0.89 (t, *J* = 7.0 Hz, 3H, CH_3_ SFA) ppm. ^13^C{H} NMR (101 MHz, CDCl_3_) δ_C_: 173.8 (Nap), 173.3 (SFA), 172.9 (EPA), 157.8, 135.3, 133.8, 132.1, 129.4, 129.0, 128.93, 128.89, 128.7, 128.4, 128.3, 128.3, 128.2, 128.0, 127.2, 127.1, 126.2, 126.1, 119.1, 105.6, 69.5, 62.1, 62.1, 55.4, 45.5, 34.1, 33.2, 31.3, 26.5, 25.7, 25.7 (2), 25.7, 24.6, 24.5, 22.4, 20.7, 18.4, 14.4, 14.0 ppm. HRMS (ESI) *m*/*z*: [M + Na]^+^ calcd for C_43_H_58_O_7_Na 709.4075; found, 709.4062.

#### 3.5.4. Synthesis of 3-[5*Z*,8*Z*,11*Z*,14*Z*,17*Z*)-Eicosa-5,8,11,14,17-pentaenoyl]-1-hexanoyl-2[(*S*)-2-(6-methoxynaphthalen-2-yl)propanoyl]-*sn*-glycerol, (*R*,*S′*)-**11a**

To a solution of 1-hexanoyl-2-[(*S*)-2-(6-methoxynaphthalen-2-yl)propanoyl]-*sn*-glycerol (*S*,*S′*)-**9a** (12 mg, 0.030 mmol) and EPA as a free acid (10 mg, 0.033 mmol) in CH_2_Cl_2_ (1.3 mL) were added DMAP (4 mg, 0.033 mmol) and EDCI (8 mg, 0.044 mmol). The solution was stirred on a magnetic stirrer at room temperature for 12 h. The reaction was disconnected by passing the reaction mixture through a short column packed with silica gel by use of Et_2_O/CH_2_Cl_2_ (1:9). The solvent was removed in vacuo on a rotary evaporator. The residue was applied to a silica gel chromatography using petroleum ether/ethyl acetate (7:3) as the eluent, which afforded the product (*R*,*S′*)-**11a** as a yellow oil in 80% yield (16 mg, 0.024 mmol). ${\left[\alpha \right]}_{D}^{20}$ = +4.20 (c. 1.0, CH_2_Cl_2_). IR (NaCl, ν_max_/cm^−1^): 3059 (br), 3012 (s), 2958 (vs), 2872 (vs), 1740 (vs, 1634 (s), 1607 (s). ^1^H NMR (400 MHz, CDCl_3_) δ_H_: 7.72–7.63 (m, 3H, Nap-1,4,8), 7.38 (dd, *J* = 8.5, 1.8 Hz, 1H, Nap-3), 7.14 (dd, *J* = 8.9, 2.4 Hz, 1H, Nap-7), 7.10 (d, *J* = 2.4 Hz, 1H, Nap-5), 5.45–5.31 (m, 10H, =CH), 5.33–5.19 (m, 1H, CH *sn*-2), 4.30 (dd, *J* = 11.9, 4.2 Hz, 1H, CH_2_ *sn*-1/3), 4.17 ((dd, *J* = 8.2, 3.7 Hz, 2H, CH_2_ *sn*-1/3), 4.14 (dd, *J* = 8.2, 3.6 Hz, 1H, CH_2_ *sn*-1/3), 4.06 (dd, *J* = 11.9, 6.4 Hz, 1H, CH_2_ *sn*-1/3), 3.91 (s, 3H, OCH_3_), 3.86 (q, *J* = 7.1 Hz, 1H, C*H*CH_3_), 2.80–2.76 (m, 8H, =CHCH_2_CH=), 2.29–2.23 (m, 2H, CH_2_COO EPA), 2.12–2.03 (m, 4H, CH_2_COO SFA and CH_2_CH_2_CH=), 1.97–1.91 (m, 2H, =CHCH_2_CH_3_), 1.65 (quint, *J* = 7.4 Hz, 2H, CH_2_CH_2_COO SFA), 1.57 (d, *J* = 7.1 Hz, CHCH_3_), 1.44–1.33 (m, 2H, CH_2_CH_2_COO EPA), 1.32–1.07 (m, 4H, CH_2_), 0.97 (t, *J* = 7.6 Hz, 3H, CH_3_ EPA), 0.89 (t, *J* = 7.2 Hz, 3H, CH_3_ SFA) ppm. ^13^C{H} NMR (101 MHz, CDCl_3_) δ_C_: 173.9 (Nap), 173.3 (SFA), 173.1 (EPA), 157.9, 135.4, 133.9, 132.2, 131.0, 129.4, 129.08, 129.05, 129.0, 128.9, 128.7, 128.4, 128.4, 128.2, 128.0, 127.24, 127.17, 126.3, 126.1, 119.2, 105.7, 69.5, 62.3, 62.0, 55.4, 45.5, 34.1, 33.2, 31.3, 26.5, 25.8, 25.70 (2), 25.66, 24.8, 24.6, 22.4, 20.7, 18.5, 14.4, 14.0 ppm. HRMS (ESI) *m*/*z*: [M + Na]^+^ calcd for C_43_H_58_O_7_Na 709.4075; found, 709.4075.

### 3.6. Coupling of DHA: Synthesis of (S,S′)-***12a***, (R,S′)-***12a***, (S,S′)-***13a*** and (R,S′)-***13a***

For synthesis of (*S*,*S′*)-**12b**–(*S*,*S′*)-**12f**, (*R*,*S′*)-**12b**–(*R*,*S′*)-**12f**, (*S*,*S′*)-**13b**–(*S*,*S′*)-**13f** and (*R*,*S′*)-**13b**–(*R*,*S′*)-**13f**, see the full experimental details in the Supplementary Materials.

#### 3.6.1. Synthesis of 1-[4*Z*,7*Z*,10*Z*,13*Z*,16*Z*,19*Z*)-Docosa-4,7,10,13,16,19-hexaenoyl]-3-hexanoyl-2-[(*S*)-2-(4-isobutylphenyl)propanoyl]-*sn*-glycerol, (*S*,*S′*)-**12a**

To a solution of 3-hexanoyl-2-[(*S*)-2-(4-isobutylphenyl)propanoyl]-*sn*-glycerol (*R*,*S′*)-**8a** (23 mg, 0.061 mmol) and DHA as a free acid (36 mg, 0.108 mmol) in CH_2_Cl_2_ (4 mL) were added DMAP (13 mg, 0.106 mmol) and EDCI (28 mg, 0.143 mmol). The solution was stirred on a magnetic stirrer at room temperature for 12 h. The reaction was disconnected by passing the reaction mixture through a short column packed with silica gel by use of Et_2_O/CH_2_Cl_2_ (1:9). The solvent was removed in vacuo on a rotary evaporator. The residue was applied to a silica gel chromatography using petroleum ether/ethyl acetate (8.5:1.5) as the eluent, which afforded the product (*S*,*S′*)-**12a** as a yellow oil in 81% yield (35 mg, 0.049 mmol). ${\left[\alpha \right]}_{D}^{20}$ = +9.95 (c. 2.0, CH_2_Cl_2_). IR (NaCl, ν_max_/cm^−1^): 3013 (vs), 2957 (vs), 2928 (vs), 2870 (vs), 1743 (vs). ^1^H NMR (400 MHz, CDCl_3_) δ_H_: 7.18 (d, *J* = 8.1 Hz, 2H, Ibu-2,6), 7.07 (d, *J* = 8.1 Hz, 2H, Ibu-3,5), 5.44–5.28 (m, 12H, =CH), 5.35–5.32 (m, 1H, CH *sn*-2), 4.30 (dd, *J* = 11.9, 4.2 Hz, 1H, CH_2_ *sn*-1/3), 4.19 (dd, *J* = 11.9, 4.5 Hz, 1H, CH_2_ *sn*-1/3), 4.13 (dd, *J* = 11.9, 6.0 Hz, 1H, CH_2_ *sn*-1/3), 4.08 (dd, *J* = 11.9, 6.4 Hz, 1H, CH_2_ *sn*-1/3), 3.70 (q, *J* = 7.2 Hz, 1H, CHCH_3_), 2.90–2.78 (m, 10H, =CHCH_2_CH=), 2.43 (d, *J* = 7.2 Hz, 2H, CH_2_CH(CH_3_)_2_), 2.34–2.19 (m 6H, CH_2_CH_2_COO DHA and CH_2_COO SFA), 2.10–2.06 (m, 2H, =CHCH_2_CH_3_), 1.83 (nonet, *J* = 6.8 Hz, 1H, CH(CH_3_)_2_), 1.65–1.55 (m, 2H, CH_2_CH_2_COO SFA), 1.49 (d, *J* = 7.2 Hz, 3H, CHCH_3_), 1.37–1.24 (m, 4H, CH_2_), 0.97 (t, *J* = 7.5 Hz, 3H, CH_3_ DHA), 0.90 (t, *J* = 7.2 Hz, 3H, CH_3_ SFA), 0.89 (d, *J* = 6.6 Hz, 6H, CH(C*H*_3_)_2_) ppm. ^13^C{H} NMR (101 MHz, CDCl_3_) δ_C_: 173.8 (Ibu), 173.3 (SFA), 172.5 (DHA), 140.6, 137.3, 132.1, 129.4 (2), 129.3, 128.6, 128.4, 128.32, 128.29, 128.14, 128.12, 128.05, 127.9, 127.7 (2), 127.2, 127.1, 69.2, 62.1, 62.1, 45.09, 45.07, 34.0, 33.8, 31.3, 30.2, 25.7 (2), 25.63, 25.59 (2), 24.6, 22.6, 22.4 (2), 22.3, 20.6, 18.4, 14.3, 13.9 ppm. HRMS (ESI) *m*/*z*: [M + Na]^+^ calcd for C_44_H_64_O_6_Na 711.4595; found, 711.4584.

#### 3.6.2. Synthesis of 3-[4*Z*,7*Z*,10*Z*,13*Z*,16*Z*,19*Z*)-Docosa-4,7,10,13,16,19-hexaenoyl]-1-hexanoyl-2-[(*S*)-2-(4-isobutylphenyl)propanoyl]-*sn*-glycerol, (*R*,*S′*)-**12a**

To a solution of 1-hexanoyl-2-[(*S*)-2-(4-isobutylphenyl)propanoyl]-*sn*-glycerol (*S*,*S′*)-**8a** (12 mg, 0.032 mmol) and DHA as a free acid (12 mg, 0.035 mmol) in CH_2_Cl_2_ (1.5 mL) were added DMAP (4 mg, 0.029 mmol) and EDCI (7 mg, 0.035 mmol). The solution was stirred on a magnetic stirrer at room temperature for 12 h. The reaction was disconnected by passing the reaction mixture through a short column packed with silica gel by use of Et_2_O/CH_2_Cl_2_ (1:9). The solvent was removed in vacuo on a rotary evaporator. The residue was applied to a silica gel chromatography using petroleum ether/ethyl acetate (9:1) as the eluent, which afforded the product (*R*,*S′*)-**12a** as a yellow oil in 81% yield (18 mg, 0.026 mmol). ${\left[\alpha \right]}_{D}^{20}$ = +6.73 (c. 1.8, CH_2_Cl_2_). IR (NaCl, ν_max_/cm^−1^): 2973 (vs), 2926 (vs), 1742 (vs). ^1^H NMR (400 MHz, CDCl_3_) δ_H_: 7.18 (d, *J* = 7.7 Hz, 2H, Ibu-2,6), 7.07 (d, *J* = 7.7 Hz, 2H, Ibu-3,5), 5.54–5.27 (m, 12H, =CH), 5.27–5.23 (m, 1H, CH *sn*-2), 4.30 (dd, *J* = 11.9, 4.3 Hz, 1H, CH_2_ *sn*-1/3), 4.19–4.13 (m, 2H, CH_2_ *sn*-1/3), 4.06 (dd, J = 11.9, 6.3 Hz, 1H, CH_2_ *sn*-1/3), 3.70 (q, *J* = 7.2 Hz, 1H, CHCH_3_), 2.90–2.78 (m, 10H, =CHC*H*_2_CH=), 2.43 (d, *J* = 7.1 Hz, 2H, CH_2_CH(CH_3_)_2_), 2.40–2.27 (m 4H, CH_2_CH_2_COO DHA), 2.15 (m, 2H, =CHCH_2_CH_3_), 2.08 (t, *J* = 7.4 Hz, 2H, CH_2_COO SFA), 1.83 (nonet, *J* = 6.8 Hz, 1H, CH(CH_3_)_2_), 1.65–1.55 (m, 2H, CH_2_CH_2_COO SFA), 1.49 (d, *J* = 7.2 Hz, 3H, CHCH_3_), 1.37–1.24 (m, 4H, CH_2_), 0.97 (t, *J* = 7.5 Hz, 3H, CH_3_ DHA), 0.90 (d, *J* = 6.6 Hz, 6H, CH(CH_3_)_2_), 0.89 (t, *J* = 7.2 Hz, 3H, CH_3_ SFA) ppm. ^13^C{H} NMR (101 MHz, CDCl_3_) δ_C_: 174.0 (Ibu), 173.3 (SFA), 172.7 (DHA), 140.6, 137.3, 132.2, 129.6 (2), 129.4, 128.7, 128.4, 128.3, 128.23 (2), 128.16, 128.0, 127.9, 127.8, 127.7 (2), 127.3, 69.3, 62.4, 62.1, 45.2, 45.1, 34.0, 33.8, 32.1, 30.3, 29.8 (2), 25.8, 25.7 (2), 24.9, 22.9, 22.8 (2), 22.5, 20.7, 18.5, 14.31, 14.27 ppm. HRMS (ESI) *m*/*z*: [M + Na]^+^ calcd for C_44_H_64_O_6_Na 711.4595; found, 711.4590.

#### 3.6.3. Synthesis of 1-[4*Z*,7*Z*,10*Z*,13*Z*,16*Z*,19*Z*)-Docosa-4,7,10,13,16,19-hexaenoyl]-3-hexanoyl-2-[(*S*)-2-(6-methoxynaphthalen-2-yl)propanoyl]-*sn*-glycerol, (*S*,*S′*)-**13a**

To a solution of 3-hexanoyl-2-[(*S*)-2-(6-methoxynaphthalen-2-yl)propanoyl]-*sn*-glycerol (*R*,*S′*)-**9a** (35 mg, 0.087 mmol) and DHA as a free acid (31 mg, 0.093 mmol) in CH_2_Cl_2_ (4 mL) were added DMAP (11 mg, 0.094 mmol) and EDCI (24 mg, 0.127 mmol). The solution was stirred on a magnetic stirrer at room temperature for 12 h. The reaction was disconnected by passing the reaction mixture through a short column packed with silica gel by use of Et_2_O/CH_2_Cl_2_ (1:9). The solvent was removed in vacuo on a rotary evaporator. The residue was applied to a silica gel chromatography using petroleum ether/ethyl acetate (7:3) as the eluent, which afforded the product (*S*,*S′*)-**13a** as a yellow oil in 84% yield (52 mg, 0.073 mmol). ${\left[\alpha \right]}_{D}^{20}$ = +6.81 (c. 5.2, CH_2_Cl_2_). IR (NaCl, ν_max_/cm^−1^): 3013 (vs), 2961 (vs), 2934 (vs), 2873 (vs), 1743 (vs), 1607 (vs). ^1^H NMR (400 MHz, CDCl_3_) δ_H_: 7.72–7.62 (m, 3H, Nap-1,4,8), 7.38 (dd, *J* = 8.5, 1.9 Hz, 1H, Nap-3), 7.13 (dd, *J* = 8.9, 2.5 Hz, 1H, Nap-7), 7.09 (d, *J* = 2.5 Hz, 1H, Nap-5), 5.43–5.23 (m, 12H, =CH), 5.21–5.14 (m, 1H, CH *sn*-2), 4.30 (dd, *J* = 11.9, 4.2 Hz, 1H, CH_2_ *sn*-1/3), 4.18 (dd, *J* = 11.9, 4.3 Hz, 1H, CH_2_ *sn*-1/3), 4.14 (dd, *J* = 11.9, 6.1 Hz, 1H, CH_2_ *sn*-1/3), 4.07 (dd, J = 11.9, 6.5 Hz, 1H, CH_2_ *sn*-1/3), 3.90 (s, 3H, OCH_3_), 3.86 (q, *J* = 7.5 Hz, 1H, C*H*CH_3_), 2.88–2.74 (m, 10H, =CHCH_2_CH=), 2.27–2.21 (m, 2H, CH_2_COO DHA), 2.20–2.13 (m, 2H, CH_2_COO DHA), 2.12–1.99 (m, 4H, CH_2_COO SFA and =CHCH_2_CH_3_), 1.61–1.52 (m, 5H, C*H*_2_CH_2_COO SFA and CHC*H*_3_), 1.35–1.20 (m, 4H, CH_2_), 0.97 (t, *J* = 7.5 Hz, 3H, CH_3_ DHA), 0.89 (t, *J* = 7.0 Hz, 3H, CH_3_ SFA) ppm. ^13^C{H} NMR (101 MHz, CDCl_3_) δ_C_: 173.9 (Nap), 173.4 (SFA), 172.5 (DHA), 157.8, 135.3, 133.9, 132.2, 129.4 (2), 129.0, 128.7, 128.6, 128.41, 128.38, 128.3, 128.21, 128.16, 128.0, 127.8, 127.23, 127.15, 126.2, 126.1, 119.2, 105.7, 69.5, 62.18, 62.15, 55.4, 45.5, 34.1, 33.7, 31.4, 25.8 (2), 25.7, 25.6 (2), 24.6, 22.5, 22.4, 20.7, 18.4, 14.1, 14.0 ppm. HRMS (ESI) *m*/*z*: [M + Na]^+^ calcd for C_45_H_60_O_7_Na 735.4231; found, 735.4227.

#### 3.6.4. Synthesis of 3-[4*Z*,7*Z*,10*Z*,13*Z*,16*Z*,19*Z*)-Docosa-4,7,10,13,16,19-hexaenoyl]-1-hexanoyl-2-[(*S*)-2-(6-methoxynaphthalen-2-yl)propanoyl]-*sn*-glycerol, (*R*,*S′*)-**13a**

To a solution of 1-hexanoyl-2-[(*S*)-2-(6-methoxynaphthalen-2-yl)propanoyl]-*sn*-glycerol (*S*,*S′*)-**9a** (12 mg, 0.030 mmol) and DHA as a free acid (11 mg, 0.033 mmol) in CH_2_Cl_2_ (1.3 mL) were added DMAP (4 mg, 0.033 mmol) and EDCI (8 mg, 0.044 mmol). The solution was stirred on a magnetic stirrer at room temperature for 12 h. The reaction was disconnected by passing the reaction mixture through a short column packed with silica gel by use of Et_2_O/CH_2_Cl_2_ (1:9). The solvent was removed in vacuo on a rotary evaporator. The residue was applied to a silica gel chromatography using petroleum ether/ethyl acetate (8.5:1.5) as the eluent, which afforded the product (*R*,*S′*)-**13a** as a yellow oil in 74% yield (22 mg, 0.022 mmol). ${\left[\alpha \right]}_{D}^{20}$ = +8.80 (c. 0.6, CH_2_Cl_2_). IR (NaCl, ν_max_/cm^−1^): 3013 (vs), 2960 (vs), 2932 (vs), 1732 (vs), 1634 (s), 1607 (s). ^1^H NMR (400 MHz, CDCl_3_) δ_H_: 7.72–7.62 (m, 3H, Nap-1,4,8), 7.38 (dd, *J* = 8.5, 1.9 Hz, 1H, Nap-3), 7.13 (dd, *J* = 8.9, 2.5 Hz, 1H, Nap-7), 7.10 (d, *J* = 2.5 Hz, 1H, Nap-5), 5.46–5.26 (m, 12H, =CH), 5.31–5.21 (m, 1H, CH *sn*-2), 4.30 (dd, *J* = 11.9, 4.2 Hz, 1H, CH_2_ *sn*-1/3), 4.21–4.10 (m, 2H, CH_2_ *sn*-1/3), 4.06 (dd, J = 11.9, 6.4 Hz, 1H, CH_2_ *sn*-1/3), 3.91 (s, 3H, OCH_3_), 3.70 (q, *J* = 7.2 Hz, 1H, CHCH_3_), 2.89–2.77 (m, 10H, =CHC*H*_2_CH=), 2.46–2.28 (m, 4H, CH_2_COO DHA), 2.14–2.01 (m, 2H, CH_2_COO SFA), 2.01–1.88 (m, 2H, =CHC*H*_2_CH_3_), 1.58 (d, *J* = 7.2 Hz, 3H, CHC*H*_3_), 1.37 (quint, *J* = 7.5 Hz, 2H, C*H*_2_CH_2_COO SFA), 1.30–1.20 (m, 4H, CH_2_), 0.97 (t, *J* = 7.5 Hz, 3H, CH_3_ DHA), 0.84 (t, *J* = 7.0 Hz, 3H, CH_3_ SFA) ppm. ^13^C{H} NMR (101 MHz, CDCl_3_) δ_C_: 173.9 (Nap), 173.3 (SFA), 172.7 (DHA), 157.9, 135.4, 133.9, 132.2, 129.6, 129.4, 129.1, 128.7, 128.5, 128.44, 128.41, 128.3, 128.23, 128.16, 128.0, 127.8, 127.24, 127.17, 126.3, 126.1, 119.2, 105.7, 69.5, 62.4, 62.0, 55.4, 45.5, 34.0, 33.8, 31.3, 25.8 (2), 25.7, 25.7 (2), 24.4, 22.7, 22.4, 20.7, 18.5, 14.4, 14.0 ppm. HRMS (ESI) *m*/*z*: [M + Na]^+^ calcd for C_45_H_60_O_7_Na 735.4231; found, 735.4231.

## 4. Conclusions

The synthesis of a focused library of enantiostructured TAGs comprised of an SFA, a bioactive PUFA and a potent drug has been successfully executed by a six-step chemoenzymatic approach, starting from enantiopure (*R*)- and (*S*)-solketals as chiral precursors. All combinations of even number saturated fatty acids ranging from C6:0 to C16:0, EPA and DHA, and (S)-ibuprofen and (S)-naproxen, were prepared with the SFA and the PUFA accommodating each of the terminal positions and the drug entity located at the mid position of the glycerol backbone of the molecule. A total of 48 such enantiostructured TAG molecular species were prepared. They may be divided into two series of diastereomeric analogs, 24 each, of the (*R*,*S′*) and the (S,*S′*)-stereochemistry by interconversion of the SFA and the PUFA at the end-position.

The immobilized lipase, CAL-B, played a crucial role in the regiocontrol of the synthesis. All products and intermediates were obtained in a high chemical, regio- and stereo-isomeric purity and in very high to excellent yields in almost all cases. All products (48) and intermediates (60) were isolated, purified and fully characterized.

It is anticipated that the resulting structured TAGs may possibly find use as an interesting and novel type of prodrugs. An acylglycerol-based prodrug possessing a potent NSAID along with a bioactive n-3 PUFA that is also considered a prodrug offering pro-inflammatory properties, but also a drug, may offer some interesting properties, perhaps by some synergistic effects. The idea of introducing a saturated fatty acyl group to the molecule may indeed offer some interesting properties in terms of the timing of the release of the different acyl groups, including the drug, and hence some site-specificity. An alternative type of related prodrugs, namely those possessing the potent drug in one of the terminal positions, with the SFA in the remaining one and the PUFA, this time in the mid position, is underway to be reported. That second category of enantiostructured TAG prodrugs may offer some complementary possibilities in modifying the time of release and a subsequent site of release of the drug and the acyl counterparts.

## Figures and Tables

**Table 1:** (**a**) Summary of the yields and specific rotation of the intermediates (*R*)-**5a**–**f**. (**b**) Summary of the yields and specific rotation of the intermediates (*S*)-**5a**–**f**.

(**a**)					
Compound	*sn*-1	*sn*-2	*sn*-3	Yields	${\left[\alpha \right]}_{D}^{20}$
(*R*)-**5a**	OBn	OH	6:0	94%	−2.29
(*R*)-**5b**	OBn	OH	8:0	94%	−2.22
(*R*)-**5c**	OBn	OH	10:0	97%	−1.59
(*R*)-**5d**	OBn	OH	12:0	97%	−1.91
(*R*)-**5e**	OBn	OH	14:0	94%	−1.31
(*R*)-**5f**	OBn	OH	16:0	94%	−2.37
(**b**)					
Compound	*sn*-1	*sn*-2	*sn*-3	Yields	${\left[\alpha \right]}_{D}^{20}$
(*S*)-**5a**	6:0	OH	OBn	95%	+2.01
(*S*)-**5b**	8:0	OH	OBn	95%	+2.10
(*S*)-**5c**	10:0	OH	OBn	97%	+1.48
(*S*)-**5d**	12:0	OH	OBn	96%	+1.31
(*S*)-**5e**	14:0	OH	OBn	94%	+3.64
(*S*)-**5f**	16:0	OH	OBn	87%	+1.11

**Table 2:** (**a**) Summary of the yields and specific rotation of the intermediates (*R*,*S′*)-**6a**-**f**. (**b**) Summary of the yields and specific rotation of the intermediates (*S*,*S′*)-**6a**–**f**.

(**a**)					
Compound	*sn*-1	*sn*-2	*sn*-3	Yields	${\left[\alpha \right]}_{D}^{20}$
(*R*,*S′*)-**6a**	OBn	Ibu	6:0	80%	−0.77
(*R*,*S′*)-**6b**	OBn	Ibu	8:0	91%	−0.99
(*R*,*S′*)-**6c**	OBn	Ibu	10:0	98%	−0.58
(*R*,*S′*)-**6d**	OBn	Ibu	12:0	91%	−0.86
(*R*,*S′*)-**6e**	OBn	Ibu	14:0	94%	−0.93
(*R*,*S′*)-**6f**	OBn	Ibu	16:0	98%	−0.57
(**b**)					
Compound	*sn*-1	*sn*-2	*sn*-3	Yields	${\left[\alpha \right]}_{D}^{20}$
(*S*,*S′*)-**6a**	6:0	Ibu	OBn	93%	+22.2
(*S*,*S′*)-**6b**	8:0	Ibu	OBn	95%	+21.0
(*S*,*S′*)-**6c**	10:0	Ibu	OBn	81%	+22.0
(*S*,*S′*)-**6d**	12:0	Ibu	OBn	84%	+19.3
(*S*,*S′*)-**6e**	14:0	Ibu	OBn	91%	+17.9
(*S*,*S′*)-**6f**	16:0	Ibu	OBn	84%	+11.6

**Table 3:** (**a**) Summary of the yields and specific rotation of the intermediates (*R*,*S′*)-**7a**–**f**. (**b**) Summary of the yields and specific rotation of the intermediates (*S*,*S′*)-**7a**–**f**.

(**a**)					
Compound	*sn*-1	*sn*-2	*sn*-3	Yields	${\left[\alpha \right]}_{D}^{20}$
(*R*,*S′*)-**7a**	OBn	Nap	6:0	90%	−3.90
(*R*,*S′*)-**7b**	OBn	Nap	8:0	95%	−3.02
(*R*,*S′*)-**7c**	OBn	Nap	10:0	96%	−3.23
(*R*,*S′*)-**7d**	OBn	Nap	12:0	92%	−2.00
(*R*,*S′*)-**7e**	OBn	Nap	14:0	98%	−1.33
(*R*,*S′*)-**7f**	OBn	Nap	16:0	96%	−2.00
(**b**)					
Compound	*sn*-1	*sn*-2	*sn*-3	Yields	${\left[\alpha \right]}_{D}^{20}$
(*S*,*S′*)-**7a**	6:0	Nap	OBn	97%	+20.8
(*S*,*S′*)-**7b**	8:0	Nap	OBn	97%	+4.82
(*S*,*S′*)-**7c**	10:0	Nap	OBn	92%	+6.69
(*S*,*S′*)-**7d**	12:0	Nap	OBn	92%	+17.1
(*S*,*S′*)-**7e**	14:0	Nap	OBn	75%	+15.3
(*S*,*S′*)-**7f**	16:0	Nap	OBn	77%	+15.3

**Table 4:** (**a**) Summary of the yields and specific rotation of the intermediates (*R*,*S′*)-**8a**–**f**. (**b**) Summary of the yields and specific rotation of the intermediates (*S*,*S′*)-**8a**-**f**.

(**a**)					
Compound	*sn*-1	*sn*-2	*sn*-3	Yields	${\left[\alpha \right]}_{D}^{20}$
(*R*,*S′*)-**8a**	OH	Ibu	6:0	84%	+23.9
(*R*,*S′*)-**8b**	OH	Ibu	8:0	86%	+18.3
(*R*,*S′*)-**8c**	OH	Ibu	10:0	98%	+7.50
(*R*,*S′*)-**8d**	OH	Ibu	12:0	92%	+2.43
(*R*,*S′*)-**8e**	OH	Ibu	14:0	84%	+8.00
(*R*,*S′*)-**8f**	OH	Ibu	16:0	90%	+0.75
(**b**)					
Compound	*sn*-1	*sn*-2	*sn*-3	Yields	${\left[\alpha \right]}_{D}^{20}$
(*S*,*S′*)-**8a**	6:0	Ibu	OH	95%	+5.65
(*S*,*S′*)-**8b**	8:0	Ibu	OH	86%	+18.8
(*S*,*S′*)-**8c**	10:0	Ibu	OH	89%	+20.2
(*S*,*S′*)-**8d**	12:0	Ibu	OH	93%	+13.8
(*S*,*S′*)-**8e**	14:0	Ibu	OH	94%	+4.20
(*S*,*S′*)-**8f**	16:0	Ibu	OH	90%	+15.0

**Table 5:** (**a**) Summary of the yields and specific rotation of the intermediates (*R*,*S′*)-**9a**–**f**. (**b**) Summary of the yields and specific rotation of the intermediates (*S*,*S′*)-**9a**–**f**.

(**a**)					
Compound	*sn*-1	*sn*-2	*sn*-3	Yields	${\left[\alpha \right]}_{D}^{20}$
(*R*,*S′*)-**9a**	OBn	Nap	6:0	98%	+5.63
(*R*,*S′*)-**9b**	OBn	Nap	8:0	94%	+7.17
(*R*,*S′*)-**9c**	OBn	Nap	10:0	84%	+5.70
(*R*,*S′*)-**9d**	OBn	Nap	12:0	94%	+3.08
(*R*,*S′*)-**9e**	OBn	Nap	14:0	89%	+2.04
(*R*,*S′*)- **9f**	OBn	Nap	16:0	91%	+4.95
(**b**)					
Compound	*sn*-1	*sn*-2	*sn*-3	Yields	${\left[\alpha \right]}_{D}^{20}$
(*S*,*S′*)-**9a**	6:0	Nap	OH	97%	+8.60
(*S*,*S′*)-**9b**	8:0	Nap	OH	97%	+18.0
(*S*,*S′*)-**9c**	10:0	Nap	OH	92%	+8.56
(*S*,*S′*)-**9d**	12:0	Nap	OH	87%	+17.2
(*S*,*S′*)-**9e**	14:0	Nap	OH	90%	+17.9
(*S*,*S′*)-**9f**	16:0	Nap	OH	90%	+17.0

**Table 6:** (**a**) Summary of the yields and specific rotation of the TAG prodrug products (*S*,*S′*)-**10a**–**f**. (**b**) Summary of the yields and specific rotation of the TAG prodrug products (*R*,*S′*)-**10a**–**f**.

(**a**)					
Compound	*sn*-1	*sn*-2	*sn*-3	Yields	${\left[\alpha \right]}_{D}^{20}$
(*S*,*S′*)-**10a**	EPA	Ibu	6:0	90%	+11.7
(*S*,*S′*)-**10b**	EPA	Ibu	8:0	91%	+9.60
(*S*,*S′*)-**10c**	EPA	Ibu	10:0	88%	+8.40
(*S*,*S′*)-**10d**	EPA	Ibu	12:0	87%	+7.98
(*S*,*S′*)-**10e**	EPA	Ibu	14:0	89%	+6.58
(*S*,*S′*)-**10f**	EPA	Ibu	16:0	86%	+6.68
(**b**)					
Compound	*sn*-1	*sn*-2	*sn*-3	Yields	${\left[\alpha \right]}_{D}^{20}$
(*R*,*S′*)-**10a**	6:0	Ibu	EPA	74%	+6.50
(*R*,*S′*)-**10b**	8:0	Ibu	EPA	91%	+9.11
(*R*,*S′*)-**10c**	10:0	Ibu	EPA	88%	+7.44
(*R*,*S′*)-**10d**	12:0	Ibu	EPA	87%	+7.57
(*R*,*S′*)-**10e**	14:0	Ibu	EPA	89%	+7.12
(*R*,*S′*)-**10f**	16:0	Ibu	EPA	86%	+7.43

**Table 7:** (**a**) Summary of the yields and specific rotation of the TAG prodrug products (*S*,*S′*)-**11a**–**f**. (**b**) Summary of the yields and specific rotation of the TAG prodrug products (*R*,*S′*)-**11a**–**f**.

(**a**)					
Compound	*sn*-1	*sn*-2	*sn*-3	Yields	${\left[\alpha \right]}_{D}^{20}$
(*S*,*S′*)-**11a**	EPA	Nap	6:0	91%	+8.12
(*S*,*S′*)-**11b**	EPA	Nap	8:0	94%	+6.00
(*S*,*S′*)-**11c**	EPA	Nap	10:0	84%	+6.58
(*S*,*S′*)-**11d**	EPA	Nap	12:0	96%	+4.89
(*S*,*S′*)-**11e**	EPA	Nap	14:0	90%	+5.29
(*S*,*S′*)-**11f**	EPA	Nap	16:0	91%	+5.44
(**b**)					
Compound	*sn*-1	*sn*-2	*sn*-3	Yields	${\left[\alpha \right]}_{D}^{20}$
(*R*,*S′*)-**11a**	6:0	Nap	EPA	80%	+4.20
(*R*,*S′*)-**11b**	8:0	Nap	EPA	80%	+4.97
(*R*,*S′*)-**11c**	10:0	Nap	EPA	86%	+8.76
(*R*,*S′*)-**11d**	12:0	Nap	EPA	74%	+5.20
(*R*,*S′*)-**11e**	14:0	Nap	EPA	76%	+6.30
(*R*,*S′*)-**11f**	16:0	Nap	EPA	89%	+6.23

**Table 8:** (**a**) Summary of the yields and specific rotation of the TAG prodrug products (*S*,*S′*)-**12a**–**f**. (**b**) Summary of the yields and specific rotation of the TAG prodrug products (*R*,*S′*)-**12a**–**f**.

(**a**)					
Compound	*sn*-1	*sn*-2	*sn*-3	Yields	${\left[\alpha \right]}_{D}^{20}$
(*S*,*S′*)-**12a**	DHA	Ibu	6:0	81%	+9.95
(*S*,*S′*)-**12b**	DHA	Ibu	8:0	86%	+4.44
(*S*,*S′*)-**12c**	DHA	Ibu	10:0	95%	+4.05
(*S*,*S′*)-**12d**	DHA	Ibu	12:0	89%	+5.30
(*S*,*S′*)-**12e**	DHA	Ibu	14:0	82%	+7.54
(*S*,*S′*)-**12f**	DHA	Ibu	16:0	85%	+3.16
(**b**)					
Compound	*sn*-1	*sn*-2	*sn*-3	Yields	${\left[\alpha \right]}_{D}^{20}$
(*R*,*S′*)-**12a**	6:0	Ibu	DHA	81%	+6.73
(*R*,*S′*)-**12b**	8:0	Ibu	DHA	86%	+7.86
(*R*,*S′*)-**12c**	10:0	Ibu	DHA	80%	+4.57
(*R*,*S′*)-**12d**	12:0	Ibu	DHA	78%	+6.57
(*R*,*S′*)-**12e**	14:0	Ibu	DHA	82%	+7.01
(*R*,*S′*)-**12f**	16:0	Ibu	DHA	79%	+3.93

**Table 9:** (**a**) Summary of the yields and specific rotation of the TAG prodrug products (*S*,*S′*)-**13a**–**f**. (**b**) Summary of the yields and specific rotation of the TAG prodrug products (*R*,*S′*)-**13a**–**f**.

(**a**)					
Compound	*sn*-1	*sn*-2	*sn*-3	Yields	${\left[\alpha \right]}_{D}^{20}$
(*S*,*S′*)-**13a**	DHA	Nap	6:0	84%	+6.81
(*S*,*S′*)-**13b**	DHA	Nap	8:0	85%	+2.78
(*S*,*S′*)-**13c**	DHA	Nap	10:0	89%	+6.60
(*S*,*S′*)-**13d**	DHA	Nap	12:0	92%	+3.67
(*S*,*S′*)-**13e**	DHA	Nap	14:0	93%	+7.37
(*S*,*S′*)-**13f**	DHA	Nap	16:0	92%	+3.60
(**b**)					
Compound	*sn*-1	*sn*-2	*sn*-3	Yields	${\left[\alpha \right]}_{D}^{20}$
(*R*,*S′*)-**13a**	6:0	Nap	DHA	74%	+8.80
(*R*,*S′*)-**13b**	8:0	Nap	DHA	72%	+2.01
(*R*,*S′*)-**13c**	10:0	Nap	DHA	80%	+3.93
(*R*,*S′*)-**13d**	12:0	Nap	DHA	92%	+3.50
(*R*,*S′*)-**13e**	14:0	Nap	DHA	88%	+4.58
(*R*,*S′*)-**13f**	16:0	Nap	DHA	89%	+2.14

## Data Availability

The data underlying this study are available in the published article and its online Supplementary Materials.

## Supplementary Materials

The following supporting information can be downloaded at: https://www.mdpi.com/article/10.3390/molecules29235745/s1, Figure S1: Comparison of the glyceryl proton region of the ^1^H NMR spectra for the 1-*O*-benzyl-*sn*-glycerol starting material and the benzyl-protected monoacylglycerol (*R*)-**5a** (p. S1); Figure S2: Comparison of the glyceryl proton region of the ^1^H NMR spectra for (*R*)-**5a** and (*R*,*S′*)-**6a** possessing (*S*)-ibuprofen (p. S2); Figure S3: Comparison of the glyceryl proton region of the ^1^H NMR spectra for (*R*,*S′*)-**6a** and its deprotected product (*R*,*S′*)-**8a** (p. S3); Figure S4: Comparison of the glyceryl proton region of the ^1^H NMR spectra for (*R*,*S′*)-**9c** and its product (*R*,*S′*)-**11c** acylated with EPA (p. S4); Experimental Information: pS5-S37; NMR spectra (^1^H and ^13^C NMR, ^1^H-^1^H COSY and ^13^C-^1^H HSQC shown for all compounds belonging to **5a**–**13a**): pp. S38–S73.
