# Supplementary material for: Synthesis of Enantiostructured Triacylglycerols Possessing a Saturated Fatty Acid, a Polyunsaturated Fatty Acid and an Active Drug Intended as Novel Prodrugs
Source: Molecules. 2024 Dec 5;29(23):5745. doi: 10.3390/molecules29235745 (PMC11643827; doi:10.3390/molecules29235745)

Supplementary Materials

Synthesis of Enantiostructured Triacylglycerols Possessing a Saturated Fatty Acid, a Polyunsaturated Fatty Acid and an Active Drug Intended as Novel Prodrugs

Lena Rós Jónsdóttir[1] and Gudmundur G. Haraldsson[1]*

1) Science Institute, Chemistry Department, University of Iceland, Dunhaga 3, 107 Reykjavik, Iceland

Table of Contents

**Figures *S1 – S4***	*S1-S4*
**Experimental Information**	*S5-S37*
**NMR Spectra**	*S38-S73*
Compound (*R*)-**5a**	*S38*
Compound (*S*)-**5a**	*S40*
Compound (*R,S′*)-**6a**	*S42*
Compound (*S,S′*)-**6a**	*S44*
Compound (*R,S′*)-**7a**	*S46*
Compound (*S,S′*)-**7a**	*S48*
Compound (*R,S′*)-**8a**	*S50*
Compound (*S,S′*)-**8a**	*S52*

Compound (*R*,*S′*)-**9a** *S54*

Compound (*S*,*S′*)-**9a** *S56*

Compound (*S*,*S′*)-**10a** *S58*

Compound (*R*,*S′*)-**10a** *S60*

Compound (*S*,*S′*)-**11a** *S62*

Compound (*R*,*S′*)-**11a** *S64*

Compound (S,S′)-**12a** *S66*

Compound (R,S′)-**12a** *S68*

Compound (*S*,*S′*)-**13a** *S70*

Compound (*R*,*S′*)-**13a** *S72*

Figures *S1* – *S4*

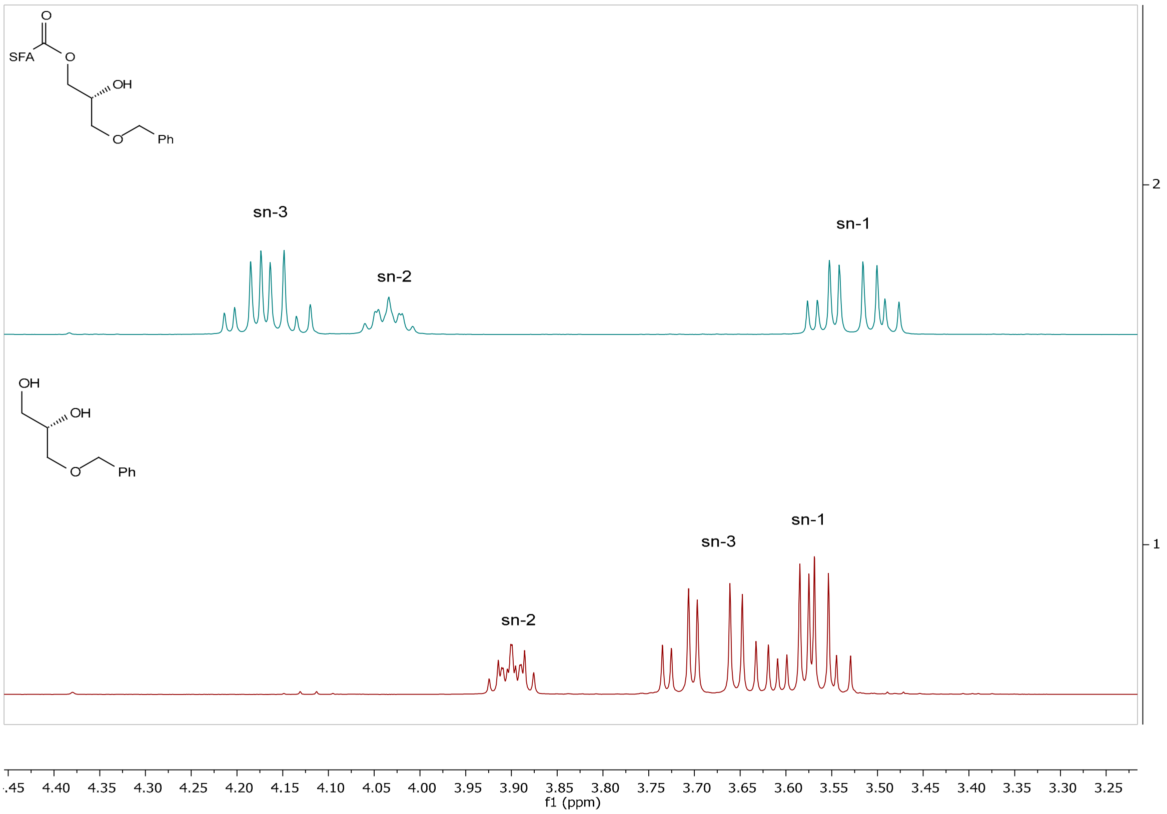

Figure *S1*. Comparison of the glyceryl proton region of the ^{1}H NMR spectra for the 1-*O*-benzyl-*sn*-glycerol starting material (bottom) and the benzyl-protected monoacylglycerol (*R*)-**5a** (top).

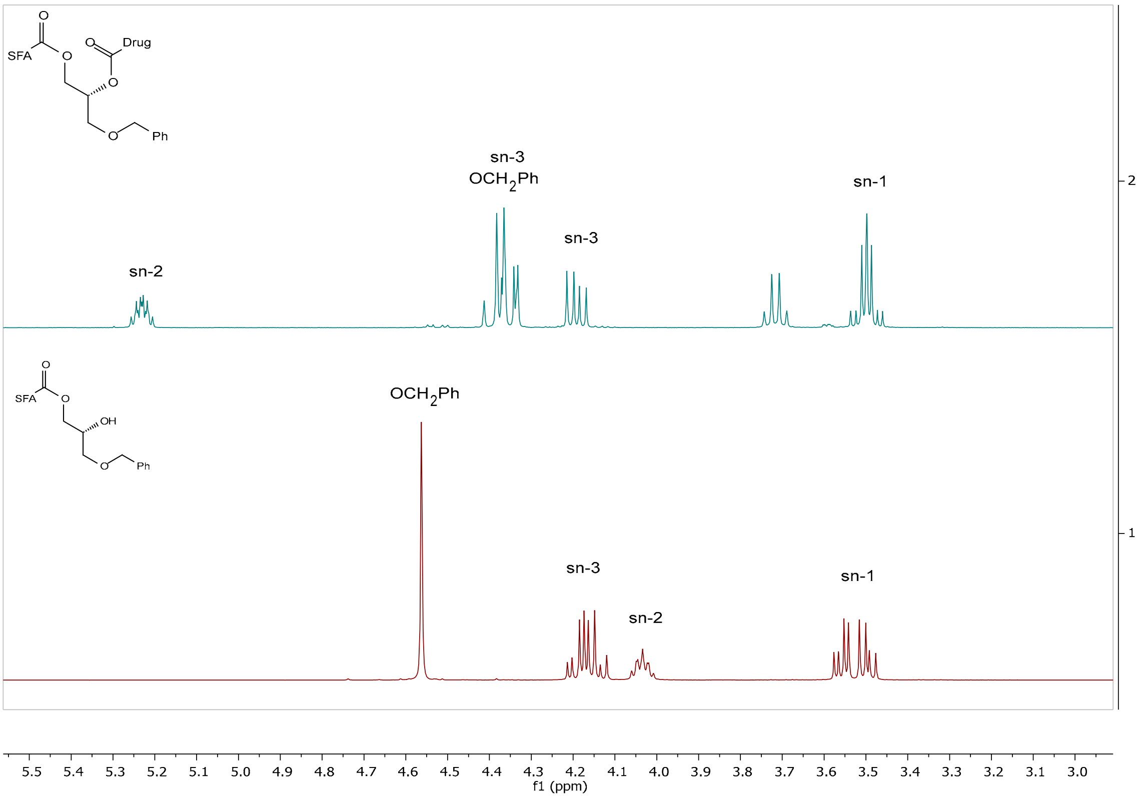

Figure S2. Comparison of the glyceryl proton region of the ^{1}H NMR spectra for (*R*)-**5a** (bottom) and (*R*,*S*′)-**6a** posessing (*S*)-ibuprofen (bottom).

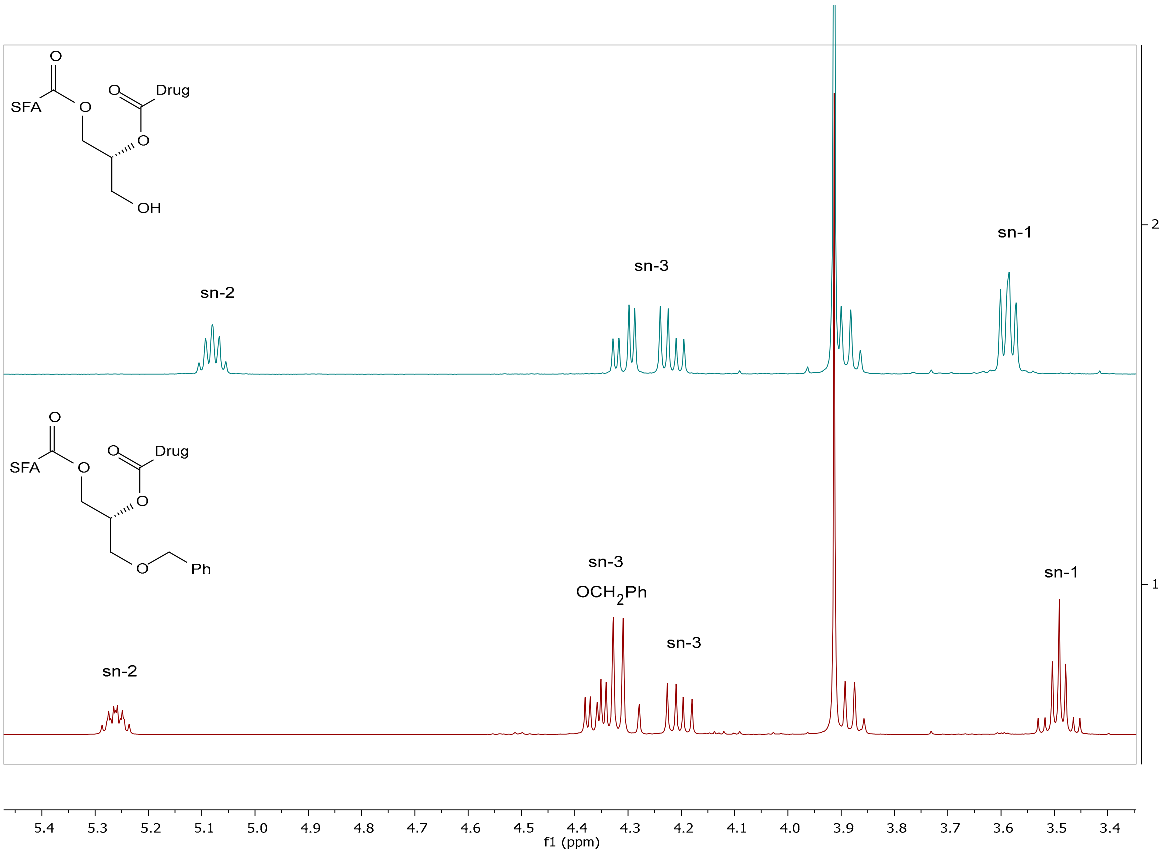

Figure *S3*. Comparison of the glyceryl proton region of the ^{1}H NMR spectra for (*R*,*S′*)-**6a** (bottom) and its deprotected product (*R*,*S′*)-**8a** (bottom).

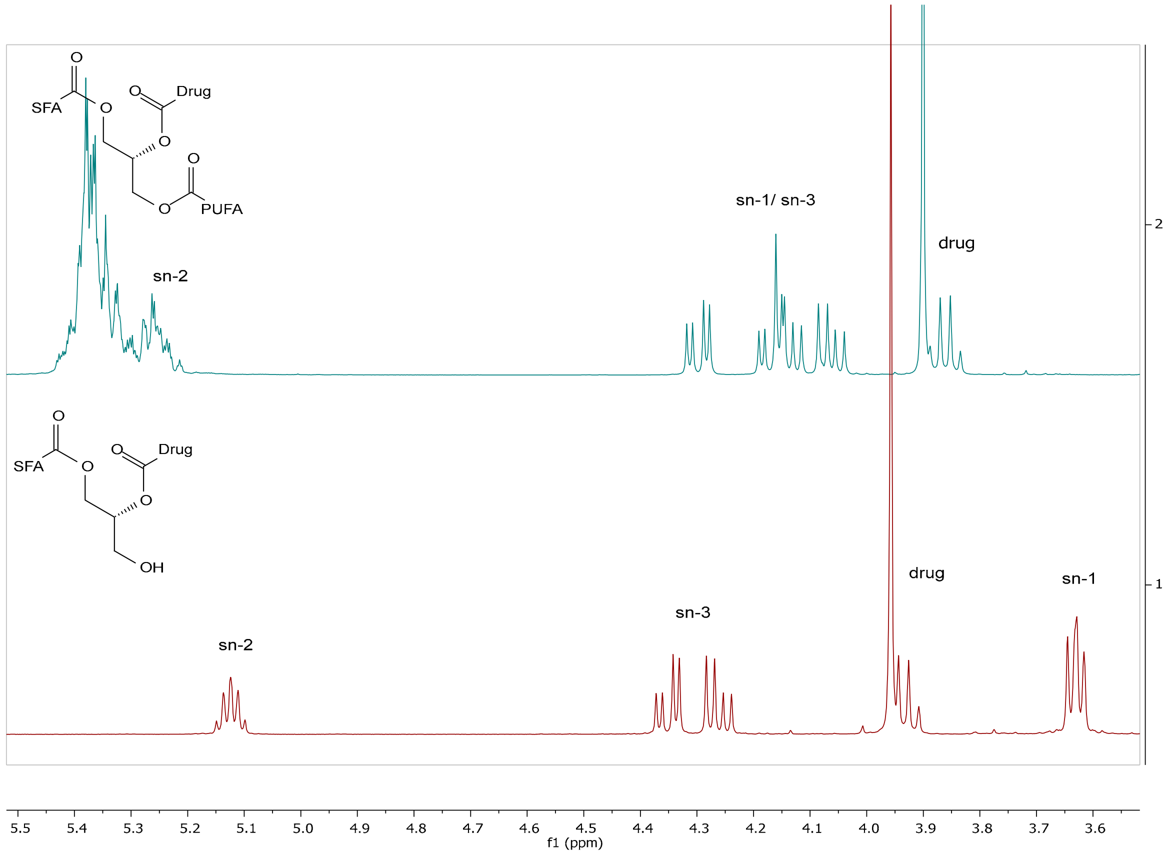

Figure ***S4***. Comparison of the glyceryl proton region of the ^{1}H NMR spectra for (*R*,*S'*)-**9c** (bottom) and its product (*R*,*S'*)-**11c** acylated with EPA (top).

Experimental Information

*3.2. The enzymatic coupling of the SFAs: Synthesis of (R)-**5b**-**f** and (S)-**5b**-**f***

3.2.1. Synthesis of 1-*O*-benzyl-3-octanoyl-*sn*-glycerol, (*R*)-**5b**

The same procedure was followed as described for (*R*)-**5a** using immobilized CAL-B (18 mg), 1-*O*-benzyl-*sn*-glycerol (150 mg, 0.823 mmol), vinyl octanoate (160 mg, 0.940 mmol) and CH_2Cl_2 (3 mL). Purification on a 4% boric acid impregnated flash silica gel chromatography using pet. ether/ethyl acetate (7:3) as eluent afforded the product (*R*)-**5b** as a colorless liquid in 94% yield (239 mg, 0.775 mmol). $[\alpha]^{20}_D$ = -2.22 (c. 10.1, CH_2Cl_2). IR (NaCl, ν_{max} / cm^{-1}): 3459 (br), 3031 (s), 2955 (vs), 2928 (vs), 2857 (vs), 1739 (vs). ^1H NMR (400 MHz, $CDCl_3$) δ_H: 7.38-7.27 (m, 5H, Ph-H), 4.56 (s, 2H, $PhCH_2$), 4.19 (dd, *J*=11.5, 4.5 Hz, 1H, CH_2 *sn*-3), 4.14 (dd, *J*=11.5, 6.0 Hz, 1H, CH_2 *sn*-3), 4.06-4.01 (m, 1H, CH *sn*-2), 3.56 (dd, *J*=9.6, 4.4 Hz, 1H, CH_2 *sn*-1), 3.49 (dd, *J*=9.6, 6.1 Hz, 1H, CH_2 *sn*-1), 2.56 (bs, 1H, OH), 2.32 (t, *J*=7.6 Hz, 2H, CH_2COO), 1.65-1.58 (m, 2H, $C\mathit{H}_2CH_2COO$), 1.32-1.22 (m, 8H, CH_2), 0.88 (t, *J*=6.9 Hz, 3H, CH_3) ppm. $^{13}C\{H\}$ NMR (101 MHz, $CDCl_3$) δ_C: 174.1, 137.8, 128.6 (2), 128.0, 127.9 (2), 73.6, 71.0, 69.1, 65.48, 34.3, 31.6, 29.2, 29.0, 25.0, 22.7, 14.2 ppm. HRMS (ESI) *m*/*z*: $[M + Na]^+$ calcd for $C_{18}H_{28}O_4Na$ 331.1880; found, 331.1871.

3.2.2. Synthesis of 1-*O*-benzyl-3-decanoyl-*sn*-glycerol, (*R*)-**5c**

The same procedure was followed as described for (*R*)-**5a** using immobilized CAL-B (18 mg), 1-*O*-benzyl-*sn*-glycerol (150 mg, 0.823 mmol), vinyl decanoate (186 mg, 0.940 mmol) and CH_2Cl_2 (3 mL). Purification on a 4% boric acid impregnated flash silica gel chromatography using pet. ether/ethyl acetate (7:3) as eluent afforded the product (*R*)-**5c** as a colorless liquid in 97% yield (268 mg, 0.797 mmol). $[\alpha]^{20}_D$ = -1.59 (c. 10.0, CH_2Cl_2). IR (NaCl, ν_{max} / cm^{-1}): 3459 (br), 3031 (s), 2926 (vs), 2857 (vs), 1739 (vs), 1173 (br s). ^1H NMR (400 MHz, $CDCl_3$) δ_H: 7.38-7.27 (m, 5H, Ph-H), 4.56 (s, 2H, $PhCH_2$), 4.19 (dd, *J*=11.5, 4.5 Hz, 1H, CH_2 *sn*-3), 4.14 (dd, *J*=11.5, 6.0 Hz, 1H, CH_2 *sn*-3), 4.06-4.01 (m, 1H, CH *sn*-2), 3.56 (dd, *J*=9.6, 4.4 Hz, 1H, CH_2 *sn*-1), 3.55 (dd, *J*=9.6, 6.1 Hz, 1H, CH_2 *sn*-1), 2.57 (bs, 1H, OH), 2.32 (t, *J*=7.6 Hz, 2H, CH_2COO), 1.66-1.57 (m, 2H, $C\mathit{H}_2CH_2COO$), 1.35-1.26 (m, 12H, CH_2), 0.87 (t, *J*=6.9 Hz, 3H, CH_3) ppm. $^{13}C\{H\}$ NMR (101 MHz, $CDCl_3$) δ_C: 174.1, 137.8, 128.6 (2), 128.0, 127.9 (2), 73.6, 71.0, 69.1, 65.5, 34.3, 32.0, 29.5 (2), 29.4, 29.3, 24.0, 22.8, 14.2 ppm. HRMS (ESI) *m*/*z*: $[M + Na]^+$ calcd for $C_{20}H_{32}O_4Na$ 359.2193; found, 359.2193.

3.2.3. Synthesis of 1-*O*-benzyl-3-dodecanoyl-*sn*-glycerol, (*R*)-**5d**

The same procedure was followed as described for (*R*)-**5a** using immobilized CAL-B (18 mg), 1-*O*-benzyl-*sn*-glycerol (150 mg, 0.823 mmol), vinyl dodecanoate (213 mg, 0.940 mmol) and CH_2Cl_2 (3 mL). Purification on a 4% boric acid impregnated flash silica gel chromatography using pet. ether/ethyl acetate (7:3) as eluent afforded the product (*R*)-**5d** as a colorless liquid in 97% yield (277 mg, 0.799 mmol). $[\alpha]^{20}_D$ = -1.91 (c. 10.0, CH_2Cl_2). IR (NaCl, ν_{max} / cm^{-1}): 3459 (br), 3031 (s), 2925 (vs), 2854 (vs), 1739 (v), 1174 (br s). ^1H NMR (400 MHz, $CDCl_3$) δ_H: 7.38-7.27 (m, 5H, Ph-H), 4.56 (s, 2H, $PhCH_2$), 4.19 (dd, *J*=11.5, 4.5 Hz, 1H, CH_2 *sn*-3), 4.14 (dd, *J*=11.5, 6.0 Hz, 1H, CH_2 *sn*-3), 4.06-4.00 (m, 1H, CH *sn*-2), 3.56 (dd, *J*=9.6, 4.3 Hz, 1H, CH_2 *sn*-1), 3.50 (dd, *J*=9.6, 6.1 Hz, 1H, CH_2 *sn*-1), 2.48 (bs, 1H, OH), 2.32 (t, *J*=7.5 Hz, 2H, CH_2COO), 1.65-1.57 (m, 2H, $C\mathit{H}_2CH_2COO$), 1.32-1.26 (m, 16H, CH_2), 0.88 (t, *J*=6.9 Hz, 3H, CH_3) ppm. $^{13}C\{H\}$ NMR (101 MHz, $CDCl_3$) δ_C: 174.1, 137.8, 128.6 (2), 128.0, 127.9, 73.6, 71.0, 69.1, 65.5, 34.3, 32.0, 29.7 (2), 29.6, 29.5, 29.4, 29.3, 25.1, 22.8, 14.2 ppm. HRMS (ESI) *m*/*z*: $[M + Na]^+$ calcd for $C_{22}H_{36}O_4Na$ 387.2506; found, 387.2498.

3.2.4. Synthesis of 1-*O*-benzyl-3-tetradecanoyl-*sn*-glycerol, (*R*)-**5e**

The same procedure was followed as described for (*R*)-**5a** using immobilized CAL-B (18 mg), 1-*O*-benzyl-*sn*-glycerol (150 mg, 0.823 mmol), vinyl tetradecanoate (239 mg, 0.940 mmol) and CH_2Cl_2 (3 mL). Purification on a 4% boric acid impregnated flash silica gel chromatography using pet. ether/ethyl acetate

(7:3) as eluent afforded the product (*R*)-**5e** as a colorless liquid in 94% yield (304 mg, 0.774 mmol). $[\alpha]^{20}_{D}$ = -1.31 (c. 10.2, CH_2Cl_2). IR (NaCl, ν_{max} / cm^{-1}): 3458 (br), 3064 (s), 3031 (s), 2925 (vs), 2854 (vs), 1739 (vs), 1174 (br s). ^1H NMR (400 MHz, $CDCl_3$) δ_H: 7.38-7.27 (m, 5H, Ph-H), 4.56 (s, 2H, $PhCH_2$), 4.19 (dd, *J*=11.5, 4.5 Hz, 1H, CH_2 *sn*-3), 4.14 (dd, *J*=11.5, 6.0 Hz, 1H, CH_2 *sn*-3), 4.06-4.00 (m, 1H, CH *sn*-2), 3.56 (dd, *J*=9.6, 4.3 Hz, 1H, CH_2 *sn*-1), 3.50 (dd, *J*=9.6, 6.1 Hz, 1H, CH_2 *sn*-1), 2.48 (bs, 1H, OH), 2.32 (t, *J*=7.6 Hz, 2H, CH_2COO), 1.66-1.58 (m, 2H, CH_2CH_2COO), 1.38-1.24 (m, 20H, CH_2), 0.88 (t, *J*=6.9 Hz, 3H, CH_3) ppm. $^{13}C\{H\}$ NMR (101 MHz, $CDCl_3$) δ_C: 174.1, 137.8, 128.6 (2), 128.0, 127.9 (2), 73.7, 71.0, 69.1, 65.5, 34.3, 32.1, 29.8, 29.8, 29. 8, 29.7, 29.6, 29.5, 29.4, 29.3, 25.1, 22.8, 14.2 ppm. HRMS (ESI) *m*/*z*: $[M + Na]^+$ calcd for $C_{24}H_{40}O_4Na$ 415.2819; found, 415.2806.

3.2.5. Synthesis of 1-*O*-benzyl-3-hexadecanoyl-*sn*-glycerol, (*R*)-**5f**

The same procedure was followed as described for (*R*)-**5a** using immobilized CAL-B (18 mg), 1-*O*-benzyl-*sn*-glycerol (150 mg, 0.823 mmol), vinyl hexadecanoate (266 mg, 0.940 mmol) and CH_2Cl_2 (3 mL). Purification on a 4% boric acid impregnated flash silica gel chromatography using pet. ether/ethyl acetate (7:3) as eluent afforded the product (*R*)-**5f** as a colorless liquid in 94% yield (326 mg, 0.775 mmol). $[\alpha]^{20}_{D}$ = -2.37 (c. 10.5, CH_2Cl_2). IR (NaCl, ν_{max} / cm^{-1}): 3459 (br), 3064 (s), 3031 (s), 2924 (vs), 2853 (vs), 1739 (vs), 1174 (br s). ^1H NMR (400 MHz, $CDCl_3$) δ_H: 7.39-7.27 (m, 5H, Ph-H), 4.56 (s, 2H, $PhCH_2$), 4.19 (dd, *J*=11.5, 4.5 Hz, 1H, CH_2 *sn*-3), 4.14 (dd, *J*=11.5, 6.0 Hz, 1H, CH_2 *sn*-3), 4.07-4.00 (m, 1H, CH *sn*-2), 3.56 (dd, *J*=9.6, 4.4 Hz, 1H, CH_2 *sn*-1), 3.50 (dd, *J*=9.6, 6.1 Hz, 1H, CH_2 *sn*-1), 2.51 (bs, 1H, OH), 2.32 (t, *J*=7.6 Hz, 2H, CH_2COO), 1.67-1.56 (m, 2H, CH_2CH_2COO), 1.38-1.24 (m, 24H, CH_2), 0.88 (t, *J*=6.8 Hz, 3H, CH_3) ppm. $^{13}C\{H\}$ NMR (101 MHz, $CDCl_3$) δ_C: 174.1, 137.8, 128.6 (2), 128.0, 127.9 (2), 73.7, 71.0, 69.1, 65.5, 34.3, 32.1, 29.8 (2), 29.8 (2), 29.8 (2), 29.6, 29.5, 29.4, 29.3, 25.1, 22.8, 14.3 ppm. HRMS (ESI) *m*/*z*: $[M + Na]^+$ calcd for $C_{26}H_{44}O_4Na$ 443.3132; found, 443.3127.

3.2.6. Synthesis of 3-*O*-benzyl-1-octanoyl-*sn*-glycerol, (*S*)-**5b**

The same procedure was followed as described for (*R*)-**5a** using immobilized CAL-B (35 mg), 3-*O*-benzyl-*sn*-glycerol (284 mg, 1.54 mmol), vinyl octanoate (300 mg, 1.76 mmol) and CH_2Cl_2 (3 mL). Purification on a 4% boric acid impregnated flash silica gel chromatography using pet. ether/ethyl acetate (7:3) as eluent afforded the product (*S*)-**5b** as a colorless liquid in 95% yield (450 mg, 1.46 mmol). Spectroscopic data identical to those for (*R*)-**5b** were obtained. $[\alpha]^{20}_{D}$ = +2.10 (c. 1.6, CH_2Cl_2). HRMS (ESI) *m*/*z*: $[M + Na]^+$ calcd for $C_{18}H_{28}O_4Na$ 331.1880; found, 331.1882.

3.2.7. Synthesis of 3-*O*-benzyl-1-decanoyl-*sn*-glycerol, (*S*)-**5c**

The same procedure was followed as described for (*R*)-**5a** using immobilized CAL-B (24 mg), 3-*O*-benzyl-*sn*-glycerol (250 mg, 1.37 mmol), vinyl decanoate (350 mg, 1.76 mmol) and CH_2Cl_2 (3 mL). Purification on a 4% boric acid impregnated flash silica gel chromatography using pet. ether/ethyl acetate (7:3) as eluent afforded the product (*S*)-**5c** as a colorless liquid in 97% yield (447 mg, 1.33 mmol). Spectroscopic data identical to those for (*R*)-**5c** were obtained. $[\alpha]^{20}_{D}$ = +1.48 (c. 10.0, CH_2Cl_2). HRMS (ESI) *m*/*z*: $[M + Na]^+$ calcd for $C_{20}H_{32}O_4Na$ 359.2193; found, 359.2193.

3.2.8. Synthesis of 3-*O*-benzyl-1-dodecanoyl-*sn*-glycerol, (*S*)-**5d**

The same procedure was followed as described for (*R*)-**5a** using immobilized CAL-B (20 mg), 3-*O*-benzyl-*sn*-glycerol (103 mg, 0.57 mmol), vinyl dodecanoate (163 mg, 0.72 mmol) and CH_2Cl_2 (3 mL). Purification on a 4% boric acid impregnated flash silica gel chromatography using pet. ether/ethyl acetate (7:3) as eluent afforded the product (*S*)-**5d** as a colorless liquid in 96% yield (199 mg, 0.55 mmol). Spectroscopic data identical to those for (*R*)-**5d** were obtained. $[\alpha]^{20}_{D}$ = +1.31 (c. 4.7, CH_2Cl_2). HRMS (ESI) *m*/*z*: $[M + Na]^+$ calcd for $C_{22}H_{36}O_4Na$ 387.2506; found, 387.2499.

3.2.9. Synthesis of 3-*O*-benzyl-1-tetradecanoyl-*sn*-glycerol, (*S*)-**5e**

The same procedure was followed as described for (*R*)-**5a** using immobilized CAL-B (6 mg), 3-*O*-benzyl-*sn*-glycerol (50 mg, 0.27 mmol), vinyl tetradecanoate (79 mg, 0.31 mmol) and CH_2Cl_2 (3 mL). Purification on a 4% boric acid impregnated flash silica gel chromatography using pet. ether/ethyl acetate (4:1) as eluent afforded the product (*S*)-**5e** as a colorless liquid in 94% yield (100 mg, 0.25 mmol). Spectroscopic data identical to those for (*R*)-**5e** were obtained. $[\alpha]^{20}_D$ = +3.64 (c. 5.0, CH_2Cl_2). HRMS (ESI) *m/z*: $[M + Na]^+$ calcd for $C_{24}H_{40}O_4Na$ 415.2819; found, 415.2825.

3.2.10. Synthesis of 3-*O*-benzyl-1-hexadecanoyl-*sn*-glycerol, (*S*)-**5f**

The same procedure was followed as described for (*R*)-**5a** using immobilized CAL-B (18 mg), 3-*O*-benzyl-*sn*-glycerol (100 mg, 0.55 mmol), vinyl hexaadecanoate (117 mg, 0.63 mmol) and CH_2Cl_2 (3 mL). Purification on a 4% boric acid impregnated flash silica gel chromatography using pet. ether/ethyl acetate (7:3) as eluent afforded the product (*S*)-**5f** as a colorless liquid in 87% yield (90 mg, 0.48 mmol). Spectroscopic data identical to those for (*R*)-**5f** were obtained. $[\alpha]^{20}_D$ = +1.11 (c. 9.0, CH_2Cl_2). HRMS (ESI) *m/z*: $[M + Na]^+$ calcd for $C_{26}H_{44}O_4Na$ 443.3132; found, 443.3133.

*3.3. The coupling of the active drugs: Synthesis of (R,S')-**6b-f**, (S,S')-**6b-f**, (R,S')-**7b-f** and (S,S')-**7b-f***

3.3.1. Synthesis of 1-*O*-benzyl-2-[(*S*)-2-(4-isobutylphenyl)propanoyl]-3-octanoyl-*sn*-glycerol, (*R,S*')-**6b**

The same procedure was followed as described for (*R,S*')-**6a** using 1-*O*-benzyl-3-octanoyl-*sn*-glycerol (*R*)-**5b** (100 mg, 0.324 mmol), (*S*)-ibuprofen (81 mg, 0.391 mmol), CH_2Cl_2 (3 mL), DMAP (35 mg, 0.285 mmol) and EDCI (66 mg, 0.344 mmol). Purification on a silica gel chromatography using pet. ether/ethyl acetate (7:3) as eluent afforded the product (*R,S*')-**6b** as a pale-yellow oil, in 91% yield (146 mg, 0.294 mmol). $[\alpha]^{20}_D$ = -0.99 (c. 10.0, CH_2Cl_2). IR (NaCl, ν_{max} / cm^{-1}): 3028 (s), 2955 (vs), 2927 (vs), 2868 (vs), 1740 (vs), 1161 (br s). ^1H NMR (400 MHz, $CDCl_3$) δ_H: 7.34-7.25 (m, 3H, Ph-H), 7.21 (m, 2H, Ibu-2,6 and 2H, Ph-H), 7.07 (d, *J*=8.1 Hz, 2H, Ibu-3,5), 5.27-5.21 (m, 1H, CH *sn*-2), 4.42-4.34 (m, 1H, CH_2 *sn*-3 and 2H, $PhCH_2$), 4.20 (dd, *J*=11.9, 6.6 Hz, 1H, CH_2 *sn*-3), 3.72 (q, *J*=7.2 Hz, 1H, C*H*CH_3), 3.55-3.46 (m, 2H, CH_2 *sn*-1), 2.43 (d, *J*=7.2 Hz, 2H, C*H*$_2CH(CH_3)_2$), 2.26 (t, *J*=7.5 Hz, 2H, CH_2COO), 1.83 (nonet, *J*=6.7 Hz, 1H, C*H*$(CH_3)_2$), 1.64-1.54 (m, 2H, C*H*$_2CH_2COO$), 1.50 (d, *J*=7.2 Hz, 3H, CHC*H*$_3$), 1.36-1.25 (m, 8H, CH_2), 0.90 (t, *J*=6.8 Hz, 3H, CH_2C*H*$_3$), 0.89 (d, *J*=6.6 Hz, 6H, CH(C*H*$_3)_2$) ppm. $^{13}C\{H\}$ NMR (101 MHz, $CDCl_3$) δ_C: 174.1 (Ibu), 173.5 (SFA), 140.7, 137.9, 137.6, 129.4 (2), 128.5 (2), 127.8 (2), 127.6 (2), 127.3, 73.4, 70.8, 68.4, 62.8, 45.3, 45.2, 34.2, 31.8, 30.3, 29.2, 29.1, 25.0, 22.8, 22.5 (2), 18.7, 14.2 ppm. HRMS (ESI) *m/z*: $[M + Na]^+$ calcd for $C_{31}H_{44}O_5Na$ 519.3081; found, 519.3072.

3.3.2. Synthesis of 1-*O*-benzyl-3-decanoyl-2-[(*S*)-2-(4-isobutylphenyl)propanoyl]-*sn*-glycerol, (*R,S*')-**6c**

The same procedure was followed as described for (*R,S*')-**6a** using 1-*O*-benzyl-3-decanoyl-*sn*-glycerol (*R*)-**5c** (100 mg, 0.324 mmol), (*S*)-ibuprofen (74 mg, 0.358 mmol), CH_2Cl_2 (3 mL), DMAP (32 mg, 0.261 mmol) and EDCI (60 mg, 0.315 mmol). Purification on a silica gel chromatography using pet. ether/ethyl acetate (7:3) as eluent afforded the product (*R,S*')-**6c** as a pale-yellow oil, in 98% yield (154 mg, 0.293 mmol). $[\alpha]^{20}_D$ = -0.58 (c. 3.8, CH_2Cl_2). IR (NaCl, ν_{max} / cm^{-1}): 3029 (s), 2955 (vs), 2927 (vs), 2856 (vs), 1740 (vs), 1159 (br s). ^1H NMR (400 MHz, $CDCl_3$) δ_H: 7.35-7.25 (m, 3H, Ph-H), 7.23-7.17 (m, 2H, Ibu-2,6 and 2H, Ph-H), 7.06 (d, *J*=8.1 Hz, 2H, Ibu-3,5), 5.27-5.19 (m, 1H, CH *sn*-2), 4.42-4.32 (m, 1H, CH_2 *sn*-3 and 2H, $PhCH_2$), 4.19 (dd, *J*=11.9, 6.6 Hz, 1H, CH_2 *sn*-3), 3.72 (q, *J*=7.2 Hz, 1H, C*H*CH_3), 3.54-3.45 (m, 2H, CH_2 *sn*-1), 2.42 (d, *J*=7.2 Hz, 2H, C*H*$_2CH(CH_3)_2$), 2.26 (t, *J*=7.6 Hz, 2H, CH_2COO), 1.82 (nonet, *J*=6.8 Hz, 1H, C*H*$(CH_3)_2$), 1.63-1.52 (m, 2H, C*H*$_2CH_2COO$), 1.50 (d, *J*=7.2 Hz, 3H, CHC*H*$_3$), 1.35-1.21 (m, 12H, CH_2), 0.89 (t, *J*=7.2 Hz, 3H, CH_2C*H*$_3$), 0.89 (d, *J*=6.6 Hz, 6H, CH(C*H*$_3)_2$) ppm. $^{13}C\{H\}$ NMR (101 MHz, $CDCl_3$) δ_C: 174.1 (Ibu), 173.5 (SFA),

140.7, 137.9, 137.6, 129.4 (2), 128.5 (2), 127.8 (2), 127.6 (2), 127.3, 73.5, 70.8, 68.4, 62.8, 45.3, 45.2, 34.25, 32.0, 30.3, 29.6, 29.4, 29.3, 25.01, 22.8 (2), 22.5, 18.7, 14.3 ppm. HRMS (ESI) *m/z*: $[M + Na]^+$ calcd for $C_{33}H_{48}O_5Na$ 547.3394; found, 547.3388.

3.3.3. Synthesis of 1-*O*-benzyl-3-dodecanoyl-2-[(*S*)-2-(4-isobutylphenyl)propanoyl]-*sn*-glycerol, (*R*,*S*')-**6d**

The same procedure was followed as described for (*R*,*S*')-**6a** using 1-*O*-benzyl-3-dodecanoyl-*sn*-glycerol (*R*)-**5d** (100 mg, 0.289 mmol), (*S*)-ibuprofen (72 mg, 0.348 mmol), CH_2Cl_2 (3 mL), DMAP (31 mg, 0.254 mmol) and EDCI (59 mg, 0.301 mmol). Purification on a silica gel chromatography using pet. ether/ethyl acetate (4:1) as eluent afforded the product (*R*,*S*')-**6d** as a pale-yellow oil, in 91% yield (141 mg, 0.262 mmol). $[\alpha]^{20}_D$ = -0.86 (c. 2.9, CH_2Cl_2). IR (NaCl, ν_{max} / cm^{-1}): 3029 (s), 2954 (vs), 2926 (vs), 2854 (vs), 1741 (vs,) 1161 (br s). ^1H NMR (400 MHz, $CDCl_3$) δ_H: 7.34-7.25 (m, 3H, Ph-H), 7.23-7.17 (m, 2H, Ibu-2,6 and 2H, Ph-H), 7.07 (d, *J*=8.2 Hz, 2H, Ibu-3,5), 5.27-5.21 (m, 1H, CH *sn*-2), 4.42-4.33 (m, 1H, CH_2 *sn*-3 and 2H, $PhCH_2$), 4.20 (dd, *J*=11.9, 6.6 Hz, 1H, CH_2 *sn*-3), 3.72 (q, *J*=7.2 Hz, 1H, C*H*CH_3), 3.55-3.46 (m, 2H, CH_2 *sn*-1), 2.43 (d, *J*=7.2 Hz, 2H, C*H*$_2$CH(CH_3)$_2$), 2.26 (t, *J*=7.2 Hz, 2H, CH_2COO), 1.83 (nonet, *J*=6.7 Hz, 1H, C*H*(CH_3)$_2$), 1.63-1.52 (m, 2H, C*H*$_2$$CH_2$COO), 1.50 (d, *J*=7.2 Hz, 3H, CHC*H*$_3$), 1.34-1.23 (m, 16H, CH_2), 0.90 (t, *J*=7.8 Hz, 3H, CH_2C*H*$_3$), 0.89 (d, *J*=6.6 Hz, 6H, CH(C*H*$_3$)$_2$) ppm. $^{13}C\{H\}$ NMR (101 MHz, $CDCl_3$) δ_C: 174.1 (Ibu), 173.5 (SFA), 140.7, 137.9, 137.6, 129.4 (2), 128.5 (2), 127.8 (2), 127.6 (2), 127.3, 73.5, 70.8, 68.4, 62.8, 45.3, 45.2, 34.3, 32.1, 30.3, 29.8 (2), 29.6, 29.5, 29.4, 29.3, 25.0, 22.8 (2), 22.5, 18.7, 14.3 ppm. HRMS (ESI) *m/z*: $[M + Na]^+$ calcd for $C_{35}H_{52}O_5Na$ 575.3707; found, 575.3711.

3.3.4. Synthesis of 1-*O*-benzyl-2-[(*S*)-2-(4-isobutylphenyl)propanoyl]-3-tetradecanoyl-*sn*-glycerol, (*R*,*S*')-**6e**

The same procedure was followed as described for (*R*,*S*')-**6a** using 1-*O*-benzyl-3-teradecanoyl-*sn*-glycerol (*R*)-**5e** (100 mg, 0.255 mmol), (*S*)-ibuprofen (63 mg, 0.307 mmol), CH_2Cl_2 (3 mL), DMAP (27 mg, 0.224 mmol) and EDCI (52 mg, 0.271 mmol). Purification on a silica gel chromatography using pet. ether/ethyl acetate (7:3) as eluent afforded the product (*R*,*S*')-**6e** as a pale-yellow oil, in 95% yield (141 mg, 0.243 mmol). $[\alpha]^{20}_D$ = -0.93 (c. 3.0, CH_2Cl_2). IR (NaCl, ν_{max} / cm^{-1}): 3029 (s), 2923 (vs), 2854 (vs), 1739 (vs), 1647 (vs), 1159 (s). ^1H NMR (400 MHz, $CDCl_3$) δ_H: 7.34-7.25 (m, 3H, Ph-H), 7.21 (m, 2H, Ibu-2,6 and 2H, Ph-H), 7.07 (d, *J*=8.1 Hz, 2H, Ibu-3,5), 5.27-5.21 (m, 1H, CH *sn*-2), 4.42-4.34 (m, 1H, CH_2 *sn*-3 and 2H, $PhCH_2$), 4.20 (dd, *J*=11.9, 6.6 Hz, 1H, CH_2 *sn*-3), 3.72 (q, *J*=7.2 Hz, 1H, C*H*CH_3), 3.55-3.46 (m, 2H, CH_2 *sn*-1), 2.43 (d, *J*=7.2 Hz, 2H, C*H*$_2$CH(CH_3)$_2$), 2.26 (t, *J*=7.5 Hz, 2H, CH_2COO), 1.83 (nonet, *J*=6.7 Hz, 1H, C*H*(CH_3)$_2$), 1.63-1.52 (m, 2H, C*H*$_2$$CH_2$COO), 1.50 (d, *J*=7.2 Hz, 3H, CHC*H*$_3$), 1.36-1.25 (m, 20H, CH_2), 0.90 (t, *J*=6.8 Hz, 3H, CH_2C*H*$_3$), 0.89 (d, *J*=6.6 Hz, 6H, CH(C*H*$_3$)$_2$) ppm. $^{13}C\{H\}$ NMR (101 MHz, $CDCl_3$) δ_C: 174.1 (Ibu), 173.5 (SFA), 140.7, 137.9, 137.6, 129.4 (2), 128.5 (2), 127.8 (2), 127.6 (2), 127.3, 73.5, 70.8, 68.4, 62.8, 45.3, 45.2, 34.3, 32.1, 30.3, 29.83 (2), 29.81, 29.8, 29.6, 29.5, 29.4, 29.3, 25.0, 22.8 (2), 22.5, 18.7, 14.3 ppm. HRMS (ESI) *m/z*: $[M + Na]^+$ calcd for $C_{37}H_{56}O_5Na$ 603.4020; found, 603.4006.

3.3.5. Synthesis of 1-*O*-benzyl-3-hexadecanoyl-2-[(*S*)-2-(4-isobutylphenyl)propanoyl]-*sn*-glycerol, (*R*,*S*')-**6f**

The same procedure was followed as described for (*R*,*S*')-**6a** using 1-*O*-benzyl-3-hexadecanoyl-*sn*-glycerol (*R*)-**5f** (115 mg, 0.273 mmol), (*S*)-ibuprofen (59 mg, 0.287 mmol), CH_2Cl_2 (3 mL), DMAP (26 mg, 0.210 mmol) and EDCI (49 mg, 0.253 mmol). Purification on a silica gel chromatography using pet. ether/ethyl acetate (4:1) as eluent afforded the product (*R*,*S*')-**6f** as a pale-yellow oil, in 98% yield (164 mg, 0.268 mmol). $[\alpha]^{20}_D$ = -0.57 (c. 3.0, CH_2Cl_2). IR (NaCl, ν_{max} / cm^{-1}): 3029 (s), 2925 (vs), 2854 (vs), 1742 (vs), 1513 (s), 1162 (br s). ^1H NMR (400 MHz, $CDCl_3$) δ_H: 7.34-7.25 (m, 3H, Ph-H), 7.22-7.20 (m, 2H, Ibu-2,6 and 2H, Ph-H), 7.07 (d, *J*=8.1 Hz, 2H, Ibu-3,5), 5.27-5.21 (m, 1H, CH *sn*-2), 4.42-4.34 (m, 1H, CH_2 *sn*-3 and 2H, $PhCH_2$), 4.20 (dd, *J*=11.9, 6.6 Hz, 1H, CH_2 *sn*-3), 3.72 (q, *J*=7.2 Hz, 1H, C*H*CH_3), 3.55-3.46 (m, 2H, CH_2 *sn*-1), 2.43 (d, *J*=7.2 Hz, 2H, C*H*$_2$CH(CH_3)$_2$), 2.26 (t, *J*=7.5 Hz, 2H, CH_2COO), 1.83 (nonet, *J*=6.7 Hz, 1H, C*H*(CH_3)$_2$), 1.64-1.54 (m, 2H, C*H*$_2$$CH_2$COO), 1.50 (d, *J*=7.2 Hz, 3H, CHC*H*$_3$), 1.36-1.25 (m, 24H, CH_2), 0.90 (t, *J*=6.8 Hz, 3H,

CH_2CH_3), 0.89 (d, *J*=6.6 Hz, 6H, CH(C*H*$_3$)$_2$) ppm. ^{13}C{H} NMR (101 MHz, $CDCl_3$) δ_C: 174.1 (Ibu), 173.5 (SFA), 140.7, 137.9, 137.6, 129.4 (2), 128.5 (2), 127.8 (2), 127.6 (2), 127.3, 73. 5, 70. 8, 68.4, 62.8, 45.3, 45.2, 34.3, 32.1, 30.3, 29.84, 29.81 (2), 29. 8 (2), 29.6, 29.5, 29.4, 29.3, 25.0, 22.8 (2), 22.5, 18.7, 14.3 ppm. HRMS (ESI) *m*/*z*: [M + Na]$^+$ calcd for $C_{39}H_{60}O_5Na$ 631.4333; found, 631.4336.

3.3.6. Synthesis of 3-*O*-benzyl-2-[(*S*)-2-(4-isobutylphenyl)propanoyl]-1-octanoyl-*sn*-glycerol, (*S*,*S*')-**6b**

The same procedure was followed as described for (*R*,*S*')-**6a** using 3-*O*-benzyl-1-octanoyl-*sn*-glycerol (*S*)-**5b** (102 mg, 0.331 mmol), (*S*)-ibuprofen (75 mg, 0.364 mmol), CH_2Cl_2 (3 mL), DMAP (35 mg, 0.286 mmol) and EDCI (67 mg, 0.350 mmol). Purification on a silica gel chromatography using pet. ether/ethyl acetate (4:1) as eluent afforded the product (*S*,*S*')-**6b** as a pale-yellow oil, in 95% yield (154 mg, 0.314 mmol). $[\alpha]^{20}_D$ = +21.0 (c. 10.0, CH_2Cl_2). IR (NaCl, ν_{max} / cm^{-1}): 3028 (s), 2955 (vs), 2929 (vs), 2869 (vs), 1740 (vs), 1162 (br s). ^{1}H NMR (400 MHz, $CDCl_3$) δ_H: 7.37-7.26 (m, 5H, Ph-H), 7.19 (d, *J*=8.1 Hz, 2H, Ibu-2,6), 7.06 (d, *J*=8.1 Hz, 2H, Ibu-3,5), 5.26-5.20 (m, 1H, CH *sn*-2), 4.51 (AB q, *J*=12.1 Hz, 2H, $PhCH_2$), 4.24 (dd, *J*=11.9, 3.9 Hz, 1H, CH_2 *sn*-1), 4.12 (dd, J=11.9, 6.8 Hz, 1H, CH_2 *sn*-1), 3.72 (q, *J*=7.1 Hz, 1H, C*H*CH_3), 3.63-3.55 (m, 2H, CH_2 *sn*-3), 2.43 (d, *J*=7.2 Hz, 2H, C*H*$_2$CH(CH_3)$_2$), 2.11 (t, *J*=7.6 Hz, 2H, CH_2COO), 1.83 (nonet, *J*=6.7 Hz, 1H, C*H*(CH_3)$_2$), 1.48 (m, 2H, C*H*$_2$$CH_2$COO and 3H, CHC*H*$_3$), 1.33-1.20 (m, 8H, CH_2), 0.90 (t, *J*=6.8 Hz, 3H, CH_2C*H*$_3$), 0.89 (d, *J*=6.6 Hz, 6H, CH(C*H*$_3$)$_2$) ppm. ^{13}C{H} NMR (101 MHz, $CDCl_3$) δ_C: 174.0 (Ibu), 173.3 (SFA), 140.5, 137.7, 137.4, 129.2 (2), 128.4 (2), 127.7 (2), 127.6 (2), 127.1, 73.3, 70.4, 68.3, 62.5, 45.1, 45.0, 33.9, 31.7, 30.2, 29.1, 28.9, 24.7, 22.6 (2), 22.4, 18.4, 14.1 ppm. HRMS (ESI) *m*/*z*: [M + Na]$^+$ calcd for $C_{31}H_{44}O_5Na$ 519.3081; found, 519.3072.

3.3.7. Synthesis of 3-*O*-benzyl-1-decanoyl-2-[(*S*)-2-(4-isobutylphenyl)propanoyl]-*sn*-glycerol, (*S*,*S*')-**6c**

The same procedure was followed as described for (*R*,*S*')-**6a** using 3-*O*-benzyl-1-decanoyl-*sn*-glycerol (*S*)-**5c** (200 mg, 0.590 mmol), (*S*)-ibuprofen (132 mg, 0.640 mmol), CH_2Cl_2 (5.5 mL), DMAP (64 mg, 0.520 mmol) and EDCI (121 mg, 0.630 mmol). Purification on a silica gel chromatography using pet. ether/ethyl acetate (4:1) as eluent afforded the product (*S*,*S*')-**6c** as a pale-yellow oil, in 81% yield (254 mg, 0.480 mmol). $[\alpha]^{20}_D$ = +22.0 (c. 9.5, CH_2Cl_2). IR (NaCl, ν_{max} / cm^{-1}): 3028 (s), 2951 (vs), 2926 (vs), 2855 (vs), 1740 (vs), 1162 (br s). ^{1}H NMR (400 MHz, $CDCl_3$) δ_H: 7.40-7.22 (m, 5H, Ph-H), 7.22- 7.16 (m, 2H, Ibu-2,6), 7.10-7.02 (m, 2H, Ibu-3,5), 5.24 (dtd, *J*=6.8, 5.2, 3.9 Hz, 1H, CH *sn*-2), 4.51 (AB q, *J*=12.1 Hz, 2H, $PhCH_2$), 4.26 (dd, *J*=11.8, 3.9 Hz, 1H, CH_2 *sn*-1), 4.12 (dd, J=11.8, 6.8 Hz, 1H, CH_2 *sn*-1), 3.73 (q, *J*=7.2 Hz, 1H, C*H*CH_3), 3.63-3.50 (m, 2H, CH_2 *sn*-3), 2.44 (d, *J*=7.2 Hz, 2H, C*H*$_2$CH(CH_3)$_2$), 2.12 (t, *J*=7.6 Hz, 2H, CH_2COO), 1.84 (nonet, *J*=6.8 Hz, 1H, C*H*(CH_3)$_2$), 1.55-1.39 (m, 2H, C*H*$_2$$CH_2$COO and 3H, CHC*H*$_3$), 1.30-1.22 (m, 12H, CH_2), 0.89 (t, *J*=7.2 Hz, 3H, CH_2C*H*$_3$), 0.88 (d, *J*=6.6 Hz, 6H, CH(C*H*$_3$)$_2$) ppm. ^{13}C{H} NMR (101 MHz, $CDCl_3$) δ_C: 174.1 (Ibu), 173.4 (SFA), 140.6, 137.9, 137.6, 129.4 (2), 128.5 (2), 127.9 (2), 127.7 (2), 127.3, 73.4, 70.6, 68.4, 62.7, 45.2, 45.2, 34.3, 32.0, 30.3 (2), 29.6, 29.4, 29.3, 25.0, 22.8 (2), 22.5, 18.5, 14.2 ppm. HRMS (ESI) *m*/*z*: [M + Na]$^+$ calcd for $C_{33}H_{48}O_5Na$ 547.3394; found, 547.3390.

3.3.8. Synthesis of 3-*O*-benzyl-1-dodecanoyl-2-[(*S*)-2-(4-isobutylphenyl)propanoyl]-*sn*-glycerol, (*S*,*S*')-**6d**

The same procedure was followed as described for (*R*,*S*')-**6a** using 3-*O*-benzyl-1-dodecanoyl-*sn*-glycerol (*S*)-**5d** (150 mg, 0.411 mmol), (*S*)-ibuprofen (95 mg, 0.460 mmol), CH_2Cl_2 (4 mL), DMAP (45 mg, 0.368 mmol) and EDCI (90 mg, 0.469 mmol). Purification on a silica gel chromatography using pet. ether/ethyl acetate (4:1) as eluent afforded the product (*S*,*S*')-**6d** as a pale-yellow oil, in 84% yield (191 mg, 0.346 mmol). $[\alpha]^{20}_D$ = +19.3 (c. 3.9, CH_2Cl_2). IR (NaCl, ν_{max} / cm^{-1}): 3028 (s), 2954 (vs), 2925 (vs), 2854 (vs), 1740 (vs), 1162 (br s). ^{1}H NMR (400 MHz, $CDCl_3$) δ_H: 7.38-7.26 (m, 5H, Ph-H), 7.19 (d, *J*=8.1 Hz, 2H, Ibu-2,6), 7.06 (d, *J*=8.1 Hz, 2H, Ibu-3,5), 5.26-5.20 (m, 1H, CH *sn*-2), 4.51 (AB q, *J*=12.1 Hz, 2H, $PhCH_2$), 4.24 (dd, *J*=11.8, 3.9 Hz, 1H, CH_2 *sn*-1), 4.12 (dd, J=11.8, 6.8 Hz, 1H, CH_2 *sn*-1), 3.72 (q, *J*=7.1 Hz, 1H, C*H*CH_3), 3.64-3.55 (m, 2H, CH_2 *sn*-3), 2.43 (d, *J*=7.2 Hz, 2H, C*H*$_2$CH(CH_3)$_2$), 2.11 (t, *J*=7.6 Hz, 2H, CH_2COO), 1.83 (nonet, *J*=6.7 Hz, 1H,

CH(CH$_3$)$_2$), 1.48 (m, 2H, CH_2CH$_2$COO and 3H, CHCH_3), 1.33-1.20 (m, 16H, CH$_2$), 0.90 (t, *J*=6.9 Hz, 3H, CH$_2$CH_3), 0.88 (d, *J*=6.6 Hz, 6H, CH(CH_3)$_2$) ppm. ^{13}C{H} NMR (101 MHz, CDCl$_3$) δ_C: 174.0 (Ibu), 173.2 (SFA), 140.4, 137.7, 137.4, 129.5 (2), 128.4 (2), 127.9 (2), 127.6 (2), 127.1, 73.3, 70.4, 68.3, 62.5, 45.3, 45.2, 33.9, 31.8, 30.1, 29.4 (2), 29.3 (2), 29.1 (2), 24.7, 22.6 (2), 22.4, 18.4, 14.1 ppm. HRMS (ESI) *m*/*z*: [M + Na]$^+$ calcd for $C_{35}H_{52}O_5Na$ 575.3707; found, 575.3708.

3.3.9. Synthesis of 3-*O*-benzyl-2-[(*S*)-2-(4-isobutylphenyl)propanoyl]-1-tetradecanoyl-*sn*-glycerol, (*S*,*S*')-**6e**

The same procedure was followed as described for (*R*,*S*')-**6a** using 3-*O*-benzyl-1-tetradecanoyl-*sn*-glycerol (*S*)-**5e** (50 mg, 0.127 mmol), (*S*)-ibuprofen (30 mg, 0.145 mmol), CH_2Cl_2 (2 mL), DMAP (16 mg, 0.131 mmol) and EDCI (30 mg, 0.156 mmol). Purification on a silica gel chromatography using pet. ether/ethyl acetate (9:1) as eluent afforded the product (*S*,*S*')-**6e** as a pale-yellow oil, in 91% yield (69 mg, 0.119 mmol). $[\alpha]^{20}_D$ = +17.9 (c. 7.2, CH_2Cl_2). IR (NaCl, ν_{max} / cm^{-1}): 2955 (vs), 2929 (vs), 2854 (vs), 1740 (vs), 1159 (br s). ^{1}H NMR (400 MHz, CDCl$_3$) δ_H: 7.34-7.25 (m, 5H, Ph-H), 7.19 (d, *J*=8.1 Hz, 2H, Ibu-2,6), 7.06 (d, *J*=8.1 Hz, 2H, Ibu-3,5), 5.27-5.21 (m, 1H, CH *sn*-2), 4.51 (AB q, *J*=12.1 Hz, 2H, PhCH$_2$), 4.24 (dd, *J*=11.8, 3.9 Hz, 1H, CH$_2$ *sn*-1), 4.12 (dd, J=11.8, 6.8 Hz, 1H, CH$_2$ *sn*-1), 3.72 (q, *J*=7.2 Hz, 1H, CHCH$_3$), 3.55-3.46 (m, 2H, CH$_2$ *sn*-3), 2.43 (d, *J*=7.2 Hz, 2H, CH_2CH(CH$_3$)$_2$), 2.26 (t, *J*=7.5 Hz, 2H, CH$_2$COO), 1.83 (nonet, *J*=6.7 Hz, 1H, CH(CH$_3$)$_2$), 1.64-1.54 (m, 2H, CH_2CH$_2$COO and 3H, CHCH_3), 1.36-1.25 (m, 20H, CH$_2$), 0.90 (t, *J*=6.8 Hz, 3H, CH$_2$CH_3), 0.89 (d, *J*=6.6 Hz, 6H, CH(CH_3)$_2$) ppm. ^{13}C{H} NMR (101 MHz, CDCl$_3$) δ_C: 174.12 (Ibu), 173.4 (SFA), 140.6, 137.9, 137.6, 129.4 (2), 128.6 (2), 127.9 (2), 127.7 (2), 127.3, 73.5, 70.6, 68.5, 62.7, 45.2, 45.2, 34.1, 32.1, 30.3, 29.83 (2), 29.80, 29.7, 29.5, 29.4, 29.3, 24.9, 22.8 (2), 22.5, 18.5, 14.3 ppm. HRMS (ESI) *m*/*z*: [M + Na]$^+$ calcd for $C_{37}H_{56}O_5Na$ 603.4020; found, 603.4009.

3.3.10. Synthesis of 3-*O*-benzyl-1-hexadecanoyl-2-[(*S*)-2-(4-isobutylphenyl)propanoyl]-*sn*-glycerol, (*S*,*S*')-**6f**

The same procedure was followed as described for (*R*,*S*')-**6a** using 3-*O*-benzyl-1-hexaadecanoyl-*sn*-glycerol (*S*)-**5f** (45 mg, 0.107 mmol), (*S*)-ibuprofen (24 mg, 0.118 mmol), CH_2Cl_2 (1 mL), DMAP (12 mg, 0.094 mmol) and EDCI (22 mg, 0.113 mmol). Purification on a silica gel chromatography using pet. ether/ethyl acetate (7:3) as eluent afforded the product (*S*,*S*')-**6f** as a pale-yellow oil, in 84% yield (55 mg, 0.090 mmol). $[\alpha]^{20}_D$ = +11.6 (c. 5.0, CH_2Cl_2). IR (NaCl, ν_{max} / cm^{-1}): 3028 (s), 2955 (vs), 2924 (vs), 2854 (vs), 1740(vs), 1686 (s, 1163 (br s). ^{1}H NMR (400 MHz, CDCl$_3$) δ_H: 7.37-7.25 (m, 5H, Ph-H), 7.19 (d, *J*=8.1 Hz, 2H, Ibu-2,6), 7.06 (d, *J*=8.1 Hz, 2H, Ibu-3,5), 5.27-5.19 (m, 1H, CH *sn*-2), 4.51 (AB q, *J*=12.1 Hz, 2H, PhCH$_2$), 4.25 (dd, *J*=11.8, 3.9 Hz, 1H, CH$_2$ *sn*-1), 4.13 (dd, J=11.8, 6.8 Hz, 1H, CH$_2$ *sn*-1), 3.72 (q, *J*=7.2 Hz, 1H, CHCH$_3$), 3.64-3.55 (m, 2H, CH$_2$ *sn*-3), 2.43 (d, *J*=7.2 Hz, 2H, CH_2CH(CH$_3$)$_2$), 2.11 (t, *J*=7.5 Hz, 2H, CH$_2$COO), 1.83 (nonet, *J*=6.7 Hz, 1H, CH(CH$_3$)$_2$), 1.55-1.46 (m, 2H, CH_2CH$_2$COO and 3H, CHCH_3), 1.36-1.25 (m, 24H, CH$_2$), 0.90 (t, *J*=6.8 Hz, 3H, CH$_2$CH_3), 0.89 (d, *J*=6.6 Hz, 6H, CH(CH_3)$_2$) ppm. ^{13}C{H} NMR (101 MHz, CDCl$_3$) δ_C: 174.2 (Ibu), 173.4 (SFA), 140.6, 137.9, 137.6, 129.4 (2), 128.6 (2), 127.9 (2), 127.7 (2), 127.3, 73.5, 70.6, 68.5, 62.7, 45.5, 45.2, 34.1, 32.1, 30.3, 29.84 (2), 29.81 (2), 29.78, 29.7, 29.5, 29.4, 29.3, 24.9, 22.8 (2), 22.5, 18.5, 14.3 ppm. HRMS (ESI) *m*/*z*: [M + Na]$^+$ calcd for $C_{39}H_{60}O_5Na$ 631.4333; found, 631.4332.

3.3.11. Synthesis of 1-*O*-benzyl-2-[(*S*)-2-(6-methoxynaphthalen-2-yl)propanoyl]-3-octanoyl-*sn*-glycerol, (*R*,*S*')-**7b**

The same procedure was followed as described for (*R*,*S*')-**7a** using 1-*O*-benzyl-3-octanoyl-*sn*-glycerol (*R*)-**5b** (109 mg, 0.353 mmol), (*S*)-naproxen (90 mg, 0.391 mmol), CH_2Cl_2 (3 mL), DMAP (35 mg, 0.285 mmol) and EDCI (66 mg, 0.344 mmol). Purification on a silica gel chromatography using pet. ether/ethyl acetate (7:3) as eluent afforded the product (*R*,*S*')-**7b** as a pale-yellow oil, in 95% yield (175 mg, 0.336 mmol). $[\alpha]^{20}_D$ = -3.02 (c. 17.5, CH_2Cl_2). IR (NaCl, ν_{max} / cm^{-1}): 3031 (vs), 2955 (vs), 2931 (vs), 2857 (vs), 1739 (vs), 1634 (vs), 1607 (vs), 1175 (br s). ^{1}H NMR (400 MHz, CDCl$_3$) δ_H: 7.68-7.66 (m, 3H, H-1,4,8 Nap), 7.41 (dd, *J*=8.5, 1.7 Hz, 1H, H-3 Nap), 7.25-7.19 (m, 3H, Ph-H), 7.14 (dd, *J*=8.9, 2.6 Hz, 1H, H-7 Nap), 7.12-

7.09 (m, 2H, Ph-H and 1H, Nap-5), 5.30-5.23 (m, 1H, CH *sn*-2), 4.36-4.28 (m, 1H, CH_2 *sn*-3 and 2H, $PhCH_2$), 4.21 (dd, *J*=11.9, 6.6 Hz, 1H, CH_2 *sn*-3), 3.91 (s, 3H, OCH_3), 3.89 (q, *J*=7.2, 1H, C*H*CH_3), 3.54-3.44 (m, 2H, CH_2 *sn*-1), 2.22 (t, *J*=7.2 Hz, 2H, CH_2COO), 1.60-1.54 (m, 2H, C*H*$_2CH_2COO$ and 3H, CHC*H*$_3$), 1.33-1.21 (m, 8H, CH_2), 0.90 (t, *J*=6.9 Hz, 3H, CH_2C*H*$_3$) ppm. ^{13}C{H} NMR (101 MHz, $CDCl_3$) δ_C: 174.0 (Nap), 173.5 (SFA), 157.8, 137.8, 135.6, 133.8, 129.4, 129.1, 128.4 (2), 127.7, 127.5 (2), 127.2, 126.3, 126.1, 119.1, 105.7, 73.4, 70.9, 68.4, 62.7, 55.4, 45.6, 34.2, 31.8, 29.2, 29.0, 25.0, 22.7, 18.6, 14.2 ppm. HRMS (ESI) *m*/*z*: [M + Na]$^+$ calcd for $C_{32}H_{40}O_6Na$ 543.2717; found, 543.2720.

3.3.12. Synthesis of 1-*O*-benzyl-3-decanoyl-2-[(*S*)-2-(6-methoxynaphthalen-2-yl)propanoyl]-*sn*-glycerol, (*R*,*S*')-**7c**

The same procedure was followed as described for (*R*,*S*')-**7a** using 1-*O*-benzyl-3-decanoyl-*sn*-glycerol (*R*)-**5c** (100 mg, 0.297 mmol), (*S*)-naproxen (82 mg, 0.358 mmol), CH_2Cl_2 (3 mL), DMAP (32 mg, 0.261 mmol) and EDCI (60 mg, 0.315 mmol). Purification on a silica gel chromatography using pet. ether/ethyl acetate (4:1) as eluent followed by recrystallization from n-hexane afforded the product (*R*,*S*')-**7c** as very fine white needles, in 96% yield (157 mg, 0.286 mmol). Mp. 37.4-37.8°C. $[\alpha]^{20}_D$ = -3.23 (c. 3.0, CH_2Cl_2). IR (NaCl, ν_{max} / cm^{-1}): 3061 (s), 3031 (s), 2923 (vs), 2854 (vs), 1732 (vs), 1634 (vs), 1607 (vs), 1184 (br s). ^{1}H NMR (400 MHz, $CDCl_3$) δ_H: 7.68-7.66 (m, 3H, H-1,4,8 Nap), 7.41 (dd, *J*=8.5, 1.7 Hz, 1H, H-3 Nap), 7.25-7.19 (m, 3H, Ph-H), 7.14 (dd, *J*=8.9, 2.6 Hz, 1H, H-7 Nap), 7.11-7.09 (m, 2H, Ph-H and 1H, Nap-5), 5.30-5.23 (m, 1H, CH *sn*-2), 4.36 (dd, J=11.9, 3.8 Hz, 1H, CH_2 *sn*-3), 4.36-4.28 (m, 2H, $PhCH_2$), 4.21 (dd, *J*=11.9, 6.6 Hz, 1H, CH_2 *sn*-3), 3.91 (s, 3H, OCH_3), 3.89 (q, *J*=7.2, 1H, C*H*CH_3), 3.54-3.44 (m, 2H, CH_2 *sn*-1), 2.22 (t, *J*=7.7 Hz, 2H, CH_2COO), 1.60-1.53 (m, 2H, C*H*$_2CH_2COO$), 1.59 (d, *J*=7.2 Hz, 3H, CHC*H*$_3$), 1.33-1.21 (m, 12H, CH_2), 0.90 (t, *J*=6.9 Hz, 3H, CH_2C*H*$_3$) ppm. ^{13}C{H} NMR (101 MHz, $CDCl_3$) δ_C: 174.1 (Nap), 173.5 (SFA), 157.8, 137.8, 135.6, 133.9, 129.4, 129.1, 128.4 (2), 127.7, 127.5 (2), 127.2, 126.4, 126.1, 119.1, 105.7, 73.4, 70.9, 68.4, 62.7, 55.4, 45.6, 34.2, 32.0, 29.6 (2), 29.4, 29.3, 25.0, 22.8, 18.6, 14.3 ppm. HRMS (ESI) *m*/*z*: [M + Na]$^+$ calcd for $C_{34}H_{44}O_6Na$ 571.3030; found, 571.3026.

3.3.13. Synthesis of 1-*O*-benzyl-3-dodecanoyl-2-[(*S*)-2-(6-methoxynaphthalen-2-yl)propanoyl]-*sn*-glycerol, (*R*,*S*')-**7d**

The same procedure was followed as described for (*R*,*S*')-**7a** using 1-*O*-benzyl-3-dodecanoyl-*sn*-glycerol (*R*)-**5d** (100 mg, 0.289 mmol), (*S*)-naproxen (80 mg, 0.348 mmol), CH_2Cl_2 (3 mL), DMAP (31 mg, 0.254 mmol) and EDCI (59 mg, 0.307 mmol). Purification on a silica gel chromatography using pet. ether/ethyl acetate (4:1) as eluent followed by recrystallization from n-hexane afforded the product (*R*,*S*')-**7d** as very fine white needles, in 92% yield (148 mg, 0.265 mmol). Mp. 36.5-37.1°C. $[\alpha]^{20}_D$ = -2.00 (c. 4.3, CH_2Cl_2). IR (NaCl, ν_{max} / cm^{-1}): 2917 (vs), 2851 (vs), 1742 (vs), 1720 (vs), 1606 (vs), 1184 (br s). ^{1}H NMR (400 MHz, $CDCl_3$) δ_H: 7.68-7.66 (m, 3H, H-1,4,8 Nap), 7.41 (dd, *J*=8.5, 1.7 Hz, 1H, H-3 Nap), 7.25-7.19 (m, 3H, Ph-H), 7.14 (dd, *J*=8.9, 2.6 Hz, 1H, H-7 Nap), 7.11-7.09 (m, 2H, Ph-H and 1H, Nap-5), 5.30-5.23 (m, 1H, CH *sn*-2), 4.36 (dd, J=11.9, 3.8 Hz, 1H, CH_2 *sn*-3), 4.36-4.28 (m, 2H, $PhCH_2$), 4.21 (dd, *J*=11.9, 6.6 Hz, 1H, CH_2 *sn*-3), 3.91 (s, 3H, OCH_3), 3.89 (q, *J*=7.1, 1H, C*H*CH_3), 3.54-3.44 (m, 2H, CH_2 *sn*-1), 2.22 (t, *J*=7.5 Hz, 2H, CH_2COO), 1.60-1.53 (m, 2H, C*H*$_2CH_2COO$), 1.59 (d, *J*=7.2 Hz, 3H, CHC*H*$_3$), 1.33-1.21 (m, 16H, CH_2), 0.90 (t, *J*=6.9 Hz, 3H, CH_2C*H*$_3$) ppm. ^{13}C{H} NMR (101 MHz, $CDCl_3$) δ_C: 174.1 (Nap), 173.5 (SFA), 157.8, 137.8, 135.6, 133.9, 129.4, 129.1, 128.4 (2), 127.7, 127.5 (2), 127.2, 126.4, 126.1, 119.1, 105.7, 73.4, 70.9, 68.4, 62.7, 55.4, 45.6, 34.2, 32.1, 29.8 (2), 29.6, 29.5, 29.4, 29.3, 25.0, 22.8, 18.6, 14.3 ppm. HRMS (ESI) *m*/*z*: [M + Na]$^+$ calcd for $C_{36}H_{48}O_6Na$ 599.3343; found, 599.3349.

3.3.14. Synthesis of 1-*O*-benzyl-2-[(*S*)-2-(6-methoxynaphthalen-2-yl)propanoyl]-3-tetradecanoyl-*sn*-glycerol, (*R*,*S*')-**7e**

The same procedure was followed as described for (*R*,*S*')-**7a** using 1-*O*-benzyl-3-tetradecanoyl-*sn*-glycerol (*R*)-**5e** (100 mg, 0.255 mmol), (*S*)-naproxen (71 mg, 0.307 mmol), CH_2Cl_2 (2.6 mL), DMAP (27 mg, 0.224 mmol) and EDCI (52 mg, 0.271 mmol). Purification on a silica gel chromatography using pet. ether/ethyl acetate (4:1) as eluent followed by recrystallization from n-hexane afforded the product (*R*,*S*')-**7e** as very fine white powder, in 98% yield (151 mg, 0.250 mmol). Mp. 45.8-46.5°C. $[\alpha]^{20}_D$ = -1.33 (c. 4.5, CH_2Cl_2). IR (NaCl, ν_{max} / cm^{-1}): 2917 (vs), 2850 (vs), 1742 (vs), 1723 (vs), 1605 (s), 1157 (br s). ^1H NMR (400 MHz, $CDCl_3$) δ_H: 7.68-7.66 (m, 3H, H-1,4,8 Nap), 7.41 (dd, *J*=8.5, 1.6 Hz, 1H, H-3 Nap), 7.25-7.19 (m, 3H, Ph-H), 7.14 (dd, *J*=8.9, 2.6 Hz, 1H, H-7 Nap), 7.11-7.08 (m, 2H, Ph-H and 1H, Nap-5), 5.32-5.24 (m, 1H, CH *sn*-2), 4.36 (dd, J=11.9, 3.8 Hz, 1H, CH_2 *sn*-3), 4.36-4.28 (m, 2H, $PhCH_2$), 4.21 (dd, *J*=11.9, 6.6 Hz, 1H, CH_2 *sn*-3), 3.91 (s, 3H, OCH_3), 3.89 (q, *J*=7.1, 1H, C*H*CH_3), 3.54-3.44 (m, 2H, CH_2 *sn*-1), 2.22 (t, *J*=7.5 Hz, 2H, CH_2COO), 1.60-1.53 (m, 2H, C*H*$_2CH_2COO$), 1.59 (d, *J*=7.2 Hz, 3H, CHC*H*$_3$), 1.33-1.21 (m, 20H, CH_2), 0.90 (t, *J*=6.8 Hz, 3H, CH_2C*H*$_3$) ppm. $^{13}C\{H\}$ NMR (101 MHz, $CDCl_3$) δ_C: 174.1 (Nap), 173.5 (SFA), 157.8, 137.8, 135.6, 133.9, 129.4, 129.1, 128.4 (2), 127.7, 127.5 (2), 127.3, 126.4, 126.1, 119.1, 105.7, 73.4, 70.9, 68.4, 62.7, 55.4, 45.6, 34.2, 32.1, 29.8 (2), 29.8 (2), 29.6, 29.5, 29.4, 29.3, 25.0, 22.8, 18.6, 14.3 ppm. HRMS (ESI) *m*/*z*: $[M + Na]^+$ calcd for $C_{38}H_{52}O_6Na$ 627.3656; found, 627.3647.

3.3.15. Synthesis of 1-*O*-benzyl-3-hexadecanoyl-2-[(*S*)-2-(6-methoxynaphthalen-2-yl)propanoyl]-*sn*-glycerol, (*R*,*S*')-**7f**

The same procedure was followed as described for (*R*,*S*')-**7a** using 1-*O*-benzyl-3-hexadecanoyl-*sn*-glycerol (*R*)-**5e** (100 mg, 0.238 mmol), (*S*)-naproxen (66 mg, 0.287 mmol), CH_2Cl_2 (2.5 mL), DMAP (26 mg, 0.210 mmol) and EDCI (49 mg, 0.253 mmol). Purification on a silica gel chromatography using pet. ether/ethyl acetate (4:1) as eluent followed by recrystallization from n-hexane afforded the product (*R*,*S*')-**7f** as very fine white powder, in 96% yield (145 mg, 0.229 mmol). Mp. 50.6-51.6°C. $[\alpha]^{20}_D$ = -2.00 (c. 3.3, CH_2Cl_2). IR (NaCl, ν_{max} / cm^{-1}): 2917 (vs), 2849 (vs), 1743 (vs), 1727 (vs, 1634, 1605 (vs), 1156 (br s). ^1H NMR (400 MHz, $CDCl_3$) δ_H: 7.68-7.66 (m, 3H, H-1,4,8 Nap), 7.41 (dd, *J*=8.5, 1.7 Hz, 1H, H-3 Nap), 7.25-7.19 (m, 3H, Ph-H), 7.14 (dd, *J*=8.9, 2.6 Hz, 1H, H-7 Nap), 7.11-7.08 (m, 2H, Ph-H and 1H, Nap-5), 5.30-5.23 (m, 1H, CH *sn*-2), 4.36 (dd, J=11.9, 3.8 Hz, 1H, CH_2 *sn*-3), 4.36-4.28 (m, 2H, $PhCH_2$), 4.21 (dd, *J*=11.9, 6.6 Hz, 1H, CH_2 *sn*-3), 3.91 (s, 3H, OCH_3), 3.89 (q, *J*=7.1, 1H, C*H*CH_3), 3.54-3.44 (m, 2H, CH_2 *sn*-1), 2.22 (t, *J*=7.8 Hz, 2H, CH_2COO), 1.60-1.53 (m, 2H, C*H*$_2CH_2COO$), 1.59 (d, *J*=7.2 Hz, 3H, CHC*H*$_3$), 1.33-1.21 (m, 24H, CH_2), 0.90 (t, *J*=6.9 Hz, 3H, CH_2C*H*$_3$) ppm. $^{13}C\{H\}$ NMR (101 MHz, $CDCl_3$) δ_C: 174.1 (Nap), 173.5 (SFA), 157.8, 137.8, 135.6, 133.85, 129.4, 129.1, 128.4 (2), 127.7, 127.5 (2), 127.2, 126.4, 126.1, 119.1, 105.7, 73.4, 70.9, 68.4, 62.7, 55.4, 45.6, 34.2, 32.1, 29.9 (2), 29.8 (2), 29.8 (2), 29.6, 29.5, 29.4, 29.3, 25.0, 22.8, 18.6, 14.3 ppm. HRMS (ESI) *m*/*z*: $[M + Na]^+$ calcd for $C_{40}H_{56}O_6Na$ 655.3969; found, 655.3960.

3.3.16. Synthesis of 3-*O*-benzyl-2-[(*S*)-2-(6-methoxynaphthalen-2-yl)propanoyl]-1-octanoyl-*sn*-glycerol, (*S*,*S*')-**7b**

To a solution of 3-*O*-benzyl-1-octanoyl-*sn*-glycerol (*S*)-**5b** (100 mg, 0.324 mmol) and (*S*)-naproxen (82 mg, 0.357 mmol) in CH_2Cl_2 (3 mL) were added DMAP (35 mg, 0.285 mmol) and EDCI (66 mg, 0.344 mmol). The solution was stirred on a magnetic stirrer at room temperature for 31 h. The reaction was disconnected by passing the reaction mixture through a short column packed with silica gel by use of Et_2O/CH_2Cl_2 (1:9). The solvent was removed in vacuo on a rotary evaporator. The concentrate was applied to a silica gel chromatography using petroleum ether/ethyl acetate (8.5:1.5) as eluent, which afforded the product (*S*,*S*')-**7b** as a clear oil, in 97% yield (164 mg, 0.314 mmol). $[\alpha]^{20}_D$ = +4.82 (c. 3.2, CH_2Cl_2). IR (NaCl, ν_{max} / cm^{-1}): 3030 (s), 2931 (vs), 2857 (vs), 1739 (vs), 1634 (vs), 1162 (br s). ^1H NMR (400 MHz, $CDCl_3$) δ_H: 7.73-7.63 (m, 3H, H-1,4,8 Nap), 7.41 (dd, *J*=8.6, 1.8 Hz, 1H, H-3 Nap), 7.37-7.26 (m, 5H, Ph-H), 7.14 (dd, *J*=8.9, 2.5 Hz, 1H, H-7 Nap), 7.10 (d, *J*=2.5 Hz, 1H, Nap-5), 5.26-5.20 (m, 1H, CH *sn*-2), 4.51 (AB q, *J*=12.1 Hz, 2H, $PhCH_2$), 4.24 (dd, *J*=11.9, 3.7 Hz, 1H, CH_2 *sn*-1), 4.12 (dd, *J*=11.9, 6.9 Hz, 1H, CH_2 *sn*-1), 3.91 (s, 3H, OCH_3), 3.72 (q, *J*=7.1, 1H, C*H*CH_3), 3.61-3.59 (m, 2H, CH_2 *sn*-3), 1.94-1.87 (m, 2H, CH_2COO), 1.58 (d, *J*=7.2 Hz, 3H,

$CHCH_3$), 1.40-1.31 (m, 2H, CH_2CH_2COO), 1.23-1.05 (m, 8H, CH_2), 0.85 (t, *J*=7.2 Hz, 3H, CH_2CH_3) ppm. $^{13}C\{H\}$ NMR (101 MHz, $CDCl_3$) δ_C: 174.0 (Nap), 173.3 (SFA), 157.8, 137.8, 135.6, 133.8, 129.4, 129.1, 128.5 (2), 127.9, 127.7 (2), 127.2, 126.4, 126.1, 119.1, 105.7, 73.4, 70.7, 68.5, 62.6, 55.4, 45.6, 33.8, 31.3, 29.5, 29.4, 24.4, 22.4, 18.5, 14.1 ppm. HRMS (ESI) *m*/*z*: $[M + Na]^+$ calcd for $C_{32}H_{40}O_6Na$ 543.2717; found, 543.2720.

3.3.17. Synthesis of 3-*O*-benzyl-1-decanoyl-2-[(*S*)-2-(6-methoxynaphthalen-2-yl)propanoyl]-*sn*-glycerol, (*S*,*S*')-**7c**

The same procedure was followed as described for (S,*S*')-**7b** using 3-*O*-benzyl-1-decanoyl-*sn*-glycerol (*S*)-**5c** (102 mg, 0.297 mmol), (*S*)-naproxen (75 mg, 0.327 mmol), CH_2Cl_2 (10.0 mL), DMAP (32 mg, 0.261 mmol) and EDCI (65 mg, 0.356 mmol). Purification on a silica gel chromatography using pet. ether/ethyl acetate (4:1) as eluent afforded the product (*R*,*S*')-**7c** as a clear oil, in 92% yield (151 mg, 0.273 mmol). $[\alpha]^{20}_D$ = +6.69 (c. 3.0, CH_2Cl_2). IR (NaCl, ν_{max} / cm^{-1}): 2945 (vs), 2927 (vs), 2855 (vs), 1739 (vs), 1634 (s), 1607 (s), 1177 (br s). ^1H NMR (400 MHz, $CDCl_3$) δ_H: 7.73-7.63 (m, 3H, H-1,4,8 Nap), 7.41 (dd, *J*=8.6, 1.8 Hz, 1H, H-3 Nap), 7.37-7.26 (m, 5H, Ph-H), 7.14 (dd, *J*=8.9, 2.5 Hz, 1H, H-7 Nap), 7.10 (d, *J*=2.5 Hz, 1H, Nap-5), 5.26-5.20 (m, 1H, CH *sn*-2), 4.51 (AB q, *J*=12.1 Hz, 2H, $PhCH_2$), 4.23 (dd, *J*=11.9, 3.7 Hz, 1H, CH_2 *sn*-1), 4.12 (dd, *J*=11.9, 6.9 Hz, 1H, CH_2 *sn*-1), 3.91 (s, 3H, OCH_3), 3.88 (q, *J*=7.1, 1H, $CHCH_3$), 3.61-3.59 (m, 2H, CH_2 *sn*-3), 1.94-1.87 (m, 2H, CH_2COO), 1.58 (d, *J*=7.2 Hz, 3H, $CHCH_3$), 1.40-1.31 (m, 2H, CH_2CH_2COO), 1.28-1.12 (m, 12H, CH_2), 0.85 (t, *J*=7.2 Hz, 3H, CH_2CH_3) ppm. $^{13}C\{H\}$ NMR (101 MHz, $CDCl_3$) δ_C: 174.1 (Nap), 173.4 (SFA), 157.8, 137.8, 135.6, 133.8, 129.4, 129.1, 128.5 (2), 127.9, 127.7 (2), 127.2, 126.4, 126.1, 119.1, 105.7, 73.5, 70.7, 68.5, 62.6, 55.4, 45.6, 33.9, 32.0, 29.6, 29.4, 29.4, 29.2, 24.8, 22.8, 18.5, 14.3 ppm. HRMS (ESI) *m*/*z*: $[M + Na]^+$ calcd for $C_{34}H_{44}O_6Na$ 571.3030; found, 571.3032.

3.3.18. Synthesis of 3-*O*-benzyl-1-dodecanoyl-2-[(*S*)-2-(6-methoxynaphthalen-2-yl)propanoyl]-*sn*-glycerol, (*S*,*S*')-**7d**

The same procedure was followed as described for (*S*,*S*')-**7b** using 3-*O*-benzyl-1-dodecanoyl-*sn*-glycerol (*S*)-**5d** (41 mg, 0.112 mmol), (*S*)-naproxen (30 mg, 0.130 mmol), CH_2Cl_2 (2 mL), DMAP (13 mg, 0.106 mmol) and EDCI (25 mg, 0.130 mmol). Purification on a silica gel chromatography using pet. ether/ethyl acetate (9:1) as eluent followed by recrystallization from n-hexane afforded the product (*S*,*S*')-**7d** as very fine white needles, in 92% yield (148 mg, 0.265 mmol). Mp. 34.1-34.6°C. $[\alpha]^{20}_D$ = +17.1 (c. 5.8, CH_2Cl_2). IR (NaCl, ν_{max} / cm^{-1}): 3009 (vs), 2982 (vs), 2948 (vs), 2917 (vs), 2848 (vs), 2804 (vs), 1734 (vs), 1631 (s), 1605 (s), 1083 (br s). ^1H NMR (400 MHz, $CDCl_3$) δ_H: 7.70-7.63 (m, 3H, H-1,4,8 Nap), 7.41 (dd, *J*=8.6, 1.8 Hz, 1H, H-3 Nap), 7.37-7.27 (m, 5H, Ph-H), 7.14 (dd, *J*=8.9, 2.5 Hz, 1H, H-7 Nap), 7.10 (d, *J*=2.5 Hz, 1H, Nap-5), 5.26-5.20 (m, 1H, CH *sn*-2), 4.51 (AB q, *J*=12.1 Hz, 2H, $PhCH_2$), 4.24 (dd, *J*=11.9, 3.7 Hz, 1H, CH_2 *sn*-1), 4.13 (dd, *J*=11.9, 6.9 Hz, 1H, CH_2 *sn*-1), 3.91 (s, 3H, OCH_3), 3.88 (q, *J*=7.1, 1H, $CHCH_3$), 3.61-3.59 (m, 2H, CH_2 *sn*-3), 2.02-1.87 (m, 2H, CH_2COO), 1.58 (d, *J*=7.2 Hz, 3H, $CHCH_3$), 1.40-1.31 (m, 2H, CH_2CH_2COO), 1.28-1.05 (m, 16H, CH_2), 0.85 (t, *J*=6.8 Hz, 3H, CH_2CH_3) ppm. $^{13}C\{H\}$ NMR (101 MHz, $CDCl_3$) δ_C: 174.1 (Nap), 173.4 (SFA), 157.8, 137.8, 135.5, 133.8, 129.4, 129.0, 128.5 (2), 127.9, 127.7 (2), 127.5, 127.2, 126.0, 119.1, 105.7, 73.4, 70.7, 68.5, 62.6, 55.4, 45.5, 33.9, 32.0, 29.8 (2), 29.6, 29.5, 29.4, 29.2, 24.7, 22.8, 18.5, 14.2 ppm. HRMS (ESI) *m*/*z*: $[M + Na]^+$ calcd for $C_{36}H_{48}O_6Na$ 599.3343; found, 599.3334.

3.3.19. Synthesis of 3-*O*-benzyl-2-[(*S*)-2-(6-methoxynaphthalen-2-yl)propanoyl]-1-tetradecanoyl-*sn*-glycerol, (*S*,*S*')-**7e**

The same procedure was followed as described for (*S*,*S*')-**7b** using 1-*O*-benzyl-3-tetradecanoyl-*sn*-glycerol (*S*)-**5e** (50 mg, 0.127 mmol), (*S*)-naproxen (34 mg, 0.147 mmol), CH_2Cl_2 (2.3 mL), DMAP (15 mg, 0.121 mmol) and EDCI (28 mg, 0.145 mmol). Purification on a silica gel chromatography using pet. ether/ethyl acetate (8.5:1.5) as eluent followed by recrystallization from n-hexane afforded the product (*S*,*S*')-**7e** as very fine white needles, in 75% yield (57 mg, 0.095 mmol). Mp. 45.2-45.9°C. $[\alpha]^{20}_D$ = +15.3 (c. 5.2,

CH_2Cl_2). IR (NaCl, ν_{max} / cm^{-1}): 2952 (vs), 2913 (vs), 2869 (vs), 2848 (vs), 1732 (vs), 1631, 1604 (s), 1115 (br s). ^1H NMR (400 MHz, $CDCl_3$) δ_H: 7.70-7.63 (m, 3H, H-1,4,8 Nap), 7.41 (dd, *J*=8.6, 1.8 Hz, 1H, H-3 Nap), 7.37-7.27 (m, 5H, Ph-H), 7.14 (dd, *J*=8.9, 2.5 Hz, 1H, H-7 Nap), 7.10 (d, *J*=2.5 Hz, 1H, Nap-5), 5.26-5.20 (m, 1H, CH *sn*-2), 4.51 (AB q, *J*=12.1 Hz, 2H, $PhCH_2$), 4.24 (dd, *J*=11.9, 3.7 Hz, 1H, CH_2 *sn*-1), 4.13 (dd, *J*=11.9, 6.9 Hz, 1H, CH_2 *sn*-1), 3.91 (s, 3H, OCH_3), 3.88 (q, *J*=7.1, 1H, C*H*CH_3), 3.61-3.59 (m, 2H, CH_2 *sn*-3), 2.02-1.87 (m, 2H, CH_2COO), 1.58 (d, *J*=7.2 Hz, 3H, $CHCH_3$), 1.42-1.31 (m, 2H, CH_2CH_2COO), 1.28-1.05 (m, 20H, CH_2), 0.85 (t, *J*=6.8 Hz, 3H, CH_2CH_3) ppm. $^{13}C\{H\}$ NMR (101 MHz, $CDCl_3$) δ_C: 174.1 (Nap), 173.4 (SFA), 157.6, 137.7, 135.4, 133.7, 129.2, 129.0, 128.5 (2), 127.9, 127.7 (2), 127.5, 127.2, 126.0, 119.1, 105.7, 73.4, 70.7, 68.45, 62.6, 55.4, 45.5, 33.9, 32.0, 29.8 (2), 29.8 (2), 29.6, 29.5, 29.4, 29.2, 24.7, 22.8, 18.5, 14.2 ppm. HRMS (ESI) *m*/*z*: $[M + Na]^+$ calcd for $C_{38}H_{52}O_6Na$ 627.3656; found, 627.3635.

3.3.20. Synthesis of 3-*O*-benzyl-1-hexadecanoyl-2-[(*S*)-2-(6-methoxynaphthalen-2-yl)propanoyl]-*sn*-glycerol, (*S,S*')-**7f**

The same procedure was followed as described for (*S*,*S*')-**7b** using 3-*O*-benzyl-1-hexadecanoyl-*sn*-glycerol (*R*)-**5f** (45 mg, 0.107 mmol), (*S*)-naproxen (27 mg, 0.118 mmol), CH_2Cl_2 (1 mL), DMAP (12 mg, 0.094 mmol) and EDCI (22 mg, 0.113 mmol). Purification on a silica gel chromatography using pet. ether/ethyl acetate (7:3) as eluent followed by recrystallization from n-hexane afforded the product (*S*,*S*')-**7f** as a very fine white powder, in 77% yield (52 mg, 0.082 mmol). $[\alpha]^{20}_D$ = +15.3 (c. 5.2, CH_2Cl_2). IR (NaCl, ν_{max} / cm^{-1}): 2916 (vs), 2850 (vs), 1732 (vs), 1631 (s), 1605 (s), 1161 (br s). ^1H NMR (400 MHz, $CDCl_3$) δ_H: 7.70-7.62 (m, 3H, H-1,4,8 Nap), 7.40 (dd, *J*=8.6, 1.8 Hz, 1H, H-3 Nap), 7.35-7.27 (m, 5H, Ph-H), 7.13 (dd, *J*=8.9, 2.5 Hz, 1H, H-7 Nap), 7.11 (d, *J*=2.5 Hz, 1H, Nap-5), 5.30-5.16 (m, 1H, CH *sn*-2), 4.51 (AB q, *J*=12.1 Hz, 2H, $PhCH_2$), 4.22 (dd, *J*=11.9, 3.8 Hz, 1H, CH_2 *sn*-1), 4.12 (dd, *J*=11.9, 6.8 Hz, 1H, CH_2 *sn*-1), 3.90 (s, 3H, OCH_3), 3.89 (q, *J*=7.1, 1H, C*H*CH_3), 3.50 (m, 2H, CH_2 *sn*-3), 2.22 (t, *J*=7.5 Hz, 2H, CH_2COO), 1.57 (d, *J*=6.9 Hz, 3H, $CHCH_3$), 1.53-1.51 (m, 2H, CH_2CH_2COO), 1.38-1.30 (m, 24H, CH_2), 0.90 (t, *J*=6.8 Hz, 3H, CH_2CH_3) ppm. $^{13}C\{H\}$ NMR (101 MHz, $CDCl_3$) δ_C: 174.1 (Nap), 173.4 (SFA), 157.8, 137.6, 135.6, 133.8, 129.4, 129.1, 128.5 (2), 127.9, 127.7 (2), 127.2, 126.4, 126.1, 119.1, 105.7, 73.5, 70.7, 68.5, 62.6, 55.4, 45.6, 33.9, 32.1, 29.8 (2), 29.8 (2), 29.8 (2), 29.6, 29.5, 29.4, 29.2, 24.8, 22.8, 18.5, 14.3 ppm. HRMS (ESI) *m*/*z*: $[M + Na]^+$ calcd for $C_{40}H_{56}O_6Na$ 655.3969; found, 655.3962.

*3.4. The removal of the benzyl protective group: Synthesis of (R,S')-**8b-f**, (S,S')-**8b-f**, (R,S')-**9b-f** and (S,S')-**9b-f***

3.4.1. Synthesis of 2-[(*S*)-2-(4-isobutylphenyl)propanoyl]-3-octanoyl-*sn*-glycerol, (*R*,*S*')-**8b**

The same procedure was followed as described for (*R*,*S*')-**8a** using Pd/C catalyst (26 mg), 1-*O*-benzyl-3-octanoyl-2-[(*S*)-2-(4-isobutylphenyl)propanoyl]-*sn*-glycerol (*R*,*S*')-**6b** (116 mg, 0.236 mmol), THF (7 mL) and n-hexane (11.2 mL). Purification on 4% boric acid impregnated flash silica gel chromatography using pet. ether/ethyl acetate (1:1) as eluent, afforded the product (*R*,*S*')-**8b** as a pale-yellow oil, in 86% yield (77 mg, 0.203 mmol). $[\alpha]^{20}_D$ = +18.3 (c. 2.0, CH_2Cl_2). ^1H NMR (400 MHz, $CDCl_3$) δ_H: 7.19 (d, *J*=8.1 Hz, 2H, Ibu-2,6), 7.10 (d, *J*=8.1 Hz, 2H, Ibu-3,5), 5.07-5.03 (m, 1H, CH *sn*-2), 4.29 (dd, *J*=11.9, 4.5 Hz, 1H, CH_2 *sn*-3), 4.20 (dd, *J*=11.9, 5.9 Hz, 1H, CH_2 *sn*-3), 3.73 (q, *J*=7.1 Hz, 1H, C*H*CH_3), 3.60-3.57 (m, 2H, CH_2 *sn*-1), 2.44 (d, *J*=7.2 Hz, 2H, $CH_2CH(CH_3)_2$), 2.29 (t, *J*=7.5 Hz, 2H, CH_2COO), 1.84 (nonet, *J*=6.7 Hz, 1H, $CH(CH_3)_2$), 1.64-1.54 (m, 2H, CH_2CH_2COO), 1.50 (d, *J*=7.2 Hz, 3H, $CHCH_3$), 1.32-1.24 (m, 8H, CH_2), 0.90 (t, *J*=6.1 Hz, 3H, CH_2CH_3), 0.88 (d, *J*=6.6 Hz, 6H, $CH(CH_3)_2$) ppm. $^{13}C\{H\}$ NMR (101 MHz, $CDCl_3$) δ_C: 174.4 (Ibu), 173.8 (SFA), 141.0, 137.8, 129.6 (2), 127.2 (2), 72.7, 62.1, 61.6, 45.3, 45.1, 34.2, 31.8, 30.3, 29.2, 29.1, 25.0, 22.7 (2), 22.5, 18.4, 14.2 ppm. HRMS (ESI) *m*/*z*: $[M + Na]^+$ calcd for $C_{24}H_{38}O_5Na$ 429.2611; found, 429.2608.

3.4.2. Synthesis of 3-decanoyl-2-[(*S*)-2-(4-isobutylphenyl)propanoyl]-*sn*-glycerol, (*R*,*S*')-**8c**

The same procedure was followed as described for (*R*,*S*')-**8a** using Pd/C catalyst (25 mg), 1-*O*-benzyl-3-decanoyl-2-[(*S*)-2-(4-isobutylphenyl)propanoyl]-*sn*-glycerol (*R*,*S*')-**6c** (116 mg, 0.224 mmol), THF (6.4 mL) and n-hexane (10.5 mL). Purification on 4% boric acid impregnated flash silica gel chromatography using pet. ether/ethyl acetate (7:3) as eluent, afforded the product (*R*,*S*')-**8c** as a pale-yellow oil, in 98% yield (94 mg, 0.216 mmol). $[\alpha]^{20}_D$ = +7.50 (c. 0.9, CH_2Cl_2). ^{1}H NMR (400 MHz, $CDCl_3$) δ_H: 7.19 (d, *J*=8.1 Hz, 2H, Ibu-2,6), 7.10 (d, *J*=8.1 Hz, 2H, Ibu-3,5), 5.07-5.03 (m, 1H, CH *sn*-2), 4.29 (dd, *J*=11.9, 4.5 Hz, 1H, CH_2 *sn*-3), 4.21 (dd, *J*=11.9, 5.9 Hz, 1H, CH_2 *sn*-3), 3.73 (q, *J*=7.2 Hz, 1H, C*H*CH_3), 3.61-3.57 (m, 2H, CH_2 *sn*-1), 2.44 (d, *J*=7.1 Hz, 2H, C*H*$_2$CH(CH_3)$_2$), 2.29 (t, *J*=7.5 Hz, 2H, CH_2COO), 1.84 (nonet, *J*=6.8 Hz, 1H, C*H*(CH_3)$_2$), 1.62-1.55 (m, 2H, C*H*$_2$$CH_2$COO), 1.50 (d, *J*=7.2 Hz, 3H, CHC*H*$_3$), 1.33-1.23 (m, 12H, CH_2), 0.90 (t, *J*=6.1 Hz, 3H, CH_2C*H*$_3$), 0.88 (d, *J*=6.5 Hz, 6H, CH(C*H*$_3$)$_2$) ppm. ^{13}C{H} NMR (101 MHz, $CDCl_3$) δ_C: 174.4 (Ibu), 173.8 (SFA), 141.0, 137.8, 129.6 (2), 127.2 (2), 72.7, 62.1, 61.6, 45.3, 45.2, 34.2, 32.0, 30.3, 29.6 (2), 29.4, 29.3, 25.0, 22.8 (2), 22.5, 18.4, 14.3 ppm. HRMS (ESI) *m*/*z*: [M + Na]$^+$ calcd for $C_{26}H_{42}O_5Na$ 457.2924; found, 457.2922.

3.4.3. Synthesis of 3-dodecanoyl-2-[(*S*)-2-(4-isobutylphenyl)propanoyl]-*sn*-glycerol, (*R*,*S*')-**8d**

The same procedure was followed as described for (*R*,*S*')-**8a** using Pd/C catalyst (23 mg), 1-*O*-benzyl-3-dodecanoyl-2-[(*S*)-2-(4-isobutylphenyl)propanoyl]-*sn*-glycerol (*R*,*S*')-**6d** (111 mg, 0.208 mmol), THF (6 mL) and n-hexane (10 mL). Purification on 4% boric acid impregnated flash silica gel chromatography using pet. ether/ethyl acetate (7:3) as eluent, afforded the product (*R*,*S*')-**8d** as a pale-yellow oil, in 92% yield (85 mg, 0.191 mmol). $[\alpha]^{20}_D$ = +2.43 (c. 3.0, CH_2Cl_2). ^{1}H NMR (400 MHz, $CDCl_3$) δ_H: 7.19 (d, *J*=8.1 Hz, 2H, Ibu-2,6), 7.10 (d, *J*=8.1 Hz, 2H, Ibu-3,5), 5.10-5.00 (m, 1H, CH *sn*-2), 4.29 (dd, *J*=11.9, 4.5 Hz, 1H, CH_2 *sn*-3), 4.21 (dd, *J*=11.9, 5.9 Hz, 1H, CH_2 *sn*-3), 3.73 (q, *J*=7.1 Hz, 1H, C*H*CH_3), 3.61-3.57 (m, 2H, CH_2 *sn*-1), 2.44 (d, *J*=7.1 Hz, 2H, C*H*$_2$CH(CH_3)$_2$), 2.37-2.25 (m, 2H, CH_2COO), 1.84 (nonet, *J*=6.8 Hz, 1H, C*H*(CH_3)$_2$), 1.59-1.51 (m, 2H, C*H*$_2$$CH_2$COO), 1.50 (d, *J*=7.2 Hz, 3H, CHC*H*$_3$), 1.33-1.23 (m, 16H, CH_2), 0.90 (t, *J*=6.1 Hz, 3H, CH_2C*H*$_3$), 0.88 (d, *J*=6.7 Hz, 6H, CH(C*H*$_3$)$_2$) ppm. ^{13}C{H} NMR (101 MHz, $CDCl_3$) δ_C: 174.4 (Ibu), 173.8 (SFA), 141.0, 137.8, 129.6 (2), 127.2 (2), 72.7, 62.1, 61.6, 45.3, 45.2, 34.2, 32.1, 30.3, 29.8 (2), 29.6, 29.5, 29.4, 29.3, 25.0, 22.8 (2), 22.5, 18.4, 14.3 ppm. HRMS (ESI) *m*/*z*: [M + Na]$^+$ calcd for $C_{28}H_{46}O_5Na$ 485.3237; found, 485.3237.

3.4.4. Synthesis of 2-[(*S*)-2-(4-isobutylphenyl)propanoyl]-3-tetradecanoyl-*sn*-glycerol, (*R*,*S*')-**8e**

The same procedure was followed as described for (*R*,*S*')-**8a** using Pd/C catalyst (21 mg), 1-*O*-benzyl-2-[(*S*)-2-(4-isobutylphenyl)propanoyl]-3-tetradecanoyl-*sn*-glycerol (*R*,*S*')-**6e** (111 mg, 0.191 mmol), THF (5.5 mL) and n-hexane (9 mL). Purification on 4% boric acid impregnated flash silica gel chromatography using pet. ether/ethyl acetate (7:3) as eluent, afforded the product (*R*,*S*')-**8e** as a pale-yellow oil, in 84% yield (78 mg, 0.160 mmol). $[\alpha]^{20}_D$ = +8.00 (c. 1.0, CH_2Cl_2). ^{1}H NMR (400 MHz, $CDCl_3$) δ_H: 7.19 (d, *J*=8.1 Hz, 2H, Ibu-2,6), 7.10 (d, *J*=8.1 Hz, 2H, Ibu-3,5), 5.08-5.02 (m, 1H, CH *sn*-2), 4.29 (dd, *J*=11.9, 4.5 Hz, 1H, CH_2 *sn*-3), 4.21 (dd, *J*=11.9, 5.9 Hz, 1H, CH_2 *sn*-3), 3.73 (q, *J*=7.1 Hz, 1H, C*H*CH_3), 3.62-3.56 (m, 2H, CH_2 *sn*-1), 2.44 (d, *J*=7.2 Hz, 2H, C*H*$_2$CH(CH_3)$_2$), 2.29 (t, *J*=7.5 Hz, 2H, CH_2COO), 1.84 (nonet, *J*=6.7 Hz, 1H, C*H*(CH_3)$_2$), 1.63-1.57 (m, 2H, C*H*$_2$$CH_2$COO), 1.50 (d, *J*=7.1 Hz, 3H, CHC*H*$_3$), 1.29-1.23 (m, 20H, CH_2), 0.90 (t, *J*=6.1 Hz, 3H, CH_2C*H*$_3$), 0.88 (d, *J*=6.7 Hz, 6H, CH(C*H*$_3$)$_2$) ppm. ^{13}C{H} NMR (101 MHz, $CDCl_3$) δ_C: 174.4 (Ibu), 173.8 (SFA), 141.0, 137.8, 129.6 (2), 127.2 (2), 72.7, 62.1, 61.6, 45.3, 45.2, 34.3, 32.1, 30.3, 29.8 (2), 29.8, 29.8, 29.6, 29.5, 29.4, 29.3, 25.0, 22.9 (2), 22.5, 18.4, 14.3 ppm. HRMS (ESI) *m*/*z*: [M + Na]$^+$ calcd for $C_{30}H_{50}O_5Na$ 513.3550; found, 513.3550.

3.4.5. Synthesis of 3-hexadecanoyl-2-[(*S*)-2-(4-isobutylphenyl)propanoyl]-*sn*-glycerol, (*R*,*S*')-**8f**

The same procedure was followed as described for (*R*,*S*')-**8a** using Pd/C catalyst (25 mg), 1-*O*-benzyl-3-hexadecanoyl-2-[(*S*)-2-(4-isobutylphenyl)propanoyl]-*sn*-glycerol (*R*,*S*')-**6f** (135 mg, 0.222 mmol), THF (6.4 mL) and n-hexane (10.5 mL). Purification on 4% boric acid impregnated flash silica gel

chromatography using pet. ether/ethyl acetate (7:3) as eluent, afforded the product (*R*,*S*')-**8f** as a pale-yellow oil, in 90% yield (104 mg, 0.200 mmol). $[\alpha]^{20}_{D}$ = +0.75 (c. 2.0, CH_2Cl_2). ^1H NMR (400 MHz, $CDCl_3$) δ_H: 7.19 (d, *J*=8.1 Hz, 2H, Ibu-2,6), 7.10 (d, *J*=8.1 Hz, 2H, Ibu-3,5), 5.07-5.03 (m, 1H, CH *sn*-2), 4.29 (dd, *J*=11.9, 4.5 Hz, 1H, CH_2 *sn*-3), 4.21 (dd, *J*=11.9, 5.9 Hz, 1H, CH_2 *sn*-3), 3.73 (q, *J*=7.1 Hz, 1H, C*H*CH_3), 3.61-3.57 (m, 2H, CH_2 *sn*-1), 2.44 (d, *J*=7.2 Hz, 2H, C*H*$_2$CH(CH_3)$_2$), 2.29 (t, *J*=7.5 Hz, 2H, CH_2COO), 1.84 (nonet, *J*=6.8 Hz, 1H, C*H*(CH_3)$_2$), 1.62-1.56 (m, 2H, C*H*$_2$$CH_2$COO), 1.50 (d, *J*=7.2 Hz, 3H, CHC*H*$_3$), 1.29-1.23 (m, 24H, CH_2), 0.90 (t, *J*=6.1 Hz, 3H, CH_2C*H*$_3$), 0.88 (d, *J*=6.6 Hz, 6H, CH(C*H*$_3$)$_2$) ppm. $^{13}C\{H\}$ NMR (101 MHz, $CDCl_3$) δ_C: 174.4 (Ibu), 173.8 (SFA), 141.0, 137.8, 129.6 (2), 127.2 (2), 72.7, 62.1, 61.6, 45.3, 45.2, 34.2, 32.1, 30.3, 29.8 (2), 29.8 (2), 29.8 (2), 29.6, 29.5, 29.4, 29.3, 25.0, 22.8 (2), 22.5, 18.4, 14.3 ppm. HRMS (ESI) *m*/*z*: $[M + Na]^+$ calcd for $C_{32}H_{54}O_5Na$ 541.3863; found, 541.3857.

3.4.6. Synthesis of 2-[(*S*)-2-(4-isobutylphenyl)propanoyl]-1-octanoyl-*sn*-glycerol, (*S*,*S*')-**8b**

The same procedure was followed as described for (*R*,*S*')-**8a** using Pd/C catalyst (10 mg), 3-*O*-benzyl-1-octanoyl-2-[(*S*)-2-(4-isobutylphenyl)propanoyl]-*sn*-glycerol (*S*,*S*')-**6b** (48 mg, 0.097 mmol), THF (3 mL) and n-hexane (4.6 mL). Purification on 4% boric acid impregnated flash silica gel chromatography using pet. ether/ethyl acetate (7:3) as eluent, afforded the product (*S*,*S*')-**8b** as a pale-yellow oil, in 86% yield (77 mg, 0.203 mmol). $[\alpha]^{20}_{D}$ = +18.8 (c. 1.0, CH_2Cl_2). IR (NaCl, ν_{max} / cm^{-1}): 3478 (br), 2928 (vs), 2870 (vs), 1739 (vs). ^1H NMR (400 MHz, $CDCl_3$) δ_H: 7.19 (d, *J*=8.1 Hz, 2H, Ibu-2,6), 7.09 (d, *J*=8.1 Hz, 2H, Ibu-3,5), 5.07-5.02 (m, 1H, CH *sn*-2), 4.21 (dd, *J*=11.9, 4.6 Hz, 1H, CH_2 *sn*-1), 4.20 (dd, *J*=11.9, 6.0 Hz, 1H, CH_2 *sn*-1), 3.77-3.68 (m, 2H, CH_2 *sn*-3 and 1H, C*H*CH_3), 2.44 (d, *J*=7.2 Hz, 2H, C*H*$_2$CH(CH_3)$_2$), 2.18 (t, *J*=7.6 Hz, 2H, CH_2COO), 1.98-1.92 (bs, 1H, OH), 1.84 (nonet, *J*=6.7 Hz, 1H, C*H*(CH_3)$_2$), 1.58-1.49 (m, 2H, C*H*$_2$$CH_2$COO), 1.50 (d, *J*=7.2 Hz, 3H, CHC*H*$_3$), 1.33-1.23 (m, 8H, CH_2), 0.90 (t, *J*=6.1 Hz, 3H, CH_2C*H*$_3$), 0.88 (d, *J*=6.6 Hz, 6H, CH(C*H*$_3$)$_2$) ppm. $^{13}C\{H\}$ NMR (101 MHz, $CDCl_3$) δ_C: 174.3 (Ibu), 173.6 (SFA), 140.7, 137.3, 129.3 (2), 127.0 (C-1,3 Ibu), 72.5, 61.8, 61.6, 45.1, 45.0, 33.9, 31.77, 30.2, 29.1, 28.9,), 24.8, 22.6 (2), 22.4, 18.3, 14.0 ppm. HRMS (ESI) *m*/*z*: $[M + Na]^+$ calcd for $C_{24}H_{38}O_5Na$ 429.2611; found, 429.2604.

3.4.7. Synthesis of 1-decanoyl-2-[(*S*)-2-(4-isobutylphenyl)propanoyl]-*sn*-glycerol, (*S*,*S*')-**8c**

The same procedure was followed as described for (*R*,*S*')-**8a** using Pd/C catalyst (27 mg), 3-*O*-benzyl-1-decanoyl-2-[(*S*)-2-(4-isobutylphenyl)propanoyl]-*sn*-glycerol (*S*,*S*')-**6c** (150 mg, 0.285 mmol), THF (9 mL) and n-hexane (12 mL). Purification on 4% boric acid impregnated flash silica gel chromatography using pet. ether/ethyl acetate (7:3) as eluent, afforded the product (*S*,*S*')-**8c** as a pale-yellow oil, in 89% yield (110 mg, 0.253 mmol). $[\alpha]^{20}_{D}$ = +20.2 (c. 1.5, CH_2Cl_2). IR (NaCl, ν_{max} / cm^{-1}): 3486 (br), 2955 (vs), 2926 (vs), 2855 (vs), 1739 (vs), 1162 (br s). ^1H NMR (400 MHz, $CDCl_3$) δ_H: 7.19 (d, *J*=8.1 Hz, 2H, Ibu-2,6), 7.10 (d, *J*=8.1 Hz, 2H, Ibu-3,5), 5.09-5.03 (m, 1H, CH *sn*-2), 4.21 (dd, *J*=11.9, 4.6 Hz, 1H, CH_2 *sn*-1), 4.14 (dd, *J*=11.9, 6.0 Hz, 1H, CH_2 *sn*-1), 3.77-3.68 (m, 2H, CH_2 *sn*-3 and 1H, C*H*CH_3), 2.44 (d, *J*=7.2 Hz, 2H, C*H*$_2$CH(CH_3)$_2$), 2.20-2.15 (m, 2H, CH_2COO), 1.84 (nonet, *J*=6.7 Hz, 1H, C*H*(CH_3)$_2$), 1.63 (bs, 1H, OH), 1.58-1.49 (m, 2H, C*H*$_2$$CH_2$COO and 3H, CHC*H*$_3$), 1.34-1.27 (m, 12H, CH_2), 0.90 (t, *J*=6.1 Hz, 3H, CH_2C*H*$_3$), 0.88 (d, *J*=6.6 Hz, 6H, CH(C*H*$_3$)$_2$) ppm. $^{13}C\{H\}$ NMR (101 MHz, $CDCl_3$) δ_C: 174.5 (Ibu), 173.8 (SFA), 140.8, 137.5, 129.6 (2), 129.5 (2), 72.7, 62.0, 61.7, 45.3, 45.2, 34.1, 32.0, 30.3, 29.8, 29.6, 29.4, 293, 24.9, 22.5 (2), 22.5, 18.4, 14.2 ppm. HRMS (ESI) *m*/*z*: $[M + Na]^+$ calcd for $C_{26}H_{42}O_5Na$ 457.2924; found, 457.2923.

3.4.8. Synthesis of 1-dodecanoyl-2-[(*S*)-2-(4-isobutylphenyl)propanoyl]-*sn*-glycerol, (*S*,*S*')-**8d**

The same procedure was followed as described for (*R*,*S*')-**8a** using Pd/C catalyst (8 mg), 3-*O*-benzyl-1-dodecanoyl-2-[(*S*)-2-(4-isobutylphenyl)propanoyl]-*sn*-glycerol (*S*,*S*')-**6d** (50 mg, 0.090 mmol), THF (3.5 mL) and n-hexane (4.5 mL). Purification on 4% boric acid impregnated flash silica gel chromatography using pet. ether/ethyl acetate (7:3) as eluent, afforded the product (*S*,*S*')-**8d** as a pale-yellow oil, in 93% yield (40 mg, 0.082 mmol). $[\alpha]^{20}_{D}$ = +13.8 (c. 2.0, CH_2Cl_2). IR (NaCl, ν_{max} / cm^{-1}): 3469 (br), 2956 (vs), 2926 (vs), 2855

(vs), 1736 (vs), 1513 (s), 1165 (br s). ^{1}H NMR (400 MHz, $CDCl_3$) δ_H: 7.19 (d, *J*=8.1 Hz, 2H, Ibu-2,6), 7.10 (d, *J*=8.1 Hz, 2H, Ibu-3,5), 5.07-5.02 (m, 1H, CH *sn*-2), 4.21 (dd, *J*=11.9, 4.6 Hz, 1H, CH_2 *sn*-1), 4.21 (dd, *J*=11.9, 6.0 Hz, 1H, CH_2 *sn*-1), 3.78-3.68 (m, 2H, CH_2 *sn*-3 and 1H, C*H*CH_3), 2.44 (d, *J*=7.1 Hz, 2H, C*H*$_2$CH(CH_3)$_2$), 2.18 (t, *J*=7.6 Hz, 2H, CH_2COO), 1.91 (s, 1H, OH), 1.84 (nonet, *J*=6.7 Hz, 1H, C*H*(CH_3)$_2$), 1.59-1.50 (m, 2H, C*H*$_2$$CH_2$COO), 1.50 (d, *J*=7.2 Hz, 3H, CHC*H*$_3$), 1.34-1.27 (m, 16H, CH_2), 0.90 (t, *J*=6.1 Hz, 3H, CH_2C*H*$_3$), 0.88 (d, *J*=6.6 Hz, 6H, CH(C*H*$_3$)$_2$) ppm. ^{13}C{H} NMR (101 MHz, $CDCl_3$) δ_C: 174.5 (Ibu), 173.8 (SFA), 140.8, 137.4, 129.5 (2), 127.2 (2), 72.7, 62.0, 61.7, 45.3, 45.2, 34.1, 32.32, 30.3, 29.8 (2), 29.6, 29.5, 29.4, 29.3, 24.9, 22.8 (2), 22.5, 18.4, 14.3 ppm. HRMS (ESI) *m*/*z*: [M + Na]$^+$ calcd for $C_{28}H_{46}O_5Na$ 485.3237; found, 485.3226.

3.4.9. Synthesis of 2-[(*S*)-2-(4-isobutylphenyl)propanoyl]-1-tetradecanoyl-*sn*-glycerol, (*S,S'*)-**8e**

The same procedure was followed as described for (*R,S'*)-**8a** using Pd/C catalyst (14 mg), 3-*O*-benzyl-2-[(*S*)-2-(4-isobutylphenyl)propanoyl]-3-tetradecanoyl-*sn*-glycerol (*S,S'*)-**6e** (69 mg, 0.119 mmol), THF (4.5 mL) and n-hexane (6 mL). Purification on 4% boric acid impregnated flash silica gel chromatography using pet. ether/ethyl acetate (3:2) as eluent, afforded the product (*S,S'*)-**8e** as a pale-yellow oil, in 94% yield (55 mg, 0.112 mmol). $[\alpha]^{20}_D$ = +4.20 (c. 4.9, CH_2Cl_2). IR (NaCl, ν_{max} / cm^{-1}): 3461 (br), 2955 (vs), 2925 (vs), 2854 (vs), 1740 (vs), 1512 (s), 1164 (br s). ^{1}H NMR (400 MHz, $CDCl_3$) δ_H: 7.19 (d, *J*=8.1 Hz, 2H, Ibu-2,6), 7.10 (d, *J*=8.1 Hz, 2H, Ibu-3,5), 5.11-5.01 (m, 1H, CH *sn*-2), 4.29 (dd, *J*=11.9, 4.6 Hz, 1H, CH_2 *sn*-1), 4.21 (dd, *J*=11.9, 6.0 Hz, 1H, CH_2 *sn*-1), 3.79-3.66 (m, 2H, CH_2 *sn*-3 and 1H, C*H*CH_3), 2.44 (d, *J*=7.2 Hz, 2H, C*H*$_2$CH(CH_3)$_2$), 2.17 (t, *J*=7.6 Hz, 2H, CH_2COO), 1.92 (s, 1H, OH), 1.84 (nonet, *J*=6.8 Hz, 1H, C*H*(CH_3)$_2$), 1.59-1.50 (m, 2H, C*H*$_2$$CH_2$COO), 1.50 (d, *J*=7.2 Hz, 3H, CHC*H*$_3$), 1.34-1.27 (m, 20H, CH_2), 0.90 (t, *J*=6.1 Hz, 3H, CH_2C*H*$_3$), 0.88 (d, *J*=6.6 Hz, 6H, CH(C*H*$_3$)$_2$) ppm. ^{13}C{H} NMR (101 MHz, $CDCl_3$) δ_C: 174.5 (Ibu), 173.8 (SFA), 140.8, 137.4, 129.5 (2), 127.2 (2), 72.7, 62.0, 61.7, 45.3, 45.2, 34.1, 32.1, 30.3, 29.8 (2), 29.8, 29.8, 29.6, 29.5, 29.4, 29.3, 24.9, 22.8 (2), 22.5, 18.4, 14.3 ppm. HRMS (ESI) *m*/*z*: [M + Na]$^+$ calcd for $C_{30}H_{50}O_5Na$ 513.3550; found, 513.3546.

3.4.10. Synthesis of 1-hexadecanoyl-2-[(*S*)-2-(4-isobutylphenyl)propanoyl]-*sn*-glycerol, (*S,S'*)-**8f**

The same procedure was followed as described for (*R,S'*)-**8a** using Pd/C catalyst (4.6 mg), 3-*O*-benzyl-1-hexadecanoyl-2-[(*S*)-2-(4-isobutylphenyl)propanoyl]-*sn*-glycerol (*S,S'*)-**6f** (25 mg, 0.041 mmol), THF (1.6 mL) and n-hexane (2.3 mL). Purification on 4% boric acid impregnated flash silica gel chromatography using pet. ether/ethyl acetate (3:2) as eluent, afforded the product (*S,S'*)-**8f** as a pale-yellow oil, in 90% yield (19 mg, 0.037 mmol). $[\alpha]^{20}_D$ = +15.0 (c. 1.9, CH_2Cl_2). IR (NaCl, ν_{max} / cm^{-1}): 3378 (br), 2925 (vs), 2855 (vs), 1740 (vs), 1512 (s), 1162 (br s). ^{1}H NMR (400 MHz, $CDCl_3$) δ_H: 7.19 (d, *J*=8.1 Hz, 2H, Ibu-2,6), 7.10 (d, *J*=8.1 Hz, 2H, Ibu-3,5), 5.06-5.02 (m, 1H, CH *sn*-2), 4.21 (dd, *J*=11.9, 4.5 Hz, 1H, CH_2 *sn*-1), 4.13 (dd, *J*=11.9, 6.0 Hz, 1H, CH_2 *sn*-1), 3.75-3.70 (m, 2H, CH_2 *sn*-3 and 1H, C*H*CH_3), 2.44 (d, *J*=7.2 Hz, 2H, C*H*$_2$CH(CH_3)$_2$), 2.20-2.14 (m, 2H, CH_2COO), 1.84 (nonet, *J*=6.8 Hz, 1H, C*H*(CH_3)$_2$), 1.67-1.55 (m, 2H, C*H*$_2$$CH_2$COO), 1.61 (s, 1H, OH), 1.50 (d, *J*=7.2 Hz, 3H, CHC*H*$_3$), 1.34-1.27 (m, 24H, CH_2), 0.90 (t, *J*=6.1 Hz, 3H, CH_2C*H*$_3$), 0.88 (d, *J*=6.6 Hz, 6H, CH(C*H*$_3$)$_2$) ppm. ^{13}C{H} NMR (101 MHz, $CDCl_3$) δ_C: 174.5 (Ibu), 173.8 (SFA), 140.8, 137.4, 129.5 (2), 127.2 (2), 72.7, 62.0, 61.7, 45.2, 45.2, 34.1, 32.1, 30.3, 29.8 (2), 29.8 (2), 29.8 (2), 29.6, 29.5, 29.4, 29.27, 24.9, 22.8 (2), 22.5, 18.4, 14.3 ppm. HRMS (ESI) *m*/*z*: [M + Na]$^+$ calcd for $C_{32}H_{54}O_5Na$ 541.3863; found, 541.3860.

3.4.11. Synthesis of 2-[(*S*)-2-(6-methoxynaphthalen-2-yl)propanoyl]-3-octanoyl-*sn*-glycerol, (*R,S'*)-**9b**

The same procedure was followed as described for (*R,S'*)-**9a** using Pd/C catalyst (31 mg), 1-*O*-benzyl-2-[(*S*)-2-(6-methoxynaphthalen-2-yl)propanoyl]-3-octanoyl-*sn*-glycerol (*S,S'*)-**7b** (145 mg, 0.278 mmol), THF (8 mL) and n-hexane (13 mL). Purification on 4% boric acid impregnated flash silica gel chromatography using pet. ether/ethyl acetate (1:1) as eluent, afforded the product (*R,S'*)-**9b** as a pale-yellow oil, in 94% yield (112 mg, 0.260 mmol). $[\alpha]^{20}_D$ = +7.17 (c. 3.0, CH_2Cl_2). ^{1}H NMR (400 MHz, $CDCl_3$) δ_H:

7.75-7.63 (m, 3H, Nap-1,4,8), 7.39 (dd, *J*=8.5, 1.9 Hz, 1H, Nap-3), 7.14 (dd, *J*=8.9, 2.5 Hz, 1H, Nap-7), 7.11 (d, *J*=2.5 Hz, 1H, Nap-5), 5.13-5.01 (m, 1H, CH *sn*-2), 4.31 (dd, *J*=11.9, 4.4 Hz, 1H, CH_2 *sn*-3), 4.21 (dd, *J*=11.9, 5.9 Hz, 1H, CH_2 *sn*-3), 3.91 (s, 3H, OCH_3), 3.91-3.80 (m, 1H, C*H*CH_3), 3.60-3.56 (m, 2H, CH_2 *sn*-1), 2.25 (t, *J*=7,2 Hz, 2H, CH_2COO), 1.68 (t, *J*=6.6 Hz, 1H, OH), 1.58 (d, *J*=7.2 Hz, 3H, CHC*H*$_3$), 1.56 (quint, *J*=7.2 Hz, 2H, C*H*$_2$$CH_2COO$), 1.38-1.23 (m, 8H, CH_2), 0.89 (t, *J*=7.0 Hz, 3H, CH_2C*H*$_3$) ppm. ^{13}C{H} NMR (101 MHz, $CDCl_3$) δ_C: 174.3 (Nap), 173.8 (SFA), 157.9, 135.6, 133.9, 129.4, 129.1, 127.4, 126.1, 126.0, 119.3, 105.8, 72.8, 62.1, 61.5, 55.5, 45.6, 34.2, 31.8, 29.2, 29.0, 25.0, 22.7, 18.5, 14.2 ppm. HRMS (ESI) *m/z*: [M + Na]$^+$ calcd for $C_{25}H_{34}O_6Na$ 453.2248; found, 453.2248.

3.4.12. Synthesis of 3-decanoyl-2-[(*S*)-2-(6-methoxynaphthalen-2-yl)propanoyl]-*sn*-glycerol, (*R,S*')-**9c**

The same procedure was followed as described for (*R,S*')-**9a** using Pd/C catalyst (22 mg), 1-*O*-benzyl-3-decanoyl-2-[(*S*)-2-(6-methoxynaphthalen-2-yl)propanoyl]-*sn*-glycerol, (*R,S*')-**7c** (107 mg, 0.195 mmol), THF (5.6 mL) and n-hexane (9.2 mL). Purification on 4% boric acid impregnated flash silica gel chromatography using pet. ether/ethyl acetate (1:1) as eluent, followed by recrystallization from n-hexane afforded the product (*R,S*')-**9c** as very fine white needles, in 84% yield (75 mg, 0.164 mmol). Mp. 64.7-65.3°C. $[\alpha]^{20}_D$ = +5.70 (c. 2.0, CH_2Cl_2). ^{1}H NMR (400 MHz, $CDCl_3$) δ_H: 7.74-7.64 (m, 3H, Nap-1,4,8), 7.39 (dd, *J*=8.5, 1.9 Hz, 1H, Nap-3), 7.15 (dd, *J*=8.9, 2.5 Hz, 1H, Nap-7), 7.11 (d, *J*=2.5 Hz, 1H, Nap-5), 5.13-5.03 (m, 1H, CH *sn*-2), 4.31 (dd, *J*=11.9, 4.4 Hz, 1H, CH_2 *sn*-3), 4.22 (dd, *J*=11.9, 5.9 Hz, 1H, CH_2 *sn*-3), 3.91 (s, 3H, OCH_3), 3.89 ((q, *J*=7.2 Hz, C*H*CH_3), 3.62-3.56 (m, 2H, CH_2 *sn*-1), 2.26 (m, 2H, CH_2COO and 3H, CHC*H*$_3$), 1.66-1.52 (m, 2H, C*H*$_2$$CH_2COO$), 1.30-1.22 (m, 12H, CH_2), 0.89 (t, *J*=7.0 Hz, 3H, CH_2C*H*$_3$) ppm. ^{13}C{H} NMR (101 MHz, $CDCl_3$) δ_C: 174.3 (Nap), 173.8 (SFA), 157.9, 135.6, 133.9, 129.4, 129.1, 127.5, 126.1, 126.0, 119.3, 105.8, 72.8, 62.1, 61.6, 55.5, 45.6, 34.2, 32.0, 29.6, 29.4, 29.4, 29.3, 25.0, 22.8, 18.5, 14.3 ppm. HRMS (ESI) *m/z*: [M + Na]$^+$ calcd for $C_{27}H_{38}O_6Na$ 481.2561; found, 481.2565.

3.4.13. Synthesis of 3-dodecanoyl-2-[(*S*)-2-(6-methoxynaphthalen-2-yl)propanoyl]-*sn*-glycerol, (*R,S*')-**9d**

The same procedure was followed as described for (*R,S*')-**9a** using Pd/C catalyst (21 mg), 1-*O*-benzyl-3-dodecanoyl-2-[(*S*)-2-(6-methoxynaphthalen-2-yl)propanoyl]-*sn*-glycerol, (*R,S*')-**7d** (103 mg, 0.184 mmol), THF (5.3 mL) and n-hexane (8.7 mL). Purification on 4% boric acid impregnated flash silica gel chromatography using pet. ether/ethyl acetate (1:1) as eluent, followed by recrystallization from n-hexane afforded the product (*R,S*')-**9d** as very fine white needles, in 94% yield (81 mg, 0.173 mmol). Mp. 68.4-68.7°C. $[\alpha]^{20}_D$ = +3.08 (c. 2.6, CH_2Cl_2). ^{1}H NMR (400 MHz, $CDCl_3$) δ_H: 7.75-7.65 (m, 3H, Nap-1,4,8), 7.39 (dd, *J*=8.5, 1.9 Hz, 1H, Nap-3), 7.15 (dd, *J*=8.9, 2.5 Hz, 1H, Nap-7), 7.11 (d, *J*=2.5 Hz, 1H, Nap-5), 5.13-5.03 (m, 1H, CH *sn*-2), 4.31 (dd, *J*=11.9, 4.4 Hz, 1H, CH_2 *sn*-3), 4.22 (dd, *J*=11.9, 5.9 Hz, 1H, CH_2 *sn*-3), 3.91 (s, 3H, OCH_3), 3.89 ((q, *J*=7.2 Hz, C*H*CH_3), 3.62-3.56 (m, 2H, CH_2 *sn*-1), 2.26 (m, 2H, CH_2COO and 3H, CHC*H*$_3$), 1.65-1.51 (m, 2H, C*H*$_2$$CH_2COO$), 1.30-1.22 (m, 16H, CH_2), 0.89 (t, *J*=7.0 Hz, 3H, CH_2C*H*$_3$) ppm. ^{13}C{H} NMR (101 MHz, $CDCl_3$) δ_C: 174.3 (Nap), 173.8 (SFA), 157.9, 135.6, 133.9, 129.4, 129.1, 127.5, 126.1, 126.0, 119.3, 105.8, 72.8, 62.1, 61.6, 55.5, 45.6, 34.2, 32.1, 29.8 (2), 29.6, 29.5, 29.4, 29.3, 25.0, 22.8, 18.5, 14.3 ppm. HRMS (ESI) *m/z*: [M + Na]$^+$ calcd for $C_{29}H_{42}O_6Na$ 509.2874; found, 509.2867.

3.4.14. Synthesis of 2-[(*S*)-2-(6-methoxynaphthalen-2-yl)propanoyl]-3-tetradecanoyl-*sn*-glycerol, (*R,S*')-**9e**

The same procedure was followed as described for (*R,S*')-**9a** using Pd/C catalyst (20 mg), 1-*O*-benzyl-2-[(*S*)-2-(6-methoxynaphthalen-2-yl)propanoyl]-3-tetradecanoyl-*sn*-glycerol, (*R,S*')-**7e** (106 mg, 0.175 mmol), THF (5 mL) and n-hexane (8.3 mL). Purification on 4% boric acid impregnated flash silica gel chromatography using pet. ether/ethyl acetate (1:1) as eluent, followed by recrystallization from n-hexane afforded the product (*R,S*')-**9e** as very fine white needles, in 89% yield (80 mg, 0.155 mmol). Mp. 64.9-65.2°C. $[\alpha]^{20}_D$ = +2.04 (c. 2.5, CH_2Cl_2). ^{1}H NMR (400 MHz, $CDCl_3$) δ_H: 7.72-7.65 (m, 3H, Nap-1,4,8), 7.39 (dd, *J*=8.5, 1.9 Hz, 1H, Nap-3), 7.15 (dd, *J*=8.9, 2.5 Hz, 1H, Nap-7), 7.11 (d, *J*=2.5 Hz, 1H, Nap-5), 5.13-5.03 (m, 1H, CH *sn*-

2), 4.31 (dd, J=12.0, 4.4 Hz, 1H, CH_2 *sn*-3), 4.22 (dd, J=12.0, 5.9 Hz, 1H, CH_2 *sn*-3), 3.91 (s, 3H, OCH_3), 3.89 ((q, J=7.2 Hz, C*H*CH_3), 3.63-3.55 (m, 2H, CH_2 *sn*-1), 2.26 (m, 2H, CH_2COO and 3H, CHCH_3), 1.62-1.52 (m, 2H, C$H_2$$CH_2COO$), 1.30-1.22 (m, 20H, CH_2), 0.88 (t, J=6.8 Hz, 3H, CH_2CH_3) ppm. ^{13}C{H} NMR (101 MHz, $CDCl_3$) δ_C: 174.3 (Nap), 173.8 (SFA), 157.9, 135.6, 133.9, 129.4, 129.1, 127.5, 126.1, 126.1, 119.3, 105.8, 72.9, 62.1, 61.6, 55.5, 45.6, 34.2, 32.1, 29.9 (2), 29.8, 29.8, 29.6, 29.5, 29.4, 29.3, 25.0, 22.8, 18.5, 14.3 ppm. HRMS (ESI) *m*/*z*: [M + Na]$^+$ calcd for $C_{31}H_{46}O_6Na$ 537.3187; found, 537.3181.

3.4.15. Synthesis of 3-hexadecanoyl-2-[(*S*)-2-(6-methoxynaphthalen-2-yl)propanoyl]-*sn*-glycerol, (*R,S*')-**9f**

The same procedure was followed as described for (*R,S*')-**9a** using Pd/C catalyst (20 mg), 1-*O*-benzyl-3-hexadecanoyl-2-[(*S*)-2-(6-methoxynaphthalen-2-yl)propanoyl]-*sn*-glycerol, (*R,S*')-**7f** (110 mg, 0.174 mmol), THF (5 mL) and n-hexane (8.3 mL). Purification on 4% boric acid impregnated flash silica gel chromatography using pet. ether/ethyl acetate (1:1) as eluent, followed by recrystallization from n-hexane afforded the product (*R,S*')-**9f** as very fine white needles, in 91% yield (86 mg, 0.158 mmol). Mp. 73.6-74.9°C. $[\alpha]^{20}_D$ = +4.95 (c. 2.0, CH_2Cl_2). ^{1}H NMR (400 MHz, $CDCl_3$) δ_H: 7.79-7.58 (m, 3H, Nap-1,4,8), 7.39 (dd, J=8.5, 1.9 Hz, 1H, Nap-3), 7.15 (dd, J=8.9, 2.5 Hz, 1H, Nap-7), 7.11 (d, J=2.5 Hz, 1H, Nap-5), 5.13-5.03 (m, 1H, CH *sn*-2), 4.31 (dd, J=12.0, 4.4 Hz, 1H, CH_2 *sn*-3), 4.22 (dd, J=12.0, 5.9 Hz, 1H, CH_2 *sn*-3), 3.91 (s, 3H, OCH_3), 3.89 ((q, J=7.2 Hz, C*H*CH_3), 3.63-3.55 (m, 2H, CH_2 *sn*-1), 2.26 (m, 2H, CH_2COO and 3H, CHCH_3), 1.62-1.52 (m, 2H, C$H_2$$CH_2COO$), 1.30-1.22 (m, 24H, CH_2), 0.88 (t, J=6.8 Hz, 3H, CH_2CH_3) ppm. ^{13}C{H} NMR (101 MHz, $CDCl_3$) δ_C: 174.3 (Nap), 173.8 (SFA), 157.9, 135.6, 133.9, 129.4, 129.1, 127.5, 126.0, 126.0, 119.3, 105.8, 72.8, 62.1, 61.6, 55.5, 45.6, 34.2, 32.1, 29.9 (2), 29.8 (2), 29.8 (2), 29.6, 29.5, 29.4, 29.3, 25.0, 22.9, 18.5, 14.3 ppm. HRMS (ESI) *m*/*z*: [M + Na]$^+$ calcd for $C_{33}H_{50}O_6Na$ 565.3500; found, 565.3500.

3.4.16. Synthesis of 2-[(*S*)-2-(6-methoxynaphthalen-2-yl)propanoyl]-1-octanoyl-*sn*-glycerol, (*S,S*')-**9b**

The same procedure was followed as described for (*R,S*')-**9a** using Pd/C catalyst (10 mg), 3-*O*-benzyl-2-[(*S*)-2-(6-methoxynaphthalen-2-yl)propanoyl]-1-octanoyl-*sn*-glycerol (*S,S*')-**7b** (50 mg, 0.096 mmol), THF (3 mL) and n-hexane (4.6 mL). Purification on 4% boric acid impregnated flash silica gel chromatography using pet. ether/ethyl acetate (1:1) as eluent, afforded the product (*S,S*')-**9b** as a pale-yellow oil, in 97% yield (40 mg, 0.093 mmol). $[\alpha]^{20}_D$ = +18.0 (c. 3.4, CH_2Cl_2). IR (NaCl, ν_{max} / cm^{-1}): 3497 (br), 2930 (vs), 2856 (vs), 1736 (vs), 1634 (cs), 1607 (vs). 1606 (vs). ^{1}H NMR (400 MHz, $CDCl_3$) δ_H: 7.77-7.62 (m, 3H, Nap-1,4,8), 7.39 (dd, J=8.5, 1.9 Hz, 1H, Nap-3), 7.14 (dd, J=8.9, 2.5 Hz, 1H, Nap-7), 7.10 (d, J=2.5 Hz, 1H, Nap-5), 5.14-5.01 (m, 1H, CH *sn*-2), 4.19 (dd, J=11.9, 4.4 Hz, 1H, CH_2 *sn*-1), 4.13 (dd, J=11.9, 6.1 Hz, 1H, CH_2 *sn*-1), 3.91 (s, 3H, OCH_3), 3.72 (q, J=7.1 Hz, 1H, C*H*CH_3), 3.60-3.56 (m, 2H, CH_2 *sn*-1), 2.05-1.90 (t, J=7,2 Hz, 2H, CH_2COO), 1.59 (d, J=7.1 Hz, 3H, CHCH_3), 1.39 (quint, J=7.5 Hz, 2H, C$H_2$$CH_2COO$), 1.34-1.08 (m, 8H, CH_2), 0.86 (t, J=6.8 Hz, 3H, CH_2CH_3) ppm. ^{13}C{H} NMR (101 MHz, $CDCl_3$) δ_C: 174.4 (Nap), 173.7 (SFA), 157.9, 135.4, 133.9, 129.4, 129.1, 127.3, 126.2, 126.1, 119.2, 105.7, 72.8, 61.9, 61.7, 55.5, 45.6, 34.0, 32.0, 29.4, 29.2, 24.8, 22.8, 18.5, 14.3 ppm. HRMS (ESI) *m*/*z*: [M + Na]$^+$ calcd for $C_{25}H_{34}O_6Na$ 453.2248; found, 453.2253.

3.4.17. Synthesis of 1-decanoyl-2-[(*S*)-2-(6-methoxynaphthalen-2-yl)propanoyl]-*sn*-glycerol, (*S,S*')-**9c**

The same procedure was followed as described for (*R,S*')-**9a** using Pd/C catalyst (21 mg), 3-*O*-benzyl-1-decanoyl-2-[(*S*)-2-(6-methoxynaphthalen-2-yl)propanoyl]-*sn*-glycerol, (*S,S*')-**7c** (100 mg, 0.181 mmol), THF (7 mL) and n-hexane (9.2 mL). Purification on 4% boric acid impregnated flash silica gel chromatography using pet. ether/ethyl acetate (1:1) as eluent, afforded the product (*S,S*')-**9c** as a pale-yellow oil in 92% yield (75 mg, 0.164 mmol). $[\alpha]^{20}_D$ = +8.56 (c. 0.9, CH_2Cl_2). IR (NaCl, ν_{max} / cm^{-1}): 3363 (br), 2973 (vs), 2927 (vs), 1739 (vs), 1634 (s), 1607 (s), 1177 (br s). ^{1}H NMR (400 MHz, $CDCl_3$) δ_H: 7.77-7.62 (m, 3H, Nap-1,4,8), 7.39 (dd, J=8.5, 1.9 Hz, 1H, Nap-3), 7.14 (dd, J=8.9, 2.5 Hz, 1H, Nap-7), 7.10 (d, J=2.5 Hz, 1H, Nap-5), 5.14-5.01 (m, 1H, CH *sn*-2), 4.19 (dd, J=11.9, 4.4 Hz, 1H, CH_2 *sn*-1), 4.13 (dd, J=11.9, 6.1 Hz, 1H, CH_2 *sn*-1), 3.91 (s, 3H, OCH_3), 3.72 ((q, J=7.0 Hz, C*H*CH_3), 3.62-3.56 (m, 2H, CH_2 *sn*-3), 2.05-1.90 (m, 2H, CH_2COO), 1.59

(d, *J*=7.1 Hz, 3H, CHC*H*$_3$), 1.39 (quint, *J*=7.5 Hz, 2H, C*H*$_2$CH$_2$COO), 1.34-1.08 (m, 12H, CH$_2$), 0.86 (t, *J*=6.8 Hz, 3H, CH$_2$C*H*$_3$) ppm. ^{13}C{H} NMR (101 MHz, CDCl$_3$) δ$_C$: 174.4 (Nap), 173.7 (SFA), 157.9, 135.4, 133.9, 129.4, 129.1, 127.3, 126.2, 126.1, 119.2, 105.7, 72.8, 61.9, 61.7, 55.5, 45.6, 34.0, 32.0, 29.6, 29.4, 29.4, 29.2, 24.8, 22.8, 18.5, 14.3 ppm. HRMS (ESI) *m*/*z*: [M + Na]$^+$ calcd for $C_{27}H_{38}O_6Na$ 481.2561; found, 481.2561.

3.4.18. Synthesis of 1-dodecanoyl-2-[(*S*)-2-(6-methoxynaphthalen-2-yl)propanoyl]-*sn*-glycerol, (*S*,*S*')-**9d**

The same procedure was followed as described for (*R*,*S*')-**9a** using Pd/C catalyst (10 mg), 3-*O*-benzyl-1-dodecanoyl-2-[(*S*)-2-(6-methoxynaphthalen-2-yl)propanoyl]-*sn*-glycerol, (*S*,*S*')-**7d** (50 mg, 0.087 mmol), THF (3.5 mL) and n-hexane (5 mL). Purification on 4% boric acid impregnated flash silica gel chromatography using pet. ether/ethyl acetate (1:1) as eluent. A second chromatography was needed to obtain pure product (*S*,*S*')-**9d** as a colourless oil in 87% yield (38 mg, 0.076 mmol). $[\alpha]^{20}_D$ = +17.2 (c. 1.6, CH$_2$Cl$_2$). IR (NaCl, ν_{max} / cm^{-1}): 3501 (br), 2926 (vs), 2854 (vs), 1736 (vs), 1634 (s), 1607 (vs), 1177 (br s). ^{1}H NMR (400 MHz, CDCl$_3$) δ$_H$: 7.71-7.65 (m, 3H, Nap-1,4,8), 7.39 (dd, *J*=8.5, 1.9 Hz, 1H, Nap-3), 7.14 (dd, *J*=8.9, 2.5 Hz, 1H, Nap-7), 7.10 (d, *J*=2.5 Hz, 1H, Nap-5), 5.12-5.03 (m, 1H, CH *sn*-2), 4.19 (dd, *J*=11.9, 4.4 Hz, 1H, CH$_2$ *sn*-1), 4.12 (dd, *J*=11.9, 6.1 Hz, 1H, CH$_2$ *sn*-3), 3.91 (s, 3H, OCH$_3$), 3.72 ((q, *J*=7.1 Hz, C*H*CH$_3$), 3.63-3.56 (m, 2H, CH$_2$ *sn*-1), 2.05-1.90 (m, 2H, CH$_2$COO), 1.59 (d, *J*=7.1 Hz, 3H, CHC*H*$_3$), 1.43-1.34 (m, 2H, C*H*$_2$CH$_2$COO), 1.32-1.09 (m, 16H, CH$_2$), 0.86 (t, *J*=6.8 Hz, 3H, CH$_2$C*H*$_3$) ppm. ^{13}C{H} NMR (101 MHz, CDCl$_3$) δ$_C$: 174.4 (Nap), 173.7 (SFA), 157.9, 135.4, 133.9, 129.4, 129.1, 127.3, 126.2, 126.1, 119.2, 105.7, 72.8, 61.9, 61.7, 55.4, 45.6, 33.9, 32.1, 29.8 (2), 29.8 (2), 29.6, 29.5, 29.4, 29.2, 24.8, 22.8, 18.4, 14.3 ppm. HRMS (ESI) *m*/*z*: [M + Na]$^+$ calcd for $C_{29}H_{42}O_6Na$ 509.2874; found, 509.2874.

3.4.19. Synthesis of 2-[(*S*)-2-(6-methoxynaphthalen-2-yl)propanoyl]-1-tetradecanoyl-*sn*-glycerol, (*S*,*S*')-**9e**

The same procedure was followed as described for (*R*,*S*')-**9a** using Pd/C catalyst (5 mg), 3-*O*-benzyl-2-[(*S*)-2-(6-methoxynaphthalen-2-yl)propanoyl]-1-tetradecanoyl-*sn*-glycerol, (*S*,*S*')-**7e** (30 mg, 0.050 mmol), THF (1.5 mL) and n-hexane (2.5 mL). Purification on 4% boric acid impregnated flash silica gel chromatography using pet. ether/ethyl acetate (1:1) as eluent, afforded the product (*S*,*S*')-**9e** as a pale-yellow oil in 90% yield (23 mg, 0.045 mmol). $[\alpha]^{20}_D$ = +17.9 (c. 3.0, CH$_2$Cl$_2$). IR (NaCl, ν_{max} / cm^{-1}): 3366 (br), 3061 (s), 2924 (vs), 854 (vs), 1739 (vs), 1634 (s), 1607 (vs), 1162 (br s). ^{1}H NMR (400 MHz, CDCl$_3$) δ$_H$: 7.72-7.63 (m, 3H, Nap-1,4,8), 7.39 (dd, *J*=8.5, 1.9 Hz, 1H, Nap-3), 7.14 (dd, *J*=8.9, 2.5 Hz, 1H, Nap-7), 7.10 (d, *J*=2.5 Hz, 1H, Nap-5), 5.12-5.03 (m, 1H, CH *sn*-2), 4.19 (dd, *J*=11.9, 4.4 Hz, 1H, CH$_2$ *sn*-1), 4.12 (dd, *J*=11.9, 6.1 Hz, 1H, CH$_2$ *sn*-1), 3.91 (s, 3H, OCH$_3$), 3.71 ((q, *J*=7.1 Hz, C*H*CH$_3$), 3.64-3.56 (m, 2H, CH$_2$ *sn*-3), 2.07-1.90 (m, 2H, CH$_2$COO), 1.59 (d, *J*=7.1 Hz, 3H, CHC*H*$_3$), 1.44-1.34 (m, 2H, C*H*$_2$CH$_2$COO), 1.32-1.09 (m, 20H, CH$_2$), 0.86 (t, *J*=6.8 Hz, 3H, CH$_2$C*H*$_3$) ppm. ^{13}C{H} NMR (101 MHz, CDCl$_3$) δ$_C$: 174.4 (Nap), 173.7 (SFA), 157.9, 135.4, 133.9, 129.4, 129.0, 127.3, 126.3, 126.1, 119.2, 105.7, 72.7, 62.0, 61.7, 55.4, 45.5, 33.9, 32.1, 29.8 (2), 29.8, 29.8, 29.6, 29.5, 29.4, 29.2, 24.8, 22.83, 18.4, 14.3 ppm. HRMS (ESI) *m*/*z*: [M + Na]$^+$ calcd for $C_{31}H_{46}O_6Na$ 537.3187; found, 537.3189.

3.4.20. Synthesis of 1-hexadecanoyl-2-[(*S*)-2-(6-methoxynaphthalen-2-yl)propanoyl]-*sn*-glycerol, (*S*,*S*')-**9f**

The same procedure was followed as described for (*R*,*S*')-**9a** using Pd/C catalyst (8 mg), 3-*O*-benzyl-1-hexadecanoyl-2-[(*S*)-2-(6-methoxynaphthalen-2-yl)propanoyl]-*sn*-glycerol, (*S*,*S*')-**7f** (46 mg, 0.073 mmol), THF (2.8 mL) and n-hexane (4.1 mL). Purification on 4% boric acid impregnated flash silica gel chromatography using pet. ether/ethyl acetate (1:1) as eluent, afforded the product (*S*,*S*')-**9f** as a pale-yellow oil in 90% yield (36 mg, 0.066 mmol). $[\alpha]^{20}_D$ = +17.0 (c. 3.5, CH$_2$Cl$_2$). IR (NaCl, ν_{max} / cm^{-1}): 3409 (br), 3060 (s), 2924 (vs), 2853 (vs), 1738 (vs), 1635 (s), 1607 (vs), 1175 (br s). ^{1}H NMR (400 MHz, CDCl$_3$) δ$_H$: 7.78-7.53 (m, 3H, Nap-1,4,8), 7.39 (dd, *J*=8.5, 1.9 Hz, 1H, Nap-3), 7.14 (dd, *J*=8.9, 2.5 Hz, 1H, Nap-7), 7.10 (d, *J*=2.5 Hz, 1H, Nap-5), 5.12-5.03 (m, 1H, CH *sn*-2), 4.19 (dd, *J*=11.9, 4.4 Hz, 1H, CH$_2$ *sn*-1), 4.12 (dd, *J*=11.9, 6.1 Hz, 1H, CH$_2$ *sn*-1), 3.91 (s, 3H, OCH$_3$), 3.72 ((q, *J*=7.2 Hz, C*H*CH$_3$), 3.64-3.55 (m, 2H, CH$_2$ *sn*-3), 2.06-1.91 (m, 2H, CH$_2$COO), 1.59 (d, *J*=7.1 Hz, 3H, CHC*H*$_3$), 1.43-1.33 (m, 2H, C*H*$_2$CH$_2$COO), 1.30-1.22 (m, 24H, CH$_2$), 0.86 (t, *J*=6.8 Hz,

3H, CH_2CH_3) ppm. $^{13}C\{H\}$ NMR (101 MHz, $CDCl_3$) δ_C: 174.4 (Nap), 173.7 (SFA), 157.9, 135.4, 133.9, 129.4, 129.1, 127.3, 126.2, 126.1, 119.2, 105.7, 72.8, 61.9, 61.7, 55.4, 45.6, 33.9, 32.1, 29.9 (2), 29.8 (2), 29.8, 29.6, 29.5, 29.4, 29.2, 24.8, 22.8, 18.4, 14.3 ppm. HRMS (ESI) *m/z*: $[M + Na]^+$ calcd for $C_{33}H_{50}O_6Na$ 565.3500; found, 565.3496.

*3.5. Coupling of EPA: Synthesis of (S,S')-**10b-f**, (R,S')-**10b-f**, (S,S')-**11b-f** and (R,S')-**11b-f***

3.5.1. Synthesis of 1-[5Z,8Z,11Z,14Z,17Z)-eicosa-5,8,11,14,17-pentaenoyl]-2-[(*S*)-2-(4-isobutylphenyl)-propanoyl]-3-octanoyl-*sn*-glycerol, (*S,S*')-**10b**

The same procedure was followed as described for (*S,S*')-**10a** using 2-[(*S*)-2-(4-isobutylphenyl)propanoyl]-3-octanoyl-*sn*-glycerol (*R,S*')-**8b** (37 mg, 0.096 mmol), EPA (32 mg, 0.095 mmol), CH_2Cl_2 (3 mL), DMAP (10 mg, 0.078 mmol) and EDCI (18 mg, 0.094 mmol). Purification on a silica gel chromatography using pet. ether/ethyl acetate (4:1) as eluent afforded the product (*S,S*')-**10b** as a pale-yellow oil, in 91% yield (60 mg, 0.087 mmol). $[\alpha]^{20}_D$ = +9.60 (c. 4.0, CH_2Cl_2). ^1H NMR (400 MHz, $CDCl_3$) δ_H: 7.18 (d, *J*=8.1 Hz, 2H, Ibu-2,6), 7.07 (d, *J*=8.1 Hz, 2H, Ibu-3,5), 5.41-5.33 (m, 10H, =CH), 5.32-5.24 (m, 1H, CH *sn*-2), 4.30 (dd, *J*=11.9, 4.2 Hz, 1H, CH_2 *sn*-1/3), 4.19 (dd, *J*=11.9, 4.5 Hz, 1H, CH_2 *sn*-1/3), 4.13 (dd, J=11.9, 6.0 Hz, 1H, CH_2 *sn*-1/3), 4.07 (dd, J=11.9, 6.4 Hz, 1H, CH_2 *sn*-1/3), 3.70 (q, *J*=7.1 Hz, 1H, C*H*CH_3), 2.86-2.80 (m, 8H, =CHC*H*$_2$CH=), 2.43 (d, *J*=7.2 Hz, 2H, C*H*$_2$CH$(CH_3)_2$), 2.28 (t, *J*=7.5 Hz, 2H, CH_2COO EPA), 2.18 (t, *J*=7.2 Hz, 2H, CH_2COO), 2.09-2.00 (m, 4H, CH_2C*H*$_2$CH= and =CHC*H*$_2$$CH_3$), 1.84 (nonet, *J*=6.8 Hz, 1H, C*H*$(CH_3)_2$), 1.63-1.50 (m, 4H, C*H*$_2$$CH_2$COO SFA and C*H*$_2$$CH_2$COO EPA), 1.49 (d, *J*=7.2 Hz, 3H, CHC*H*$_3$), 1.32-1.26 (m, 8H, CH_2), 0.97 (t, *J*=7.5 Hz, 3H, CH_3 EPA), 0.90 (t, *J*=7.2 Hz, 3H, CH_3 SFA), 0.89 (d, *J*=6.6 Hz, 6H, CH(C*H*$_3$)$_2$) ppm. $^{13}C\{H\}$ NMR (101 MHz, $CDCl_3$) δ_C: 173.9 (Ibu), 173.4 (SFA), 173.0 (EPA), 140.7, 137.4, 132.2, 129.4 (2), 129.0, 129.0, 128.7, 128.4, 128.3, 128.3, 128.2, 128.0, 127.3 (2), 127.1, 69.3, 62.2, 62.1, 45.2, 45.1, 34.1, 33.4, 31.8, 30.3, 29.2, 29.0, 26.6, 25.8 (2), 25.7 (2), 25.0, 24.7, 22.7 (2), 22.5, 20.7, 18.5, 14.4, 14.0 ppm. HRMS (ESI) *m/z*: $[M + Na]^+$ calcd for $C_{44}H_{66}O_6Na$ 713.4752; found, 713.4747.

3.5.2. Synthesis of 3-decanoyl-1-[5Z,8Z,11Z,14Z,17Z)-eicosa-5,8,11,14,17-pentaenoyl]-2-[(*S*)-2-(4-isobutyl-phenyl)propanoyl]-*sn*-glycerol, (*S,S*')-**10c**

The same procedure was followed as described for (*S,S*')-**10a** using 3-decanoyl-2-[(*S*)-2-(4-isobutylphenyl)propanoyl]-*sn*-glycerol (*R,S*')-**8c** (45 mg, 0.103 mmol), EPA (33 mg, 0.109 mmol), CH_2Cl_2 (4 mL), DMAP (13 mg, 0.106 mmol) and EDCI (28 mg, 0.146 mmol). Purification on a silica gel chromatography using pet. ether/ethyl acetate (4:1) as eluent afforded the product (*S,S*')-**10c** as a pale-yellow oil, in 88% yield (66 mg, 0.091 mmol). $[\alpha]^{20}_D$ = +8.40 (c. 6.5, CH_2Cl_2). ^1H NMR (400 MHz, $CDCl_3$) δ_H: 7.18 (d, *J*=8.1 Hz, 2H, Ibu-2,6), 7.07 (d, *J*=8.1 Hz, 2H, Ibu-3,5), 5.41-5.33 (m, 10H, =CH), 5.32-5.24 (m, 1H, CH *sn*-2), 4.30 (dd, *J*=11.9, 4.2 Hz, 1H, CH_2 *sn*-1/3), 4.19 (dd, *J*=11.9, 4.5 Hz, 1H, CH_2 *sn*-1/3), 4.13 (dd, J=11.9, 6.0 Hz, 1H, CH_2 *sn*-1/3), 4.07 (dd, J=11.9, 6.4 Hz, 1H, CH_2 *sn*-1/3), 3.70 (q, *J*=7.1 Hz, 1H, C*H*CH_3), 2.86-2.80 (m, 8H, =CHC*H*$_2$CH=), 2.43 (d, *J*=7.2 Hz, 2H, C*H*$_2$CH$(CH_3)_2$), 2.28 (t, *J*=7.6 Hz, 2H, CH_2COO EPA), 2.18 (t, *J*=7.2 Hz, 2H, CH_2COO), 2.09-2.00 (m, 4H, CH_2C*H*$_2$CH= and =CHC*H*$_2$$CH_3$), 1.84 (nonet, *J*=6.8 Hz, 1H, C*H*$(CH_3)_2$), 1.62-1.51 (m, 4H, C*H*$_2$$CH_2$COO SFA and C*H*$_2$$CH_2$COO EPA), 1.49 (d, *J*=7.2 Hz, 3H, CHC*H*$_3$), 1.32-1.26 (m, 12H, CH_2), 0.97 (t, *J*=7.5 Hz, 3H, CH_3 EPA), 0.90 (t, *J*=7.2 Hz, 3H, CH_3 SFA), 0.89 (d, *J*=6.6 Hz, 6H, CH(C*H*$_3$)$_2$) ppm. $^{13}C\{H\}$ NMR (101 MHz, $CDCl_3$) δ_C: 173.9 (Ibu), 173.4 (SFA), 173.0 (EPA), 140.7, 137.4, 132.1, 129.4 (2), 129.1, 128.9, 128.7, 128.4, 128.3, 128.3, 128.2, 129.0, 128.2 (2), 127.1, 69.3, 62.2, 62.1, 45.2, 45.2, 34.1, 33.4, 32.0, 30.3, 29.6, 29.5, 29.4, 29.2, 26.6, 25.8 (2), 25.7 (2), 25.0, 24.7, 22.8 (2), 22.5, 20.7, 18.5, 14.4, 14.2 ppm. HRMS (ESI) *m/z*: $[M + Na]^+$ calcd for $C_{46}H_{70}O_6Na$ 741.5065; found, 741.5067.

3.5.3. Synthesis of 3-dodecanoyl-1-[5Z,8Z,11Z,14Z,17Z)-eicosa-5,8,11,14,17-pentaenoyl]-2-[(*S*)-2-(4-iso-butylphenyl)propanoyl]-*sn*-glycerol, (*S,S*')-**10d**

The same procedure was followed as described for (*S,S'*)-**10a** using 3-dodecanoyl-2-[(*S*)-2-(4-isobutylphenyl)propanoyl]-*sn*-glycerol (*R,S'*)-**8d** (40 mg, 0.090 mmol), EPA (26 mg, 0.087 mmol), CH_2Cl_2 (3 mL), DMAP (9 mg, 0.071 mmol) and EDCI (17 mg, 0.086 mmol). Purification on a silica gel chromatography using pet. ether/ethyl acetate (4:1) as eluent afforded the product (*S,S'*)-**10d** as a pale-yellow oil, in 87% yield (57 mg, 0.078 mmol). $[\alpha]^{20}_D$ = +7.98 (c. 5.5, CH_2Cl_2). ^{1}H NMR (400 MHz, $CDCl_3$) δ_H: 7.18 (d, *J*=8.1 Hz, 2H, Ibu-2,6), 7.07 (d, *J*=8.1 Hz, 2H, Ibu-3,5), 5.41-5.33 (m, 10H, =CH), 5.32-5.24 (m, 1H, CH *sn*-2), 4.30 (dd, *J*=11.9, 4.2 Hz, 1H, CH_2 *sn*-1/3), 4.19 (dd, *J*=11.9, 4.5 Hz, 1H, CH_2 *sn*-1/3), 4.13 (dd, J=11.9, 6.0 Hz, 1H, CH_2 *sn*-1/3), 4.07 (dd, J=11.9, 6.4 Hz, 1H, CH_2 *sn*-1/3), 3.70 (q, *J*=7.1 Hz, 1H, C*H*CH_3), 2.86-2.80 (m, 8H, =CHC*H*$_2$CH=), 2.43 (d, *J*=7.2 Hz, 2H, C*H*$_2$CH(CH_3)$_2$), 2.27 (t, *J*=7.6 Hz, 2H, CH_2COO EPA), 2.18 (t, *J*=7.2 Hz, 2H, CH_2COO), 2.09-2.00 (m, 4H, CH_2C*H*$_2$CH= and =CHC*H*$_2$$CH_3$), 1.84 (nonet, *J*=6.8 Hz, 1H, C*H*(CH_3)$_2$), 1.66-1.53 (m, 4H, C*H*$_2$$CH_2$COO SFA and C*H*$_2$$CH_2$COO EPA), 1.48 (d, *J*=7.2 Hz, 3H, CHC*H*$_3$), 1.32-1.26 (m, 16H, CH_2), 0.97 (t, *J*=7.5 Hz, 3H, CH_3 EPA), 0.90 (t, *J*=7.2 Hz, 3H, CH_3 SFA), 0.89 (d, *J*=6.6 Hz, 6H, CH(C*H*$_3$)$_2$) ppm. ^{13}C{H} NMR (101 MHz, $CDCl_3$) δ_C: 173.9 (Ibu), 173.4 (SFA), 173.0 (EPA), 140.7, 137.4, 132.1, 129.4 (2), 129.0, 128.9, 128.7, 128.4, 128.3, 128.3, 128.2, 128.0, 128.2 (2), 127.1, 69.3, 62.2, 62.1, 45.2, 45.15, 34.1, 33.4, 32.0, 30.3, 29.7 (2), 29.6, 29.5, 29.4, 29.2, 26.6, 25.6 (2), 25.7 (2), 25.0, 24.7, 22.8 (2), 22.5, 20.7, 18.5, 14.4, 14.2 ppm. HRMS (ESI) *m/z*: [M + Na]$^+$ calcd for $C_{48}H_{74}O_6Na$ 769.5378; found, 769.5366.

3.5.4. Synthesis of 1-[5Z,8Z,11Z,14Z,17Z)-eicosa-5,8,11,14,17-pentaenoyl]-2-[(*S*)-2-(4-isobutylphenyl)-propanoyl]-3-tetradecanoyl-*sn*-glycerol, (*S,S'*)-**10e**

The same procedure was followed as described for (*S,S'*)-**10a** using 2-[(*S*)-2-(4-isobutylphenyl)propanoyl]-3-tetradecanoyl-*sn*-glycerol (*R,S'*)-**8e** (49 mg, 0.100 mmol), EPA (33 mg, 0.110 mmol), CH_2Cl_2 (3.8 mL), DMAP (11 mg, 0.090 mmol) and EDCI (21 mg, 0.109 mmol). Purification on a silica gel chromatography using pet. ether/ethyl acetate (8.5:1.5) as eluent afforded the product (*S,S'*)-**10e** as a pale-yellow oil, in 89% yield (67 mg, 0.089 mmol). $[\alpha]^{20}_D$ = +6.58 (c. 6.4, CH_2Cl_2). ^{1}H NMR (400 MHz, $CDCl_3$) δ_H: 7.18 (d, *J*=8.1 Hz, 2H, Ibu-2,6), 7.07 (d, *J*=8.1 Hz, 2H, Ibu-3,5), 5.40-5.35 (m, 10H, =CH), 5.34-5.25 (m, 1H, CH *sn*-2), 4.30 (dd, *J*=11.9, 4.2 Hz, 1H, CH_2 *sn*-1/3), 4.19 (dd, *J*=11.9, 4.5 Hz, 1H, CH_2 *sn*-1/3), 4.13 (dd, J=11.9, 6.0 Hz, 1H, CH_2 *sn*-1/3), 4.07 (dd, J=11.9, 6.4 Hz, 1H, CH_2 *sn*-1/3), 3.70 (q, *J*=7.1 Hz, 1H, C*H*CH_3), 2.86-2.80 (m, 8H, =CHC*H*$_2$CH=), 2.43 (d, *J*=7.2 Hz, 2H, C*H*$_2$CH(CH_3)$_2$), 2.27 (t, *J*=7.6 Hz, 2H, CH_2COO EPA), 2.18 (t, *J*=7.2 Hz, 2H, CH_2COO), 2.10-2.01 (m, 4H, CH_2C*H*$_2$CH= and =CHC*H*$_2$$CH_3$), 1.84 (nonet, *J*=6.8 Hz, 1H, C*H*(CH_3)$_2$), 1.65-1.54 (m, 4H, C*H*$_2$$CH_2$COO SFA and C*H*$_2$$CH_2$COO EPA), 1.48 (d, *J*=7.2 Hz, 3H, CHC*H*$_3$), 1.32-1.26 (m, 20H, CH_2), 0.97 (t, *J*=7.5 Hz, 3H, CH_3 EPA), 0.88 (t, *J*=7.2 Hz, 3H, CH_3 SFA), 0.87 (d, *J*=6.6 Hz, 6H, CH(C*H*$_3$)$_2$) ppm. ^{13}C{H} NMR (101 MHz, $CDCl_3$) δ_C: 173.9 (Ibu), 173.4 (SFA), 173.0 (EPA), 140.7, 137.4, 132.2, 129.4 (2), 129.0, 129.0, 128.7, 128.4, 128.3, 128.3, 128.2, 128.0, 128.2, 127.1 (2), 69.3, 62.2, 62.1, 45.2, 45.1, 34.2, 33.4, 32.1, 30.3, 30.1, 29.8, 29.8, 29.8, 29.6, 29.5, 29.4, 29.3, 26.6, 25.8 (2), 25.7 (2), 25.0, 24.7, 22.8 (2), 22.5, 20.7, 18.5, 14.4, 14.2 ppm. HRMS (ESI) *m/z*: [M + Na]$^+$ calcd for $C_{50}H_{78}O_6Na$ 797.5691; found, 797.5685.

3.5.5. Synthesis of 3-hexadecanoyl-1-[5Z,8Z,11Z,14Z,17Z)-eicosa-5,8,11,14,17-pentaenoyl]-2-[(*S*)-2-(4-isobutylphenyl)propanoyl]-*sn*-glycerol, (*S,S'*)-**10f**

The same procedure was followed as described for (*S,S'*)-**10a** using 3-hexadecanoyl-2-[(*S*)-2-(4-isobutylphenyl)propanoyl]-*sn*-glycerol (*R,S'*)-**8f** (42 mg, 0.081 mmol), EPA (27 mg, 0.089 mmol), CH_2Cl_2 (3 mL), DMAP (9 mg, 0.073 mmol) and EDCI (17 mg, 0.088 mmol). Purification on a silica gel chromatography using pet. ether/ethyl acetate (8.5:1.5) as eluent afforded the product (*S,S'*)-**10f** as a pale-yellow oil, in 86% yield (56 mg, 0.070 mmol). $[\alpha]^{20}_D$ = +6.68 (c. 6.5, CH_2Cl_2). ^{1}H NMR (400 MHz, $CDCl_3$) δ_H: 7.18 (d, *J*=8.1 Hz, 2H, Ibu-2,6), 7.07 (d, *J*=8.1 Hz, 2H, Ibu-3,5), 5.40-5.35 (m, 10H, =CH), 5.34-5.25 (m, 1H, CH *sn*-2), 4.30 (dd, *J*=11.9, 4.2 Hz, 1H, CH_2 *sn*-1/3), 4.19 (dd, *J*=11.9, 4.5 Hz, 1H, CH_2 *sn*-1/3), 4.13 (dd, J=11.9, 6.0 Hz, 1H, CH_2 *sn*-1/3), 4.07 (dd, J=11.9, 6.4 Hz, 1H, CH_2 *sn*-1/3), 3.70 (q, *J*=7.1 Hz, 1H, C*H*CH_3), 2.86-2.80 (m, 8H, =CHC*H*$_2$CH=), 2.43 (d, *J*=7.2 Hz, 2H, C*H*$_2$CH(CH_3)$_2$), 2.27 (t, *J*=7.6 Hz, 2H, CH_2COO EPA), 2.18 (t, *J*=7.2 Hz, 2H, CH_2COO), 2.10-2.01 (m, 4H, CH_2C*H*$_2$CH= and =CHC*H*$_2$$CH_3$), 1.84 (nonet, *J*=6.8 Hz, 1H, C*H*(CH_3)$_2$), 1.65-1.54 (m, 4H,

CH_2CH$_2$COO SFA and CH_2CH$_2$COO EPA), 1.48 (d, *J*=7.2 Hz, 3H, CHCH_3), 1.32-1.26 (m, 24H, CH$_2$), 0.97 (t, *J*=7.5 Hz, 3H, CH$_3$ EPA), 0.88 (t, *J*=7.2 Hz, 3H, CH$_3$ SFA), 0.87 (d, *J*=6.6 Hz, 6H, CH(CH_3)$_2$) ppm. ^{13}C{H} NMR (101 MHz, CDCl$_3$) δC: 174.1 (Ibu), 173.8 (SFA), 173.3 (EPA), 140.7, 137.4, 132.2, 129.4 (2), 129.0, 129.0, 128.7, 128.4, 128.3, 128.3, 128.2, 128.0, 128.2 (2), 127.1, 69.3, 62.2, 62.1, 45.2, 45.1, 34.2, 33.4, 32.1, 30.3, 30.1 (2), 29.8 (2), 29.8, 29.8, 29.6, 29.5, 29.4, 29.3, 26.6, 25.8 (2), 25.7 (2), 25.0, 24.7, 22.8 (2), 22.5, 20.7, 18.5, 14.4, 14.2 ppm. HRMS (ESI) *m*/*z*: [M + Na]$^+$ calcd for $C_{52}H_{82}O_6Na$ 825.6004; found, 825.5950.

3.5.6. Synthesis of 3-[5Z,8Z,11Z,14Z,17Z)-eicosa-5,8,11,14,17-pentaenoyl]-2-[(*S*)-2-(4-isobutylphenyl)-propanoyl]-1-octanoyl-*sn*-glycerol, (*R*,*S*')-**10b**

The same procedure was followed as described for (*S*,*S*')-**10a** using 2-[(*S*)-2-(4-isobutylphenyl)propanoyl]-1-octanoyl-*sn*-glycerol (*S*,*S*')-**8b** (29 mg, 0.071 mmol), EPA (23 mg, 0.076 mmol), CH_2Cl_2 (3 mL), DMAP (10 mg, 0.077 mmol) and EDCI (21 mg, 0.110 mmol). Purification on a silica gel chromatography using pet. ether/ethyl acetate (4:1) as eluent afforded the product (*R*,*S*')-**10b** as a pale-yellow oil, in 84% yield (41 mg, 0.059 mmol). [*α*]$^{20}_D$ = +9.11 (c. 4.0, CH_2Cl_2). IR (NaCl, ν_{max} / cm^{-1}): 3013 (vs), 2957 (vs), 2929 (vs, 2859 (vs), 1743 (vs). ^{1}H NMR (400 MHz, CDCl$_3$) δH: 7.18 (dd, *J*=8.1, 1.7 Hz, 2H, Ibu-2,6), 7.07 (dd, *J*=8.1, 3.8 Hz, 2H, Ibu-3,5), 5.45-5.29 (m, 10H, =CH), 5.28-5.22 (m, 1H, CH *sn*-2), 4.30 (dd, *J*=11.9, 4.3 Hz, 1H, CH$_2$ *sn*-1/3), 4.19 (dd, *J*=11.9, 4.3 Hz, 1H, CH$_2$ *sn*-1/3), 4.13 (dd, J=11.9, 6.4 Hz, 1H, CH$_2$ *sn*-1/3), 4.06 (dd, J=11.9, 6.3 Hz, 1H, CH$_2$ *sn*-1/3), 3.70 (q, *J*=7.1 Hz, 1H, C*H*CH$_3$), 2.87-2.77 (m, 8H, =CHCH_2CH=), 2.44 (d, *J*=7.2 Hz, 2H, CH_2CH(CH$_3$)$_2$), 2.33-2.22 (m, 2H, CH$_2$COO EPA), 2.18-2.04 (m, 6H, CH$_2$COO SFA, CH$_2$CH_2CH= and =CHCH_2CH$_3$), 1.84 (nonet, *J*=6.8 Hz, 1H, C*H*(CH$_3$)$_2$), 1.61-1.59 (m, 2H, CH_2CH$_2$COO EPA), 1.58-1.50 (m, 2H, CH_2CH$_2$COO SFA), 1.49 (d, *J*=7.2 Hz, 3H, CHCH_3), 1.33-1.20 (m, 8H, CH$_2$), 0.97 (t, *J*=7.5 Hz, 3H, CH$_3$ EPA), 0.89 (d, *J*=6.6 Hz, 6H, CH(CH_3)$_2$), 0.88 (t, *J*=6.5 Hz, 3H, CH$_3$ SFA) ppm. ^{13}C{H} NMR (101 MHz, CDCl$_3$) δC: 173.8 (bu), 173.1 (SFA), 172.9 (EPA), 140.6, 137.2, 132.0, 129.3 (2), 129.2, 128.9, 128.7, 128.9, 128.8, 128.6, 128.2, 128.1, 127.8 (2), 127.1, 69.2, 62.1, 61.9, 45.2, 45.2, 34.0, 33.5, 31.6, 30.2, 29.7 (2), 29.0, 28.9, 26.5, 25.6 (2), 25.5 (2), 24.8, 24.7, 22.6 (2), 22.4, 20.5, 18.3, 14.2, 14.0 ppm. HRMS (ESI) *m*/*z*: [M + Na]$^+$ calcd for $C_{44}H_{66}O_6Na$ 713.4752; found, 713.4755.

3.5.7. Synthesis of 1-decanoyl-3-[5Z,8Z,11Z,14Z,17Z)-eicosa-5,8,11,14,17-pentaenoyl]-2-[(*S*)-2-(4-isobutyl-phenyl)propanoyl]-*sn*-glycerol, (*R*,*S*')-**10c**

The same procedure was followed as described for (*S*,*S*')-**10a** using 1-decanoyl-2-[(*S*)-2-(4-isobutylphenyl)propanoyl]-*sn*-glycerol (*S*,*S*')-**8c** (55 mg, 0.127 mmol), EPA (43 mg, 0.142 mmol), CH_2Cl_2 (4 mL), DMAP (18 mg, 0.147 mmol) and EDCI (42 mg, 0.219 mmol). Purification on a silica gel chromatography using pet. ether/ethyl acetate (9:1) as eluent afforded the product (*R*,*S*')-**10c** as a pale-yellow oil, in 76% yield (70 mg, 0.097 mmol). [*α*]$^{20}_D$ = +7.44 (c. 5.9, CH_2Cl_2). IR (NaCl, ν_{max} / cm^{-1}): 3013 (s), 2956 (vs), 2927 (vs), 2855 (vs), 1743 (vs). ^{1}H NMR (400 MHz, CDCl$_3$) δH: 7.21-7.13 (m, 2H, Ibu-2,6), 7.10-7.05 (m, 2H, Ibu-3,5), 5.45-5.29 (m, 10H, =CH), 5.25 (tt, *J*=6.3, 4.3 Hz, 1H, CH *sn*-2), 4.30 (dd, *J*=11.9, 4.3 Hz, 1H, CH$_2$ *sn*-1/3), 4.19 (dd, *J*=11.9, 4.3 Hz, 1H, CH$_2$ *sn*-1/3), 4.14 (dd, J=11.9, 6.3 Hz, 1H, CH$_2$ *sn*-1/3), 4.06 (dd, J=11.9, 6.3 Hz, 1H, CH$_2$ *sn*-1/3), 3.70 (q, *J*=7.1 Hz, 1H, C*H*CH$_3$), 2.88-2.78 (m, 8H, =CHCH_2CH=), 2.44 (d, *J*=7.2 Hz, 2H, CH_2CH(CH$_3$)$_2$), 2.30 (t, *J*=7.6 Hz, 2H, CH$_2$COO EPA), 2.17-2.05 (m, 6H, CH$_2$COO SFA, CH$_2$CH_2CH= and =CHCH_2CH$_3$), 1.84 (nonet, *J*=6.7 Hz, 1H, C*H*(CH$_3$)$_2$), 1.75-1.63 (m, 2H, CH_2CH$_2$COO EPA), 1.52-1.48 (m, 2H, CH_2CH$_2$COO SFA), 1.49 (d, *J*=7.2 Hz, 3H, CHCH_3), 1.34-1.22 (m, 12H, CH$_2$), 0.97 (t, *J*=7.5 Hz, 3H, CH$_3$ EPA), 0.89 (d, *J*=6.7 Hz, 6H, CH(CH_3)$_2$), 0.88 (t, *J*=7.1 Hz, 3H, CH$_3$ SFA) ppm. ^{13}C{H} NMR (101 MHz, CDCl$_3$) δC: 173.9 (Ibu), 173.3 (SFA), 173.1 (EPA), 140.7, 137.4, 132.2, 129.5 (2), 129.4, 129.08, 129.1, 128.9, 128.7, 128.4, 128.4, 128.3, 128.3 (2), 127.1, 69.3, 62.3, 62.1, 45.2, 45.2, 34.0, 33.5, 32.0, 30.3, 29.4, 29.2, 26.6, 26.6, 25.8, 25.7 (2), 25.0 (2), 24.9, 24.8, 22.8 (2), 22.5, 20.7, 18.5, 14.4, 14.2 ppm. HRMS (ESI) *m*/*z*: [M + Na]$^+$ calcd for $C_{46}H_{70}O_6Na$ 741.5065; found, 741.5064.

3.5.8. Synthesis of 1-dodecanoyl-3-[5Z,8Z,11Z,14Z,17Z)-eicosa-5,8,11,14,17-pentaenoyl]-2-[(*S*)-2-(4-iso-butylphenyl)propanoyl]-*sn*-glycerol, (*R,S*')-**10d**

The same procedure was followed as described for (*S,S*')-**10a** using 1-dodecanoyl-2-[(*S*)-2-(4-isobutylphenyl)propanoyl]-*sn*-glycerol (*S,S*')-**8d** (30 mg, 0.065 mmol), EPA (31 mg, 0.099 mmol), CH_2Cl_2 (4 mL), DMAP (14 mg, 0.112 mmol) and EDCI (32 mg, 0.166 mmol). Purification on a silica gel chromatography using pet. ether/ethyl acetate (9:1) as eluent afforded the product (*R,S*')-**10d** as a yellow oil, in 75% yield (37 mg, 0.049 mmol). $[\alpha]^{20}_D$ = +7.57 (c. 3.5, CH_2Cl_2). IR (NaCl, ν_{max} / cm^{-1}): 3013 (s), 2950 (vs), 2926 (vs), 2854 (vs), 1743 (vs). ^{1}H NMR (400 MHz, $CDCl_3$) δ_H: 7.18 (d, *J*=8.1 Hz, 2H, Ibu-2,6), 7.07 (d, *J*=8.1 Hz, 2H, Ibu-3,5), 5.44-5.30 (m, 10H, =CH), 5.25 (tt, *J*=6.3, 4.3 Hz, 1H, CH *sn*-2), 4.30 (dd, *J*=11.9, 4.3 Hz, 1H, CH_2 *sn*-1/3), 4.19 (dd, *J*=11.9, 4.3 Hz, 1H, CH_2 *sn*-1/3), 4.14 (dd, J=11.9, 6.3 Hz, 1H, CH_2 *sn*-1/3), 4.06 (dd, J=11.9, 6.3 Hz, 1H, CH_2 *sn*-1/3), 3.70 (q, *J*=7.2 Hz, 1H, C*H*CH_3), 2.89-2.77 (m, 8H, =CHC*H*$_2$CH=), 2.44 (d, *J*=7.1 Hz, 2H, C*H*$_2$CH(CH_3)$_2$), 2.30 (t, *J*=7.6 Hz, 2H, CH_2COO EPA), 2.15 (t, *J*=7.1, 2H, CH_2COO SFA), 2.12-2.03 (m, 4H, CH_2C*H*$_2$CH= and =CHC*H*$_2$$CH_3$), 1.84 (nonet, *J*=6.8 Hz, 1H, C*H*(CH_3)$_2$), 1.68 (quint, *J*=7.5 Hz, 2H, C*H*$_2$$CH_2$COO EPA), 1.54-1.47 (m, 2H, C*H*$_2$$CH_2$COO SFA), 1.49 (d, *J*=7.2 Hz, 3H, CHC*H*$_3$), 1.30-1.22 (m, 16H, CH_2), 0.97 (t, *J*=7.5 Hz, 3H, CH_3 EPA), 0.88 (t, *J*=7.1 Hz, 3H, CH_3 SFA), 0.87 (d, *J*=6.6 Hz, 6H, CH(C*H*$_3$)$_2$) ppm. ^{13}C{H} NMR (101 MHz, $CDCl_3$) δ_C: 173.9 (Ibu), 173.3 (SFA), 173.1 (EPA), 140.7, 137.4, 132.2, 129.4 (2), 129.1, 129.0, 128.7, 128.4, 128.3, 128.3, 128.2, 128.0, 127.3 (2), 127.2, 69.4, 62.3, 62.1, 45.2, 45.1, 34.01, 33.5, 32.1, 30.3, 29.8 (2), 29.6, 29.5, 29.4, 29.3, 26.7, 25.8 (2), 25.7 (2), 24.9, 24.8, 22.8 (2), 22.5, 20.7, 18.5, 14.4, 14.3 ppm. HRMS (ESI) *m*/*z*: $[M + Na]^+$ calcd for $C_{48}H_{74}O_6Na$ 769.5378; found, 769.5409.

3.5.9. Synthesis of 3-[5Z,8Z,11Z,14Z,17Z)-eicosa-5,8,11,14,17-pentaenoyl]-2-[(*S*)-2-(4-isobutylphenyl)-propanoyl]-1-tetradecanoyl-*sn*-glycerol, (*R,S*')-**10e**

The same procedure was followed as described for (*S,S*')-**10a** using 2-[(*S*)-2-(4-isobutylphenyl)propanoyl]-1-tetradecanoyl-*sn*-glycerol (*S,S*')-**8e** (30 mg, 0.061 mmol), EPA (21 mg, 0.069 mmol), CH_2Cl_2 (4 mL), DMAP (10 mg, 0.082 mmol) and EDCI (17 mg, 0.089 mmol). Purification on a silica gel chromatography using pet. ether/ethyl acetate (8.5:1.5) as eluent afforded the product (*R,S*')-**10e** as a pale-yellow oil, in 75% yield (35 mg, 0.046 mmol). $[\alpha]^{20}_D$ = +7.12 (c. 2.5, CH_2Cl_2). IR (NaCl, ν_{max} / cm^{-1}): 3013 (s), 2956 (vs), 2926 (vs), 2854 (vs), 1743 (vs). ^{1}H NMR (400 MHz, $CDCl_3$) δ_H: 7.18 (d, *J*=8.1 Hz, 2H, Ibu-2,6), 7.07 (d, *J*=8.1 Hz, 2H, Ibu-3,5), 5.44-5.30 (m, 10H, =CH), 5.25 (tt, *J*=6.3, 4.3 Hz, 1H, CH *sn*-2), 4.30 (dd, *J*=11.9, 4.3 Hz, 1H, CH_2 *sn*-1/3), 4.18 (dd, *J*=11.9, 4.3 Hz, 1H, CH_2 *sn*-1/3), 4.14 (dd, J=11.9, 6.3 Hz, 1H, CH_2 *sn*-1/3), 4.06 (dd, J=11.9, 6.3 Hz, 1H, CH_2 *sn*-1/3), 3.70 (q, *J*=7.2 Hz, 1H, C*H*CH_3), 2.89-2.77 (m, 8H, =CHC*H*$_2$CH=), 2.44 (d, *J*=7.1 Hz, 2H, C*H*$_2$CH(CH_3)$_2$), 2.30 (t, *J*=7.6 Hz, 2H, CH_2COO EPA), 2.15 (t, *J*=7.1, 2H, CH_2COO SFA), 2.12-2.03 (m, 4H, CH_2C*H*$_2$CH= and =CHC*H*$_2$$CH_3$), 1.84 (nonet, *J*=6.8 Hz, 1H, C*H*(CH_3)$_2$), 1.68 (quint, *J*=7.5 Hz, 2H, C*H*$_2$$CH_2$COO EPA), 1.54-1.47 (m, 2H, C*H*$_2$$CH_2$COO SFA), 1.49 (d, *J*=7.2 Hz, 3H, CHC*H*$_3$), 1.30-1.22 (m, 20H, CH_2), 0.97 (t, *J*=7.5 Hz, 3H, CH_3 EPA), 0.88 (t, *J*=7.1 Hz, 3H, CH_3 SFA), 0.87 (d, *J*=6.6 Hz, 6H, CH(C*H*$_3$)$_2$) ppm. ^{13}C{H} NMR (101 MHz, $CDCl_3$) δ_C: 173.9 (Ibu), 173.3 (SFA), 173.1 (EPA), 140.7, 137.4, 132.2, 129.4 (2), 129.1, 129.0, 128.7, 128.4, 128.3, 128.3, 128.2, 128.0, 127.3 (2), 127.2, 69.4, 62.3, 62.1, 45.2, 45.2, 34.0, 33.5, 32.1, 30.3, 30.1, 29.8, 29.8, 29.8, 29.6, 29.5, 29.4, 29.3, 26.7, 25.8 (2), 25.7 (2), 24.9, 24.8, 22.8 (2), 22.5, 20.7, 18.5, 14.4, 14.3 ppm. HRMS (ESI) *m*/*z*: $[M + Na]^+$ calcd for $C_{50}H_{78}O_6Na$ 797.5691; found, 797.5692.

3.5.10. Synthesis of 3-hexadecanoyl-1-[5Z,8Z,11Z,14Z,17Z)-eicosa-5,8,11,14,17-pentaenoyl]-2-[(*S*)-2-(4-iso-butylphenyl)propanoyl]-*sn*-glycerol, (*R,S*')-**10f**

The same procedure was followed as described for (*S,S*')-**10a** using 1-hexadecanoyl-2-[(*S*)-2-(4-isobutylphenyl)propanoyl]-*sn*-glycerol (*S,S*')-**8f** (12 mg, 0.023 mmol), EPA (8 mg, 0.023 mmol), CH_2Cl_2 (1 mL), DMAP (3 mg, 0.021 mmol) and EDCI (5 mg, 0.025 mmol). Purification on a silica gel chromatography using pet. ether/ethyl acetate (9:1) as eluent afforded the product (*R,S*')-**10f** as a pale-yellow oil, in 78% yield (14 mg, 0.017 mmol). $[\alpha]^{20}_D$ = +7.43 (c. 1.4, CH_2Cl_2). IR (NaCl, ν_{max} / cm^{-1}): 3012 (s), 2965 (vs), 2925 (vs), 2855

(vs), 1744 (vs). ^{1}H NMR (400 MHz, $CDCl_3$) δ_H: 7.18 (d, *J*=8.1 Hz, 2H, Ibu-2,6), 7.07 (d, *J*=8.1 Hz, 2H, Ibu-3,5), 5.46-5.30 (m, 10H, =CH), 5.29-5.19 (m, 1H, CH *sn*-2), 4.29 (dd, *J*=11.9, 4.3 Hz, 1H, CH_2 *sn*-1/3), 4.18 (dd, *J*=11.9, 4.3 Hz, 1H, CH_2 *sn*-1/3), 4.14 (dd, J=11.9, 6.3 Hz, 1H, CH_2 *sn*-1/3), 4.06 (dd, J=11.9, 6.3 Hz, 1H, CH_2 *sn*-1/3), 3.70 (q, *J*=7.1 Hz, 1H, C*H*CH_3), 2.88-2.77 (m, 8H, =CHC*H*$_2$CH=), 2.43 (d, *J*=7.2 Hz, 2H, C*H*$_2$CH(CH_3)$_2$), 2.30 (t, *J*=7.2 Hz, 2H, CH_2COO EPA), 2.18 (t, *J*=7.6, 2H, CH_2COO SFA), 2.19-2.01 (m, 4H, CH_2C*H*$_2$CH= and =CHC*H*$_2$$CH_3$), 1.84 (nonet, *J*=6.8 Hz, 1H, C*H*(CH_3)$_2$), 1.69-1.61 (m, 2H, C*H*$_2$$CH_2$COO EPA), 1.60-1.50 (m, 2H, C*H*$_2$$CH_2$COO SFA), 1.48 (d, *J*=7.2 Hz, 3H, CHC*H*$_3$), 1.32-1.23 (m, 24H, CH_2), 0.97 (t, *J*=7.5 Hz, 3H, CH_3 EPA), 0.89 (t, *J*=7.1 Hz, 3H, CH_3 SFA), 0.88 (d, *J*=6.6 Hz, 6H, CH(C*H*$_3$)$_2$) ppm. ^{13}C{H} NMR (101 MHz, $CDCl_3$) δ_C: 173.9 (Ibu), 173.3 (SFA), 173.1 (EPA), 140.7, 137.4, 132.2 (2), 129.4 (2), 129.1, 129.0, 128.7, 128.4, 128.3, 128.3, 128.0, 127.3, 127.2 (2), 69.3, 62.3, 62.1, 45.2, 45.2, 34.0, 33.5, 32.1, 30.3, 29.9 (2), 29.8 (2), 29.8 (2), 29.7, 29.5, 29.4, 29.3, 26.7, 25.8 (2), 25.7 (2), 24.9, 24.8, 22.8 (2), 22.5, 20.7, 18.5, 14.4, 14.3 ppm. HRMS (ESI) *m/z*: [M + Na]$^+$ calcd for $C_{52}H_{82}O_6Na$ 825.6004; found, 825.6004.

3.5.11. Synthesis of 1-[5Z,8Z,11Z,14Z,17Z)-eicosa-5,8,11,14,17-pentaenoyl]-2[(*S*)-2-(6-methoxynaphthalen-2-yl)propanoyl]-3-octanoyl-*sn*-glycerol, (*S,S*')-**11b**

The same procedure was followed as described for (*S,S*')-**11a** using 2-[(*S*)-2-(4-isobutylphenyl)propanoyl]-3-octanoyl-*sn*-glycerol (*R,S*')-**9b** (35 mg, 0.081 mmol), EPA (27 mg, 0.089 mmol), CH_2Cl_2 (4 mL), DMAP (9 mg, 0.074 mmol) and EDCI (17 mg, 0.089 mmol). Purification on a silica gel chromatography using pet. ether/ethyl acetate (4:1) as eluent afforded the product (*S,S*')-**11b** as a yellow oil, in 94% yield (54 mg, 0.076 mmol). $[\alpha]^{20}_D$ = +6.00 (c. 5.0, CH_2Cl_2). ^{1}H NMR (400 MHz, $CDCl_3$) δ_H: 7.73-7.62 (m, 3H, Nap-1,4,8), 7.38 (dd, *J*=8.5, 1.9 Hz, 1H, Nap-3), 7.14 (dd, *J*=8.9, 2.5 Hz, 1H, Nap-7), 7.10 (d, *J*=2.5 Hz, 1H, Nap-5), 5.45-5.28 (m, 10H, =CH), 5.30-5.19 (m, 1H, CH *sn*-2), 4.30 (dd, *J*=11.9, 4.2 Hz, 1H, CH_2 *sn*-1/3), 4.22-4.09 (m, 2H, CH_2 *sn*-1/3), 4.07 (dd, J=11.9, 6.4 Hz, 1H, CH_2 *sn*-1/3), 3.90 (s, 3H, OCH_3), 3.86 (q, *J*=7.2 Hz, 1H, C*H*CH_3), 2.89-2.72 (m, 8H, =CHC*H*$_2$CH=), 2.29-2.20 (m, 2H, CH_2COO EPA), 2.14-2.01 (m, 2H, CH_2COO SFA), 2.00 (td, *J*=7.6, 5.5 Hz, 2H, CH_2C*H*$_2$CH=), 1.98-1.88 (m, 2H, =CHC*H*$_2$$CH_3$), 1.63-1.51 (m, 5H, C*H*$_2$$CH_2$COO SFA and CHC*H*$_3$), 1.46 (quint, *J*=7.4 Hz, 2H, C*H*$_2$$CH_2$COO EPA), 1.35-1.21 (m, 8H, CH_2), 0.97 (t, *J*=7.5 Hz, 3H, CH_3 EPA), 0.89 (t, *J*=6.8 Hz, 3H, CH_3 SFA) ppm. ^{13}C{H} NMR (101 MHz, $CDCl_3$) δ_C: 173.8 (Nap), 173.4 (SFA), 172.9 (EPA), 157.8, 135.3, 133.8, 132.1, 129.4, 129.0, 128.9, 128.9, 128.7, 128.4, 128.3, 128.3, 128.2, 128.0, 127.2, 127.1, 126.2, 126.1, 119.2, 105.7, 69.5, 62.1, 62.1, 55.4, 45.5, 34.1, 33.2, 31.8, 29.2, 29.0, 26.5, 25.8, 25.7 (2), 25.7, 24.9, 24.6, 22.7, 20.7, 18.4, 14.39, 14.2 ppm. HRMS (ESI) *m/z*: [M + Na]$^+$ calcd for $C_{45}H_{62}O_7Na$ 737.4388; found, 737.4385.

3.5.12. Synthesis of 3-decanoyl-1-[5Z,8Z,11Z,14Z,17Z)-eicosa-5,8,11,14,17-pentaenoyl]-2[(*S*)-2-(6-methoxy-naphthalen-2-yl)propanoyl]-*sn*-glycerol, (*S,S*')-**11c**

The same procedure was followed as described for (*S,S*')-**11a** using 3-decanoyl-2-[(*S*)-2-(4-isobutylphenyl)propanoyl]-*sn*-glycerol (*R,S*')-**9c** (35 mg, 0.076 mmol), EPA (25 mg, 0.084 mmol), CH_2Cl_2 (2.5 mL), DMAP (8 mg, 0.067 mmol) and EDCI (16 mg, 0.081 mmol). Purification on a silica gel chromatography using pet. ether/ethyl acetate (8.5:1.5) as eluent afforded the product (*S,S*')-**11c** as a yellow oil, in 84% yield (48 mg, 0.064 mmol). $[\alpha]^{20}_D$ = +6.58 (c. 4.5, CH_2Cl_2). ^{1}H NMR (400 MHz, $CDCl_3$) δ_H: 7.73-7.62 (m, 3H, Nap-1,4,8), 7.38 (dd, *J*=8.5, 1.9 Hz, 1H, Nap-3), 7.14 (dd, *J*=8.9, 2.6 Hz, 1H, Nap-7), 7.10 (d, *J*=2.6 Hz, 1H, Nap-5), 5.45-5.27 (m, 10H, =CH), 5.29-5.21 (m, 1H, CH *sn*-2), 4.30 (dd, *J*=11.9, 4.2 Hz, 1H, CH_2 *sn*-1/3), 4.22-4.09 (m, 2H, CH_2 *sn*-1/3), 4.06 (dd, J=11.9, 6.4 Hz, 1H, CH_2 *sn*-1/3), 3.90 (s, 3H, OCH_3), 3.86 (q, *J*=7.2 Hz, 1H, C*H*CH_3), 2.89-2.71 (m, 8H, =CHC*H*$_2$CH=), 2.24 (t, *J*=7.4 Hz, 2H, CH_2COO EPA), 2.14-2.02 (m, 2H, CH_2COO SFA), 2.06-1.95 (m, 2H, CH_2C*H*$_2$CH=), 1.98-1.88 (m, 2H, =CHC*H*$_2$$CH_3$), 1.62-1.51 (m, 2H, C*H*$_2$$CH_2$COO SFA), 1.58 (d, *J*=7.2 Hz, 3H, CHC*H*$_3$), 1.52-1.39 (m, 2H, C*H*$_2$$CH_2$COO EPA), 1.31-1.26 (m, 12H, CH_2), 0.97 (t, *J*=7.5 Hz, 3H, CH_3 EPA), 0.89 (t, *J*=6.8 Hz, 3H, CH_3 SFA) ppm. ^{13}C{H} NMR (101 MHz, $CDCl_3$) δ_C: 173.9 (Nap), 173.4 (SFA), 173.0 (EPA), 157.8, 135.3, 133.9, 132.2, 129.4, 129.0, 128.92, 128.90, 128.7, 128.4, 128.3, 128.3, 128.2, 128.0, 127.2, 127.2, 126.3, 126.1, 119.2, 105.7, 69.5, 62.2, 62.1, 55.4, 45.5, 34.1, 33.2, 32.0, 29.6, 29.4, 29.4, 29.2, 26.5, 25.8, 25.7

(2), 25.7, 25.0, 24.6, 22.8, 20.7, 18.5, 14.4, 14.2 ppm. HRMS (ESI) *m/z*: $[M + Na]^+$ calcd for $C_{47}H_{66}O_7Na$ 765.4701; found, 765.4708.

3.5.13. Synthesis of 3-dodecanoyl-1-[5Z,8Z,11Z,14Z,17Z)-eicosa-5,8,11,14,17-pentaenoyl]-2[(*S*)-2-(6-methoxynaphthalen-2-yl)propanoyl]-*sn*-glycerol, (*S*,*S*')-**11d**

The same procedure was followed as described for (*S*,*S*')-**11a** using 3-dodecanoyl-2-[(*S*)-2-(4-isobutylphenyl)propanoyl]-*sn*-glycerol (*R*,*S*')-**9d** (25 mg, 0.054 mmol), EPA (18 mg, 0.058 mmol), CH_2Cl_2 (2 mL), DMAP (6 mg, 0.050 mmol) and EDCI (11 mg, 0.058 mmol). Purification on a silica gel chromatography using pet. ether/ethyl acetate (8.5:1.5) as eluent afforded the product (*S*,*S*')-**11d** as a pale-yellow oil, in 96% yield (39 mg, 0.052 mmol). $[\alpha]^{20}_D$ = +4.89 (c. 3.8, CH_2Cl_2). ^1H NMR (400 MHz, $CDCl_3$) δ_H: 7.71-7.64 (m, 3H, Nap-1,4,8), 7.38 (dd, *J*=8.5, 1.9 Hz, 1H, Nap-3), 7.13 (dd, *J*=8.9, 2.5 Hz, 1H, Nap-7), 7.10 (d, *J*=2.5 Hz, 1H, Nap-5), 5.44-5.29 (m, 10H, =CH), 5.29-5.21 (m, 1H, CH *sn*-2), 4.30 (dd, *J*=11.9, 4.2 Hz, 1H, CH_2 *sn*-1/3), 4.20-4.15 (m, 2H, CH_2 *sn*-1/3), 4.06 (dd, J=11.9, 6.4 Hz, 1H, CH_2 *sn*-1/3), 3.90 (s, 3H, OCH_3), 3.86 (q, *J*=7.2 Hz, 1H, C*H*CH_3), 2.88-2.72 (m, 8H, =CHC*H*$_2$CH=), 2.28-2.20 (m, 2H, CH_2COO EPA), 2.14-2.04 (m, 2H, CH_2COO SFA), 2.00 (td, *J*=7.7, 5.6 Hz, 2H, CH_2C*H*$_2$CH=), 1.98-1.88 (m, 2H, =CHC*H*$_2$$CH_3$), 1.62-1.51 (m, 2H, C*H*$_2$$CH_2$COO SFA), 1.58 (d, *J*=7.2 Hz, 3H, CHC*H*$_3$), 1.50-1.41 (m, 2H, C*H*$_2$$CH_2$COO EPA), 1.31-1.26 (m, 16H, CH_2), 0.97 (t, *J*=7.5 Hz, 3H, CH_3 EPA), 0.89 (t, *J*=6.8 Hz, 3H, CH_3 SFA) ppm. $^{13}C\{H\}$ NMR (101 MHz, $CDCl_3$) δ_C: 173.9 (Nap), 173.4 (SFA), 173.0 (EPA), 157.8, 135.3, 133.9, 132.2, 129.43, 129.4, 129.0, 129.0, 128.7, 128.4, 128.3, 128.3, 128.2, 128.0, 127.2, 127.2, 126.3, 126.1, 119.2, 105.7, 69.5, 62.2, 62.1, 55.4, 45.5, 34.1, 33.2, 32.1, 29.8, 29.8, 29.6, 29.5, 29.4, 29.2, 26.5, 25.8, 25.7 (2), 25.7, 25.0, 24.6, 22.8, 20.7, 18.5, 14.4, 14.3 ppm. HRMS (ESI) *m/z*: $[M + Na]^+$ calcd for $C_{49}H_{70}O_7Na$ 793.5013; found, 793.5013.

3.5.14. Synthesis of 1-[5Z,8Z,11Z,14Z,17Z)-eicosa-5,8,11,14,17-pentaenoyl]-2[(*S*)-2-(6-methoxynaphthalen-2-yl)propanoyl]-3-tetracanoyl-*sn*-glycerol, (*S*,*S*')-**11e**

The same procedure was followed as described for (*S*,*S*')-**11a** using 2-[(*S*)-2-(4-isobutylphenyl)propanoyl]-3-tetracanoyl-*sn*-glycerol (*R*,*S*')-**9e** (32 mg, 0.062 mmol), EPA (21 mg, 0.068 mmol), CH_2Cl_2 (2.3 mL), DMAP (7 mg, 0.056 mmol) and EDCI (23 mg, 0.068 mmol). Purification on a silica gel chromatography using pet. ether/ethyl acetate (8.5:1.5) as eluent afforded the product (*S*,*S*')-**11e** as a pale-yellow oil, in 90% yield (45 mg, 0.056 mmol). $[\alpha]^{20}_D$ = +5.29 (c. 4.5, CH_2Cl_2). ^1H NMR (400 MHz, $CDCl_3$) δ_H: 7.74-7.62 (m, 3H, Nap-1,4,8), 7.38 (dd, *J*=8.5, 1.9 Hz, 1H, Nap-3), 7.14 (dd, *J*=8.9, 2.5 Hz, 1H, Nap-7), 7.09 (d, *J*=2.5 Hz, 1H, Nap-5), 5.46-5.31 (m, 10H, =CH), 5.30-5.19 (m, 1H, CH *sn*-2), 4.30 (dd, *J*=11.9, 4.2 Hz, 1H, CH_2 *sn*-1/3), 4.20-4.11 (m, 2H, CH_2 *sn*-1/3), 4.06 (dd, J=11.9, 6.4 Hz, 1H, CH_2 *sn*-1/3), 3.90 (s, 3H, OCH_3), 3.86 (q, *J*=7.2 Hz, 1H, C*H*CH_3), 2.89-2.70 (m, 8H, =CHC*H*$_2$CH=), 2.28-2.20 (m, 2H, CH_2COO EPA), 2.14-2.04 (m, 2H, CH_2COO SFA), 2.00 (td, *J*=7.7, 5.6 Hz, 2H, CH_2C*H*$_2$CH=), 1.97-1.89 (m, 2H, =CHC*H*$_2$$CH_3$), 1.63-1.52 (m, 2H, C*H*$_2$$CH_2$COO SFA), 1.58 (d, *J*=7.2 Hz, 3H, CHC*H*$_3$), 1.54 (quint, *J*=7.3 Hz, 2H, C*H*$_2$$CH_2$COO EPA), 1.31-1.26 (m, 20H, CH_2), 0.97 (t, *J*=7.5 Hz, 3H, CH_3 EPA), 0.89 (t, *J*=6.8 Hz, 3H, CH_3 SFA) ppm. $^{13}C\{H\}$ NMR (101 MHz, $CDCl_3$) δ_C: 173.9 (Nap), 173.4 (SFA), 173.0 (EPA), 157.8, 135.3, 133.9, 132.2, 129.4, 129.4, 129.0, 128.9, 128.7, 128.4, 128.32, 128.30, 128.2, 128.0, 127.22, 127.15, 126.3, 126.1, 119.2, 105.7, 69.5, 62.2, 62.1, 55.4, 45.5, 34.1, 33.2, 32.1, 30.1, 29.8, 29.8, 29.8, 29.6, 29.5, 29.4, 29.3, 26.5, 25.8, 25.72 (2), 25.68, 25.0, 24.6, 22.8, 20.7, 18.5, 14.4, 14.3 ppm. HRMS (ESI) *m/z*: $[M + Na]^+$ calcd for $C_{51}H_{74}O_7Na$ 821.5327; found, 821.5327.

3.5.15. Synthesis of 1-[5Z,8Z,11Z,14Z,17Z)-eicosa-5,8,11,14,17-pentaenoyl]-3-hexadecanoyl-2[(*S*)-2-(6-methoxynaphthalen-2-yl)propanoyl]-*sn*-glycerol, (*S*,*S*')-**11f**

The same procedure was followed as described for (*S*,*S*')-**11a** using 3-hexadecanoyl-2-[(*S*)-2-(4-isobutylphenyl)propanoyl]-*sn*-glycerol (*R*,*S*')-**9f** (40 mg, 0.074 mmol), EPA (25 mg, 0.081 mmol), CH_2Cl_2 (3 mL), DMAP (8 mg, 0.067 mmol) and EDCI (16 mg, 0.081 mmol). Purification on a silica gel chromatography using pet. ether/ethyl acetate (8.5:1.5) as eluent afforded the product (*S*,*S*')-**11f** as a pale-yellow oil, in 91%

yield (51 mg, 0.067 mmol). [α]$^{20}_{D}$ = +5.44 (c. 5.0, CH_2Cl_2). ^{1}H NMR (400 MHz, $CDCl_3$) δ_H: 7.71-7.64 (m, 3H, Nap-1,4,8), 7.38 (dd, *J*=8.5, 1.9 Hz, 1H, Nap-3), 7.13 (dd, *J*=8.9, 2.5 Hz, 1H, Nap-7), 7.09 (d, *J*=2.5 Hz, 1H, Nap-5), 5.44-5.23 (m, 10H, =CH), 5.23-5.12 (m, 1H, CH *sn*-2), 4.30 (dd, *J*=11.9, 4.2 Hz, 1H, CH_2 *sn*-1/3), 4.18 (dd, *J*=11.9, 4.3 Hz, 1H, CH_2 *sn*-1/3), 4.14 (dd, *J*=11.9, 6.1 Hz, 1H, CH_2 *sn*-1/3), 4.07 (dd, J=11.9, 6.5 Hz, 1H, CH_2 *sn*-1/3), 3.90 (s, 3H, OCH_3), 3.86 (q, *J*=7.2 Hz, 1H, C*H*CH_3), 2.90-2.73 (m, 8H, =CHC*H*$_2$CH=), 2.24 (t, *J*=7.2 Hz, 2H, CH_2COO EPA), 2.20-2.13 (m, 2H, CH_2COO SFA), 2.14-1.97 (m, 4H, CH_2C*H*$_2$CH= and =CHC*H*$_2CH_3$), 1.58 (d, *J*=7.2 Hz, 3H, CHC*H*$_3$), 1.56-1.49 (m, 2H, C*H*$_2CH_2$COO SFA), 1.46 (quint, *J*=7.3 Hz, 2H, C*H*$_2CH_2$COO EPA), 1.37-1.20 (m, 24H, CH_2), 0.97 (t, *J*=7.5 Hz, 3H, CH_3 EPA), 0.89 (t, *J*=6.8 Hz, 3H, CH_3 SFA) ppm. ^{13}C{H} NMR (101 MHz, $CDCl_3$) δ_C: 173.9 (Nap), 173.4 (SFA), 173.0 (EPA), 157.8, 135.3, 133.9, 132.12, 129.4, 129.03, 128.95, 128.9, 128.7, 128.4, 128.32, 128.29, 128.2, 128.0, 127.2, 127.1, 126.3, 126.1, 119.2, 105.7, 69.5, 62.2 (2), 62.1, 55.4, 45.5, 34.1, 33.2, 32.1, 29.83 (2), 29.80 (2), 29.76 (2), 29.6 (2), 29.5, 29.4, 29.3, 25.8 (2), 25.7 (2), 25.0, 23.0, 22.6, 20.8, 18.7, 14.4, 14.3 ppm. HRMS (ESI) *m*/*z*: [M + Na]$^+$ calcd for $C_{53}H_{78}O_7Na$ 849.5640; found, 849.5600.

3.5.16. Synthesis of 3-[5Z,8Z,11Z,14Z,17Z)-eicosa-5,8,11,14,17-pentaenoyl]-2[(*S*)-2-(6-methoxynaphthalen-2-yl)propanoyl]-1-octanoyl-*sn*-glycerol, (*R*,*S*')-**11b**

The same procedure was followed as described for (*R*,*S*')-**11a** using 2-[(*S*)-2-(4-isobutylphenyl)propanoyl]-1-octanoyl-*sn*-glycerol (*R*,*S*')-**9b** (35 mg, 0.081 mmol), EPA (27 mg, 0.089 mmol), CH_2Cl_2 (4 mL), DMAP (9 mg, 0.074 mmol) and EDCI (17 mg, 0.089 mmol). Purification on a silica gel chromatography using pet. ether/ethyl acetate (4:1) as eluent afforded the product (*R*,*S*')-**11b** as a yellow oil, in 94% yield (54 mg, 0.076 mmol). [α]$^{20}_{D}$ = +4.97 (c. 3.0, CH_2Cl_2). IR (NaCl, ν_{max} / cm^{-1}): 3012 (s), 2958 (vs), 2931 (vs), 2856 (vs), 1743 (vs), 1634 (s), 1607 (s). ^{1}H NMR (400 MHz, $CDCl_3$) δ_H: 7.76-7.54 (m, 3H, Nap-1,4,8), 7.38 (dd, *J*=8.5, 1.9 Hz, 1H, Nap-3), 7.13 (dd, *J*=8.9, 2.6 Hz, 1H, Nap-7), 7.09 (d, *J*=2.6 Hz, 1H, Nap-5), 5.47-5.30 (m, 10H, =CH), 5.30-5.19 (m, 1H, CH *sn*-2), 4.30 (dd, *J*=11.9, 4.2 Hz, 1H, CH_2 *sn*-1/3), 4.22-4.11 (m, 2H, CH_2 *sn*-1/3), 4.06 (dd, J=11.9, 6.4 Hz, 1H, CH_2 *sn*-1/3), 3.91 (s, 3H, OCH_3), 3.86 (q, *J*=7.1 Hz, 1H, C*H*CH_3), 2.96-2.73 (m, 8H, =CHC*H*$_2$CH=), 2.31-2.23 (m, 2H, CH_2COO EPA), 2.13-2.01 (m, 2H, CH_2COO SFA), 2.01-1.86 (m, 4H, CH_2C*H*$_2$CH= and =CHC*H*$_2CH_3$), 1.60-1.55 (m, 2H, C*H*$_2CH_2$COO SFA), 1.57 (d, *J*=7.1 Hz, CHC*H*$_3$), 1.37 (quint, *J*= 7.6 Hz, 2H, C*H*$_2CH_2$COO EPA), 1.31-1.08 (m, 8H, CH_2), 0.97 (t, *J*=7.5 Hz, 3H, CH_3 EPA), 0.89 (t, *J*=6.8 Hz, 3H, CH_3 SFA) ppm. ^{13}C{H} NMR (101 MHz, $CDCl_3$) δ_C: 173.9 (Nap), 173.3 (SFA), 173.1 (EPA), 157.9, 135.3, 133.9, 132.2, 129.4, 129.08, 129.05, 129.0, 128.7, 128.4, 128.34, 128.31, 128.2, 128.0, 127.24, 127.17, 126.3, 126.1, 119.2, 105.7, 69.5, 62.29, 62.0, 55.4, 45.5, 33.9, 33.5, 32.1, 29.4, 29.2, 26.7, 25.8 (2), 25.7 (2), 24.8, 24.8, 22.8, 20.7, 18.5, 14.4, 14.3 ppm. HRMS (ESI) *m*/*z*: [M + Na]$^+$ calcd for $C_{45}H_{62}O_7Na$ 737.4388; found, 737.4379.

3.5.17. Synthesis of 1-decanoyl-3-[5Z,8Z,11Z,14Z,17Z)-eicosa-5,8,11,14,17-pentaenoyl]-2[(*S*)-2-(6-methoxy-naphthalen-2-yl)propanoyl]-*sn*-glycerol, (*R*,*S*')-**11c**

The same procedure was followed as described for (*R*,*S*')-**11a** using 1-decanoyl-2-[(*S*)-2-(4-isobutylphenyl)propanoyl]-*sn*-glycerol (*S*,*S*')-**9c** (23 mg, 0.052 mmol), EPA (24 mg, 0.079 mmol), CH_2Cl_2 (5 mL), DMAP (11 mg, 0.087 mmol) and EDCI (20 mg, 0.105 mmol). Purification on a silica gel chromatography using pet. ether/ethyl acetate (8.5:1.5) as eluent afforded the product (*R*,*S*')-**11c** as a yellow oil, in 86% yield (34 mg, 0.045 mmol). [α]$^{20}_{D}$ = +8.76 (c. 2.1, CH_2Cl_2). IR (NaCl, ν_{max} / cm^{-1}): 3012 (vs), 2958 (vs), 2928 (vs), 2855 (vs), 1743 (vs), 1634 (s), 1607 (s). ^{1}H NMR (400 MHz, $CDCl_3$) δ_H: 7.76-7.54 (m, 3H, Nap-1,4,8), 7.38 (dd, *J*=8.5, 1.9 Hz, 1H, Nap-3), 7.13 (dd, *J*=8.9, 2.6 Hz, 1H, Nap-7), 7.09 (d, *J*=2.6 Hz, 1H, Nap-5), 5.47-5.30 (m, 10H, =CH), 5.30-5.19 (m, 1H, CH *sn*-2), 4.30 (dd, *J*=11.9, 4.2 Hz, 1H, CH_2 *sn*-1/3), 4.22-4.11 (m, 2H, CH_2 *sn*-1/3), 4.06 (dd, J=11.9, 6.4 Hz, 1H, CH_2 *sn*-1/3), 3.91 (s, 3H, OCH_3), 3.86 (q, *J*=7.1 Hz, 1H, C*H*CH_3), 2.96-2.73 (m, 8H, =CHC*H*$_2$CH=), 2.31-2.23 (m, 2H, CH_2COO EPA), 2.13-2.02 (m, 2H, CH_2COO SFA), 2.02-1.86 (m, 4H, CH_2C*H*$_2$CH= and =CHC*H*$_2CH_3$), 1.60-1.55 (m, 2H, C*H*$_2CH_2$COO SFA), 1.57 (d, *J*=7.1 Hz, CHC*H*$_3$), 1.39 (quint, *J*= 7.6 Hz, 2H, C*H*$_2CH_2$COO EPA), 1.31-1.08 (m, 12H, CH_2), 0.97 (t, *J*=7.5 Hz, 3H, CH_3

EPA), 0.89 (t, *J*=6.8 Hz, 3H, CH_3 SFA) ppm. $^{13}C\{H\}$ NMR (101 MHz, $CDCl_3$) δ_C: 173.9 (Nap), 173.3 (SFA), 173.1 (EPA), 157.9, 135.3, 133.9, 132.2, 129.4, 129.08, 129.05, 129.0, 128.7, 128.4, 128.4, 128.3, 128.2, 128.0, 127.24, 127.17, 126.3, 126.1, 119.2, 105.7, 69.5, 62.3, 62.0, 55.4, 45.5, 33.9, 33.5, 32.1, 29.6, 29.5, 29.4, 29.2, 26.7, 25.8 (2), 25.7 (2), 24.80, 24.76, 22.8, 20.7, 18.5, 14.4, 14.3 ppm. HRMS (ESI) *m*/*z*: $[M + Na]^+$ calcd for $C_{47}H_{66}O_7Na$ 765.4701; found, 765.4620.

3.5.18. Synthesis of 1-dodecanoyl-3-[5Z,8Z,11Z,14Z,17Z)-eicosa-5,8,11,14,17-pentaenoyl]-2[(*S*)-2-(6-methoxynaphthalen-2-yl)propanoyl]-*sn*-glycerol, (*R*,*S*')-**11d**

The same procedure was followed as described for (*R*,*S*')-**11a** using 1-dodecanoyl-2-[(*S*)-2-(4-isobutylphenyl)propanoyl]-*sn*-glycerol (*S*,*S*')-**9d** (19 mg, 0.038 mmol), EPA (13 mg, 0.041 mmol), CH_2Cl_2 (1.6 mL), DMAP (5 mg, 0.041 mmol) and EDCI (11 mg, 0.056 mmol). Purification on a silica gel chromatography using pet. ether/ethyl acetate (8.5:1.5) as eluent afforded the product (*R*,*S*')-**11d** as a pale-yellow oil, in 74% yield (22 mg, 0.028 mmol). $[\alpha]^{20}_D$ = +5.20 (c. 1.0, CH_2Cl_2). IR (NaCl, ν_{max} / cm^{-1}): 3012 (vs), 2926 (vs), 2854 (vs), 1743 (vs), 1634 (s), 1607 (s). ^1H NMR (400 MHz, $CDCl_3$) δ_H: 7.77-7.54 (m, 3H, Nap-1,4,8), 7.38 (dd, *J*=8.5, 1.9 Hz, 1H, Nap-3), 7.13 (dd, *J*=8.9, 2.6 Hz, 1H, Nap-7), 7.09 (d, *J*=2.6 Hz, 1H, Nap-5), 5.47-5.30 (m, 10H, =CH), 5.30-5.19 (m, 1H, CH *sn*-2), 4.30 (dd, *J*=11.9, 4.2 Hz, 1H, CH_2 *sn*-1/3), 4.22-4.11 (m, 2H, CH_2 *sn*-1/3), 4.06 (dd, J=11.9, 6.4 Hz, 1H, CH_2 *sn*-1/3), 3.91 (s, 3H, OCH_3), 3.86 (q, *J*=7.1 Hz, 1H, C*H*CH_3), 2.96-2.73 (m, 8H, =CHC*H*$_2$CH=), 2.30-2.23 (m, 2H, CH_2COO EPA), 2.13-2.01 (m, 2H, CH_2COO SFA), 2.01-1.86 (m, 4H, CH_2C*H*$_2$CH= and =CHC*H*$_2$$CH_3$), 1.60-1.55 (m, 2H, C*H*$_2$$CH_2$COO SFA), 1.57 (d, *J*=7.1 Hz, CHC*H*$_3$), 1.37 (quint, *J*= 7.6 Hz, 2H, C*H*$_2$$CH_2$COO EPA), 1.31-1.08 (m, 16H, CH_2), 0.97 (t, *J*=7.5 Hz, 3H, CH_3 EPA), 0.89 (t, *J*=6.8 Hz, 3H, CH_3 SFA) ppm. $^{13}C\{H\}$ NMR (101 MHz, $CDCl_3$) δ_C: 173.9 (Nap), 173.3 (SFA), 173.1 (EPA), 157.9, 135.3, 133.9, 132.2, 129.39, 129.08, 129.05, 129.0, 128.7, 128.44, 128.35, 128.3, 128.2, 128.0, 127.24, 127.17, 126.3, 126.1, 119.2, 105.7, 69.5, 62.3, 62.0, 55.4, 45.5, 33.9, 33.5, 32.1, 29.8 (2), 29.6, 29.5, 29.4, 29.2, 26.7, 25.8 (2), 25.7 (2), 24.80, 24.76, 22.8, 20.7, 18.5, 14.4, 14.3 ppm. HRMS (ESI) *m*/*z*: $[M + Na]^+$ calcd for $C_{49}H_{70}O_7Na$ 793.5013; found, 793.5013.

3.5.19. Synthesis of 3-[5Z,8Z,11Z,14Z,17Z)-eicosa-5,8,11,14,17-pentaenoyl]-2[(*S*)-2-(6-methoxynaphthalen-2-yl)propanoyl]-1-tetracanoyl-*sn*-glycerol, (*R*,*S*')-**11e**

The same procedure was followed as described for (*R*,*S*')-**11a** using 2-[(*S*)-2-(4-isobutylphenyl)propanoyl]-1-tetracanoyl-*sn*-glycerol (*S*,*S*')-**9e** (13 mg, 0.025 mmol), EPA (8 mg, 0.027 mmol), CH_2Cl_2 (1 mL), DMAP (3 mg, 0.024 mmol) and EDCI (7 mg, 0.037 mmol). Purification on a silica gel chromatography using pet. ether/ethyl acetate (4:1) as eluent afforded the product (*R*,*S*')-**11e** as a pale-yellow oil, in 90% yield (15 mg, 0.019 mmol). $[\alpha]^{20}_D$ = +6.30 (c. 1.5, CH_2Cl_2). IR (NaCl, ν_{max} / cm^{-1}): 3012 (s), 2951 (vs), 2926 (vs), 2854 (vs), 1743 (vs), 1633 (s), 1607 (s). ^1H NMR (400 MHz, $CDCl_3$) δ_H: 7.71-7.62 (m, 3H, Nap-1,4,8), 7.38 (dd, *J*=8.5, 1.9 Hz, 1H, Nap-3), 7.13 (dd, *J*=8.9, 2.6 Hz, 1H, Nap-7), 7.09 (d, *J*=2.6 Hz, 1H, Nap-5), 5.44-5.29 (m, 10H, =CH), 5.30-5.12 (m, 1H, CH *sn*-2), 4.29 (dd, *J*=11.9, 4.2 Hz, 1H, CH_2 *sn*-1/3), 4.19-4.11 (m, 2H, CH_2 *sn*-1/3), 4.06 (dd, J=11.9, 6.4 Hz, 1H, CH_2 *sn*-1/3), 3.91 (s, 3H, OCH_3), 3.86 (q, *J*=7.1 Hz, 1H, C*H*CH_3), 2.88-2.74 (m, 8H, =CHC*H*$_2$CH=), 2.30-2.23 (m, 2H, CH_2COO EPA), 2.13-2.02 (m, 2H, CH_2COO SFA), 2.02-1.89 (m, 4H, CH_2C*H*$_2$CH= and =CHC*H*$_2$$CH_3$), 1.60-1.55 (m, 2H, C*H*$_2$$CH_2$COO SFA), 1.57 (d, *J*=7.1 Hz, CHC*H*$_3$), 1.37 (quint, *J*= 7.6 Hz, 2H, C*H*$_2$$CH_2$COO EPA), 1.32-1.06 (m, 20H, CH_2), 0.97 (t, *J*=7.5 Hz, 3H, CH_3 EPA), 0.89 (t, *J*=6.8 Hz, 3H, CH_3 SFA) ppm. $^{13}C\{H\}$ NMR (101 MHz, $CDCl_3$) δ_C: 173.9 (Nap), 173.3 (SFA), 173.1 (EPA), 157.9, 135.4, 133.9, 132.2, 129.40, 129.09, 129.05, 129.0, 128.7, 128.44, 128.35, 128.3, 128.2, 128.0, 127.24, 127.17, 126.3, 126.1, 119.2, 105.7, 69.5, 62.30, 62.0, 55.4, 45.5, 33.9, 33.5, 32.1, 30.1 (2), 29.9, 29.82, 29.78, 29.6, 29.5, 29.4, 29.2, 26.7 (2), 25.7 (2), 24.80, 24.77, 22.9, 20.7, 18.5, 14.4, 14.3 ppm. HRMS (ESI) *m*/*z*: $[M + Na]^+$ calcd for $C_{51}H_{74}O_7Na$ 821.5327; found, 821.5302.

3.5.20. Synthesis of 3-[5Z,8Z,11Z,14Z,17Z)-eicosa-5,8,11,14,17-pentaenoyl]-1-hexadecanoyl-2[(*S*)-2-(6-methoxynaphthalen-2-yl)propanoyl]-*sn*-glycerol, (*R*,*S*')-**11f**

The same procedure was followed as described for (*R*,*S*')-**11f** using 1-hexadecanoyl-2-[(*S*)-2-(4-isobutylphenyl)propanoyl]-*sn*-glycerol (*S*,*S*')-**9f** (10 mg, 0.018 mmol), EPA (6 mg, 0.020 mmol), CH_2Cl_2 (1 mL), DMAP (2 mg, 0.016 mmol) and EDCI (4 mg, 0.020 mmol). Purification on a silica gel chromatography using pet. ether/ethyl acetate (9:1) as eluent afforded the product (*R*,*S*')-**11f** as a pale-yellow oil, in 89% yield (13 mg, 0.016 mmol). $[\alpha]^{20}_D$ = +6.23 (c. 1.3, CH_2Cl_2). IR (NaCl, ν_{max} / cm^{-1}): 3012 (s), 2962 (vs), 2925 (vs), 1741 (vs), 1635 (s), 1607 (vs). ^{1}H NMR (400 MHz, $CDCl_3$) δ_H: 7.72-7.60 (m, 3H, Nap-1,4,8), 7.38 (dd, *J*=8.5, 1.9 Hz, 1H, Nap-3), 7.13 (dd, *J*=8.9, 2.6 Hz, 1H, Nap-7), 7.09 (d, *J*=2.6 Hz, 1H, Nap-5), 5.46-5.28 (m, 10H, =CH), 5.30-5.12 (m, 1H, CH *sn*-2), 4.29 (dd, *J*=11.9, 4.2 Hz, 1H, CH_2 *sn*-1/3), 4.17-4.09 (m, 2H, CH_2 *sn*-1/3), 4.05 (dd, J=11.9, 6.4 Hz, 1H, CH_2 *sn*-1/3), 3.90 (s, 3H, OCH_3), 3.86 (q, *J*=7.1 Hz, 1H, C*H*CH_3), 2.90-2.73 (m, 8H, =CHC*H*$_2$CH=), 2.31-2.22 (m, 2H, CH_2COO EPA), 2.14-2.08 (m, 2H, CH_2COO SFA), 2.04-1.86 (m, 4H, CH_2C*H*$_2$CH= and =CHC*H*$_2$$CH_3$), 1.60-1.56 (m, 2H, C*H*$_2$$CH_2$COO SFA), 1.57 (d, *J*=7.1 Hz, CHC*H*$_3$), 1.35 (quint, *J*= 7.5 Hz, 2H, C*H*$_2$$CH_2$COO EPA), 1.18-1.06 (m, 24H, CH_2), 0.97 (t, *J*=7.5 Hz, 3H, CH_3 EPA), 0.89 (t, *J*=6.8 Hz, 3H, CH_3 SFA) ppm. ^{13}C{H} NMR (101 MHz, $CDCl_3$) δ_C: 173.9 (Nap), 173.3 (SFA), 173.1 (EPA), 157.8, 135.3, 133.9, 132.2, 129.3, 129.1, 129.04, 129.0, 128.7, 128.4, 128.34, 128.31, 128.2, 128.0, 127.23, 127.15, 126.3, 126.1, 119.2, 105.7, 69.5, 62.3, 62.0, 55.4, 45.5, 33.9, 33.5, 32.1, 30.1 (2), 29.9 (2), 29.81, 29.77, 29.6, 29.5, 29.4, 29.2, 26.6, 25.8 (2), 25.7 (2), 24.78, 24.75, 22.8, 20.7, 18.5, 14.4, 14.3 ppm. HRMS (ESI) *m*/*z*: $[M + Na]^+$ calcd for $C_{53}H_{78}O_7Na$ 849.5640; found, 849.5638.

3.6. *Coupling of DHA: Synthesis of (S,S')-**12b-f**, (R,S')-**12b-f**, (S,S')-**13b-f** and (R,S')-**13b-f***

3.6.1. Synthesis of 1-[4*Z*,7*Z*,10*Z*,13*Z*,16*Z*,19*Z*)-docosa-4,7,10,13,16,19-hexaenoyl]-2-[(*S*)-2-(4-isobutylphenyl)propanoyl]-3-octanoyl-*sn*-glycerol, (*S*,*S*')-**12b**

The same procedure was followed as described for (*S*,*S*')-**12a** using 2-[(*S*)-2-(4-isobutylphenyl)propanoyl]-3-octanoyl-*sn*-glycerol (*R*,*S*')-**8b** (25 mg, 0.061 mmol), DHA (22 mg, 0.067 mmol), CH_2Cl_2 (3 mL), DMAP (7 mg, 0.059 mmol) and EDCI (14 mg, 0.071 mmol). Purification on a silica gel chromatography using pet. ether/ethyl acetate (4:1) as eluent afforded the product (*S*,*S*')-**12b** as a pale-yellow oil, in 86% yield (38 mg, 0.052 mmol). $[\alpha]^{20}_D$ = +4.44 (c. 2.5, CH_2Cl_2). ^{1}H NMR (400 MHz, $CDCl_3$) δ_H: 7.18 (d, *J*=8.1 Hz, 2H, Ibu-2,6), 7.07 (d, *J*=8.1 Hz, 2H, Ibu-3,5), 5.45-5.22 (m, 13H, =CH and CH *sn*-2), 4.30 (dd, *J*=11.9, 4.2 Hz, 1H, CH_2 *sn*-1/3), 4.18 (dd, *J*=11.9, 4.5 Hz, 1H, CH_2 *sn*-1/3), 4.14 (dd, J=11.9, 6.0 Hz, 1H, CH_2 *sn*-1/3), 4.06 (dd, J=11.9, 6.4 Hz, 1H, CH_2 *sn*-1/3), 3.70 (q, *J*=7.2 Hz, 1H, C*H*CH_3), 2.89-2.77 (m, 10H, =CHC*H*$_2$CH=), 2.43 (d, *J*=7.2 Hz, 2H, C*H*$_2$CH(CH_3)$_2$), 2.30-2.24 (m 6H, $CH_2$$CH_2$COO DHA and CH_2COO SFA), 2.12-2.08 (m, 2H, =CHC*H*$_2$$CH_3$), 1.83 (nonet, *J*=6.9 Hz, 1H, C*H*(CH_3)$_2$), 1.64-1.54 (m, 2H, C*H*$_2$$CH_2$COO SFA), 1.49 (d, *J*=7.2 Hz, 3H, CHC*H*$_3$), 1.33-1.20 (m, 8H, CH_2), 0.97 (t, *J*=7.5 Hz, 3H, CH_3 DHA), 0.90 (t, *J*=6.4 Hz, 3H, CH_3 SFA), 0.89 (d, *J*=6.6 Hz, 6H, CH(C*H*$_3$)$_2$) ppm. ^{13}C{H} NMR (101 MHz, $CDCl_3$) δ_C: 173.8 (Ibu), 173.2 (SFA), 172.4 (DHA), 140.6, 137.3, 132.0, 129.4 (2), 129.3, 128.6, 128.30, 128.27, 128.25, 128.09, 128.07, 128.0, 127.9, 127.7 (2), 127.1, 127.0, 69.2, 62.1, 62.0, 45.04, 45.02, 34.0, 33.7, 31.7, 30.2, 29.1, 28.9, 25.7 (2), 25.6, 25.5 (2), 24.8, 22.6, 22.5 (2), 22.4, 20.6, 18.4, 14.3, 14.1 ppm. HRMS (ESI) *m*/*z*: $[M + Na]^+$ calcd for $C_{46}H_{68}O_6Na$ 739.4908; found, 739.4896.

3.6.2. Synthesis of 1-[4*Z*,7*Z*,10*Z*,13*Z*,16*Z*,19*Z*)-docosa-4,7,10,13,16,19-hexaenoyl]-3-decanoyl-2-[(*S*)-2-(4-isobutylphenyl)propanoyl]-*sn*-glycerol, (*S*,*S*')-**12c**

The same procedure was followed as described for (*S*,*S*')-**12a** using 3-decanoyl-2-[(*S*)-2-(4-isobutylphenyl)propanoyl]-*sn*-glycerol (*R*,*S*')-**8c** (40 mg, 0.092 mmol), DHA (33 mg, 0.101 mmol), CH_2Cl_2 (3.5 mL), DMAP (10 mg, 0.083 mmol) and EDCI (19 mg, 0.100 mmol). Purification on a silica gel chromatography using pet. ether/ethyl acetate (8.5:1.5) as eluent afforded the product (*S*,*S*')-**12c** as a yellow oil, in 95% yield (65 mg, 0.087 mmol). $[\alpha]^{20}_D$ = +4.05 (c. 6.5, CH_2Cl_2). ^{1}H NMR (400 MHz, $CDCl_3$) δ_H: 7.18 (d, *J*=8.1 Hz, 2H, Ibu-2,6), 7.07 (d, *J*=8.1 Hz, 2H, Ibu-3,5), 5.41-5.32 (m, 12H, =CH), 5.28-5.22 (m, 1H, CH *sn*-2), 4.30 (dd, *J*=11.9, 4.2 Hz, 1H, CH_2 *sn*-1/3), 4.19 (dd, *J*=11.9, 4.2 Hz, 1H, CH_2 *sn*-1/3), 4.13 (dd, J=11.9, 6.0 Hz,

1H, CH_2 *sn*-1/3), 4.08 (dd, J=11.9, 6.4 Hz, 1H, CH_2 *sn*-1/3), 3.70 (q, *J*=7.2 Hz, 1H, C*H*CH_3), 2.86-2.80 (m, 10H, =CHC*H*$_2$CH=), 2.43 (d, *J*=7.2 Hz, 2H, C*H*$_2$CH(CH_3)$_2$), 2.30-2.24 (m 6H, CH_2CH_2COO DHA and CH_2COO SFA), 2.08-2.06 (m, 2H, =CHC*H*$_2$$CH_3$), 1.83 (nonet, *J*=6.9 Hz, 1H, C*H*(CH_3)$_2$), 1.62-1.56 (m, 2H, C*H*$_2$$CH_2$COO SFA), 1.49 (d, *J*=7.2 Hz, 3H, CHC*H*$_3$), 1.33-1.20 (m, 12H, CH_2), 0.97 (t, *J*=7.5 Hz, 3H, CH_3 DHA), 0.90 (t, *J*=6.4 Hz, 3H, CH_3 SFA), 0.89 (d, *J*=6.6 Hz, 6H, CH(C*H*$_3$)$_2$) ppm. ^{13}C{H} NMR (101 MHz, $CDCl_3$) δ_C: 173.8 (Ibu), 173.2 (SFA), 172.4 (DHA), 140.6, 137.3, 132.0, 129.4 (2), 129.3, 128.6, 128.30, 128.29, 128.26, 128.08, 128.06, 127.91, 127.85, 127.7 (2), 127.1, 127.0, 69.2, 62.1, 62.0, 45.0, 45.0, 34.0, 33.7, 31.9, 30.2 (2), 29.4, 29.3, 29.1, 25.62 (2), 25.57, 25.5 (2), 24.8, 22.7, 22.5 (2), 22.4, 20.5, 18.4, 14.3, 14.1 ppm. HRMS (ESI) *m*/*z*: $[M + Na]^+$ calcd for $C_{48}H_{72}O_6Na$ 767.5221; found, 767.5224.

3.6.3. Synthesis of 1-[4Z,7Z,10Z,13Z,16Z,19Z)-docosa-4,7,10,13,16,19-hexaenoyl]-3-dodecanoyl-2-[(*S*)-2-(4-isobutylphenyl)propanoyl]-*sn*-glycerol, (*S*,*S*')-**12d**

The same procedure was followed as described for (*S*,*S*')-**12a** using 3-dodecanoyl-2-[(*S*)-2-(4-isobutylphenyl)propanoyl]-*sn*-glycerol (*R*,*S*')-**8d** (15 mg, 0.034 mmol), DHA (29 mg, 0.057 mmol), CH_2Cl_2 (3 mL), DMAP (9 mg, 0.071 mmol) and EDCI (12 mg, 0.088 mmol). Purification on a silica gel chromatography using pet. ether/ethyl acetate (8.5:1.5) as eluent afforded the product (*S*,*S*')-**12d** as a yellow oil, in 89% yield (23 mg, 0.030 mmol). $[\alpha]^{20}_D$ = +5.30 (c. 2.0, CH_2Cl_2). ^{1}H NMR (400 MHz, $CDCl_3$) δ_H: 7.18 (d, *J*=8.1 Hz, 2H, Ibu-2,6), 7.07 (d, *J*=8.1 Hz, 2H, Ibu-3,5), 5.41-5.25 (m, 13H, =CH and CH *sn*-2), 4.30 (dd, *J*=11.9, 4.2 Hz, 1H, CH_2 *sn*-1/3), 4.19 (dd, *J*=11.9, 4.2 Hz, 1H, CH_2 *sn*-1/3), 4.13 (dd, J=11.9, 5.8 Hz, 1H, CH_2 *sn*-1/3), 4.08 (dd, J=11.9, 6.3 Hz, 1H, CH_2 *sn*-1/3), 3.70 (q, *J*=7.2 Hz, 1H, C*H*CH_3), 2.86-2.80 (m, 10H, =CHC*H*$_2$CH=), 2.43 (d, *J*=7.2 Hz, 2H, C*H*$_2$CH(CH_3)$_2$), 2.30-2.24 (m 6H, CH_2CH_2COO DHA and CH_2COO SFA), 2.08-2.06 (m, 2H, =CHC*H*$_2$$CH_3$), 1.83 (nonet, *J*=6.9 Hz, 1H, C*H*(CH_3)$_2$), 1.59-1.48 (m, 2H, C*H*$_2$$CH_2$COO SFA), 1.49 (d, *J*=7.2 Hz, 3H, CHC*H*$_3$), 1.32-1.26 (m, 16H, CH_2), 0.97 (t, *J*=7.5 Hz, 3H, CH_3 DHA), 0.89 (d, *J*=6.6 Hz, 6H, CH(C*H*$_3$)$_2$), 0.88 (t, *J*=6.4 Hz, 3H, CH_3 SFA) ppm. ^{13}C{H} NMR (101 MHz, $CDCl_3$) δ_C: 173.9 (Ibu), 173.4 (SFA), 172.6 (DHA), 140.8, 137.4, 132.2, 129.5 (2), 129.4, 128.7, 128.5, 128.43, 128.40, 128.3, 128.23, 128.16, 128.0, 127.8 (2), 127.3, 127.2, 69.3, 62.24, 62.17, 45.19, 45.18, 34.2, 33.8, 32.1, 30.3, 29.9, 29.8, 29.6, 29.5, 29.4, 29.2, 25.8 (2), 25.74, 25.70 (2), 25.0, 22.8, 22.7 (2), 22.5, 20.7, 18.5, 14.4, 14.3 ppm. HRMS (ESI) *m*/*z*: $[M + Na]^+$ calcd for $C_{50}H_{76}O_6Na$ 795.5534; found, 795.5539.

3.6.4. Synthesis of 1-[4Z,7Z,10Z,13Z,16Z,19Z)-docosa-4,7,10,13,16,19-hexaenoyl]-2-[(*S*)-2-(4-isobutylphenyl)propanoyl]-3-tetradecanoyl-*sn*-glycerol, (*S*,*S*')-**12e**

The same procedure was followed as described for (*S*,*S*')-**12a** using 2-[(*S*)-2-(4-isobutylphenyl)propanoyl]-3-tetradecanoyl-*sn*-glycerol (*R*,*S*')-**8e** (19 mg, 0.039 mmol), DHA (25 mg, 0.076 mmol), CH_2Cl_2 (2.6 mL), DMAP (8 mg, 0.062 mmol) and EDCI (16 mg, 0.075 mmol). Purification on a silica gel chromatography using pet. ether/ethyl acetate (8.5:1.5) as eluent afforded the product (*S*,*S*')-**12e** as a yellow oil, in 82% yield (26 mg, 0.032 mmol). $[\alpha]^{20}_D$ = +7.54 (c. 2.6, CH_2Cl_2). ^{1}H NMR (400 MHz, $CDCl_3$) δ_H: 7.18 (d, *J*=8.1 Hz, 2H, Ibu-2,6), 7.07 (d, *J*=8.1 Hz, 2H, Ibu-3,5), 5.41-5.32 (m, 12H, =CH), 5.32-5.22 (m, 1H, CH *sn*-2), 4.30 (dd, *J*=11.9, 4.2 Hz, 1H, CH_2 *sn*-1/3), 4.19 (dd, *J*=11.9, 4.2 Hz, 1H, CH_2 *sn*-1/3), 4.13 (dd, J=11.9, 5.8 Hz, 1H, CH_2 *sn*-1/3), 4.08 (dd, J=11.9, 6.3 Hz, 1H, CH_2 *sn*-1/3), 3.70 (q, *J*=7.2 Hz, 1H, C*H*CH_3), 2.86-2.80 (m, 10H, =CHC*H*$_2$CH=), 2.43 (d, *J*=7.2 Hz, 2H, C*H*$_2$CH(CH_3)$_2$), 2.31-2.22 (m 6H, CH_2CH_2COO DHA and CH_2COO SFA), 2.08-2.06 (m, 2H, =CHC*H*$_2$$CH_3$), 1.83 (nonet, *J*=6.9 Hz, 1H, C*H*(CH_3)$_2$), 1.59-1.48 (m, 2H, C*H*$_2$$CH_2$COO SFA), 1.49 (d, *J*=7.2 Hz, 3H, CHC*H*$_3$), 1.32-1.26 (m, 20H, CH_2), 0.97 (t, *J*=7.5 Hz, 3H, CH_3 DHA), 0.89 (d, *J*=6.6 Hz, 6H, CH(C*H*$_3$)$_2$), 0.88 (t, *J*=6.4 Hz, 3H, CH_3 SFA) ppm. ^{13}C{H} NMR (101 MHz, $CDCl_3$) δ_C: 173.9 (Ibu), 173.4 (SFA), 172.6 (DHA), 140.8, 137.4, 132.2, 129.5 (2), 129.4, 128.7, 128.5, 128.42, 128.40, 128.3, 128.22, 128.16, 128.0, 127.8 (2), 127.3, 127.2, 69.3, 62.24, 62.17, 45.19, 45.18, 34.2, 33.8, 32.1, 30.3, 30.1 (2), 29.84, 29.81, 29.78, 29.6, 29.5, 29.4, 29.3 (2), 25.8, 25.7 (2), 25.0, 22.8, 22.7 (2), 22.5, 20.7, 18.5, 14.4, 14.3 ppm. HRMS (ESI) *m*/*z*: $[M + Na]^+$ calcd for $C_{52}H_{80}O_6Na$ 823.5847; found, 823.5840.

3.6.5. Synthesis of 1-[4Z,7Z,10Z,13Z,16Z,19Z)-docosa-4,7,10,13,16,19-hexaenoyl]-2-[(*S*)-3-hexadecanoyl-2-(4-isobutylphenyl)propanoyl]-*sn*-glycerol, (*S*,*S*')-**12f**

The same procedure was followed as described for (*S*,*S*')-**12a** using 3-hexadecanoyl-2-[(*S*)-2-(4-isobutylphenyl)propanoyl]-*sn*-glycerol (*R*,*S*')-**8f** (42 mg, 0.081 mmol), DHA (30 mg, 0.089 mmol), CH_2Cl_2 (3 mL), DMAP (9 mg, 0.073 mmol) and EDCI (17 mg, 0.088 mmol). Purification on a silica gel chromatography using pet. ether/ethyl acetate (8.5:1.5) as eluent afforded the product (*S*,*S*')-**12f** as a yellow oil, in 85% yield (57 mg, 0.069 mmol). $[\alpha]^{20}_D$ = +3.16 (c. 5.7, CH_2Cl_2). ^1H NMR (400 MHz, $CDCl_3$) δ_H: 7.18 (d, *J*=8.1 Hz, 2H, Ibu-2,6), 7.07 (d, *J*=8.1 Hz, 2H, Ibu-3,5), 5.45-5.28 (m, 12H, =CH), 5.28-5.22 (m, 1H, CH *sn*-2), 4.30 (dd, *J*=11.9, 4.2 Hz, 1H, CH_2 *sn*-1/3), 4.19 (dd, *J*=11.9, 4.2 Hz, 1H, CH_2 *sn*-1/3), 4.13 (dd, J=11.9, 5.8 Hz, 1H, CH_2 *sn*-1/3), 4.08 (dd, J=11.9, 6.3 Hz, 1H, CH_2 *sn*-1/3), 3.70 (q, *J*=7.2 Hz, 1H, C*H*CH_3), 2.89-2.78 (m, 10H, =CHC*H*$_2$CH=), 2.43 (d, *J*=7.2 Hz, 2H, C*H*$_2$CH(CH_3)$_2$), 2.34-2.19 (m 6H, CH_2CH_2COO DHA and CH_2COO SFA), 2.09-2.07 (m, 2H, =CHC*H*$_2$$CH_3$), 1.83 (nonet, *J*=6.9 Hz, 1H, C*H*(CH_3)$_2$), 1.64-1.54 (m, 2H, C*H*$_2$$CH_2$COO SFA), 1.49 (d, *J*=7.2 Hz, 3H, CHC*H*$_3$), 1.34-1.20 (m, 24H, CH_2), 0.97 (t, *J*=7.5 Hz, 3H, CH_3 DHA), 0.89 (d, *J*=6.8 Hz, 6H, CH(C*H*$_3$)$_2$), 0.88 (t, *J*=7.1 Hz, 3H, CH_3 SFA) ppm. $^{13}C\{H\}$ NMR (101 MHz, $CDCl_3$) δ_C: 173.9 (Ibu), 173.4 (SFA), 172.6 (DHA), 140.8, 137.4, 132.2, 129.5 (2), 129.4, 128.7, 128.5 (2), 128.4, 128.3, 128.22, 128.16, 128.0, 127.8 (2), 127.3, 127.2, 69.3, 62.23, 62.17, 45.19, 45.18, 34.2, 33.9, 32.1, 30.3, 30.1 (2), 29.9 (2), 29.81, 29.78, 29.6, 29.5, 29.4, 29.3, 25.8 (2), 25.73, 25.69 (2), 25.0, 22.8, 22.7 (2), 22.5, 20.7, 18.5, 14.4, 14.3 ppm. HRMS (ESI) *m*/*z*: [M + Na]$^+$ calcd for $C_{54}H_{84}O_6Na$ 851.6160; found, 851.6158.

3.6.6. Synthesis of 3-[4Z,7Z,10Z,13Z,16Z,19Z)-docosa-4,7,10,13,16,19-hexaenoyl]-2-[(*S*)-2-(4-isobutyl-phenyl)propanoyl]-1-octanoyl-*sn*-glycerol, (*R*,*S*')-**12b**

The same procedure was followed as described for (*R*,*S*')-**12a** using 2-[(*S*)-2-(4-isobutylphenyl)propanoyl]-1-octanoyl-*sn*-glycerol (*S*,*S*')-**8b** (29 mg, 0.071 mmol), DHA (25 mg, 0.076 mmol), CH_2Cl_2 (3 mL), DMAP (10 mg, 0.077 mmol) and EDCI (20 mg, 0.101 mmol). Purification on a silica gel chromatography using pet. ether/ethyl acetate (4:1) as eluent afforded the product (*R*,*S*')-**12b** as a yellow oil, in 83% yield (43 mg, 0.059 mmol). $[\alpha]^{20}_D$ = +7.86 (c. 3.0, CH_2Cl_2). IR (NaCl, ν_{max} / cm^{-1}): 3013 (s), 2958 (vs), 2931 (vs), 2870 (vs), 1741 (vs), 1657 (s). ^1H NMR (400 MHz, $CDCl_3$) δ_H: 7.18 (d, *J*=8.1 Hz, 2H, Ibu-2,6), 7.07 (d, *J*=8.1 Hz, 2H, Ibu-3,5), 5.45-5.22 (m, 13H, =CH and CH *sn*-2), 4.30 (dd, *J*=11.9, 4.2 Hz, 1H, CH_2 *sn*-1/3), 4.18 (dd, *J*=11.9, 4.2 Hz, 1H, CH_2 *sn*-1/3), 4.14 (dd, J=11.9, 6.0 Hz, 1H, CH_2 *sn*-1/3), 4.06 (dd, J=11.9, 6.3 Hz, 1H, CH_2 *sn*-1/3), 3.70 (q, *J*=7.2 Hz, 1H, C*H*CH_3), 2.89-2.77 (m, 10H, =CHC*H*$_2$CH=), 2.43 (d, *J*=7.2 Hz, 2H, C*H*$_2$CH(CH_3)$_2$), 2.40-2.27 (m 4H, CH_2CH_2COO DHA), 2.17-2.13 (m, 2H, =CHC*H*$_2$$CH_3$), 2.08 (t, *J*=7.4 Hz, 2H, CH_2COO SFA), 1.83 (nonet, *J*=6.8 Hz, 1H, C*H*(CH_3)$_2$), 1.64-1.54 (m, 2H, C*H*$_2$$CH_2$COO SFA), 1.49 (d, *J*=7.2 Hz, 3H, CHC*H*$_3$), 1.37-1.24 (m, 8H, CH_2), 0.97 (t, *J*=7.5 Hz, 3H, CH_3 DHA), 0.90 (d, *J*=6.6 Hz, 6H, CH(C*H*$_3$)$_2$), 0.89 (t, *J*=6.4 Hz, 3H, CH_3 SFA) ppm. $^{13}C\{H\}$ NMR (101 MHz, $CDCl_3$) δ_C: 173.8 (Ibu), 173.1 (SFA), 172.5 (DHA), 140.6, 137.2, 132.0, 129.5 (2), 129.3, 128.6, 128.31, 128.26, 128.2, 128.08, 128.06, 128.0, 127.9, 127.7, 127.1 (2), 127.0, 69.2, 62.2, 61.9, 45.1, 45.0, 33.9, 33.8, 31.7, 30.2, 29.0, 28.9, 25.63 (2), 25.58, 25.5 (2), 24.7, 22.9, 22.6 (2), 22.4, 20.54, 18.3, 14.3, 14.0 ppm. HRMS (ESI) *m*/*z*: [M + Na]$^+$ calcd for $C_{46}H_{68}O_6Na$ 739.4908; found, 739.4897.

3.6.7. Synthesis of 3-[4Z,7Z,10Z,13Z,16Z,19Z)-docosa-4,7,10,13,16,19-hexaenoyl]-1-decanoyl-2-[(*S*)-2-(4-iso-butylphenyl)propanoyl]-*sn*-glycerol, (*R*,*S*')-**12c**

The same procedure was followed as described for (*R*,*S*')-**12a** using 1-decanoyl-2-[(*S*)-2-(4-isobutylphenyl)propanoyl]-*sn*-glycerol (*S*,*S*')-**8c** (55 mg, 0.127 mmol), DHA (45 mg, 0.140 mmol), CH_2Cl_2 (3 mL), DMAP (19 mg, 0.197 mmol) and EDCI (38 mg, 0.197 mmol). Purification on a silica gel chromatography using pet. ether/ethyl acetate (4:1) as eluent afforded the product (*R*,*S*')-**12c** as a yellow oil, in 80% yield (75 mg, 0.101 mmol). $[\alpha]^{20}_D$ = +4.57 (c. 6.5, CH_2Cl_2). IR (NaCl, ν_{max} / cm^{-1}): 3013 (s), 2957 (vs), 2927 (vs), 2855 (vs), 1744 (vs). ^1H NMR (400 MHz, $CDCl_3$) δ_H: 7.18 (d, *J*=8.1 Hz, 2H, Ibu-2,6), 7.07 (d, *J*=8.1 Hz, 2H, Ibu-3,5), 5.45-5.22 (m, 13H, =CH) and CH *sn*-2), 4.30 (dd, *J*=11.9, 4.2 Hz, 1H, CH_2 *sn*-1/3), 4.18 (dd,

J=11.9, 4.2 Hz, 1H, CH_2 *sn*-1/3), 4.14 (dd, J=11.9, 6.0 Hz, 1H, CH_2 *sn*-1/3), 4.06 (dd, J=11.9, 6.4 Hz, 1H, CH_2 *sn*-1/3), 3.70 (q, *J*=7.2 Hz, 1H, C*H*CH_3), 2.89-2.77 (m, 10H, =CHC*H*$_2$CH=), 2.43 (d, *J*=7.2 Hz, 2H, C*H*$_2$CH(CH_3)$_2$), 2.40-2.27 (m 4H, CH_2CH_2COO DHA), 2.17-2.13 (m, 2H, =CHC*H*$_2$$CH_3$), 2.08 (t, *J*=7.4 Hz, 2H, CH_2COO SFA), 1.83 (nonet, *J*=6.8 Hz, 1H, C*H*(CH_3)$_2$), 1.61-1.56 (m, 2H, C*H*$_2$$CH_2$COO SFA), 1.49 (d, *J*=7.2 Hz, 3H, CHC*H*$_3$), 1.37-1.24 (m, 12H, CH_2), 0.97 (t, *J*=7.5 Hz, 3H, CH_3 DHA), 0.90 (d, *J*=6.6 Hz, 6H, CH(C*H*$_3$)$_2$), 0.89 (t, *J*=6.4 Hz, 3H, CH_3 SFA) ppm. ^{13}C{H} NMR (101 MHz, $CDCl_3$) δ_C: 173.9 (Ibu), 173.3 (SFA), 172.6 (DHA), 140.7, 137.4, 132.1, 129.6 (2), 129.4, 128.7, 128.5, 128.40, 128.38, 128.22, 128.20, 128.12, 128.08, 128.00 (2), 127.8, 127.2, 69.3, 62.34, 62.0, 45.16, 45.15, 34.0, 32.0, 30.3, 30.2, 29.6 (2), 29.4, 29.2, 25.8 (2), 25.72, 25.67 (2), 24.9, 22.8, 22.7 (2), 22.5, 20.7, 18.5, 14.4, 14.2 ppm. HRMS (ESI) *m*/*z*: [M + Na]$^+$ calcd for $C_{48}H_{72}O_6Na$ 767.5221; found, 767.5218.

3.6.8. Synthesis of 3-[4Z,7Z,10Z,13Z,16Z,19Z)-docosa-4,7,10,13,16,19-hexaenoyl]-1-dodecanoyl-2-[(*S*)-2-(4-isobutylphenyl)propanoyl]-*sn*-glycerol, (*R*,*S*')-**12d**

The same procedure was followed as described for (*R*,*S*')-**12a** using 3-dodecanoyl-2-[(*S*)-2-(4-isobutylphenyl)propanoyl]-*sn*-glycerol (*S*,*S*')-**8d** (40 mg, 0.082 mmol), DHA (32 mg, 0.097 mmol), CH_2Cl_2 (4 mL), DMAP (13 mg, 0.106 mmol) and EDCI (30 mg, 0.156 mmol). Purification on a silica gel chromatography using pet. ether/ethyl acetate (9:1) as eluent afforded the product (*R*,*S*')-**12d** as a yellow oil, in 78% yield (50 mg, 0.064 mmol). $[\alpha]^{20}_D$ = +6.57 (c. 4.2, CH_2Cl_2). IR (NaCl, ν_{max} / cm^{-1}): 3418 (br), 3012 (vs), 2925 (vs), 2854 (vs), 1743 (vs). ^{1}H NMR (400 MHz, $CDCl_3$) δ_H: 7.18 (d, *J*=8.1 Hz, 2H, Ibu-2,6), 7.07 (d, *J*=8.1 Hz, 2H, Ibu-3,5), 5.52-5.30 (m, 12H, =CH), 5.30-5.17 (m, 1H, CH *sn*-2), 4.30 (dd, *J*=11.9, 4.2 Hz, 1H, CH_2 *sn*-1/3), 4.23-4.09 (m, 2H, CH_2 *sn*-1/3), 4.06 (dd, J=11.9, 6.3 Hz, 1H, CH_2 *sn*-1/3), 3.70 (q, *J*=7.2 Hz, 1H, C*H*CH_3), 2.89-2.74 (m, 10H, =CHC*H*$_2$CH=), 2.44 (d, *J*=7.2 Hz, 2H, C*H*$_2$CH(CH_3)$_2$), 2.40-2.32 (m 4H, CH_2CH_2COO DHA), 2.18-2.11 (m, 2H, =CHC*H*$_2$$CH_3$), 2.08 (t, *J*=7.4 Hz, 2H, CH_2COO SFA), 1.83 (nonet, *J*=6.7 Hz, 1H, C*H*(CH_3)$_2$), 1.59-1.48 (m, 2H, C*H*$_2$$CH_2$COO SFA), 1.49 (d, *J*=7.2 Hz, 3H, CHC*H*$_3$), 1.37-1.24 (m, 16H, CH_2), 0.97 (t, *J*=7.5 Hz, 3H, CH_3 DHA), 0.90 (d, *J*=6.6 Hz, 6H, CH(C*H*$_3$)$_2$), 0.89 (t, *J*=7.2 Hz, 3H, CH_3 SFA) ppm. ^{13}C{H} NMR (101 MHz, $CDCl_3$) δ_C: 173.9 (Ibu), 173.3 (SFA), 172.6 (DHA), 140.7, 137.4, 132.2, 129.6 (2), 129.4, 128.7, 128.5, 128.41, 128.39, 128.23, 128.21, 128.1, 127.8, 127.2 (2), 127.1, 127.0, 69.3, 62.4, 62.1, 45.17, 45.15, 34.0, 32.0, 30.3, 30.2, 29.8 (2), 29.6, 29.5, 29.4, 29.3, 25.8, 25.74 (2), 25.68, 25.0 (2), 24.9, 22.82, 22.75 (2), 22.5, 20.7, 18.5, 14.4, 14.2 ppm. HRMS (ESI) *m*/*z*: [M + Na]$^+$ calcd for $C_{50}H_{76}O_6Na$ 795.5534; found, 795.5535.

3.6.9. Synthesis of 3-[4Z,7Z,10Z,13Z,16Z,19Z)-docosa-4,7,10,13,16,19-hexaenoyl]-2-[(*S*)-2-(4-isobutyl-phenyl)propanoyl]-1-tetradecanoyl-*sn*-glycerol, (*R*,*S*')-**12e**

The same procedure was followed as described for (*R*,*S*')-**12a** using 2-[(*S*)-2-(4-isobutylphenyl)propanoyl]-1-tetradecanoyl-*sn*-glycerol (*S*,*S*')-**8e** (30 mg, 0.061 mmol), DHA (23 mg, 0.070 mmol), CH_2Cl_2 (4 mL), DMAP (9 mg, 0.074 mmol) and EDCI (18 mg, 0.099 mmol). Purification on a silica gel chromatography using pet. ether/ethyl acetate (8.5:1.5) as eluent afforded the product (*R*,*S*')-**12e** as a yellow oil, in 82% yield (40 mg, 0.050 mmol). $[\alpha]^{20}_D$ = +7.01 (c. 4.0, CH_2Cl_2). IR (NaCl, ν_{max} / cm^{-1}): 3013 (s), 2956 (vs), 2925 (vs), 2853 (vs), 1743 (vs). ^{1}H NMR (400 MHz, $CDCl_3$) δ_H: 7.18 (d, *J*=8.1 Hz, 2H, Ibu-2,6), 7.07 (d, *J*=8.1 Hz, 2H, Ibu-3,5), 5.47-5.27 (m, 12H, =CH), 5.31-5.20 (m, 1H, CH *sn*-2), 4.30 (dd, *J*=11.9, 4.2 Hz, 1H, CH_2 *sn*-1/3), 4.19 (dd, *J*=11.9, 4.2 Hz, 1H, CH_2 *sn*-1/3), 4.13 (dd, J=11.9, 5.9 Hz, 1H, CH_2 *sn*-1/3), 4.06 (dd, J=11.9, 6.3 Hz, 1H, CH_2 *sn*-1/3), 3.70 (q, *J*=7.2 Hz, 1H, C*H*CH_3), 2.90-2.77 (m, 10H, =CHC*H*$_2$CH=), 2.43 (d, *J*=7.2 Hz, 2H, C*H*$_2$CH(CH_3)$_2$), 2.41-2.32 (m 4H, CH_2CH_2COO DHA), 2.20-2.11 (m, 2H, =CHC*H*$_2$$CH_3$), 2.14-2.01 (m, 2H, CH_2COO SFA), 1.83 (nonet, *J*=6.8 Hz, 1H, C*H*(CH_3)$_2$), 1.60-1.48 (m, 2H, C*H*$_2$$CH_2$COO SFA), 1.49 (d, *J*=7.2 Hz, 3H, CHC*H*$_3$), 1.36-1.18 (m, 20H, CH_2), 0.97 (t, *J*=7.5 Hz, 3H, CH_3 DHA), 0.90 (d, *J*=6.6 Hz, 6H, CH(C*H*$_3$)$_2$), 0.89 (t, *J*=6.4 Hz, 3H, CH_3 SFA) ppm. ^{13}C{H} NMR (101 MHz, $CDCl_3$) δ_C: 174.0 (Ibu), 173.3 (SFA), 172.7 (DHA), 140.7, 137.4, 132.2, 129.6 (2), 129.4, 128.7, 128.5, 128.43, 128.41, 128.3, 128.23, 128.15, 128.0, 127.8 (2), 127.3, 127.2, 69.3, 62.4, 62.1, 45.18, 45.15, 34.02, 33.97, 31.4, 30.3, 29.8 (2), 29.6 (2), 29.5 (2), 29.4, 29.3, 25.79, 25.75 (2), 25.7, 24.56 (2), 22.8, 22.4 (2), 22.8, 20.7, 18.5, 14.4, 14.1 ppm. HRMS (ESI) *m*/*z*: [M + Na]$^+$ calcd for $C_{52}H_{80}O_6Na$ 823.5847; found, 823.5843.

3.6.10. Synthesis of 3-[4Z,7Z,10Z,13Z,16Z,19Z)-docosa-4,7,10,13,16,19-hexaenoyl]-2-[(*S*)-1-hexadecanoyl-2-(4-isobutylphenyl)propanoyl]-*sn*-glycerol, (*R*,*S*')-**12f**

The same procedure was followed as described for (*R*,*S*')-**12a** using 1-hexadecanoyl-2-[(*S*)-2-(4-isobutylphenyl)propanoyl]-*sn*-glycerol (*R*,*S*')-**8f** (12 mg, 0.023 mmol), DHA (8 mg, 0.025 mmol), CH_2Cl_2 (1 mL), DMAP (3 mg, 0.021 mmol) and EDCI (5 mg, 0.025 mmol). Purification on a silica gel chromatography using pet. ether/ethyl acetate (8.5:1.5) as eluent afforded the product (*R*,*S*')-**12f** as a yellow oil, in 79% yield (18 mg, 0.018 mmol). $[\alpha]^{20}_D$ = +3.93 (c. 1.5, CH_2Cl_2). IR (NaCl, ν_{max} / cm^{-1}): 3030 (s), 2963 (vs), 2925 (vs), 2855 (vs), 1743 (vs). ^1H NMR (400 MHz, $CDCl_3$) δ_H: 7.18 (d, *J*=8.1 Hz, 2H, Ibu-2,6), 7.07 (d, *J*=8.1 Hz, 2H, Ibu-3,5), 5.47-5.27 (m, 12H, =CH), 5.28-5.22 (m, 1H, CH *sn*-2), 4.30 (dd, *J*=11.9, 4.2 Hz, 1H, CH_2 *sn*-1/3), 4.23-4.09 (m, 2H, CH_2 *sn*-1/3), 4.06 (dd, J=11.9, 6.3 Hz, 1H, CH_2 *sn*-1/3), 3.70 (q, *J*=7.2 Hz, 1H, C*H*CH_3), 2.91-2.77 (m, 10H, =CHC*H*$_2$CH=), 2.43 (d, *J*=7.2 Hz, 2H, C*H*$_2$CH(CH_3)$_2$), 2.42-2.31 (m 4H, CH_2CH_2COO DHA), 2.20-2.10 (m, 2H, =CHC*H*$_2$$CH_3$), 2.14-2.01 (m, 2H, CH_2COO SFA), 1.83 (nonet, *J*=6.7 Hz, 1H, C*H*(CH_3)$_2$), 1.63-1.54 (m, 2H, C*H*$_2$$CH_2$COO SFA), 1.48 (d, *J*=7.2 Hz, 3H, CHC*H*$_3$), 1.30-1.22 (m, 24H, CH_2), 0.97 (t, *J*=7.5 Hz, 3H, CH_3 DHA), 0.89 (d, *J*=6.8 Hz, 6H, CH(C*H*$_3$)$_2$), 0.88 (t, *J*=7.1 Hz, 3H, CH_3 SFA) ppm. $^{13}C\{H\}$ NMR (101 MHz, $CDCl_3$) δ_C: 174.0 (Ibu), 173.3 (SFA), 172.7 (DHA), 140.7, 137.4, 132.2, 129.6 (2), 129.4, 128.7, 128.5, 128.43, 128.41, 128.25, 128.23, 128.15, 128.0, 127.8 (2), 127.3, 127.2, 69.3, 62.4, 62.1, 45.19 (2), 34.0, 33.9, 32.1, 30.3, 30.1 (2), 29.9 (2), 29.82, 29.79, 29.7, 29.5, 29.4, 29.3, 25.80 (2), 25.75, 25.7 (2), 24.9, 22.9, 22.8 (2), 22.5, 20.7, 18.5, 14.4, 14.3 ppm. HRMS (ESI) *m*/*z*: [M + Na]$^+$ calcd for $C_{54}H_{84}O_6Na$ 851.6160; found, 851.6155.

3.6.11. Synthesis of 1-[4Z,7Z,10Z,13Z,16Z,19Z)-docosa-4,7,10,13,16,19-hexaenoyl]-2-[(*S*)-2-(6-methoxy-naphthalen-2-yl)propanoyl]-3-octanoyl-*sn*-glycerol, (*S*,*S*')-**13b**

The same procedure was followed as described for (*S*,*S*')-**13a** using 2-[(*S*)-2-(6-methoxynaphthalen-2-yl)propanoyl]-3-octanoyl-*sn*-glycerol (*R*,*S*')-**9b** (35 mg, 0.081 mmol), DHA (29 mg, 0.089 mmol), CH_2Cl_2 (4 mL), DMAP (9 mg, 0.074 mmol) and EDCI (17 mg, 0.089 mmol). Purification on a silica gel chromatography using pet. ether/ethyl acetate (4:1) as eluent afforded the product (*S*,*S*')-**13b** as a yellow oil, in 85% yield (51 mg, 0.069 mmol). $[\alpha]^{20}_D$ = +2.78 (c. 4.0, CH_2Cl_2). ^1H NMR (400 MHz, $CDCl_3$) δ_H: 7.72-7.62 (m, 3H, Nap-1,4,8), 7.38 (dd, *J*=8.5, 1.9 Hz, 1H, Nap-3), 7.13 (dd, *J*=8.9, 2.5 Hz, 1H, Nap-7), 7.09 (d, *J*=2.5 Hz, 1H, Nap-5), 5.49-5.22 (m, 12H, =CH), 5.20-5.14 (m, 1H, CH *sn*-2), 4.30 (dd, *J*=11.9, 4.2 Hz, 1H, CH_2 *sn*-1/3), 4.18 (dd, *J*=11.9, 4.3 Hz, 1H, CH_2 *sn*-1/3), 4.14 (dd, *J*=11.9, 6.1 Hz, 1H, CH_2 *sn*-1/3), 4.07 (dd, J=11.9, 6.5 Hz, 1H, CH_2 *sn*-1/3), 3.90 (s, 3H, OCH_3), 3.86 (q, *J*=7.5 Hz, 1H, C*H*CH_3), 2.91-2.72 (m, 10H, =CHC*H*$_2$CH=), 2.28-2.20 (m, 2H, CH_2COO DHA), 2.19-2.11 (m, 2H, CH_2COO DHA), 2.14-1.96 (m, 4H, CH_2COO SFA and =CHC*H*$_2$$CH_3$), 1.61-1.51 (m, 5H, C*H*$_2$$CH_2$COO SFA and CHC*H*$_3$), 1.36-1.19 (m, 8H, CH_2), 0.97 (t, *J*=7.5 Hz, 3H, CH_3 DHA), 0.89 (t, *J*=6.8 Hz, 3H, CH_3 SFA) ppm. $^{13}C\{H\}$ NMR (101 MHz, $CDCl_3$) δ_C: 173.9 (Nap), 173.4 (SFA), 172.5 (DHA), 157.9, 135.4, 133.9, 132.2, 129.4 (2), 129.0, 128.7, 128.42, 128.40, 128.38, 128.3, 128.23, 128.18, 128.0, 127.8, 127.24, 127.16, 126.3, 126.1, 119.2, 105.7, 69.5, 62.20, 62.17, 55.4, 45.5, 34.1, 33.7, 31.8, 29.2, 29.1, 25.8 (2), 25.7, 25.6 (2), 25.0, 22.8, 22.5, 20.7, 18.5, 14.4, 14.2 ppm. HRMS (ESI) *m*/*z*: [M + Na]$^+$ calcd for $C_{47}H_{64}O_7Na$ 763.4544; found, 763.4543.

3.6.12. Synthesis of 3-decanoyl-1-[4Z,7Z,10Z,13Z,16Z,19Z)-docosa-4,7,10,13,16,19-hexaenoyl]-2-[(*S*)-2-(6-methoxynaphthalen-2-yl)propanoyl]-*sn*-glycerol, (*S*,*S*')-**13c**

The same procedure was followed as described for (*S*,*S*')-**13a** using 3-decanoyl-2-[(*S*)-2-(6-methoxynaphthalen-2-yl)propanoyl]-*sn*-glycerol (*R*,*S*')-**9c** (20 mg, 0.044 mmol), DHA (16 mg, 0.048 mmol), CH_2Cl_2 (1.5 mL), DMAP (5 mg, 0.040 mmol) and EDCI (9 mg, 0.048 mmol). Purification on a silica gel chromatography using pet. ether/ethyl acetate (8.5:1.5) as eluent afforded the product (*S*,*S*')-**13c** as a yellow oil, in 89% yield (30 mg, 0.039 mmol). $[\alpha]^{20}_D$ = +6.60 (c. 3.0, CH_2Cl_2). ^1H NMR (400 MHz, $CDCl_3$) δ_H: 7.73-7.62 (m, 3H, Nap-1,4,8), 7.38 (dd, *J*=8.5, 1.9 Hz, 1H, Nap-3), 7.13 (dd, *J*=8.9, 2.5 Hz, 1H, Nap-7), 7.09 (d, *J*=2.5 Hz, 1H, Nap-5), 5.44-5.23 (m, 12H, =CH), 5.23-5.09 (m, 1H, CH *sn*-2), 4.30 (dd, *J*=11.9, 4.2 Hz, 1H, CH_2 *sn*-1/3),

4.18 (dd, *J*=11.9, 4.3 Hz, 1H, CH_2 *sn*-1/3), 4.14 (dd, *J*=11.9, 6.1 Hz, 1H, CH_2 *sn*-1/3), 4.07 (dd, J=11.9, 6.5 Hz, 1H, CH_2 *sn*-1/3), 3.90 (s, 3H, OCH_3), 3.86 (q, *J*=7.5 Hz, 1H, C*H*CH_3), 2.89-2.64 (m, 10H, =CHC*H*$_2$CH=), 2.28-2.20 (m, 2H, CH_2COO DHA), 2.20-2.13 (m, 2H, CH_2COO DHA), 2.13-1.98 (m, 4H, CH_2COO SFA and =CHC*H*$_2$$CH_3$), 1.61-1.51 (m, 5H, C*H*$_2$$CH_2$COO SFA and CHC*H*$_3$), 1.36-1.19 (m, 12H, CH_2), 0.97 (t, *J*=7.5 Hz, 3H, CH_3 DHA), 0.89 (t, *J*=6.8 Hz, 3H, CH_3 SFA) ppm. ^{13}C{H} NMR (101 MHz, $CDCl_3$) δ_C: 173.9 (Nap), 173.4 (SFA), 172.5 (DHA), 157.9, 135.3, 133.9, 132.2, 129.4 (2), 129.0, 128.7, 128.42, 128.42, 128.39, 128.3, 128.22, 128.17, 128.0, 127.8, 127.24, 127.16, 126.3, 126.1, 119.20, 105.7, 69.5, 62.19, 62.16, 55.4, 45.5, 34.1, 33.7, 32.0, 29.6 (2), 29.41, 29.39, 25.8 (2), 25.7, 25.6 (2), 25.0, 22.8, 22.5, 20.7, 18.5, 14.4, 14.3 ppm. HRMS (ESI) *m*/*z*: [M + Na]$^+$ calcd for $C_{49}H_{68}O_7Na$ 791.4857; found, 791.4853.

3.6.13. Synthesis of 3-dodecanoyl-1-[4Z,7Z,10Z,13Z,16Z,19Z)-docosa-4,7,10,13,16,19-hexaenoyl]-2-[(*S*)-2-(6-methoxynaphthalen-2-yl)propanoyl]-*sn*-glycerol, (*S,S*')-**13d**

The same procedure was followed as described for (*S,S*')-**13a** using 3-dodecanoyl-2-[(*S*)-2-(6-methoxynaphthalen-2-yl)propanoyl]-*sn*-glycerol (*R,S*')-**9d** (43 mg, 0.085 mmol), DHA (31 mg, 0.094 mmol), CH_2Cl_2 (3 mL), DMAP (10 mg, 0.077 mmol) and EDCI (18 mg, 0.093 mmol). Purification on a silica gel chromatography using pet. ether/ethyl acetate (8.5:1.5) as eluent afforded the product (*S,S*')-**13d** as a yellow oil, in 92% yield (61 mg, 0.078 mmol). $[\alpha]^{20}_D$ = +3.67 (c. 6.0, CH_2Cl_2). ^{1}H NMR (400 MHz, $CDCl_3$) δ_H: 7.71-7.64 (m, 3H, Nap-1,4,8), 7.38 (dd, *J*=8.5, 1.9 Hz, 1H, Nap-3), 7.13 (dd, *J*=8.9, 2.5 Hz, 1H, Nap-7), 7.09 (d, *J*=2.5 Hz, 1H, Nap-5), 5.43-5.23 (m, 12H, =CH), 5.23-5.09 (m, 1H, CH *sn*-2), 4.30 (dd, *J*=11.9, 4.2 Hz, 1H, CH_2 *sn*-1/3), 4.18 (dd, *J*=11.9, 4.3 Hz, 1H, CH_2 *sn*-1/3), 4.14 (dd, *J*=11.9, 6.1 Hz, 1H, CH_2 *sn*-1/3), 4.07 (dd, J=11.9, 6.5 Hz, 1H, CH_2 *sn*-1/3), 3.90 (s, 3H, OCH_3), 3.86 (q, *J*=7.5 Hz, 1H, C*H*CH_3), 2.88-2.74 (m, 10H, =CHC*H*$_2$CH=), 2.27-2.21 (m, 2H, CH_2COO DHA), 2.21-2.13 (m, 2H, CH_2COO DHA), 2.13-1.98 (m, 4H, CH_2COO SFA and =CHC*H*$_2$$CH_3$), 1.61-1.51 (m, 5H, C*H*$_2$$CH_2$COO SFA and CHC*H*$_3$), 1.36-1.19 (m, 12H, CH_2), 0.97 (t, *J*=7.5 Hz, 3H, CH_3 DHA), 0.89 (t, *J*=6.8 Hz, 3H, CH_3 SFA) ppm. ^{13}C{H} NMR (101 MHz, $CDCl_3$) δ_C: 173.9 (Nap), 173.4 (SFA), 172.5 (DHA), 157.8, 135.3, 133.9, 132.2, 129.4 (2), 129.0, 128.7, 128.5, 128.42, 128.39, 128.3, 128.22, 128.17, 128.0, 127.8, 127.23, 127.16, 126.3, 126.1, 119.2, 105.7, 69.5, 62.19, 62.16, 55.4, 45.5, 34.1, 33.7, 32.1, 29.84 (2), 29.76, 29.6, 29.5, 29.4, 29.3 (2), 25.8, 25.7 (2), 25.0, 22.8, 22.5, 20.7, 18.5, 14.4, 14.3 ppm. HRMS (ESI) *m*/*z*: [M + Na]$^+$ calcd for $C_{51}H_{72}O_7Na$ 819.5170; found, 819.5162.

3.6.14. Synthesis of 1-[4*Z*,7*Z*,10*Z*,13*Z*,16*Z*,19*Z*)-docosa-4,7,10,13,16,19-hexaenoyl]-2-[(*S*)-2-(6-methoxynaphthalen-2-yl)propanoyl]-3-tetradecanoyl-*sn*-glycerol, (*S,S*')-**13e**

The same procedure was followed as described for (*S,S*')-**13a** using 2-[(*S*)-2-(6-methoxynaphthalen-2-yl)propanoyl]-3-tetradecanoyl-*sn*-glycerol (*R,S*')-**9e** (25 mg, 0.049 mmol), DHA (18 mg, 0.054 mmol), CH_2Cl_2 (2 mL), DMAP (5 mg, 0.044 mmol) and EDCI (10 mg, 0.053 mmol). Purification on a silica gel chromatography using pet. ether/ethyl acetate (8.5:1.5) as eluent afforded the product (*S,S*')-**13e** as a yellow oil, in 93% yield (43 mg, 0.046 mmol). $[\alpha]^{20}_D$ = +7.37 (c. 3.0, CH_2Cl_2). ^{1}H NMR (400 MHz, $CDCl_3$) δ_H: 7.72-7.64 (m, 3H, Nap-1,4,8), 7.38 (dd, *J*=8.5, 1.9 Hz, 1H, Nap-3), 7.13 (dd, *J*=8.9, 2.5 Hz, 1H, Nap-7), 7.09 (d, *J*=2.5 Hz, 1H, Nap-5), 5.45-5.23 (m, 12H, =CH), 5.23-5.09 (m, 1H, CH *sn*-2), 4.30 (dd, *J*=11.9, 4.2 Hz, 1H, CH_2 *sn*-1/3), 4.18 (dd, *J*=11.9, 4.3 Hz, 1H, CH_2 *sn*-1/3), 4.14 (dd, *J*=11.9, 6.1 Hz, 1H, CH_2 *sn*-1/3), 4.07 (dd, J=11.9, 6.5 Hz, 1H, CH_2 *sn*-1/3), 3.90 (s, 3H, OCH_3), 3.86 (q, *J*=7.5 Hz, 1H, C*H*CH_3), 2.89-2.73 (m, 10H, =CHC*H*$_2$CH=), 2.27-2.21 (m, 2H, CH_2COO DHA), 2.21-2.13 (m, 2H, CH_2COO DHA), 2.14-1.99 (m, 4H, CH_2COO SFA and =CHC*H*$_2$$CH_3$), 1.60-1.50 (m, 5H, C*H*$_2$$CH_2$COO SFA and CHC*H*$_3$), 1.37-1.20 (m, 12H, CH_2), 0.97 (t, *J*=7.5 Hz, 3H, CH_3 DHA), 0.89 (t, *J*=6.8 Hz, 3H, CH_3 SFA) ppm. ^{13}C{H} NMR (101 MHz, $CDCl_3$) δ_C: 173.9 (Nap), 173.4 (SFA), 172.5 (DHA), 157.9, 135.3, 133.9, 132.2, 129.4 (2), 129.0, 128.7, 128.5, 128.42, 128.39, 128.3, 128.22, 128.17, 128.0, 127.8, 127.24, 127.16, 126.3, 126.1, 119.2, 105.7, 69.5, 62.19, 62.16, 55.4, 45.5, 34.1, 33.7, 32.1, 29.83 (2), 29.80 (2), 29.77, 29.6, 29.5, 29.4, 29.3 (2), 25.8, 25.7 (2), 25.0, 22.8, 22.5, 20.7, 18.5, 14.4, 14.3 ppm. HRMS (ESI) *m*/*z*: [M + Na]$^+$ calcd for $C_{53}H_{76}O_7Na$ 847.5483; found, 847.5483.

3.6.15. Synthesis of 1-[4Z,7Z,10Z,13Z,16Z,19Z)-docosa-4,7,10,13,16,19-hexaenoyl]-3-hexadecanoyl-2-[(*S*)-2-(6-methoxynaphthalen-2-yl)propanoyl]-*sn*-glycerol, (*S*,*S*')-**13f**

The same procedure was followed as described for (*S*,*S*')-**13a** using 3-hexadecanoyl-2-[(*S*)-2-(6-methoxynaphthalen-2-yl)propanoyl]-*sn*-glycerol (*R*,*S*')-**9f** (40 mg, 0.074 mmol), DHA (27 mg, 0.081 mmol), CH_2Cl_2 (3 mL), DMAP (8 mg, 0.067 mmol) and EDCI (16 mg, 0.081 mmol). Purification on a silica gel chromatography using pet. ether/ethyl acetate (8.5:1.5) as eluent afforded the product (*S*,*S*')-**13f** as a yellow oil, in 92% yield (58 mg, 0.068 mmol). $[\alpha]^{20}_D$ = +3.60 (c. 5.5, CH_2Cl_2). ^{1}H NMR (400 MHz, $CDCl_3$) δ_H: 7.71-7.64 (m, 3H, Nap-1,4,8), 7.38 (dd, *J*=8.5, 1.9 Hz, 1H, Nap-3), 7.13 (dd, *J*=8.9, 2.5 Hz, 1H, Nap-7), 7.09 (d, *J*=2.5 Hz, 1H, Nap-5), 5.44-5.23 (m, 12H, =CH), 5.23-5.09 (m, 1H, CH *sn*-2), 4.30 (dd, *J*=11.9, 4.2 Hz, 1H, CH_2 *sn*-1/3), 4.18 (dd, *J*=11.9, 4.3 Hz, 1H, CH_2 *sn*-1/3), 4.14 (dd, *J*=11.9, 6.1 Hz, 1H, CH_2 *sn*-1/3), 4.07 (dd, J=11.9, 6.5 Hz, 1H, CH_2 *sn*-1/3), 3.90 (s, 3H, OCH_3), 3.86 (q, *J*=7.5 Hz, 1H, C*H*CH_3), 2.90-2.73 (m, 10H, =CHC*H*$_2$CH=), 2.27-2.21 (m, 2H, CH_2COO DHA), 2.20-2.13 (m, 2H, CH_2COO DHA), 2.14-1.97 (m, 4H, CH_2COO SFA and =CHC*H*$_2$$CH_3$), 1.60-1.52 (m, 5H, C*H*$_2$$CH_2$COO SFA and CHC*H*$_3$), 1.37-1.20 (m, 12H, CH_2), 0.97 (t, *J*=7.5 Hz, 3H, CH_3 DHA), 0.89 (t, *J*=6.8 Hz, 3H, CH_3 SFA) ppm. ^{13}C{H} NMR (101 MHz, $CDCl_3$) δ_C: 173.9 (Nap), 173.4 (SFA), 172.5 (DHA), 157.9, 135.3, 133.9, 132.2, 129.4 (2), 129.0, 128.7, 128.5, 128.42, 128.40, 128.3, 128.22, 128.17, 128.0, 127.8, 127.24, 127.16, 126.3, 126.1, 119.2, 105.7, 69.5, 62.19, 62.16, 55.4, 45.5, 34.1, 33.7, 32.1, 29.9 (2), 29.81 (2), 29.78 (2), 29.6 (2), 29.5, 29.4, 29.3 (2), 25.8, 25.7 (2), 25.0, 22.8, 22.5, 20.7, 18.5, 14.4, 14.3 ppm. HRMS (ESI) *m*/*z*: [M + Na]$^+$ calcd for $C_{55}H_{80}O_7Na$ 875.5796; found, 875.5796.

3.6.16. Synthesis of 3-[4Z,7Z,10Z,13Z,16Z,19Z)-docosa-4,7,10,13,16,19-hexaenoyl]-2-[(*S*)-2-(6-methoxy-naphthalen-2-yl)propanoyl]-1-octanoyl-*sn*-glycerol, (*R*,*S*')-**13b**

The same procedure was followed as described for (*R*,*S*')-**13a** using 2-[(*S*)-2-(6-methoxynaphthalen-2-yl)propanoyl]-1-octanoyl-*sn*-glycerol (*S*,*S*')-**9b** (44 mg, 0.061 mmol), DHA (23 mg, 0.070 mmol), CH_2Cl_2 (4 mL), DMAP (9 mg, 0.074 mmol) and EDCI (18 mg, 0.099 mmol). Purification on a silica gel chromatography using pet. ether/ethyl acetate (4:1) as eluent afforded the product (*R*,*S*')-**13b** as a yellow oil, in 72% yield (47 mg, 0.063 mmol). $[\alpha]^{20}_D$ = +2.01 (c. 5.7, CH_2Cl_2). IR (NaCl, ν_{max} / cm^{-1}): 3013 (s), 2959 (vs), 2856 (vs), 1743 (vs), 1634 (vs), 1607 (vs). ^{1}H NMR (400 MHz, $CDCl_3$) δ_H: 7.70-7.64 (m, 3H, Nap-1,4,8), 7.38 (dd, *J*=8.5, 1.9 Hz, 1H, Nap-3), 7.13 (dd, *J*=8.9, 2.5 Hz, 1H, Nap-7), 7.10 (d, *J*=2.5 Hz, 1H, Nap-5), 5.42-5.31 (m, 12H, =CH), 5.28-5.24 (m, 1H, CH *sn*-2), 4.30 (dd, *J*=11.9, 4.2 Hz, 1H, CH_2 *sn*-1/3), 4.21-4.10 (m, 2H, CH_2 *sn*-1/3), 4.05 (dd, J=11.9, 6.4 Hz, 1H, CH_2 *sn*-1/3), 3.91 (s, 3H, OCH_3), 3.86 (q, *J*=7.2 Hz, 1H, C*H*CH_3), 2.87-2.70 (m, 10H, =CHC*H*$_2$CH=), 2.37-2.29 (m, 4H, CH_2COO DHA), 2.14-2.01 (m, 2H, CH_2COO SFA), 2.01-1.88 (m, 2H, =CHC*H*$_2$$CH_3$), 1.58 (d, *J*=7.2 Hz, 3H, CHC*H*$_3$), 1.37 (quint, *J*=7.5 Hz, 2H, C*H*$_2$$CH_2$COO SFA), 1.29-1.19 (m, 8H, CH_2), 0.97 (t, *J*=7.5 Hz, 3H, CH_3 DHA), 0.84 (t, *J*=7.0 Hz, 3H, CH_3 SFA) ppm. ^{13}C{H} NMR (101 MHz, $CDCl_3$) δ_C: 174.2 (Nap), 173.5 (SFA), 172.7 (DHA), 157.9, 137.9, 135.6, 133.9, 129.5, 129.1, 129.1, 128.7, 128.5, 128.44, 128.41, 128.3, 128.23, 128.16, 128.0, 127.8, 127.24, 127.17, 126.3, 126.1, 119.2, 105.7, 69.5, 62.4, 62.0, 55.4, 45.5, 34.0, 33.8, 31.3, 29.4, 29.2, 25.8 (2), 25.74, 25.70 (2), 24.8, 22.8, 22.4, 20.7, 18.5, 14.4, 14.0 ppm. HRMS (ESI) *m*/*z*: [M + Na]$^+$ calcd for $C_{47}H_{64}O_7Na$ 763.4544; found, 763.4546.

3.6.17. Synthesis of 1-decanoyl-3-[4Z,7Z,10Z,13Z,16Z,19Z)-docosa-4,7,10,13,16,19-hexaenoyl]-2-[(*S*)-2-(6-methoxynaphthalen-2-yl)propanoyl]-*sn*-glycerol, (*R*,*S*')-**13c**

The same procedure was followed as described for (*R*,*S*')-**13a** using 1-decanoyl-2-[(*S*)-2-(6-methoxynaphthalen-2-yl)propanoyl]-*sn*-glycerol (*S*,*S*')-**9c** (23 mg, 0.052 mmol), DHA (28 mg, 0.085 mmol), CH_2Cl_2 (5 mL), DMAP (11 mg, 0.087 mmol) and EDCI (20 mg, 0.105 mmol). Purification on a silica gel chromatography using pet. ether/ethyl acetate (8.5:1.5) as eluent afforded the product (*R*,*S*')-**13c** as a yellow oil, in 80% yield (33 mg, 0.042 mmol). $[\alpha]^{20}_D$ = +3.93 (c. 2.9, CH_2Cl_2). IR (NaCl, ν_{max} / cm^{-1}): 3013 (s), 2956 (vs), 2925 (vs), 2854 (vs), 1742 (vs), 1634 (s), 1607 (s). ^{1}H NMR (400 MHz, $CDCl_3$) δ_H: 7.74-7.59 (m, 3H, Nap-1,4,8), 7.38 (dd, *J*=8.5, 1.9 Hz, 1H, Nap-3), 7.13 (dd, *J*=8.9, 2.5 Hz, 1H, Nap-7), 7.09 (d, *J*=2.5 Hz, 1H, Nap-5), 5.46-

5.27 (m, 12H, =CH), 5.32-5.21 (m, 1H, CH *sn*-2), 4.30 (dd, *J*=11.9, 4.2 Hz, 1H, CH_2 *sn*-1/3), 4.21-4.10 (m, 2H, CH_2 *sn*-1/3), 4.05 (dd, J=11.9, 6.4 Hz, 1H, CH_2 *sn*-1/3), 3.91 (s, 3H, OCH_3), 3.86 (q, *J*=7.1 Hz, 1H, C*H*CH_3), 2.94-2.75 (m, 10H, =CHC*H*$_2$CH=), 2.41-2.07 (m, 4H, CH_2COO DHA), 2.04-1.86 (m, 4H, CH_2COO SFA) and =CHC*H*$_2$$CH_3$), 1.58 (d, *J*=7.1 Hz, 3H, CHC*H*$_3$), 1.44-1.06 (m, 2H, C*H*$_2$$CH_2$COO SFA), 1.29-1.22 (m, 12H, CH_2), 0.97 (t, *J*=7.5 Hz, 3H, CH_3 DHA), 0.89 (t, *J*=7.0 Hz, 3H, CH_3 SFA) ppm. ^{13}C{H} NMR (101 MHz, $CDCl_3$) δ_C: 173.9 (Nap), 173.3 (SFA), 172.7 (DHA), 157.9, 135.3, 133.9, 132.2, 129.6, 129.4, 129.1, 128.7, 128.5, 128.43, 128.41, 128.3, 128.23, 128.16, 128.0, 127.8, 127.24, 127.17, 126.3, 126.1, 119.2, 105.7, 69.5, 62.4, 62.0, 55.4, 45.5, 34.0, 33.9, 32.1, 29.6, 29.5, 29.4, 29.2, 25.8 (2), 25.74, 25.70 (2), 24.8, 22.8, 22.7, 20.7, 18.5, 14.4, 14.3 ppm. HRMS (ESI) *m*/*z*: [M + Na]$^+$ calcd for $C_{49}H_{68}O_7Na$ 791.4857; found, 791.4812.

3.6.18. Synthesis of 1-dodecanoyl-3-[4*Z*,7*Z*,10*Z*,13*Z*,16*Z*,19*Z*)-docosa-4,7,10,13,16,19-hexaenoyl]-2-[(*S*)-2-(6-methoxynaphthalen-2-yl)propanoyl]-*sn*-glycerol, (*R*,*S*')-**13d**

The same procedure was followed as described for (*R*,*S*')-**13a** using 1-dodecanoyl-2-[(*S*)-2-(6-methoxynaphthalen-2-yl)propanoyl]-*sn*-glycerol (*S*,*S*')-**9d** (19 mg, 0.038 mmol), DHA (14 mg, 0.041 mmol), CH_2Cl_2 (1.6 mL), DMAP (5 mg, 0.041 mmol) and EDCI (11 mg, 0.056 mmol). Purification on a silica gel chromatography using pet. ether/ethyl acetate (8.5:1.5) as eluent afforded the product (*R*,*S*')-**13d** as a yellow oil, in 92% yield (20 mg, 0.025 mmol). $[\alpha]^{20}_D$ = +3.50 (c. 2.0, CH_2Cl_2). IR (NaCl, ν_{max} / cm^{-1}): 3013 (s), 2925 (vs), 2926 (vs), 2854 (vs), 1743 (vs), 1634 (s), 1607 (s). ^{1}H NMR (400 MHz, $CDCl_3$) δ_H: 7.74-7.59 (m, 3H, Nap-1,4,8), 7.38 (dd, *J*=8.5, 1.9 Hz, 1H, Nap-3), 7.13 (dd, *J*=8.9, 2.5 Hz, 1H, Nap-7), 7.09 (d, *J*=2.5 Hz, 1H, Nap-5), 5.46-5.27 (m, 12H, =CH), 5.32-5.21 (m, 1H, CH *sn*-2), 4.30 (dd, *J*=11.9, 4.2 Hz, 1H, CH_2 *sn*-1/3), 4.21-4.10 (m, 2H, CH_2 *sn*-1/3), 4.05 (dd, J=11.9, 6.4 Hz, 1H, CH_2 *sn*-1/3), 3.91 (s, 3H, OCH_3), 3.86 (q, *J*=7.1 Hz, 1H, C*H*CH_3), 2.94-2.75 (m, 10H, =CHC*H*$_2$CH=), 22.41-2.27 (m, 4H, CH_2COO DHA), 2.04-1.86 (m, 4H, CH_2COO SFA) and =CHC*H*$_2$$CH_3$), 1.58 (d, *J*=7.1 Hz, 3H, CHC*H*$_3$), 1.44-1.06 (m, 2H, C*H*$_2$$CH_2$COO SFA), 1.29-1.22 (m, 16H, CH_2), 0.97 (t, *J*=7.5 Hz, 3H, CH_3 DHA), 0.89 (t, *J*=7.0 Hz, 3H, CH_3 SFA) ppm. ^{13}C{H} NMR (101 MHz, $CDCl_3$) δ_C: 173.9 (Nap), 173.3 (SFA), 172.7 (DHA), 157.9, 135.3, 133.9, 132.2, 129.6, 129.4, 129.0, 128.7, 128.5, 128.43, 128.41, 128.3, 128.23, 128.16, 128.0, 127.8, 127.24, 127.17, 126.3, 126.1, 119.2, 105.7, 69.5, 62.4, 62.0, 55.4, 45.5, 34.0, 33.9, 32.1, 29.8 (2), 29.6, 29.5, 29.4, 29.2, 25.8 (2), 25.7, 25.7 (2), 24.8, 22.8, 22.7, 20.7, 18.5, 14.4, 14.3 ppm. HRMS (ESI) *m*/*z*: [M + Na]$^+$ calcd for $C_{51}H_{72}O_7Na$ 819.5170; found, 819.5169.

3.6.19. Synthesis of 3-[4*Z*,7*Z*,10*Z*,13*Z*,16*Z*,19*Z*)-docosa-4,7,10,13,16,19-hexaenoyl]-2-[(*S*)-2-(6-methoxynaphthalen-2-yl)propanoyl]-1-tetradecanoyl-*sn*-glycerol, (*R*,*S*')-**13e**

The same procedure was followed as described for (*R*,*S*')-**13a** using 2-[(*S*)-2-(6-methoxynaphthalen-2-yl)propanoyl]-1-tetradecanoyl-*sn*-glycerol (*S*,*S*')-**9e** (13 mg, 0.025 mmol), DHA (9 mg, 0.027 mmol), CH_2Cl_2 (1 mL), DMAP (3 mg, 0.024 mmol) and EDCI (7 mg, 0.037 mmol). Purification on a silica gel chromatography using pet. ether/ethyl acetate (4:1) as eluent afforded the product (*R*,*S*')-**13e** as a yellow oil, in 88% yield (18 mg, 0.022 mmol). $[\alpha]^{20}_D$ = +4.58 (c. 1.8, CH_2Cl_2). IR (NaCl, ν_{max} / cm^{-1}): 3013 (s), 2924 (vs), 2953 (vs), 2853 (vs), 1743 (vs), 1632 (s), 1607 (s). ^{1}H NMR (400 MHz, $CDCl_3$) δ_H: 7.72-7.60 (m, 3H, Nap-1,4,8), 7.38 (dd, *J*=8.5, 1.9 Hz, 1H, Nap-3), 7.13 (dd, *J*=8.9, 2.5 Hz, 1H, Nap-7), 7.09 (d, *J*=2.5 Hz, 1H, Nap-5), 5.46-5.28 (m, 12H, =CH), 5.28-5.21 (m, 1H, CH *sn*-2), 4.30 (dd, *J*=11.9, 4.2 Hz, 1H, CH_2 *sn*-1/3), 4.21-4.10 (m, 2H, CH_2 *sn*-1/3), 4.05 (dd, J=11.9, 6.5 Hz, 1H, CH_2 *sn*-1/3), 3.89 (s, 3H, OCH_3), 3.85 (q, *J*=7.1 Hz, 1H, C*H*CH_3), 2.89-2.77 (m, 10H, =CHC*H*$_2$CH=), 2.40-2.07 (m, 4H, CH_2COO DHA), 2.04-1.86 (m, 4H, CH_2COO SFA and =CHC*H*$_2$$CH_3$), 1.58 (d, *J*=7.1 Hz, 3H, CHC*H*$_3$), 1.44-1.06 (m, 2H, C*H*$_2$$CH_2$COO SFA), 1.29-1.22 (m, 20H, CH_2), 0.97 (t, *J*=7.5 Hz, 3H, CH_3 DHA), 0.89 (t, *J*=7.0 Hz, 3H, CH_3 SFA) ppm. ^{13}C{H} NMR (101 MHz, $CDCl_3$) δ_C: 173.9 (Nap), 173.3 (SFA), 172.7 (DHA), 157.9, 135.3, 133.89, 132.2, 129.6, 129.4, 129.1, 128.7, 128.5, 128.44, 128.41, 128.3, 128.23, 128.16, 128.0, 127.2, 127.24, 127.17, 126.3, 126.1, 119.2, 105.7, 69.5, 62.4, 62.0, 55.3, 45.5, 34.0, 33.9, 32.1, 29.9 (2), 29.82, 29.78, 29.7, 29.5, 29.4, 29.2, 25.8 (2), 25.8, 25.7 (2), 24.8, 22.9, 22.7, 20.7, 18.5, 14.4, 14.3 ppm. HRMS (ESI) *m*/*z*: [M + Na]$^+$ calcd for $C_{53}H_{76}O_7Na$ 847.5483; found, 847.5418.

3.6.20. Synthesis of 3-[4Z,7Z,10Z,13Z,16Z,19Z)-docosa-4,7,10,13,16,19-hexaenoyl]-1-hexadecanoyl-2-[(*S*)-2-(6-methoxynaphthalen-2-yl)propanoyl]-*sn*-glycerol, (*R*,*S*')-**13f**

The same procedure was followed as described for (*R*,*S*')-**13a** using 1-hexadecanoyl-2-[(*S*)-2-(6-methoxynaphthalen-2-yl)propanoyl]-*sn*-glycerol (*S*,*S*')-**9f** (10 mg, 0.018 mmol), DHA (7 mg, 0.020 mmol), CH_2Cl_2 (1 mL), DMAP (2 mg, 0.016 mmol) and EDCI (4 mg, 0.020 mmol). Purification on a silica gel chromatography using pet. ether/ethyl acetate (9:1) as eluent afforded the product (*R*,*S*')-**13f** as a yellow oil, in 89% yield (14 mg, 0.016 mmol). $[\alpha]^{20}_D$ = +2.14 (c. 1.4, CH_2Cl_2). IR (NaCl, ν_{max} / cm^{-1}): 3013 (s), 2967 (vs), 2925 (vs), 2854 (vs), 1741 (vs), 1634 (s), 1607 (vs). ^1H NMR (400 MHz, $CDCl_3$) δ_H: 7.72-7.62 (m, 3H, Nap-1,4,8), 7.38 (dd, *J*=8.5, 1.9 Hz, 1H, Nap-3), 7.13 (dd, *J*=8.9, 2.5 Hz, 1H, Nap-7), 7.09 (d, *J*=2.5 Hz, 1H, Nap-5), 5.46-5.27 (m, 12H, =CH), 5.31-5.21 (m, 1H, CH *sn*-2), 4.30 (dd, *J*=11.9, 4.2 Hz, 1H, CH_2 *sn*-1/3), 4.21-4.10 (m, 2H, CH_2 *sn*-1/3), 4.05 (dd, J=11.9, 6.4 Hz, 1H, CH_2 *sn*-1/3), 3.91 (s, 3H, OCH_3), 3.86 (q, *J*=7.1 Hz, 1H, C*H*CH_3), 2.89-2.77 (m, 10H, =CHCH_2CH=), 2.40-2.07 (m, 4H, CH_2COO DHA), 2.04-1.86 (m, 4H, CH_2COO SFA) and =CHC$H_2$$CH_3$), 1.57 (d, *J*=7.1 Hz, 3H, CHCH_3), 1.42-1.31 (m, 2H, C$H_2$$CH_2$COO SFA), 1.29-1.22 (m, 24H, CH_2), 0.97 (t, *J*=7.5 Hz, 3H, CH_3 DHA), 0.89 (t, *J*=7.3 Hz, 3H, CH_3 SFA) ppm. $^{13}C\{H\}$ NMR (101 MHz, $CDCl_3$) δ_C: 173.9 (Nap), 173.3 (SFA), 172.7 (DHA), 157.9, 135.3, 133.9, 132.2, 129.6 (2), 129.4, 129.0, 128.73, 128.5, 128.44, 128.41, 128.23, 128.16, 128.0, 127.8, 127.24, 127.17, 126.3, 126.1, 119.2, 105.7, 69.5, 64.5, 62.4, 55.4, 45.5, 37.9, 34.0, 32.1, 30.1 (2), 29.9 (2), 29.82 (2), 29.78, 29.6, 29.5, 29.4, 29.2 (2), 25.8, 25.7 (2), 24.8, 22.9, 22.8, 20.7, 18.5, 14.4, 14.3 ppm. HRMS (ESI) *m*/*z*: $[M + Na]^+$ calcd for $C_{55}H_{80}O_7Na$ 875.5796; found, 875.5787.

NMR Spectra

^{1}H NMR (400 MHz, $CDCl_3$) of compound (*R*)-**5a**

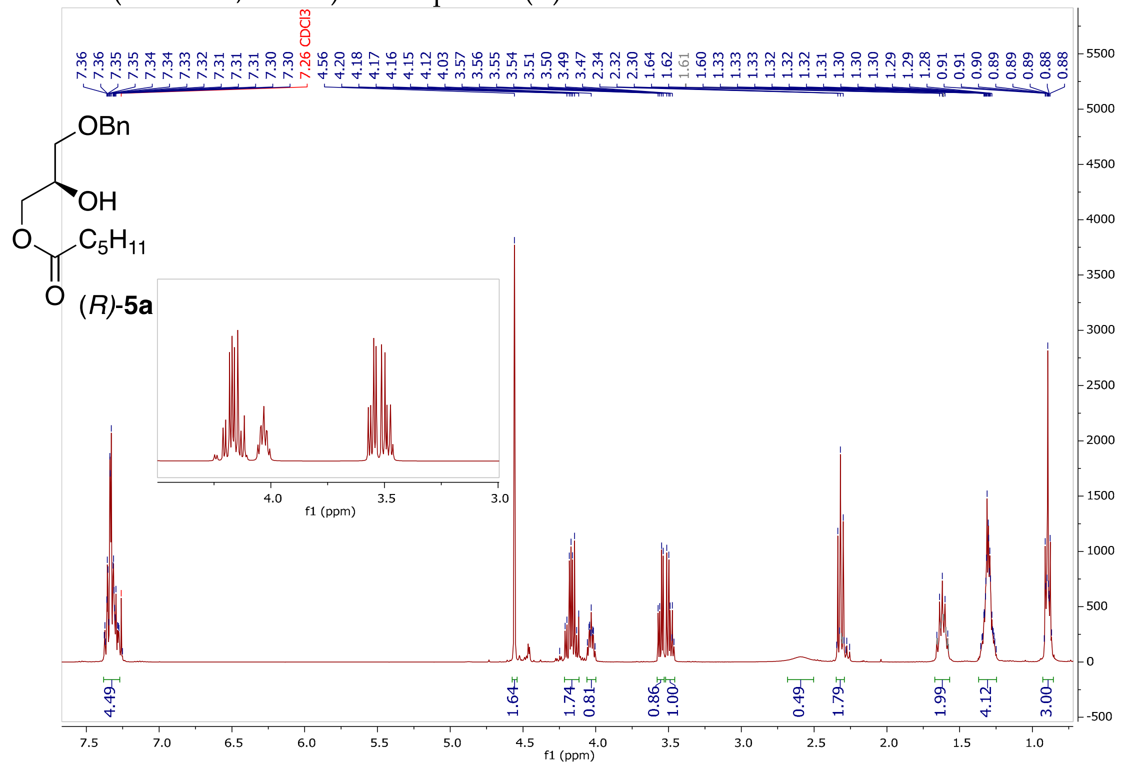

$^{13}C\{H\}$ NMR (101 MHz, $CDCl_3$) of compound (*R*)-**5a**

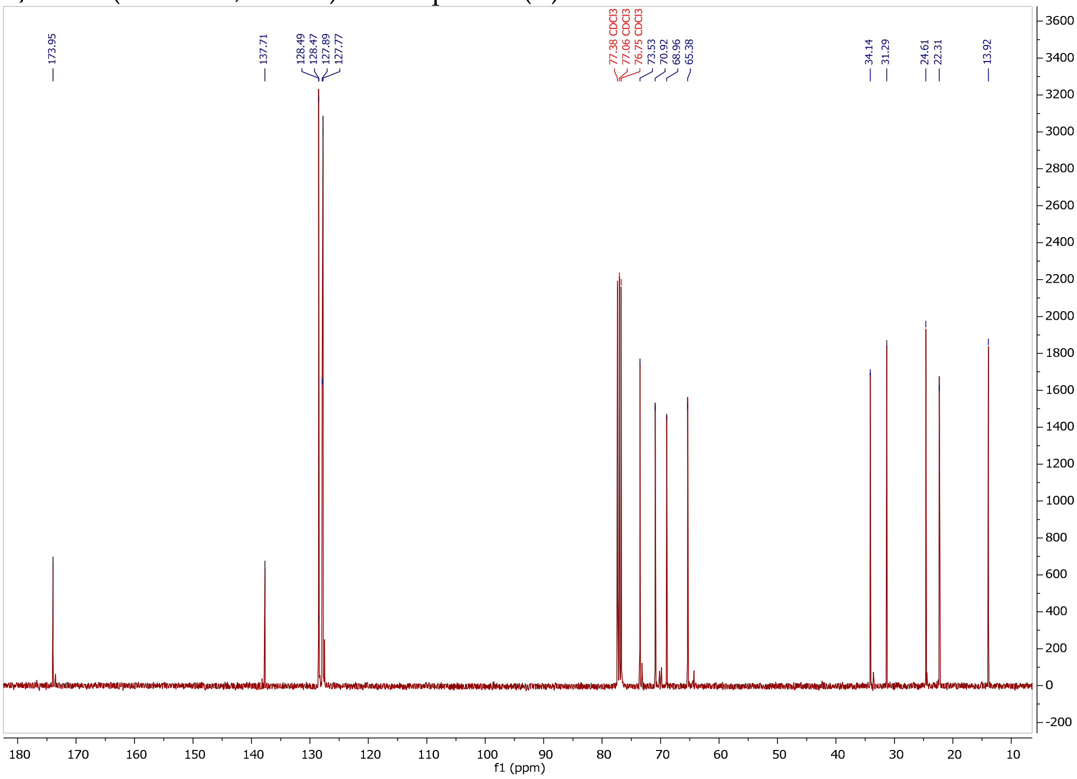

^{1}H-^{1}H COSY spectrum of compound (*R*)-**5a**

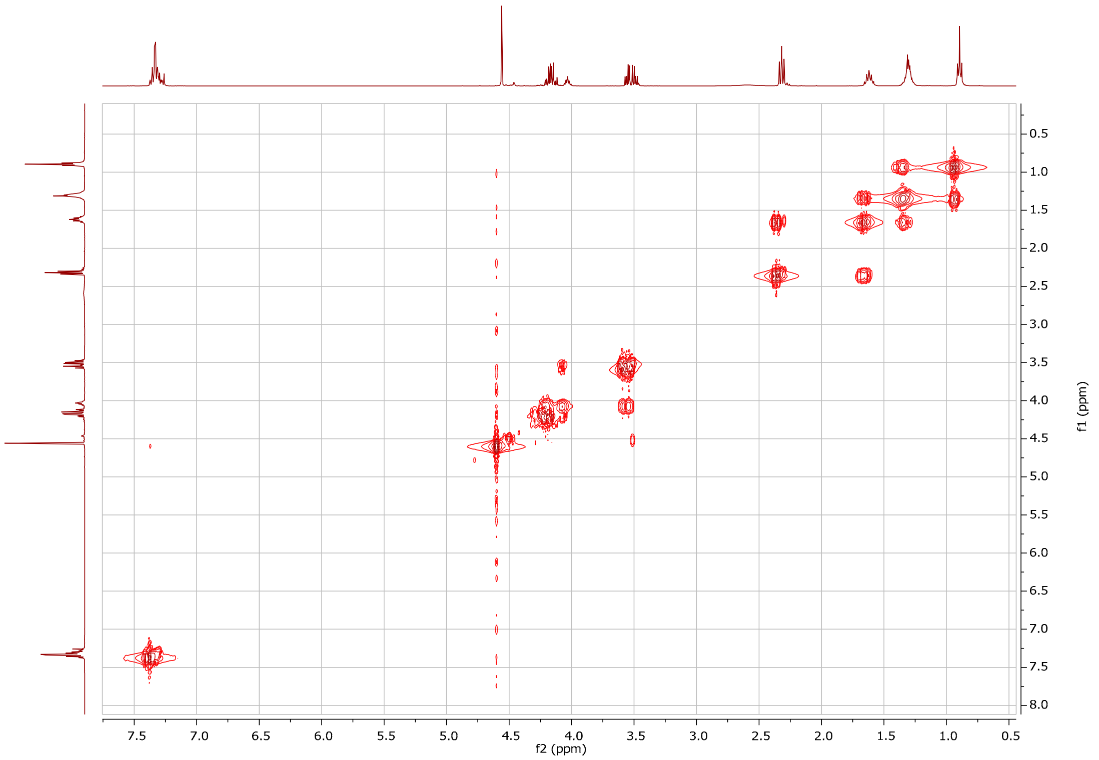

^{13}C-^{1}H HSQC spectrum of compound (*R*)-**5a**

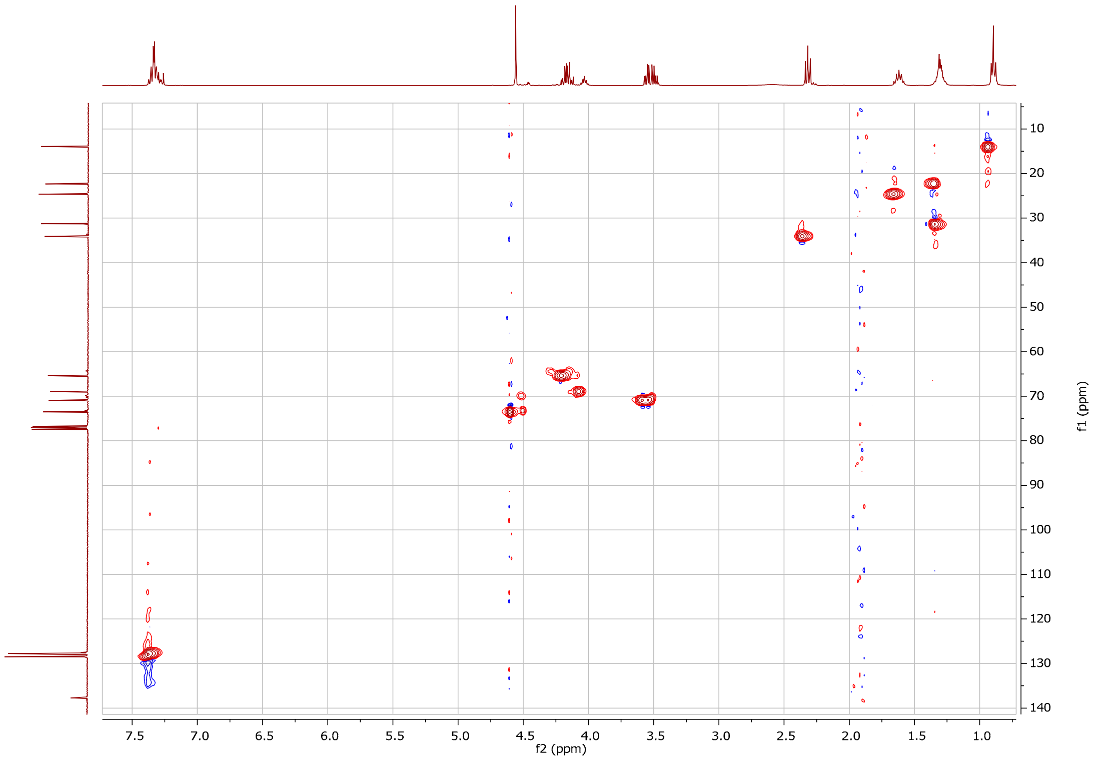

^{1}H NMR (400 MHz, CDCl$_3$) of compound (*S*)-**5a**

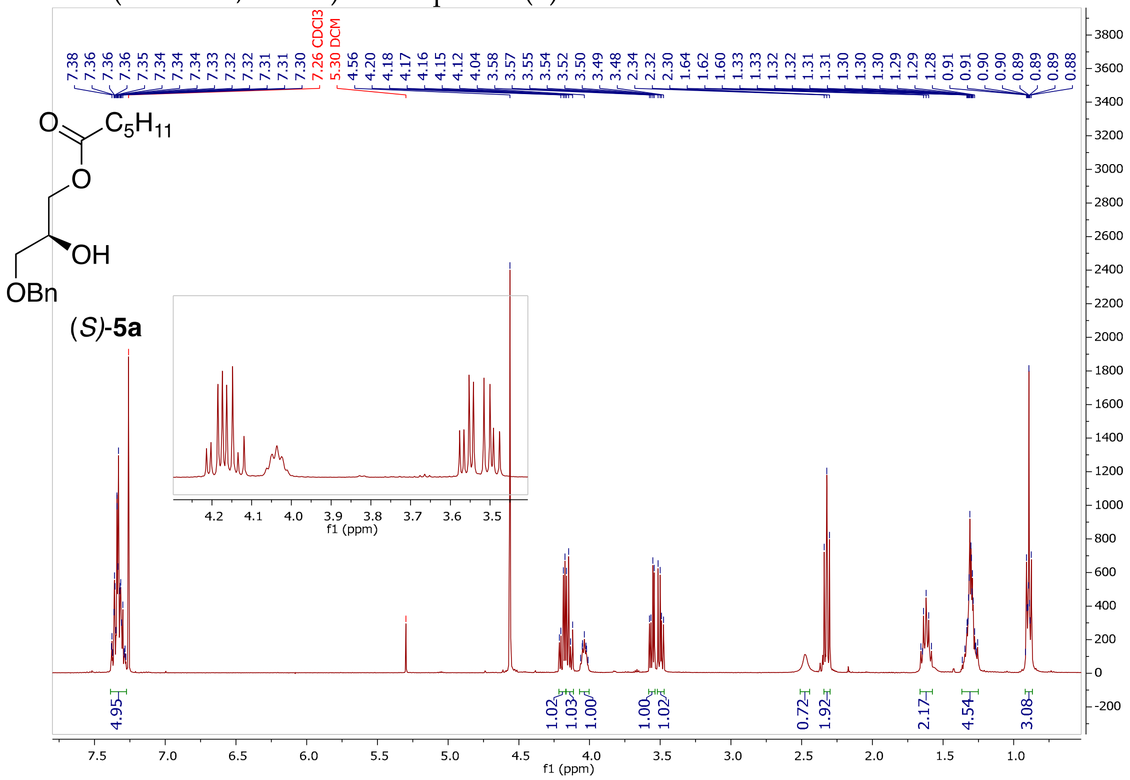

^{13}C{H} NMR (101 MHz, CDCl$_3$) of compound (*S*)-**5a**

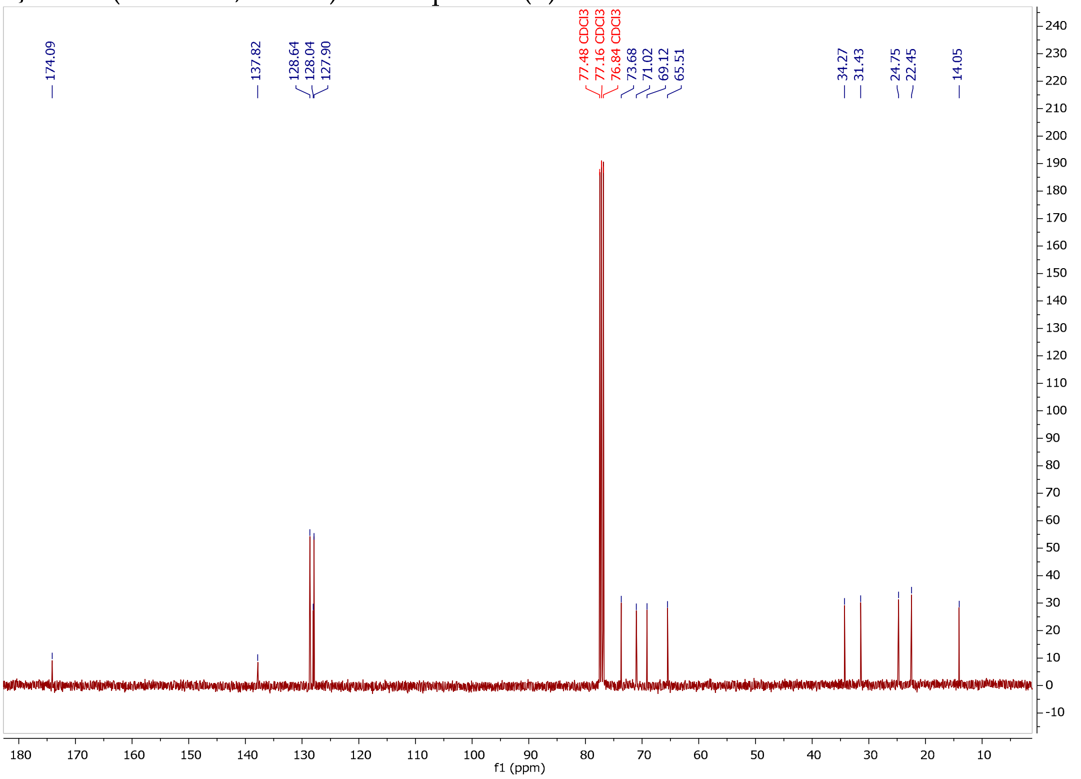

^{1}H-^{1}H COSY spectrum of compound (*S*)-**5a**

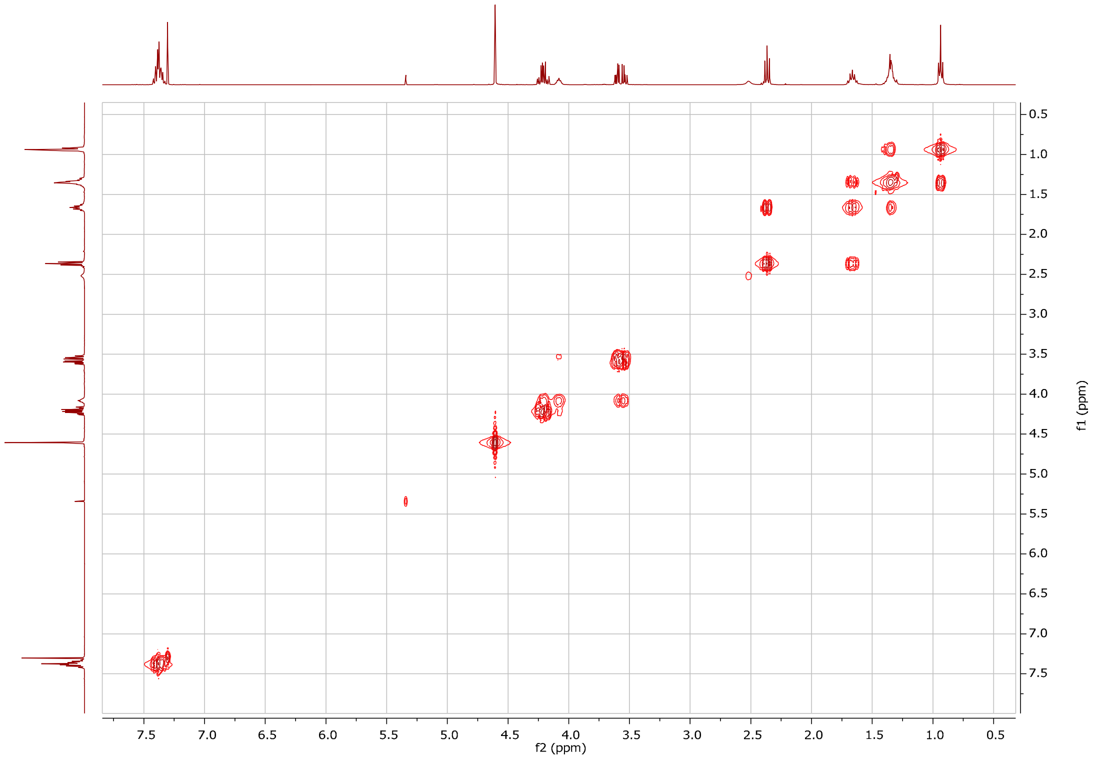

^{13}C-^{1}H HSQC spectrum of compound (*S*)-**5a**

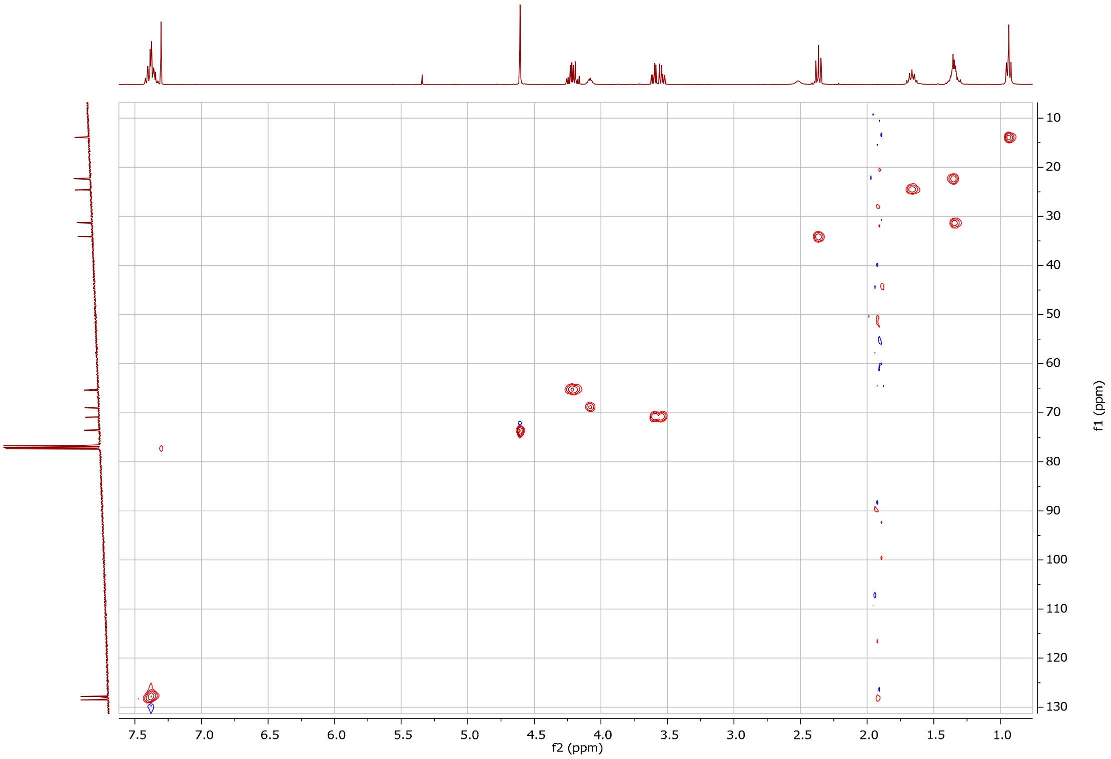

^{1}H NMR (400 MHz, $CDCl_3$) of compound (*R*,*S′*)-**6a**

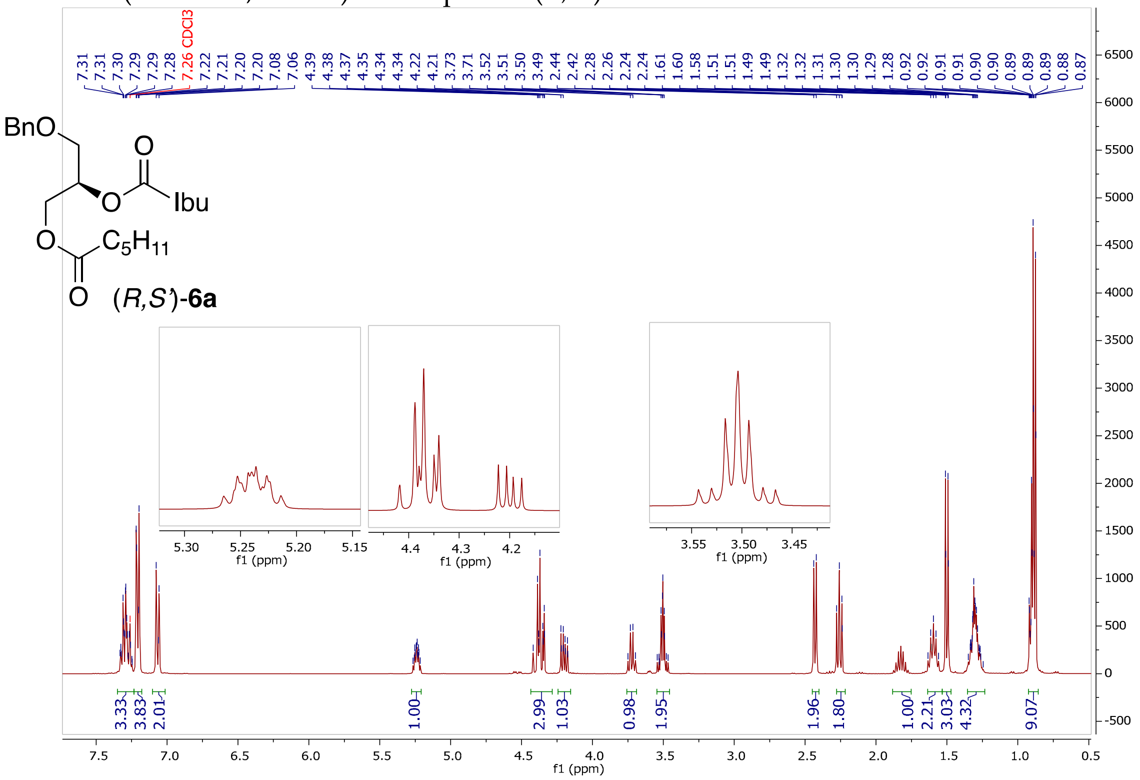

$^{13}C\{H\}$ NMR (101 MHz, $CDCl_3$) of compound (*R*,*S′*)-**6a**

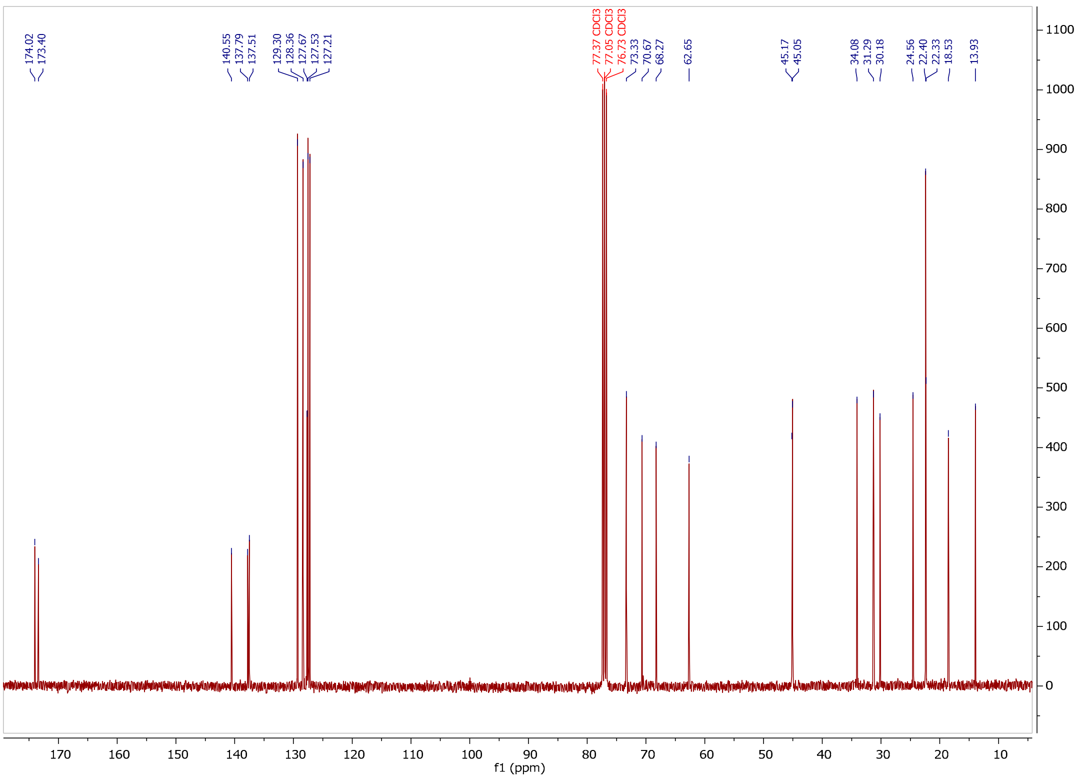

^{1}H-^{1}H COSY spectrum of compound (R,S')-**6a**

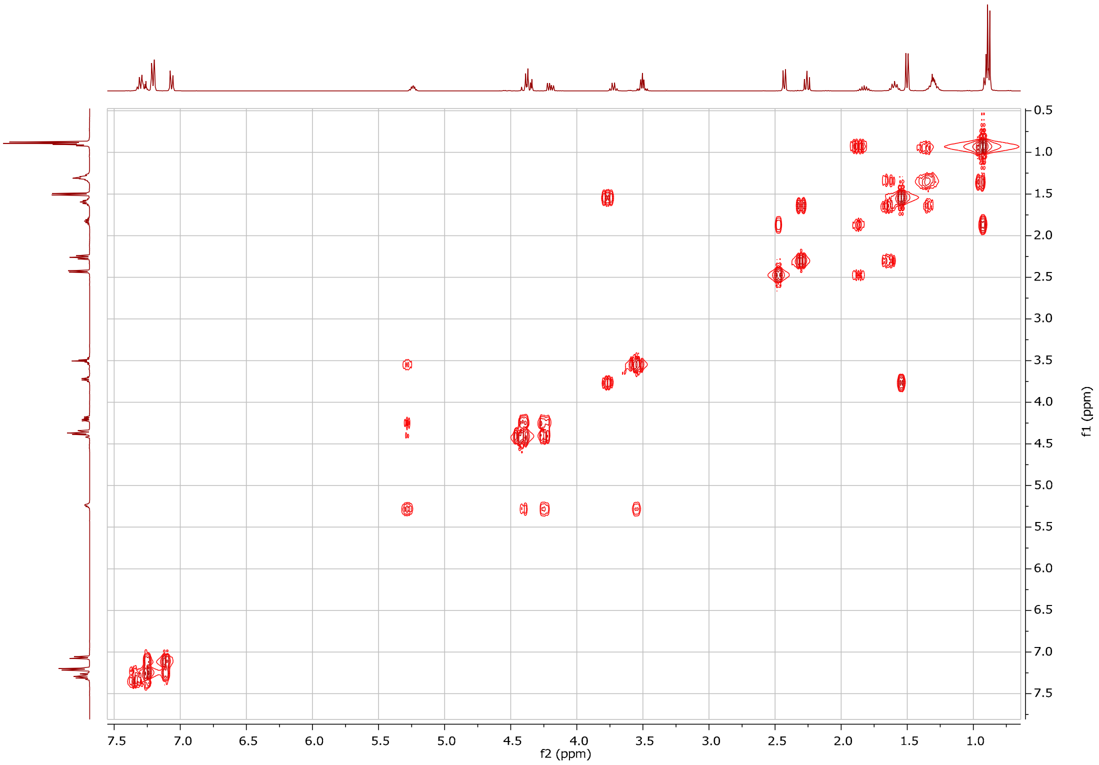

^{13}C-^{1}H HSQC spectrum of compound (R,S')-**6a**

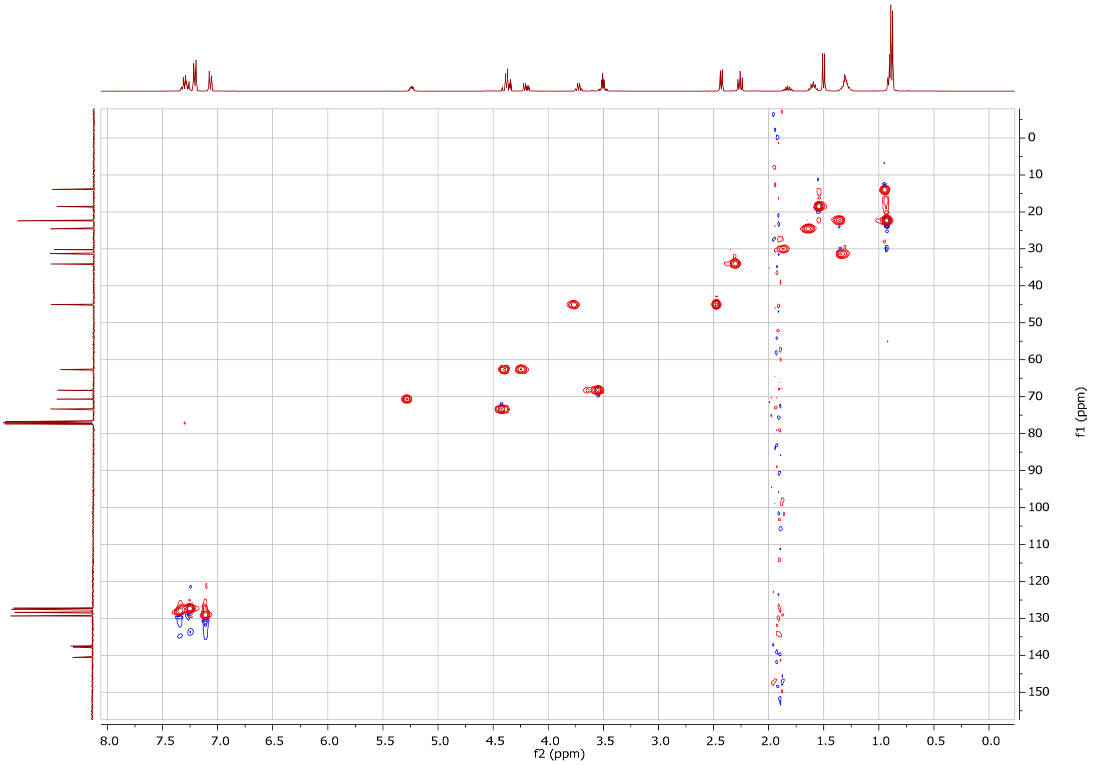

^{1}H NMR (400 MHz, $CDCl_3$) of compound (*S*,*S′*)-**6a**

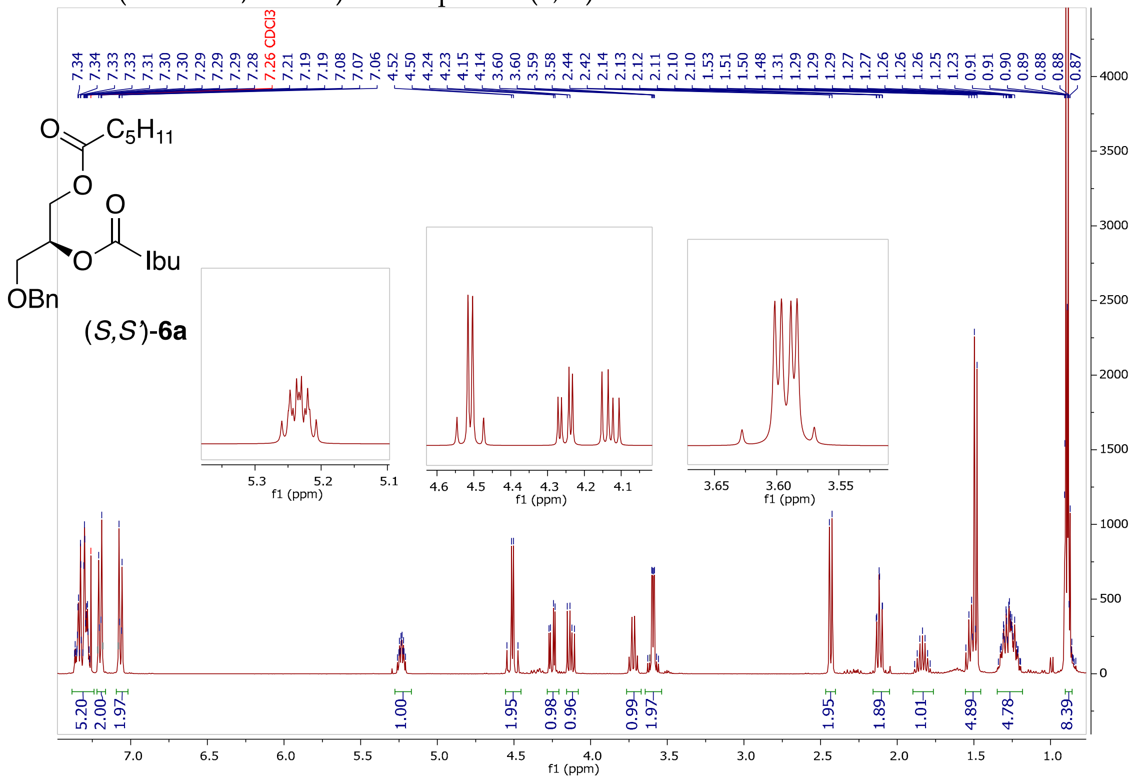

^{13}C{H} NMR (101 MHz, $CDCl_3$) of compound (*S*,*S′*)-**6a**

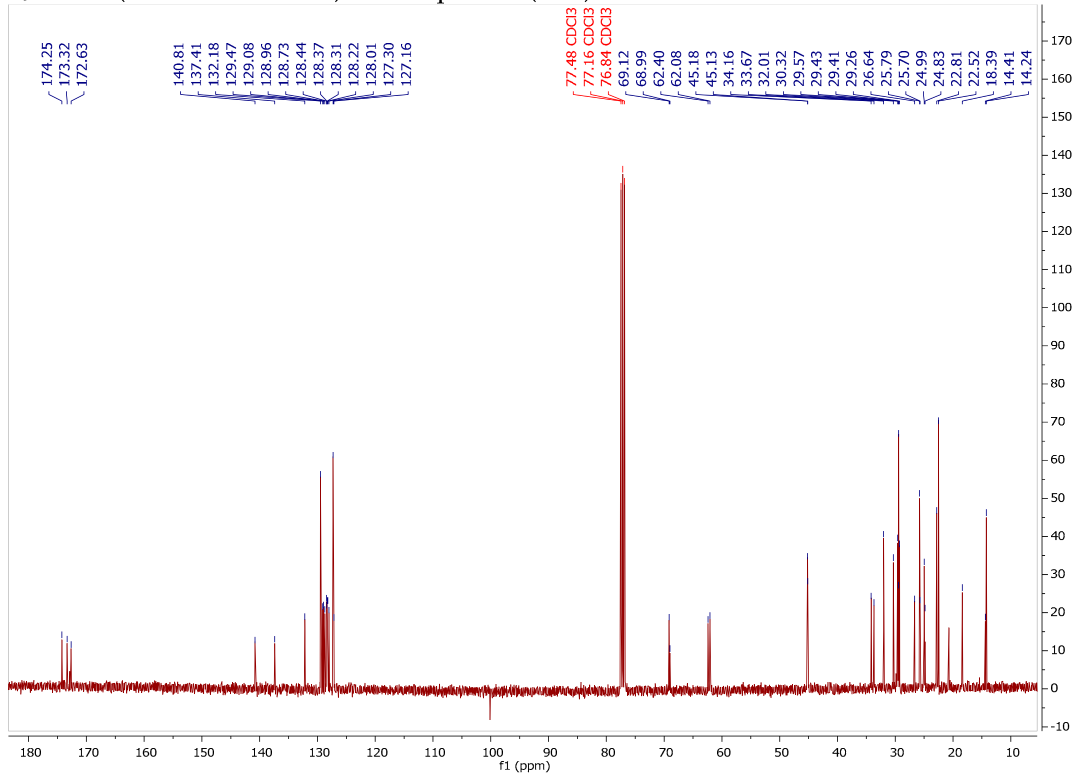

^{1}H-^{1}H COSY spectrum of compound (*S*,*S′*)-**6a**

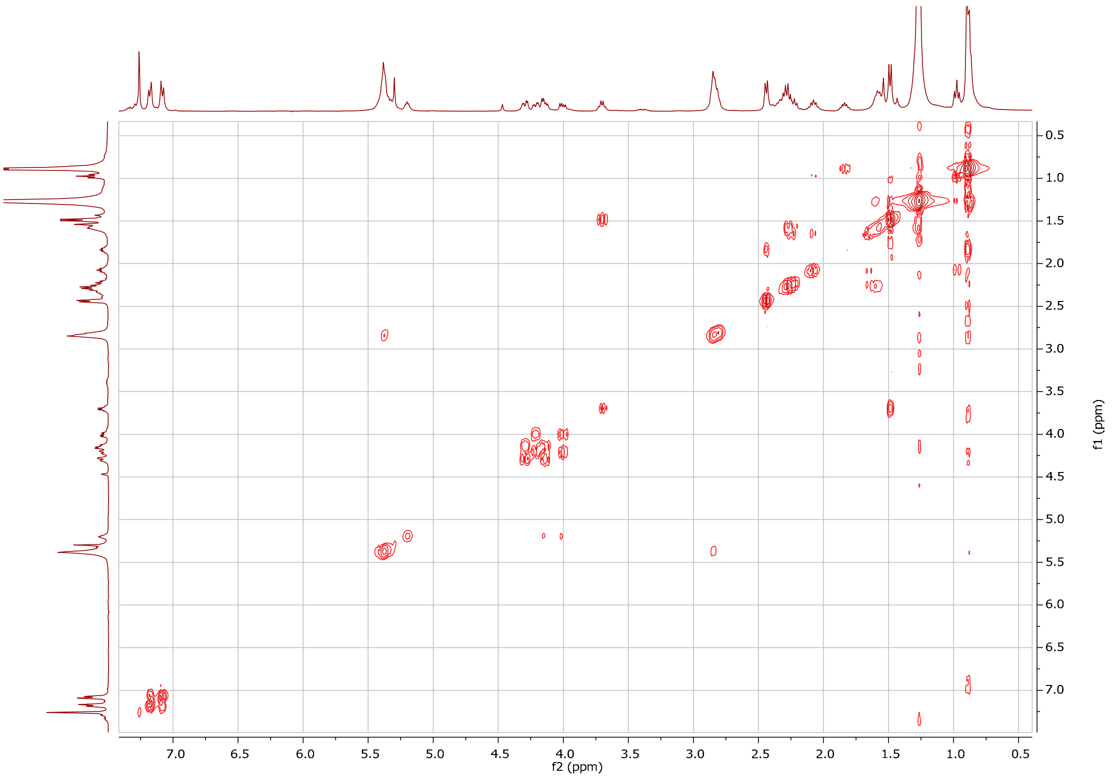

^{13}C-^{1}H HSQC spectrum of compound (*S*,*S′*)-**6a**

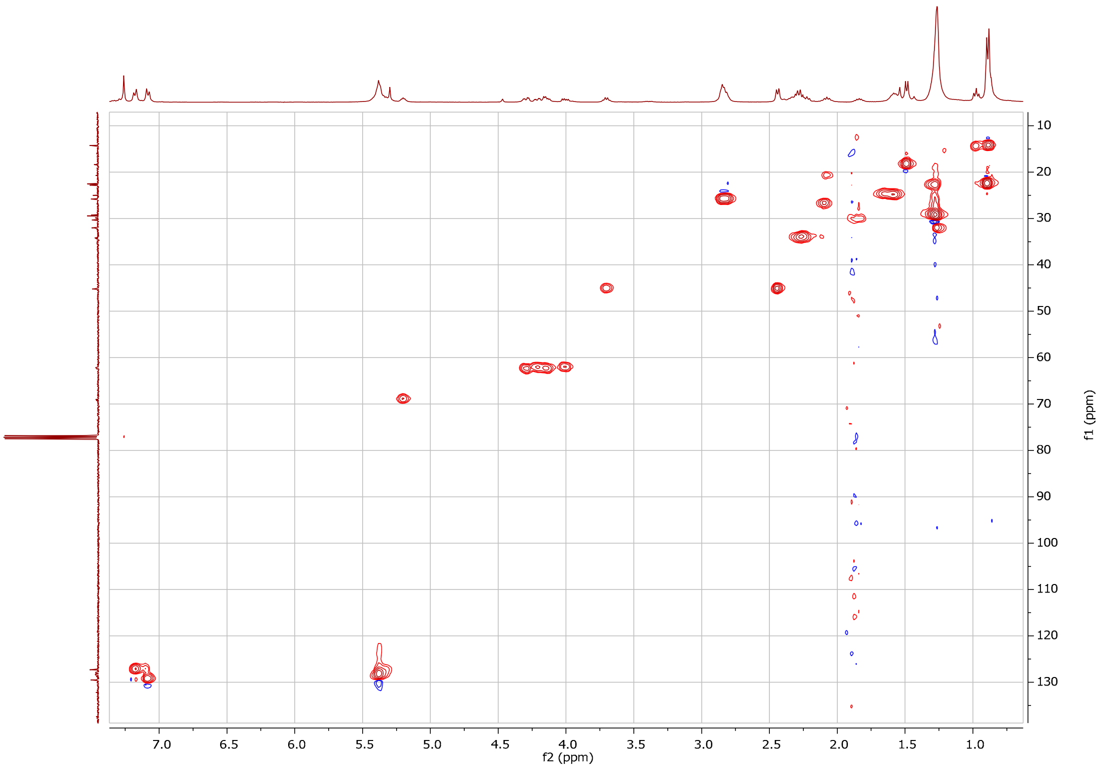

^{1}H NMR (400 MHz, $CDCl_3$) of compound (*R*,*S′*)-**7a**

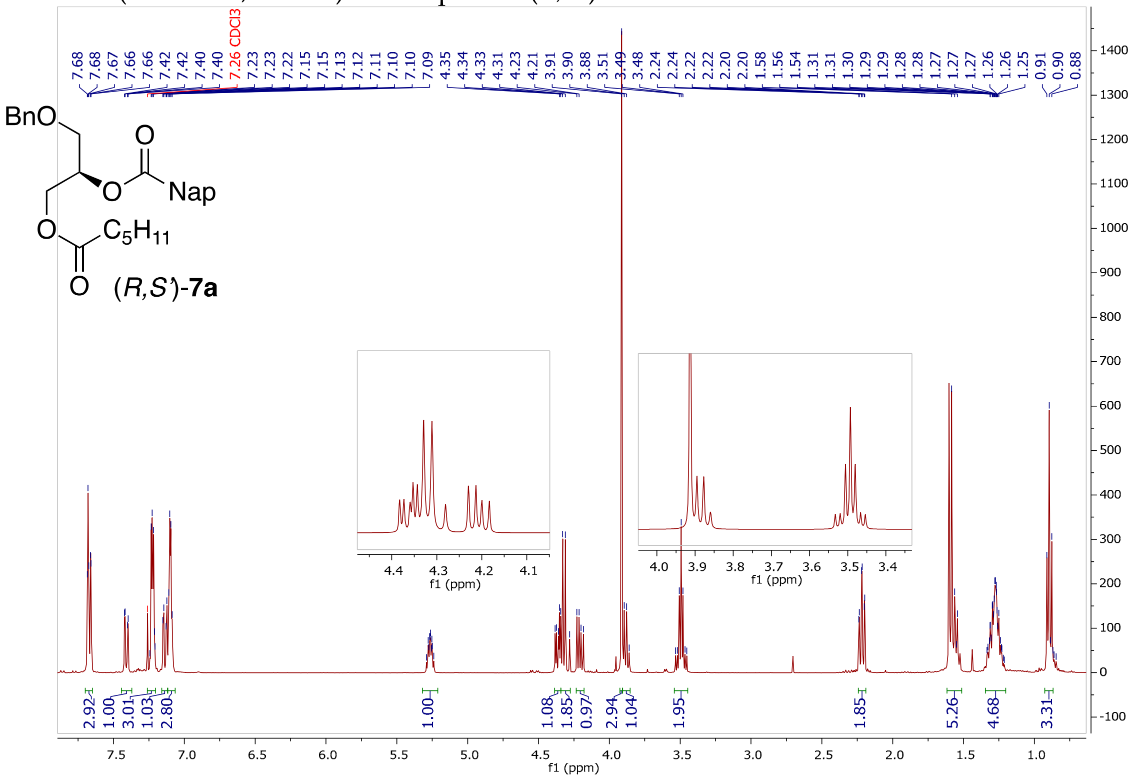

$^{13}C\{H\}$ NMR (101 MHz, $CDCl_3$) of compound (*R*,*S′*)-7**a**

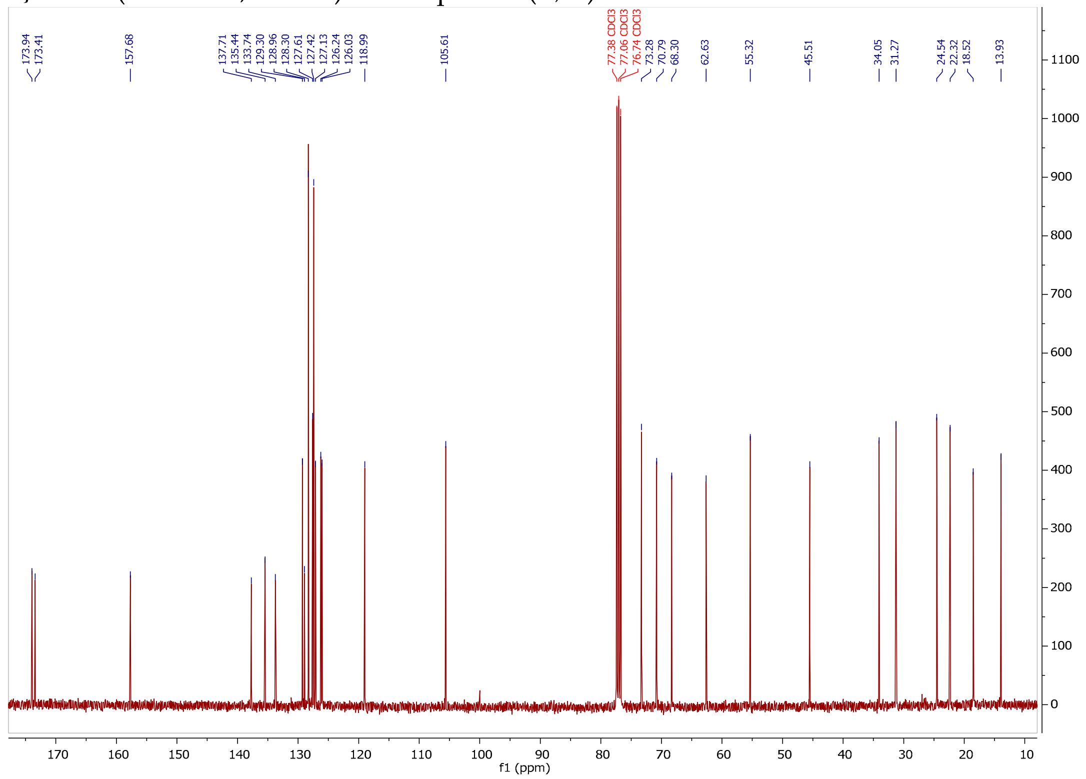

^{1}H-^{1}H COSY spectrum of compound (*R*,*S'*)-**7a**

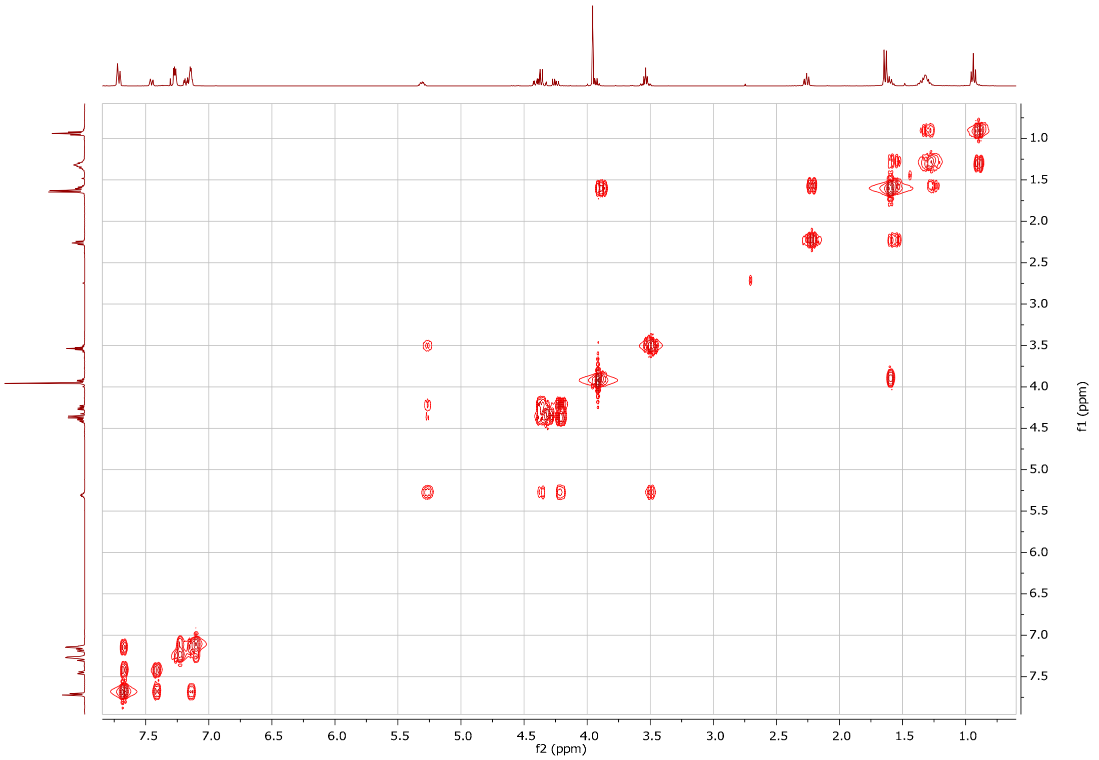

^{13}C-^{1}H HSQC spectrum of compound (*R*,*S'*)-**7a**

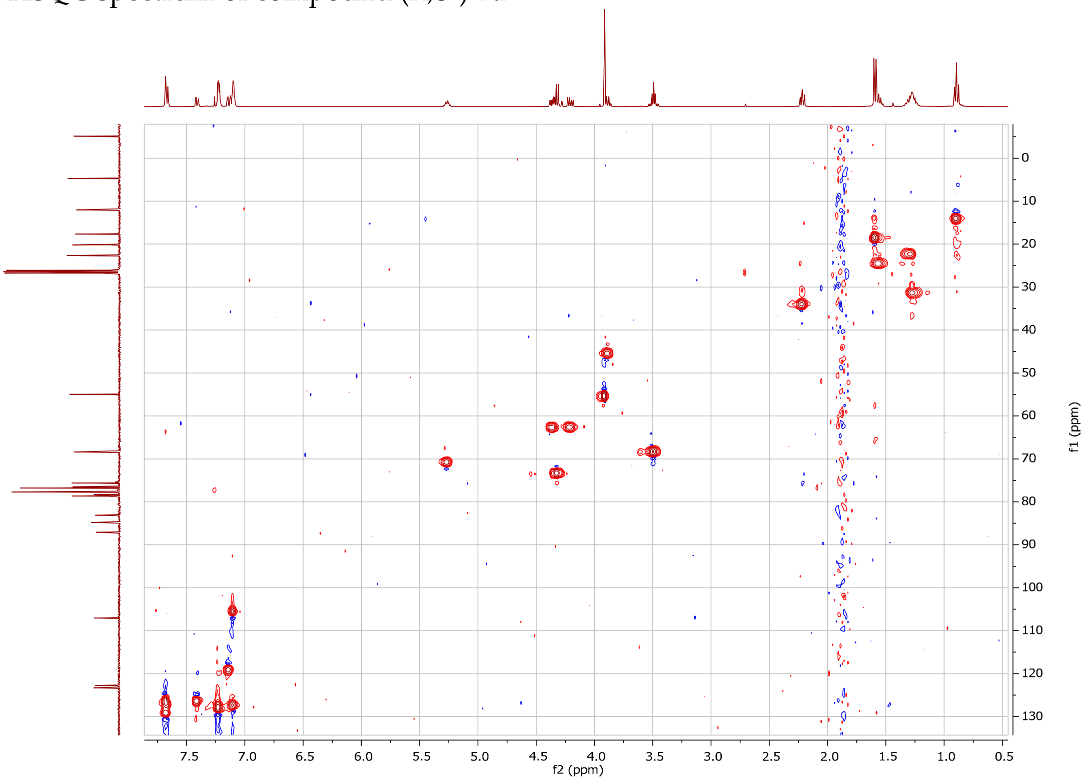

^{1}H NMR (400 MHz, $CDCl_3$) of compound (*S*,*S′*)-**7a**

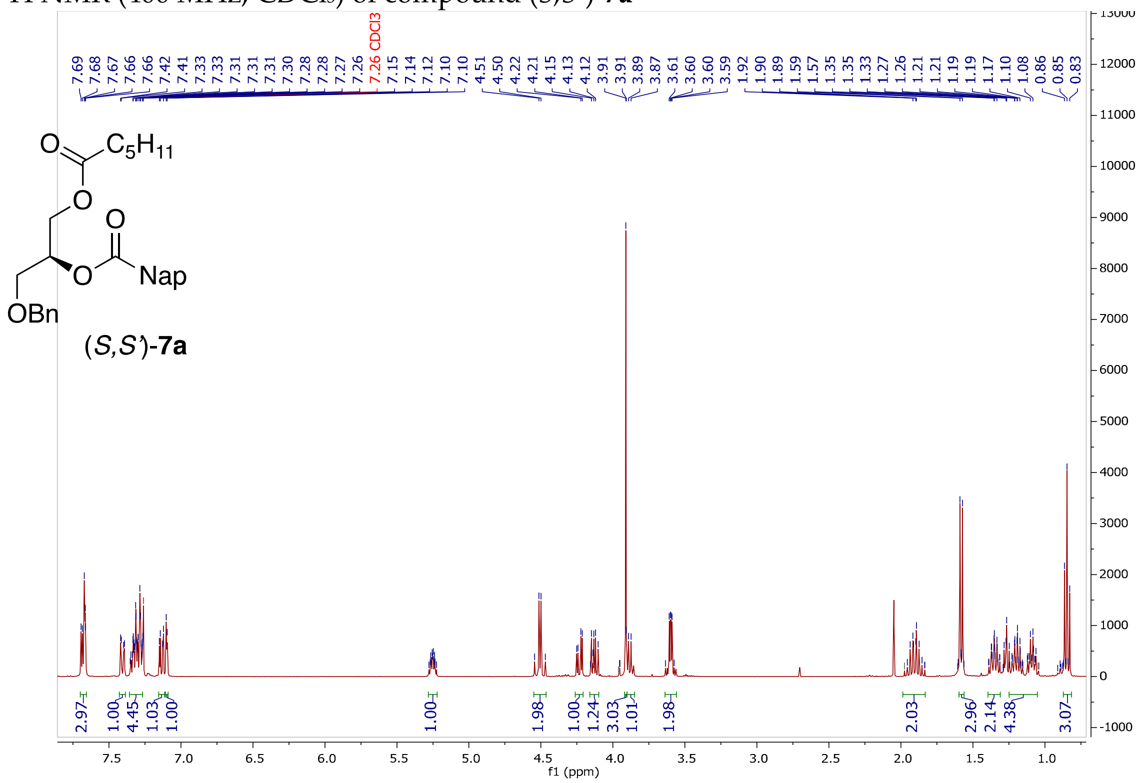

^{13}C{H} NMR (101 MHz, $CDCl_3$) of compound (*S*,*S′*)-**7a**

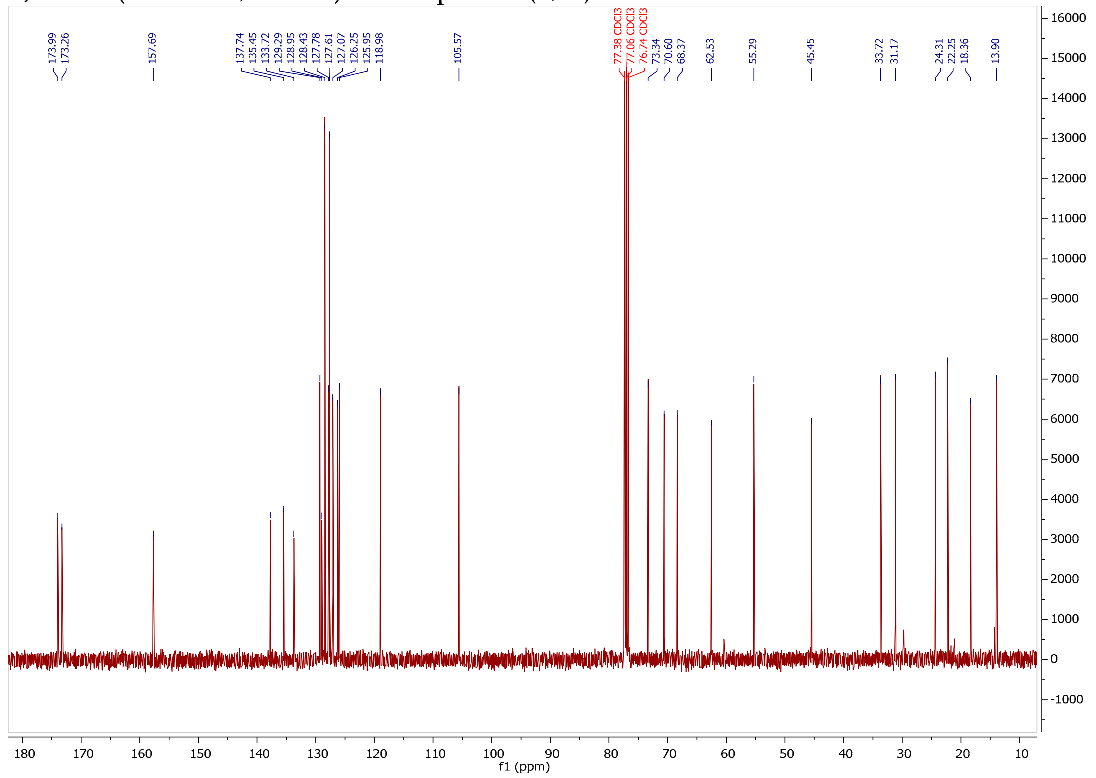

^{1}H-^{1}H COSY spectrum of compound (*S*,*S′*)-**7a**

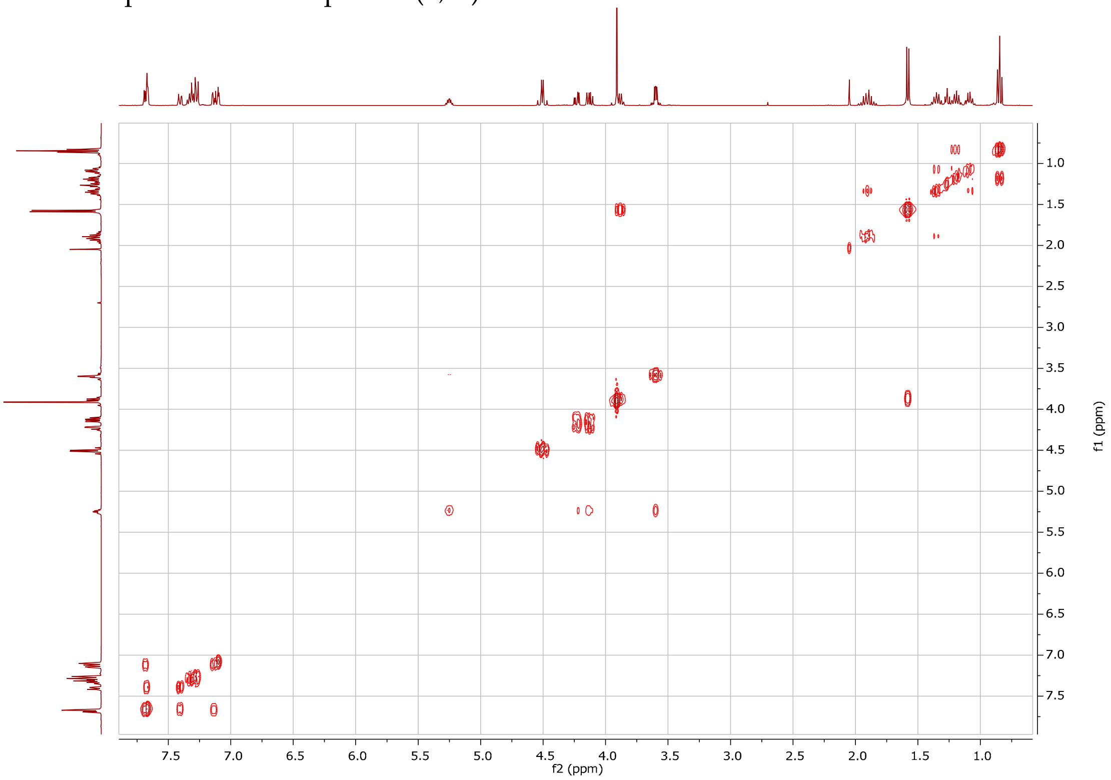

^{13}C-^{1}H HSQC spectrum of compound (*S*,*S′*)-**7a**

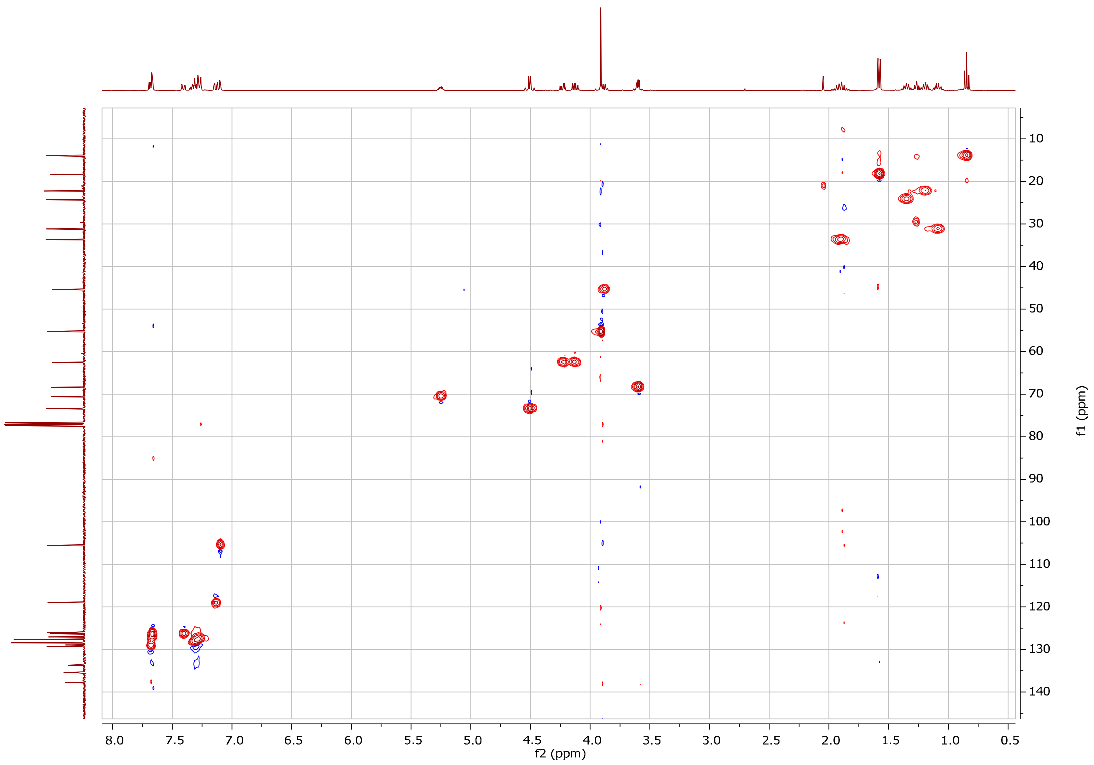

^{1}H NMR (400 MHz, CDCl$_3$) of compound (*R*,*S′*)-**8a**

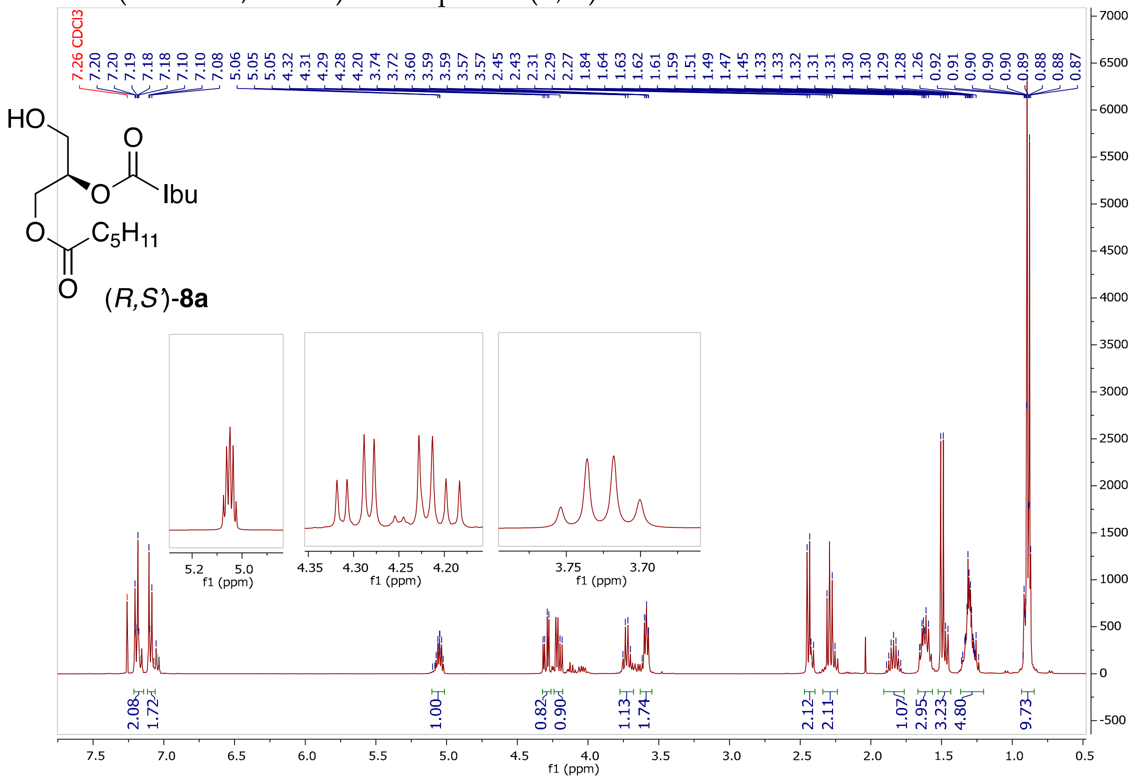

^{13}C{H} NMR (101 MHz, CDCl$_3$) of compound (*R*,*S′*)-**8a**

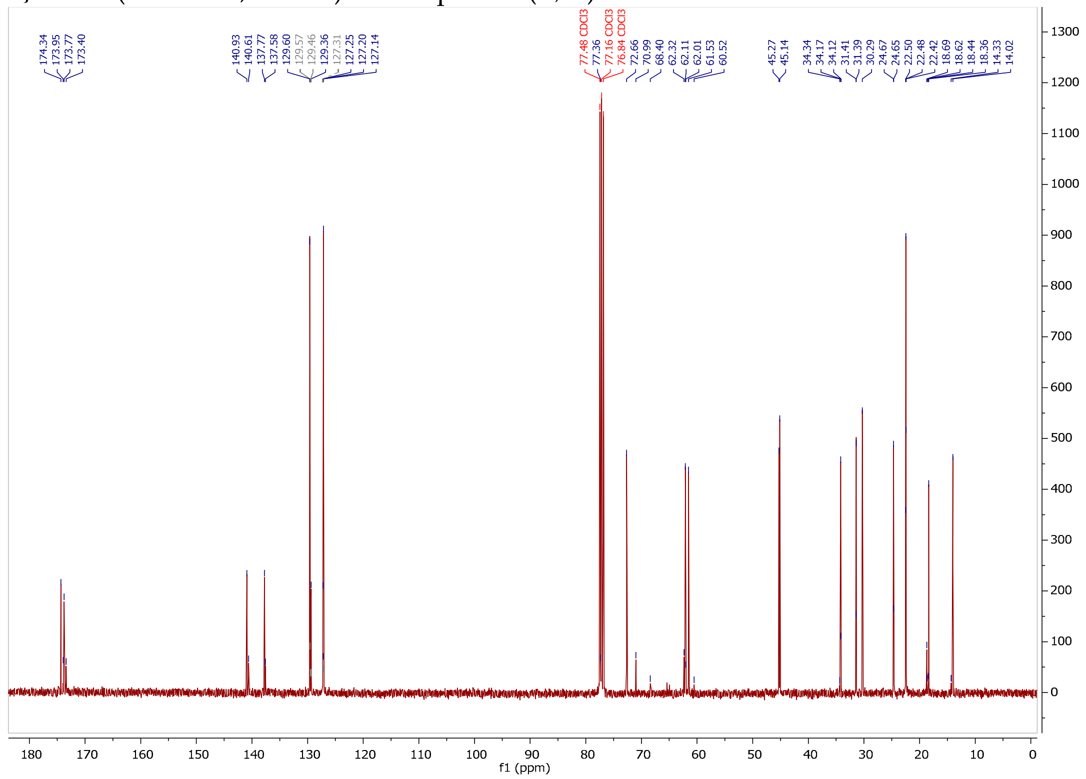

^{1}H-^{1}H COSY spectrum of compound (*R*,*S*′)-**8a**

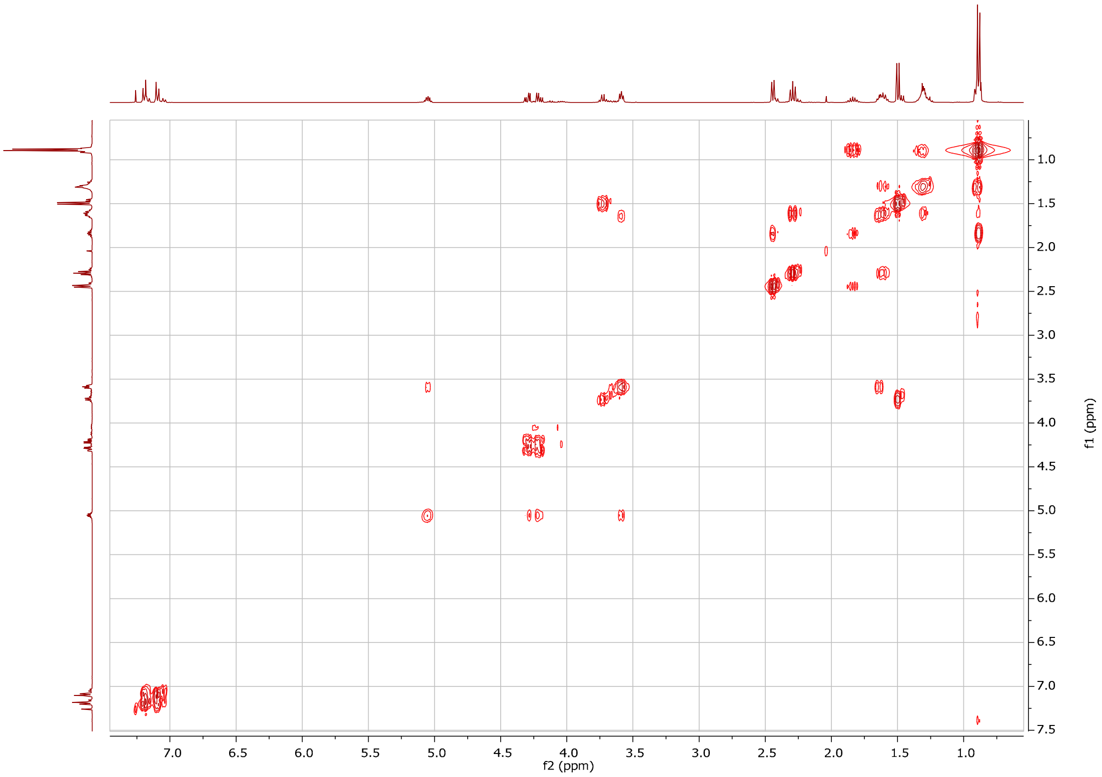

^{13}C-^{1}H HSQC spectrum of compound (*R*,*S*′)-**8a**

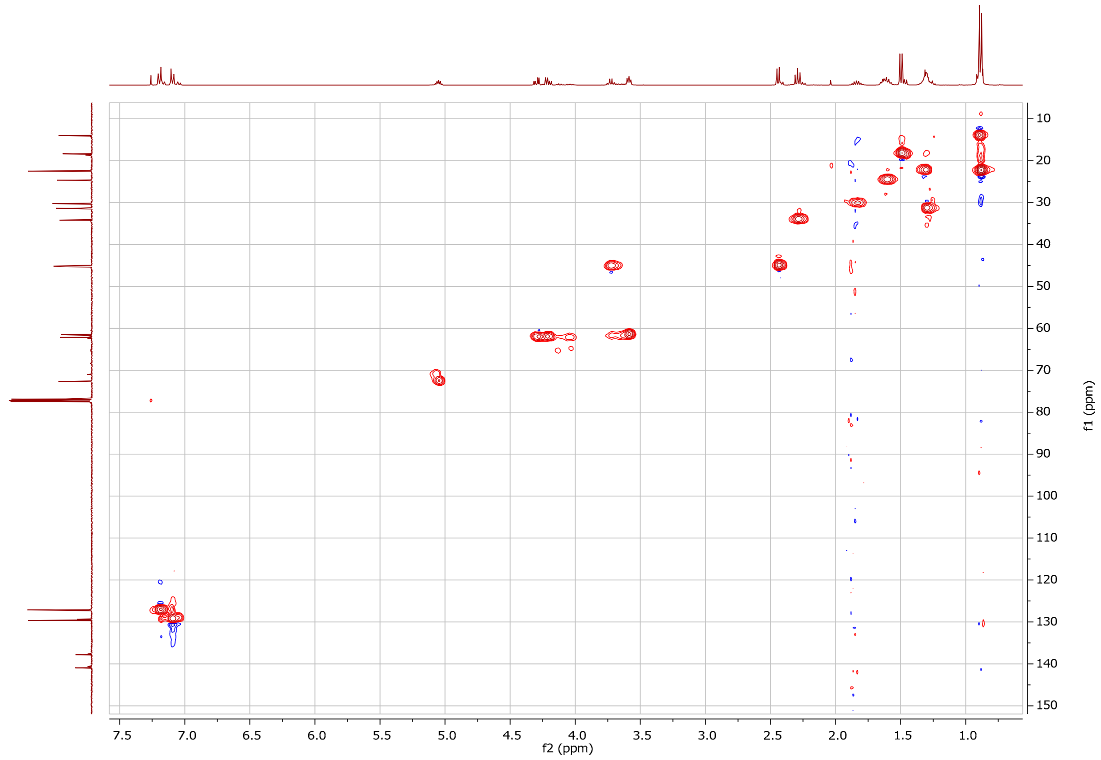

^{1}H NMR (400 MHz, $CDCl_3$) of compound (*S*,*S′*)-**8a**

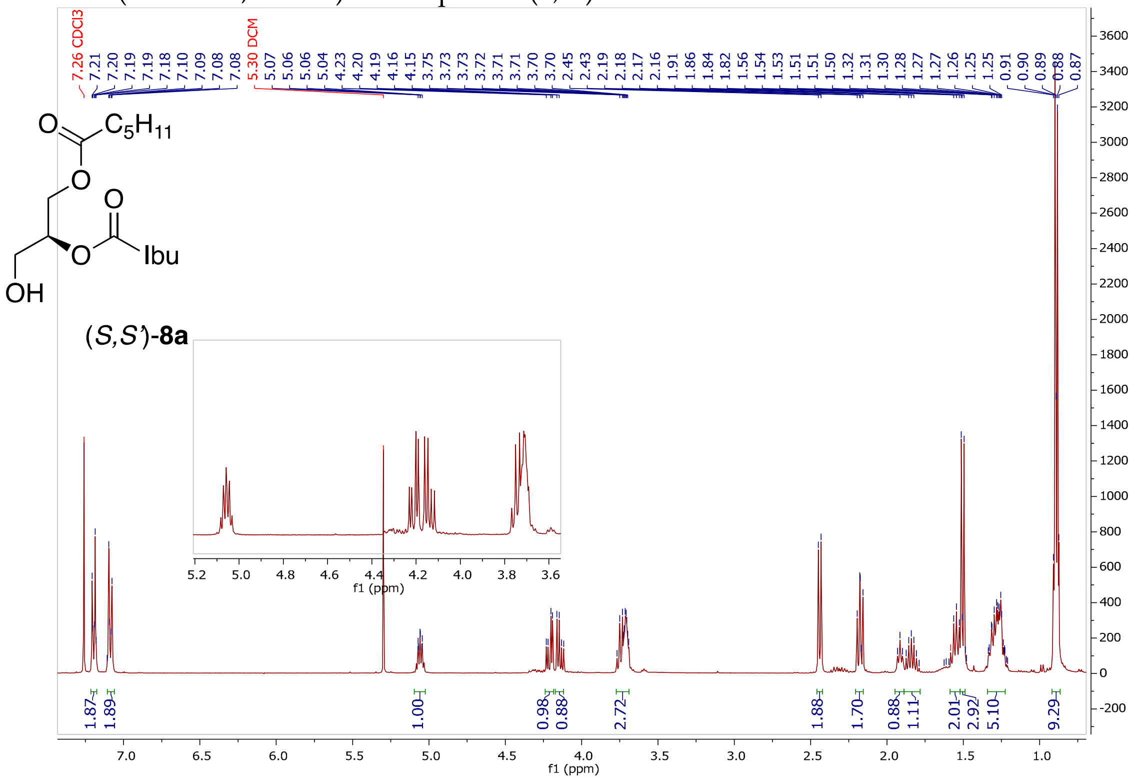

^{13}C{H} NMR (101 MHz, $CDCl_3$) of compound (*S*,*S′*)-**8a**

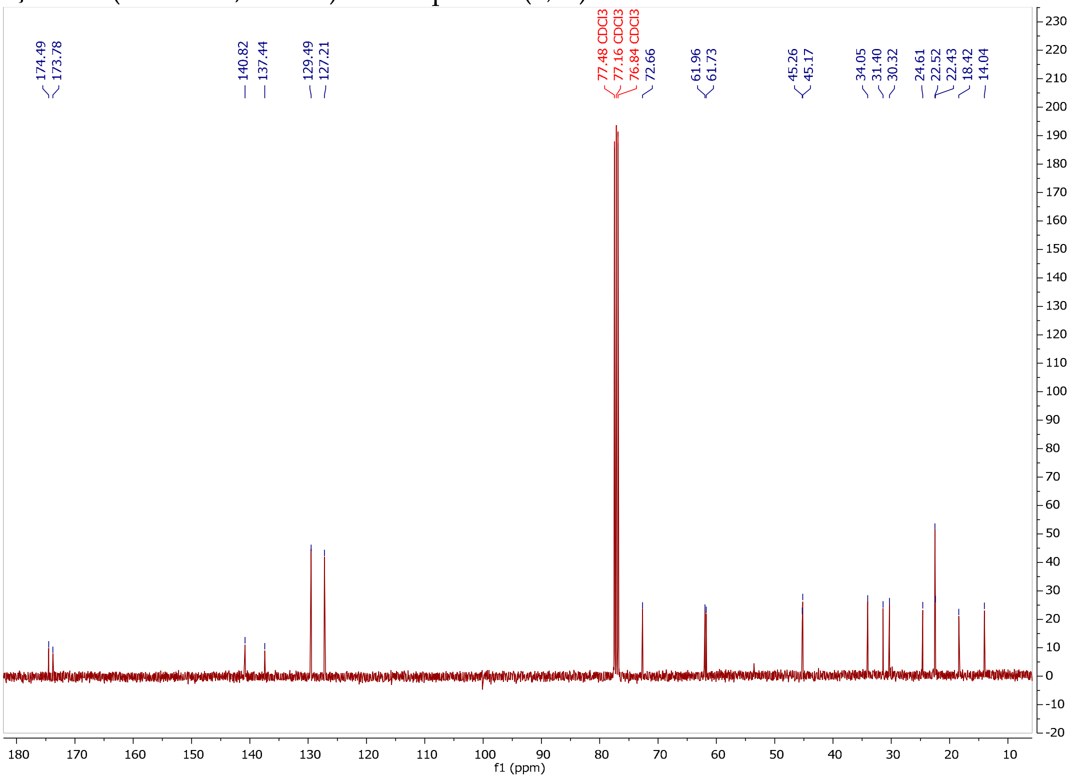

^{1}H-^{1}H COSY spectrum of compound (*S*,*S′*)-**8a**

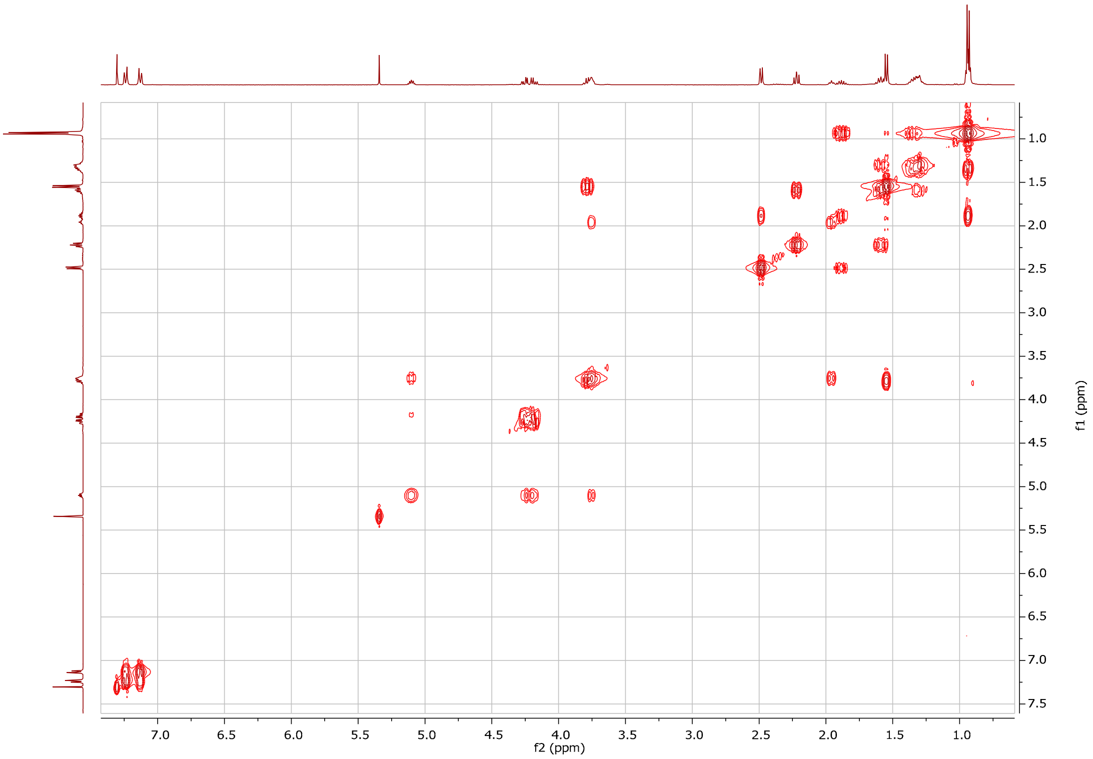

^{13}C-^{1}H HSQC spectrum of compound (*S*,*S′*)-**8a**

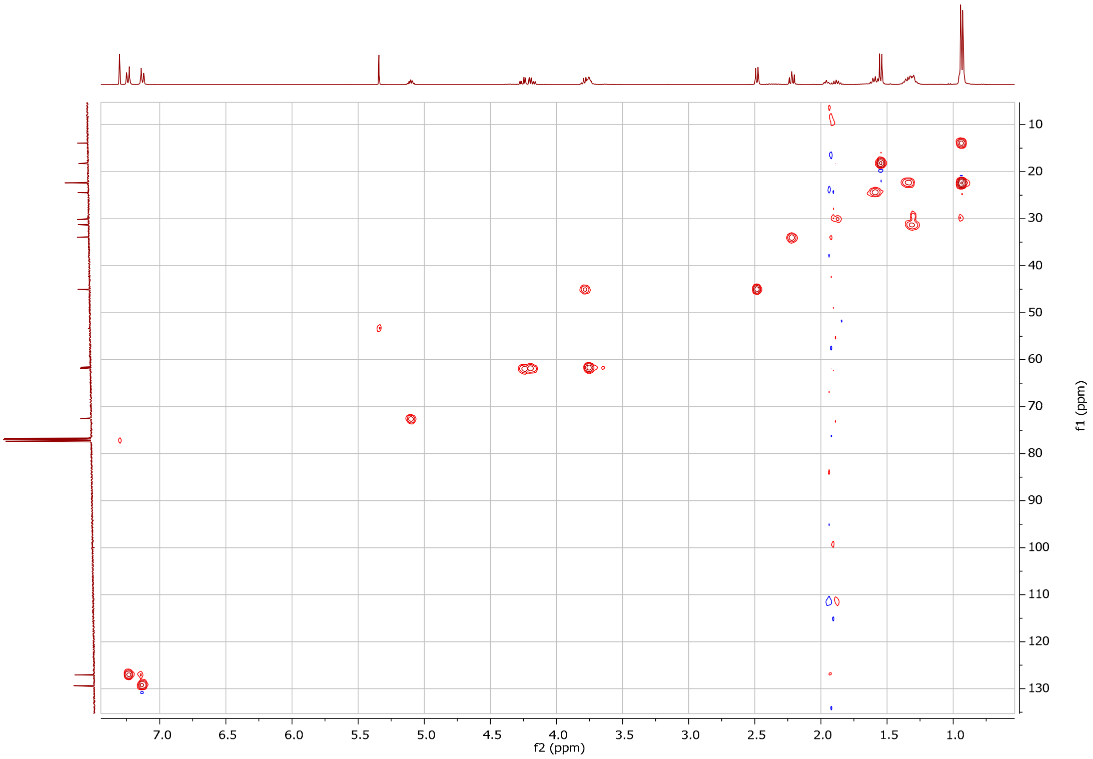

^{1}H NMR (400 MHz, $CDCl_3$) of compound (*R*,*S′*)-**9a**

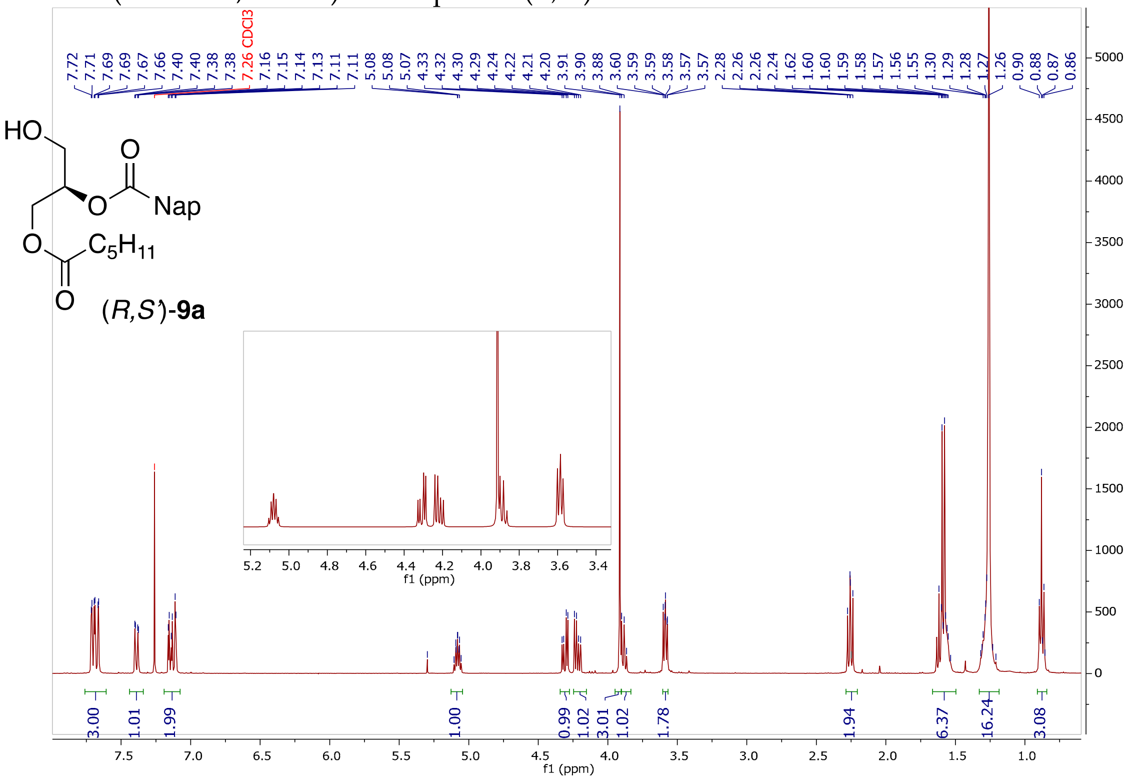

^{13}C{H} NMR (101 MHz, $CDCl_3$) of compound (*R*,*S′*)-**9a**

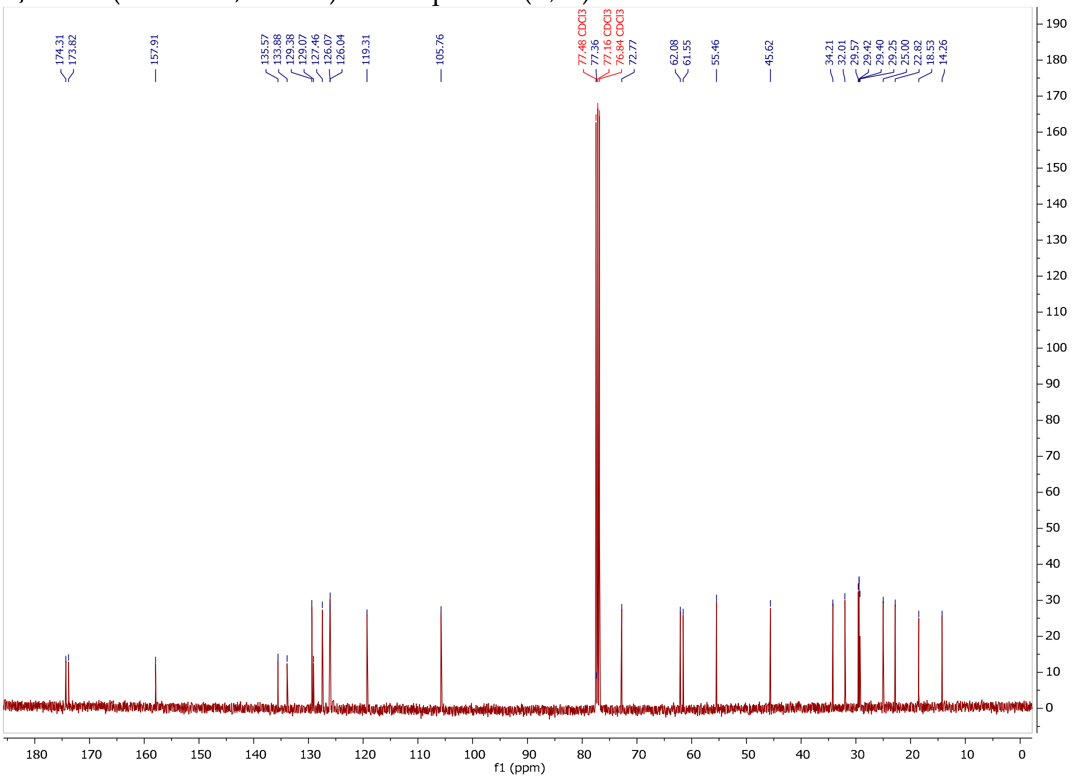

^{1}H-^{1}H COSY spectrum of compound (*R,S′*)-**9a**

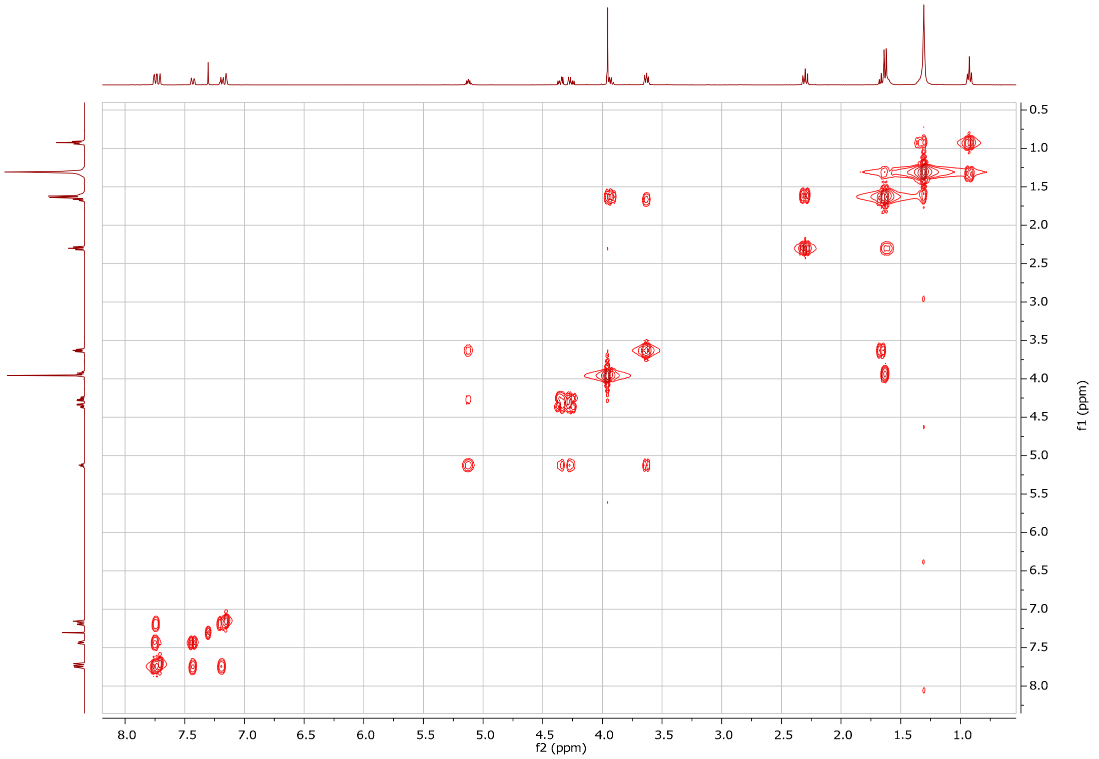

^{13}C-^{1}H HSQC spectrum of compound (*R,S′*)-**9a**

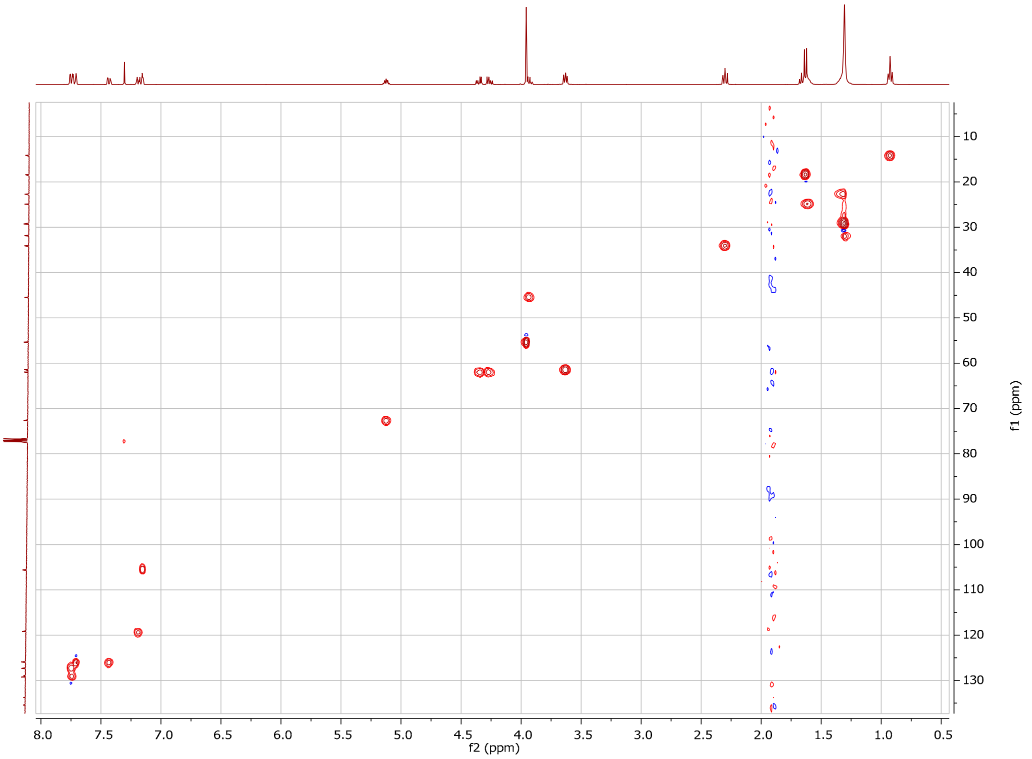

^{1}H NMR (400 MHz, $CDCl_3$) of compound (*S*,*S'*)-**9a**

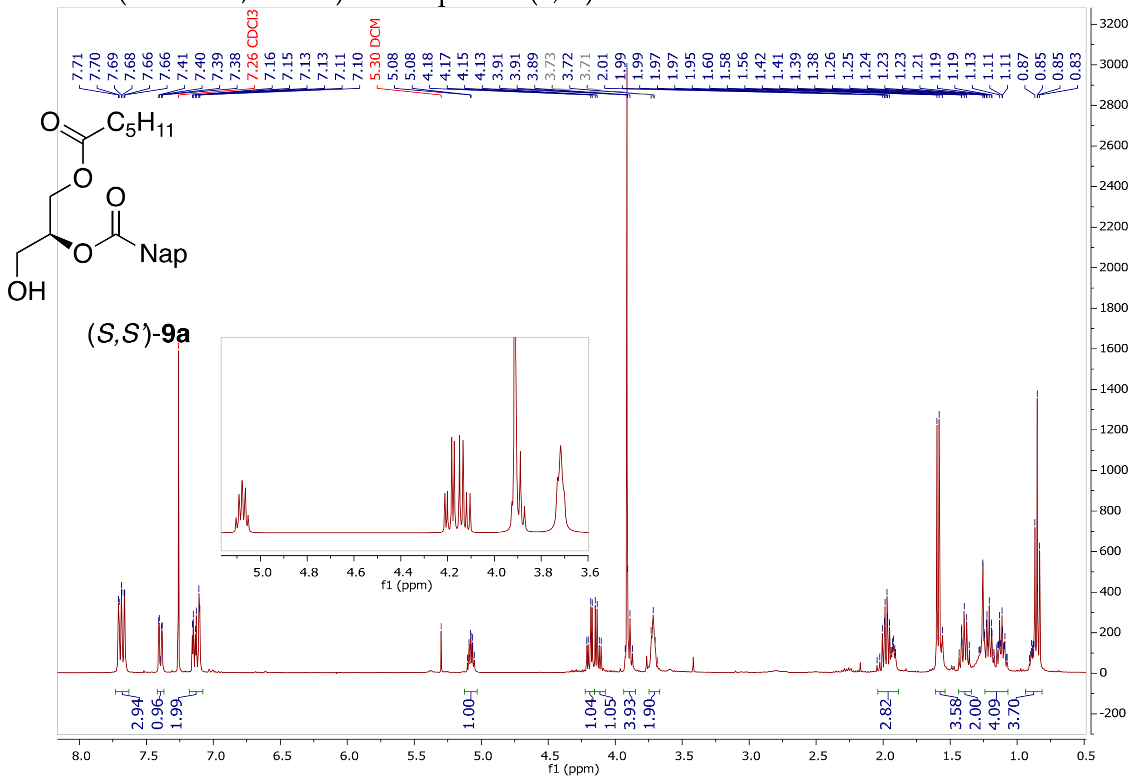

^{13}C{H} NMR (101 MHz, $CDCl_3$) of compound (*S*,*S'*)-**9a**

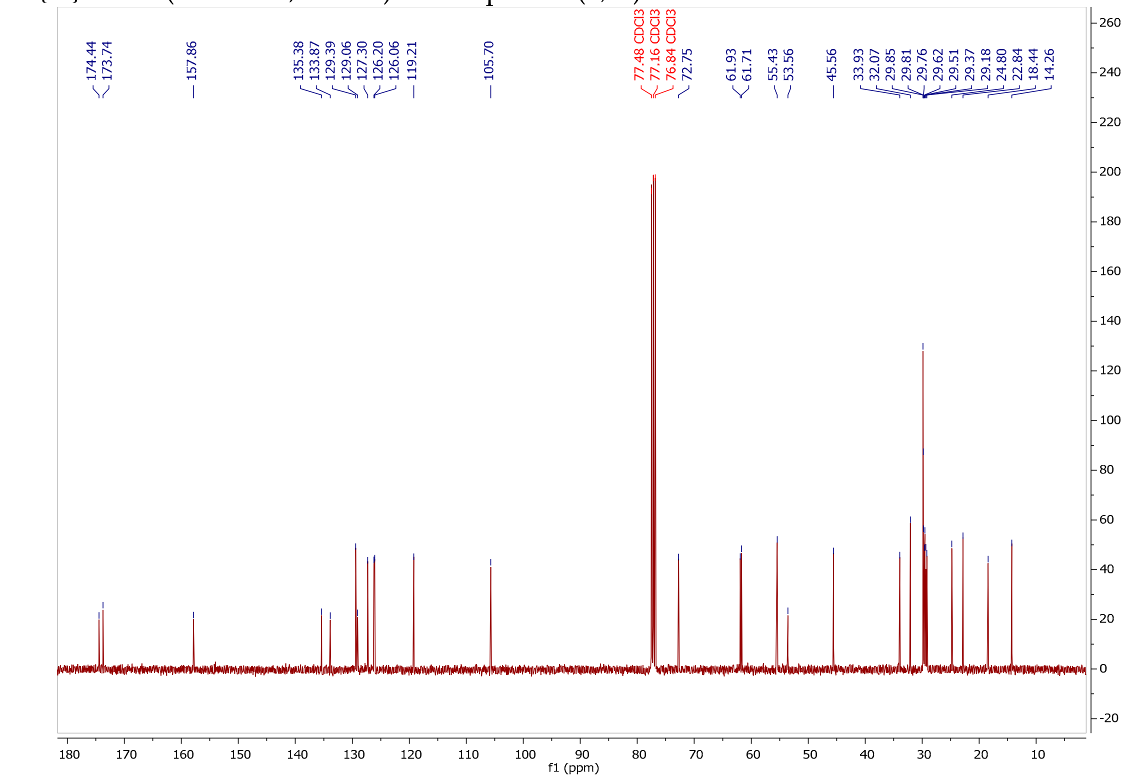

^{1}H-^{1}H COSY spectrum of compound (*S*,*S′*)-**9a**

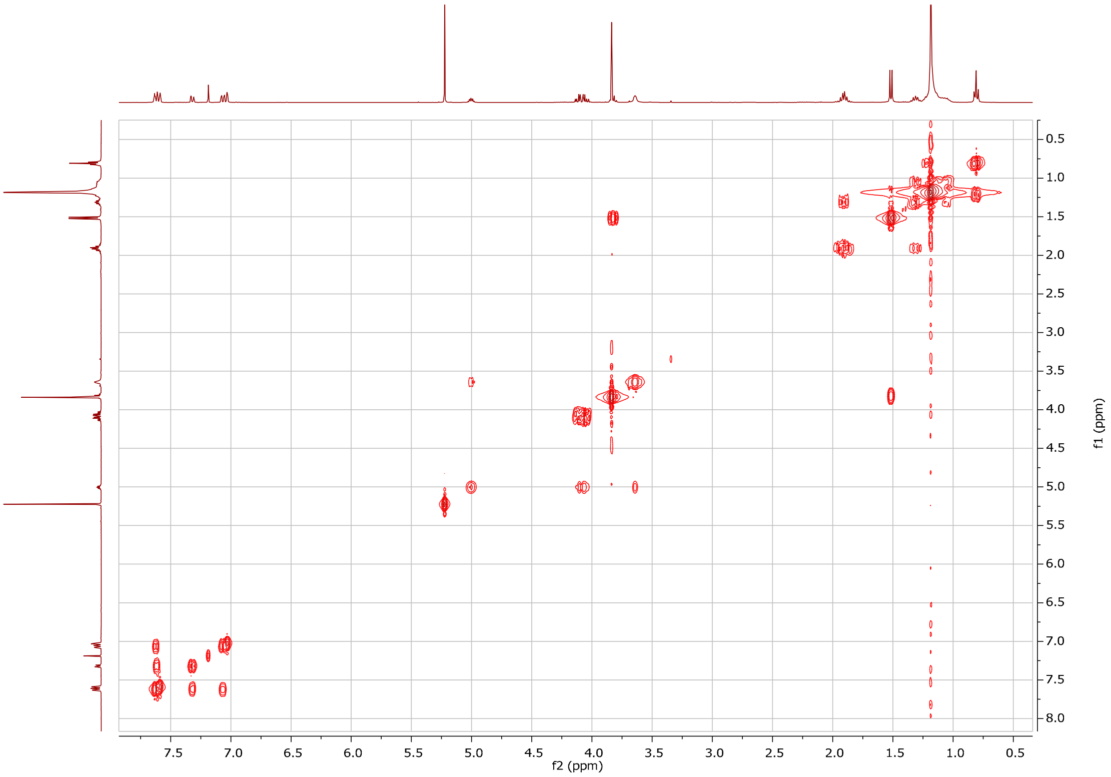

^{13}C-^{1}H HSQC spectrum of compound (*S*,*S′*)-**9a**

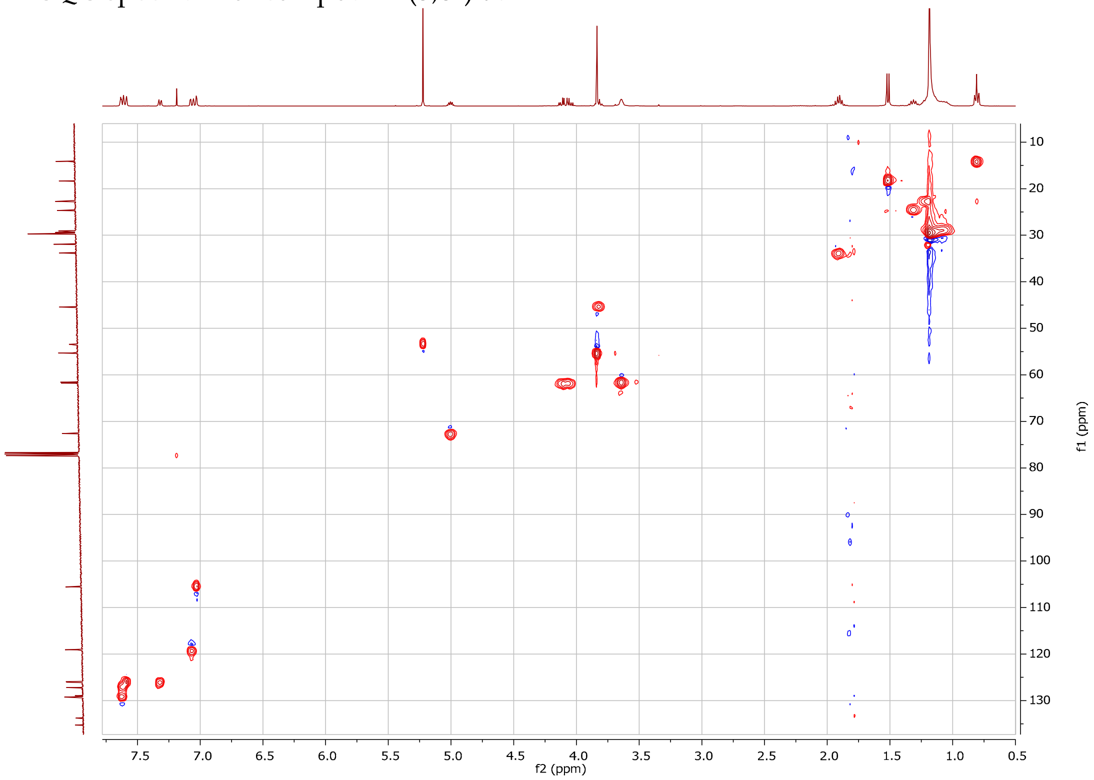

^{1}H NMR (400 MHz, $CDCl_3$) of compound (*S*,*S′*)-**10a**

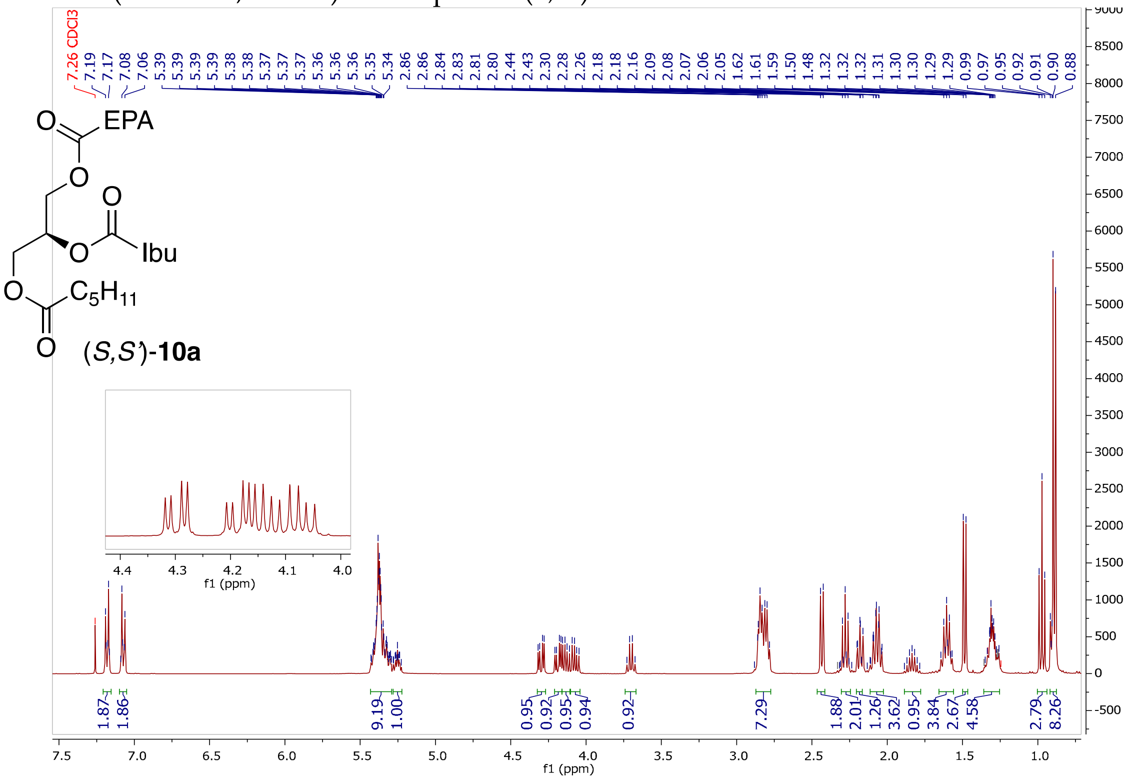

^{13}C{H} NMR (101 MHz, $CDCl_3$) of compound (*S*,*S′*)-**10a**

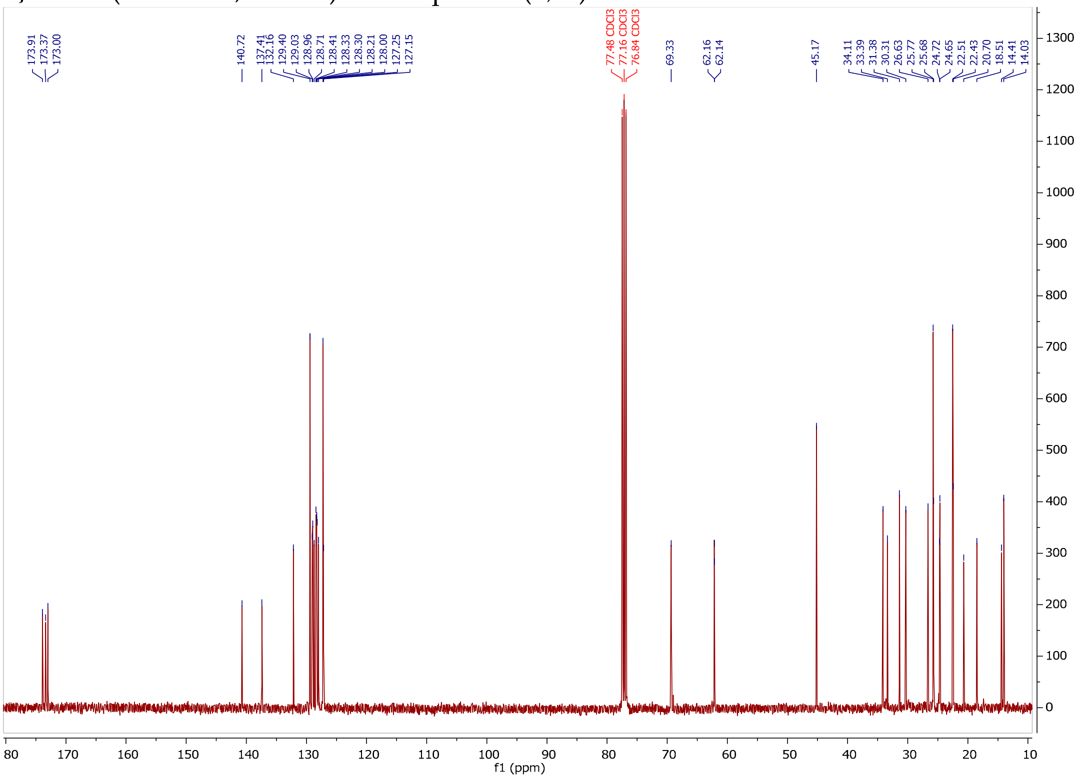

^{1}H-^{1}H COSY spectrum of compound (*S,S′*)-**10a**

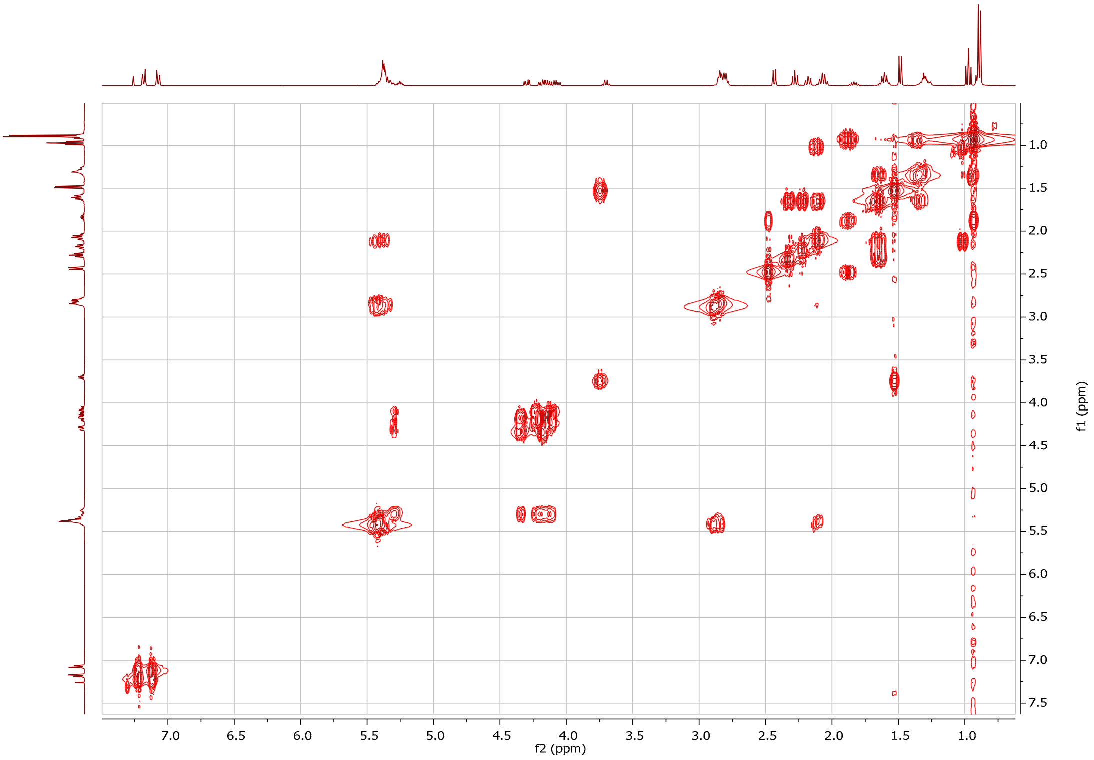

^{13}C-^{1}H HSQC spectrum of compound (*S,S′*)-**10a**

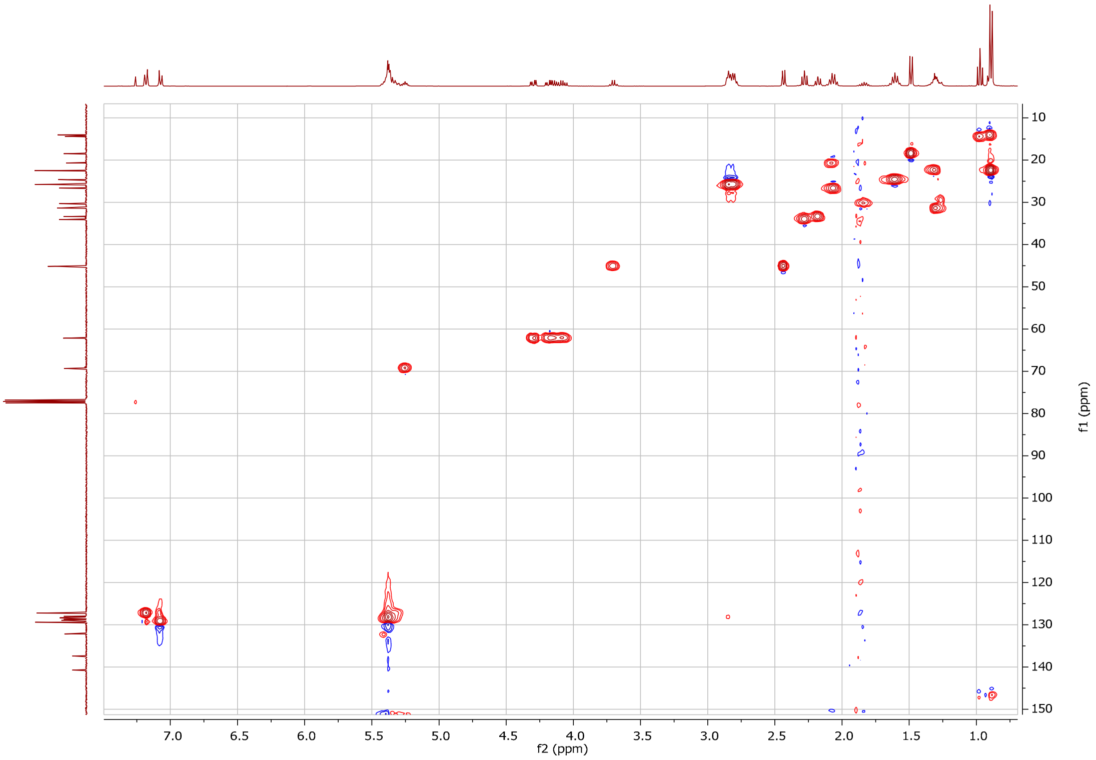

^{1}H NMR (400 MHz, $CDCl_3$) of compound (*R*,*S'*)-**10a**

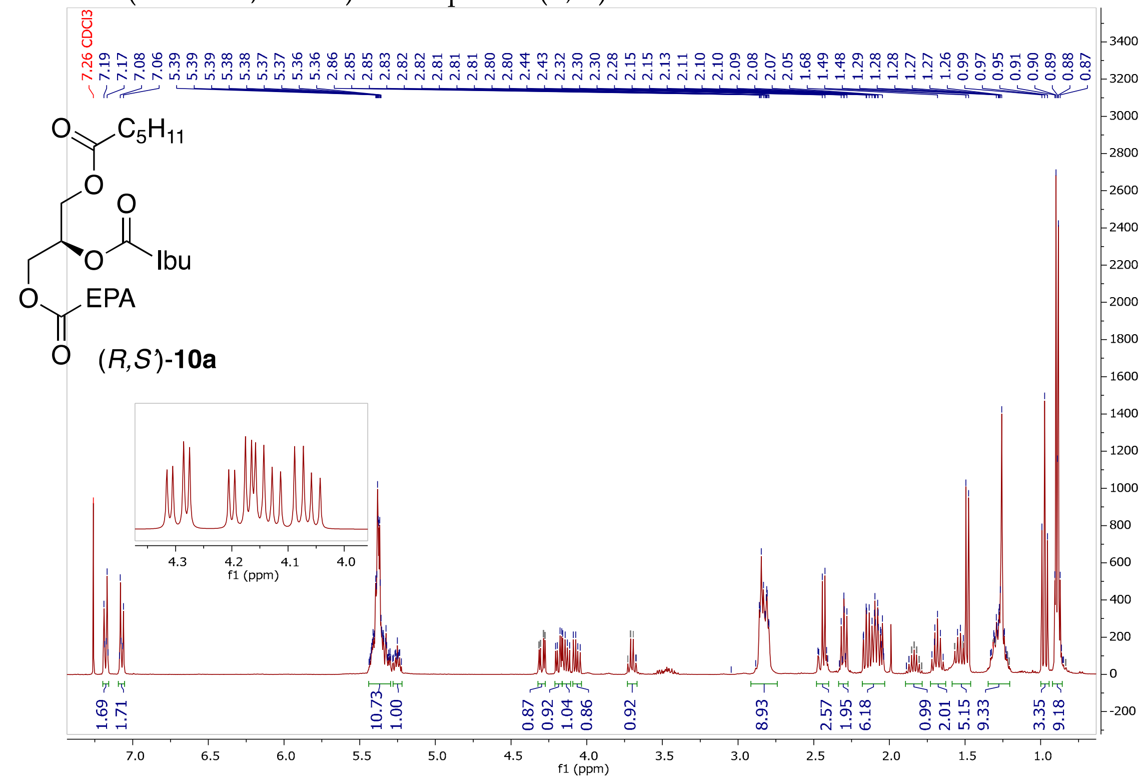

^{13}C{H} NMR (101 MHz, $CDCl_3$) of compound (*R*,*S'*)-**10a**

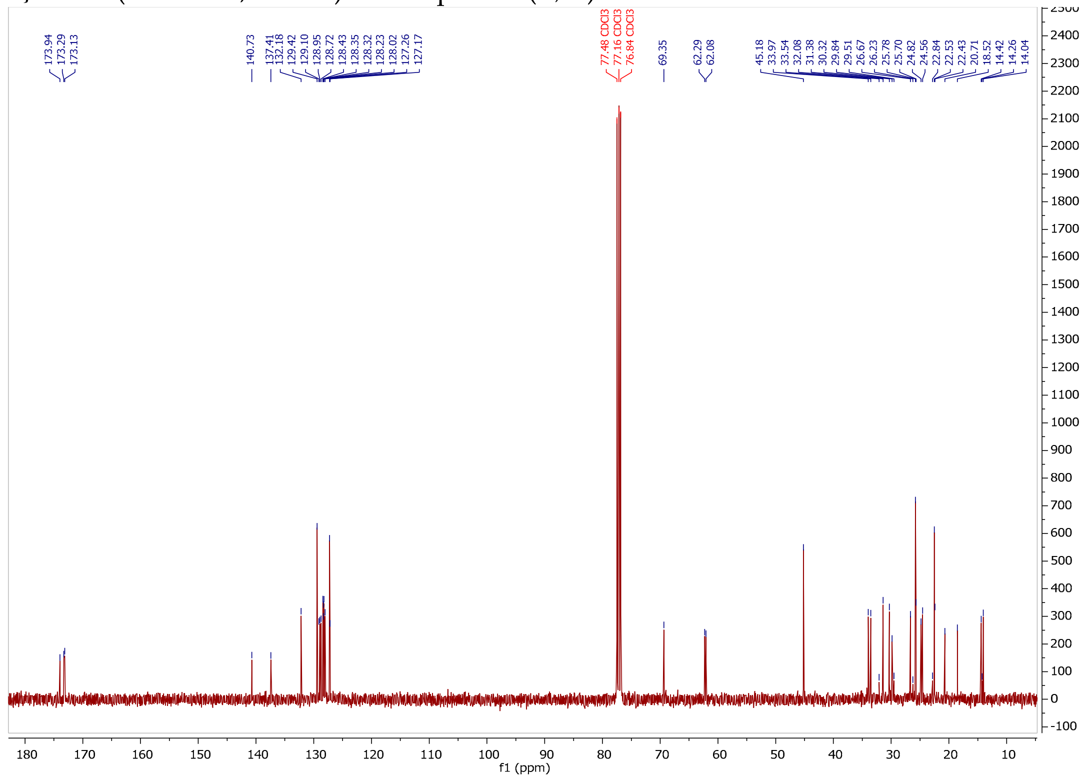

^{1}H-^{1}H COSY spectrum of compound (*R*,*S'*)-**10a**

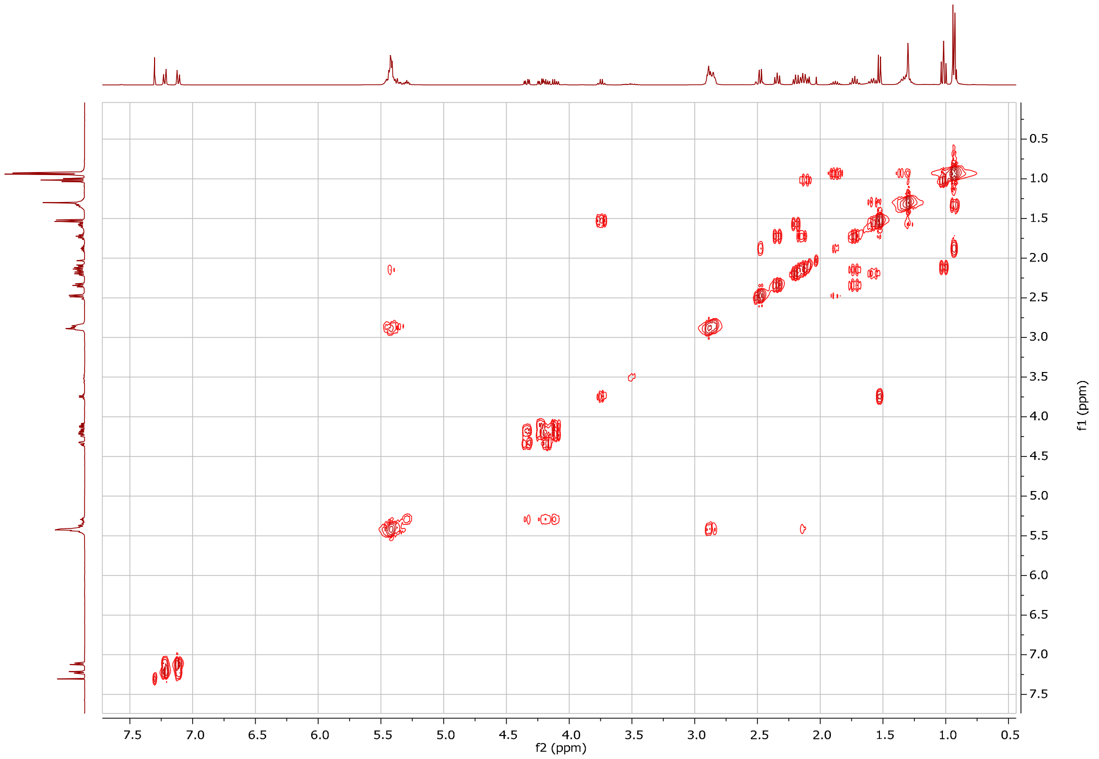

^{13}C-^{1}H HSQC spectrum of compound (*R*,*S'*)-**10a**

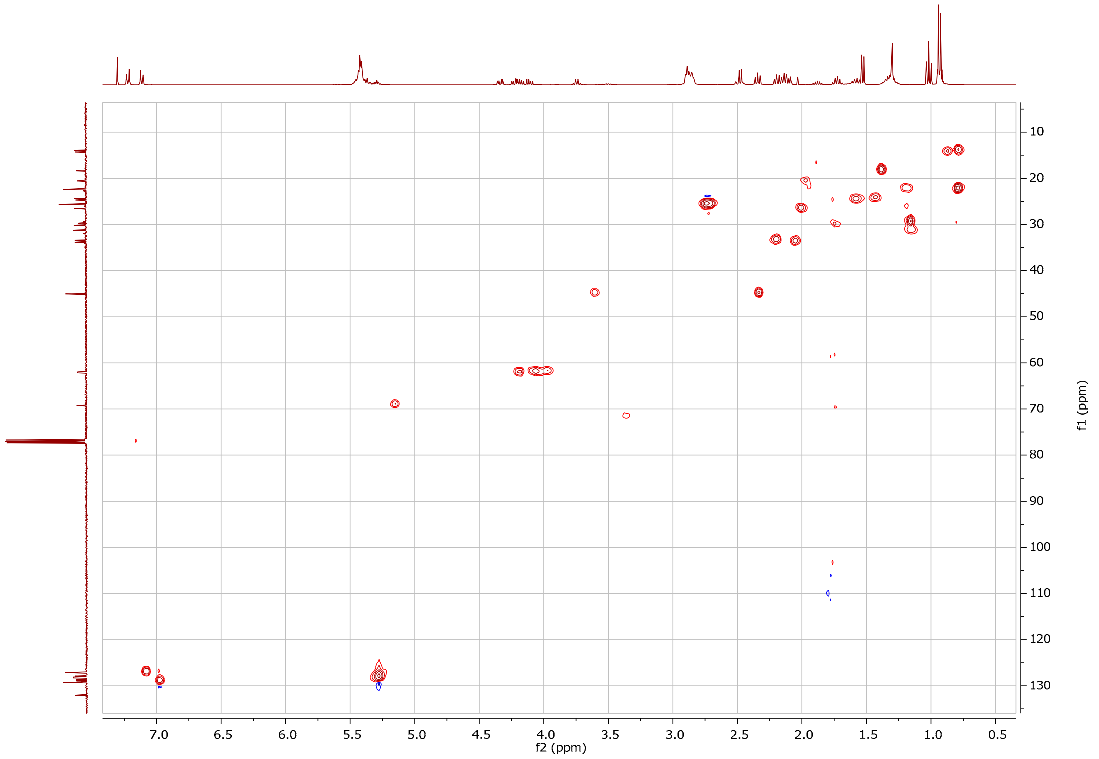

^{1}H NMR (400 MHz, CDCl3) of compound (*S*,*S′*)-**11a**

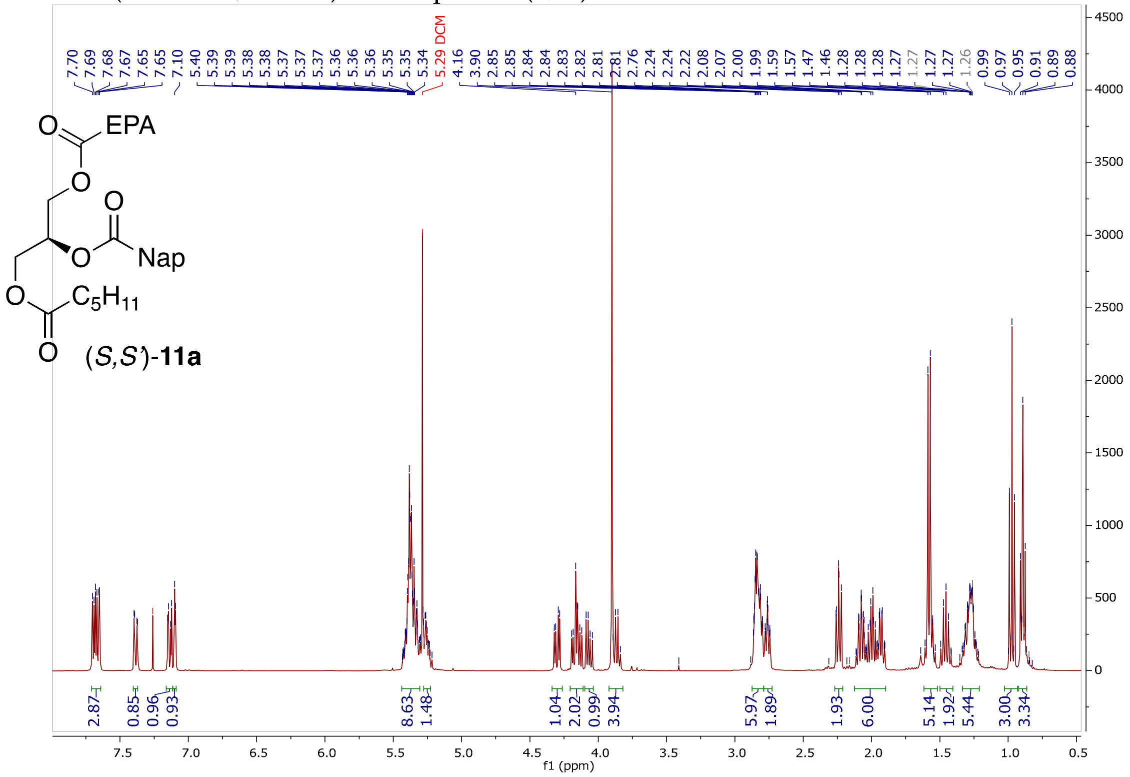

^{13}C{H} NMR (101 MHz, CDCl3) of compound (*S*,*S′*)-**11a**

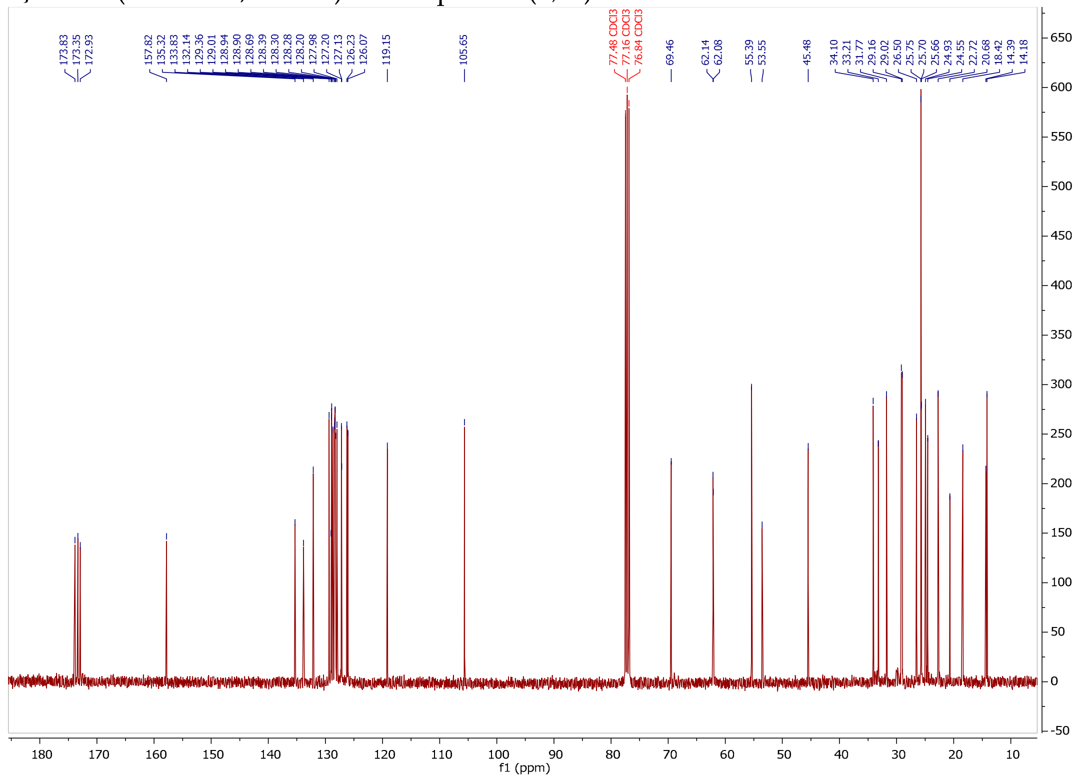

^{1}H-^{1}H COSY spectrum of compound (*S*,*S′*)-**11a**

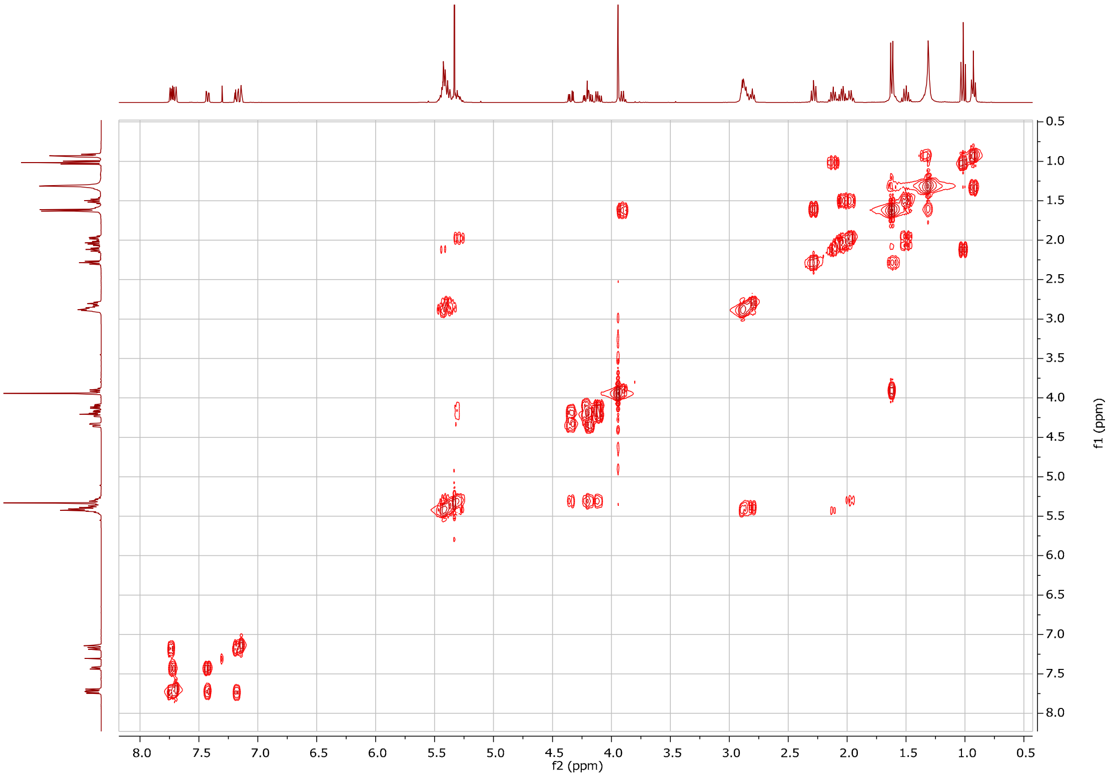

^{13}C-^{1}H HSQC spectrum of compound (*S*,*S′*)-**11a**

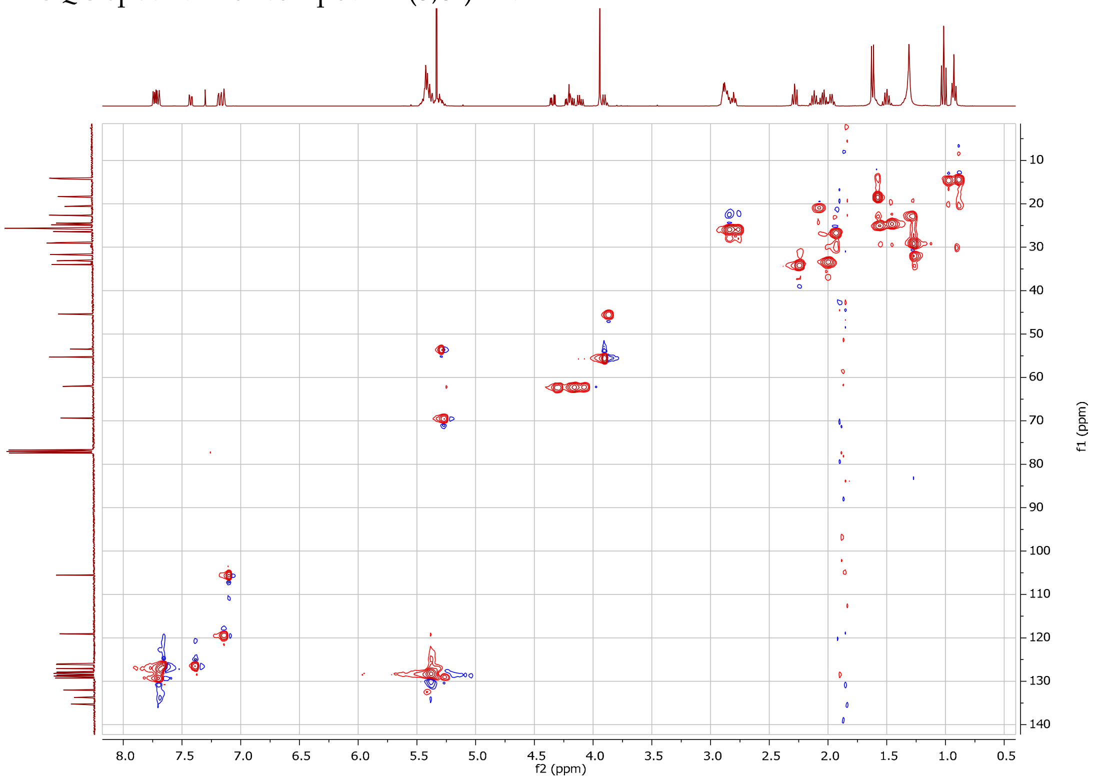

^{1}H NMR (400 MHz, $CDCl_3$) of compound (*R*,*S*′)-**11a**

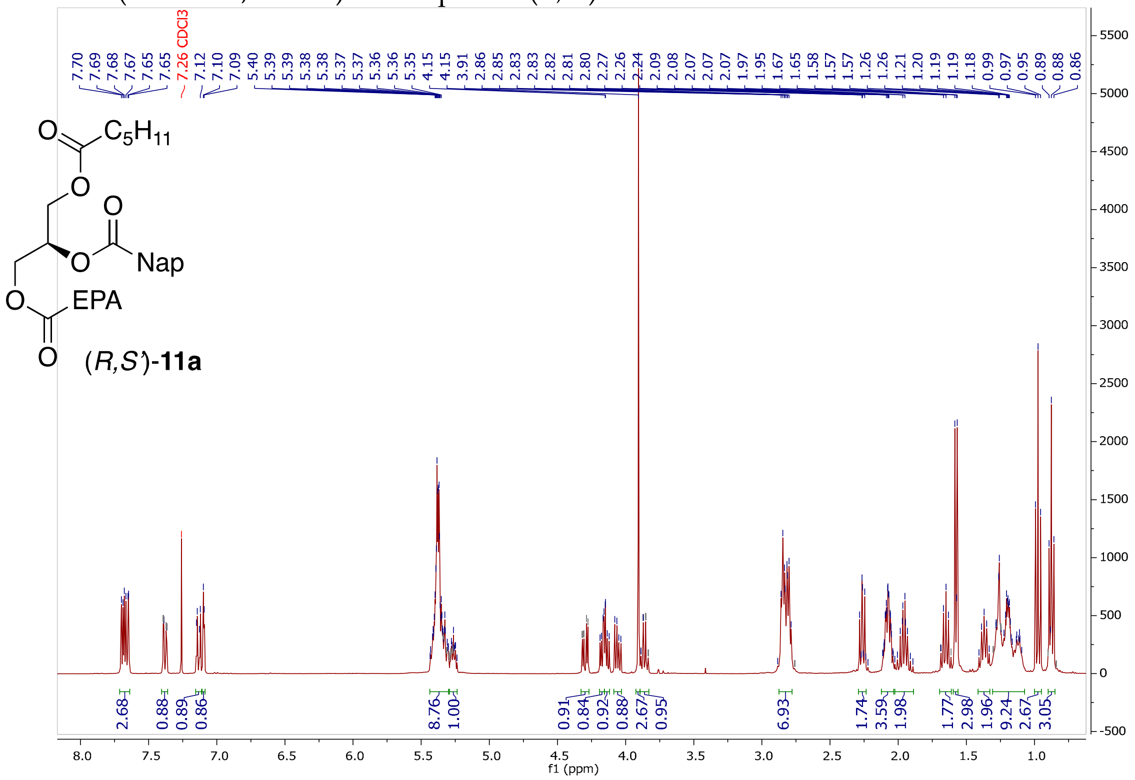

$^{13}C\{H\}$ NMR (101 MHz, $CDCl_3$) of compound (*R*,*S*′)-**11a**

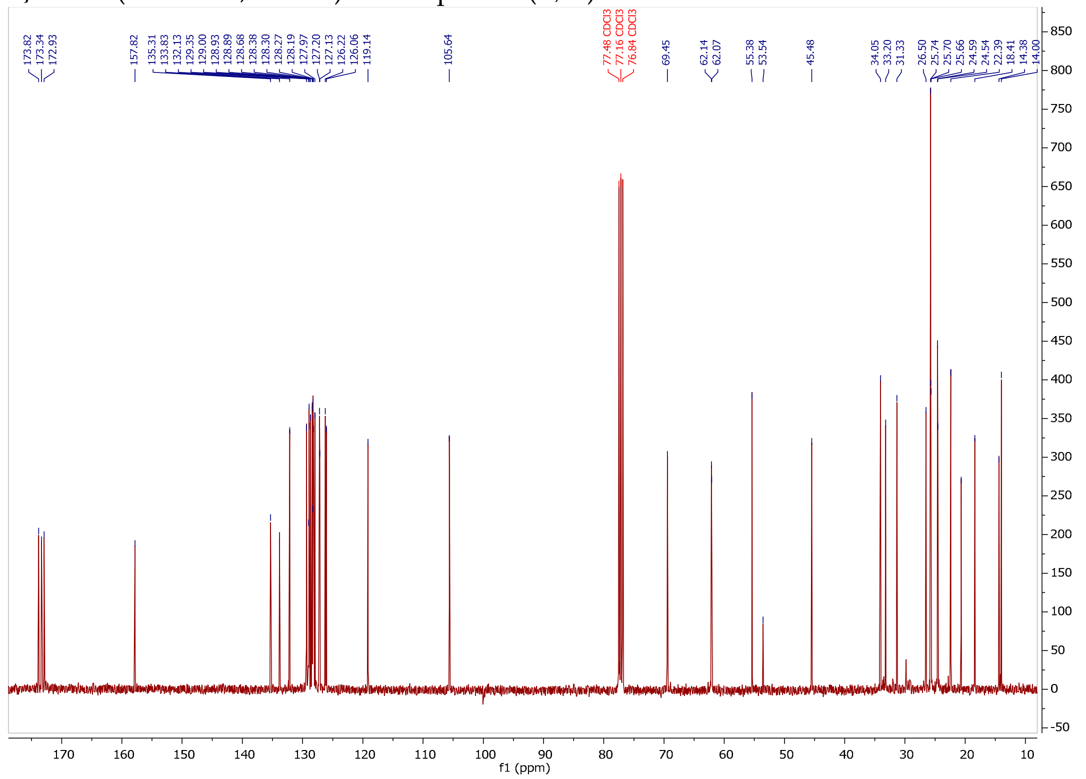

^{1}H-^{1}H COSY spectrum of compound (*R*,*S'*)-**11a**

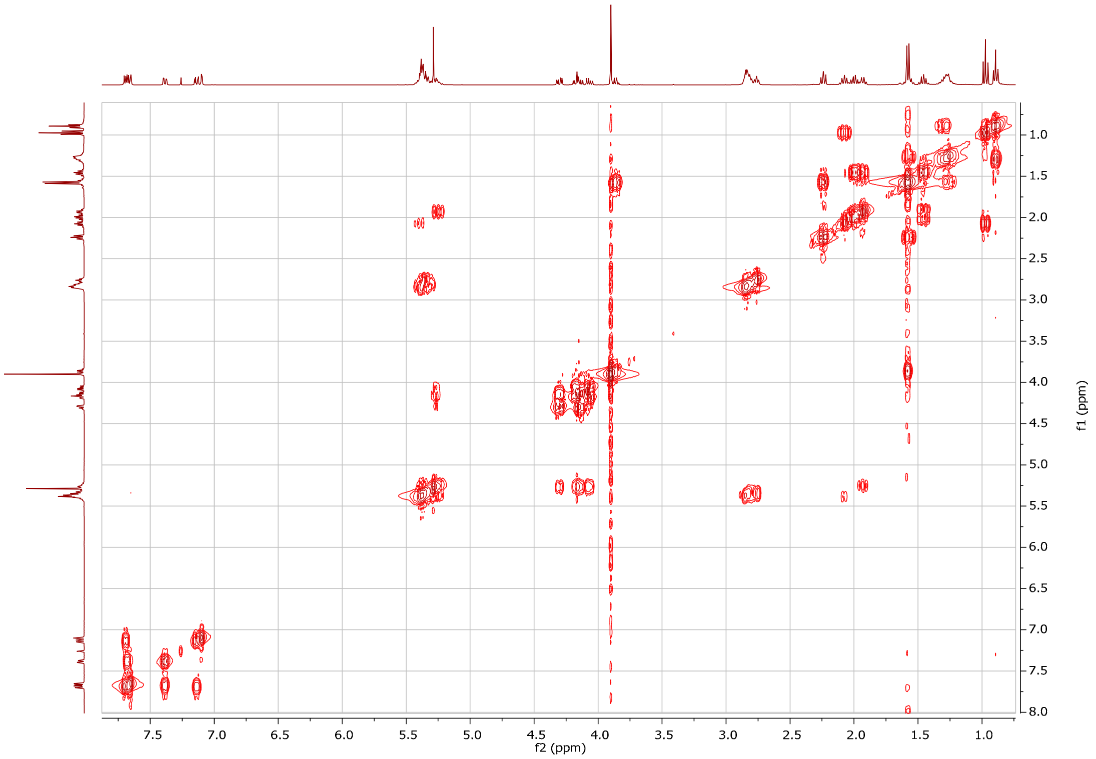

^{13}C-^{1}H HSQC spectrum of compound (*R*,*S'*)-**11a**

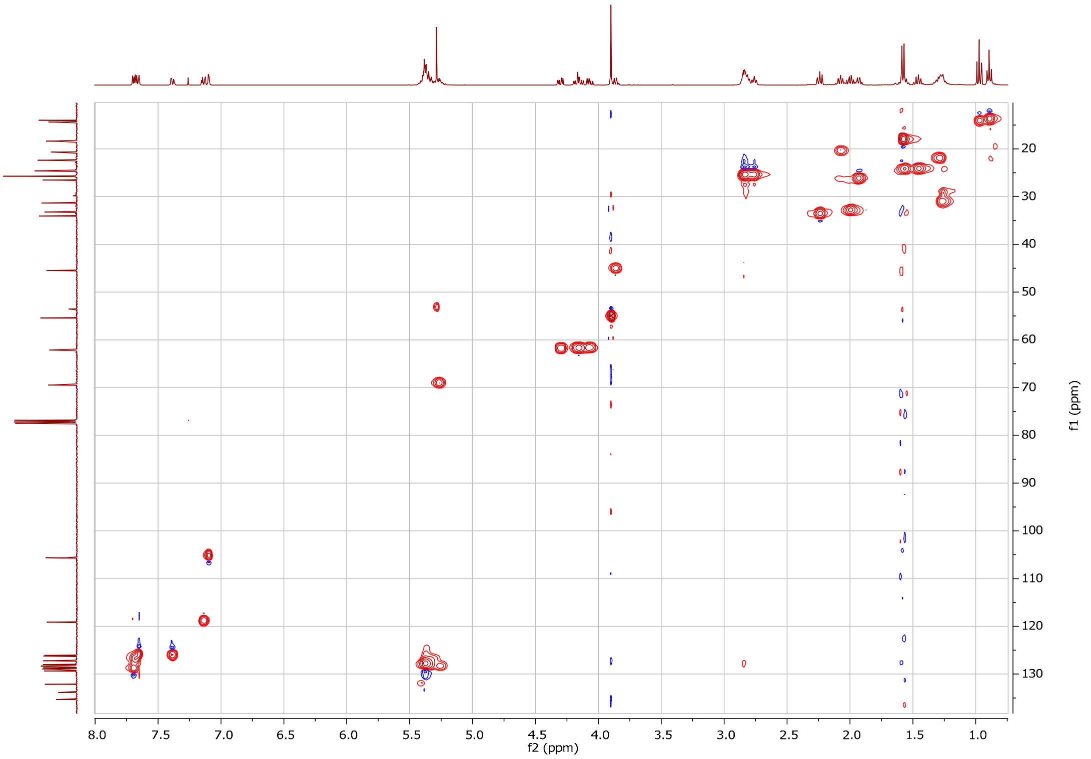

^{1}H NMR (400 MHz, $CDCl_3$) of compound (*S*,*S′*)-**12a**

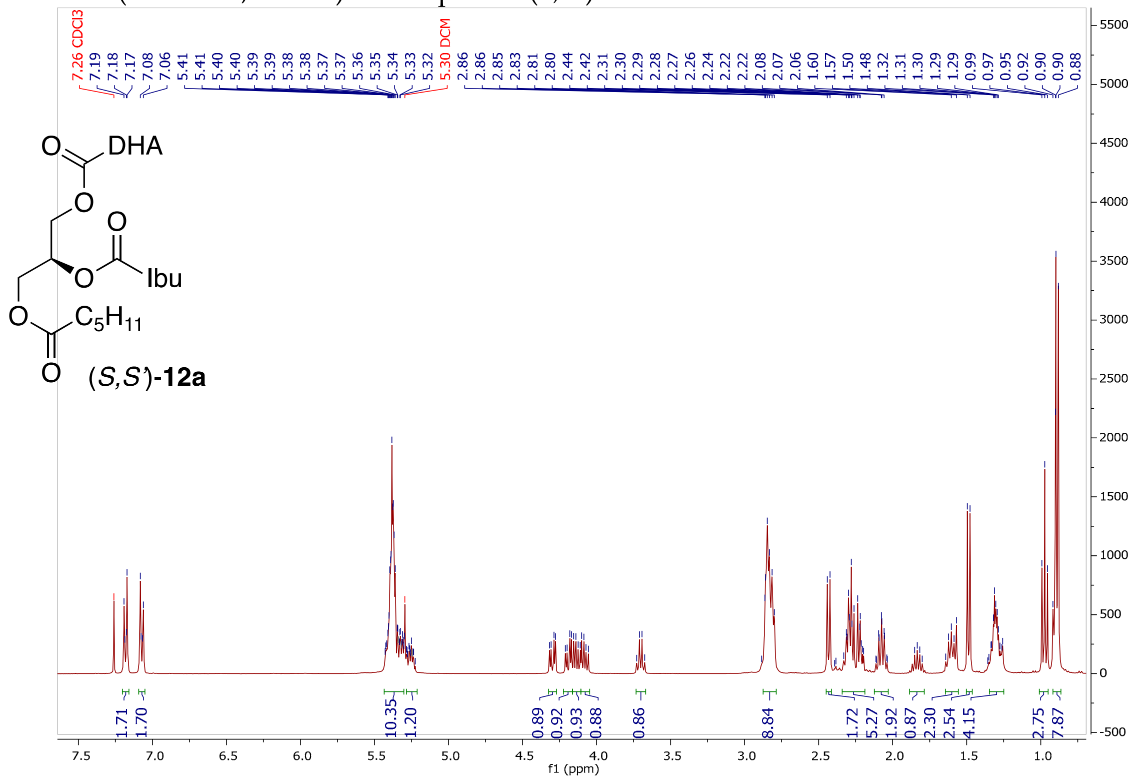

^{13}C{H} NMR (101 MHz, $CDCl_3$) of compound (*S*,*S′*)-**12a**

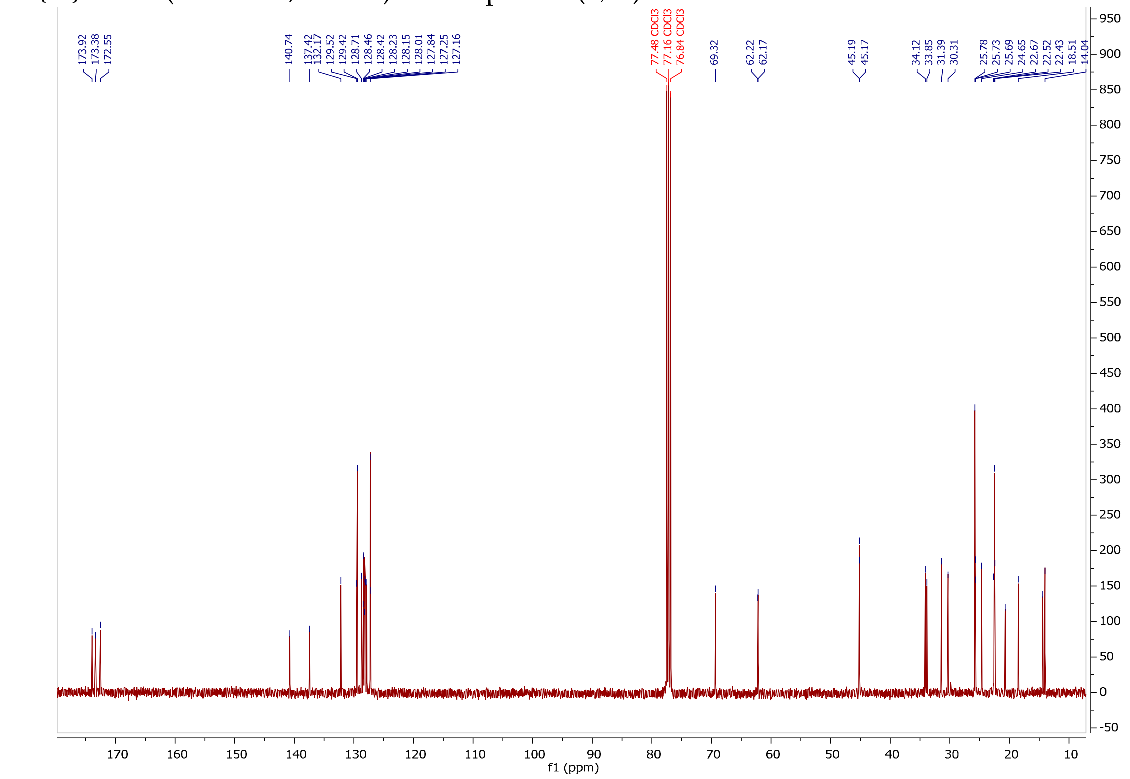

^{1}H-^{1}H COSY spectrum of compound (*S,S′*)-**12a**

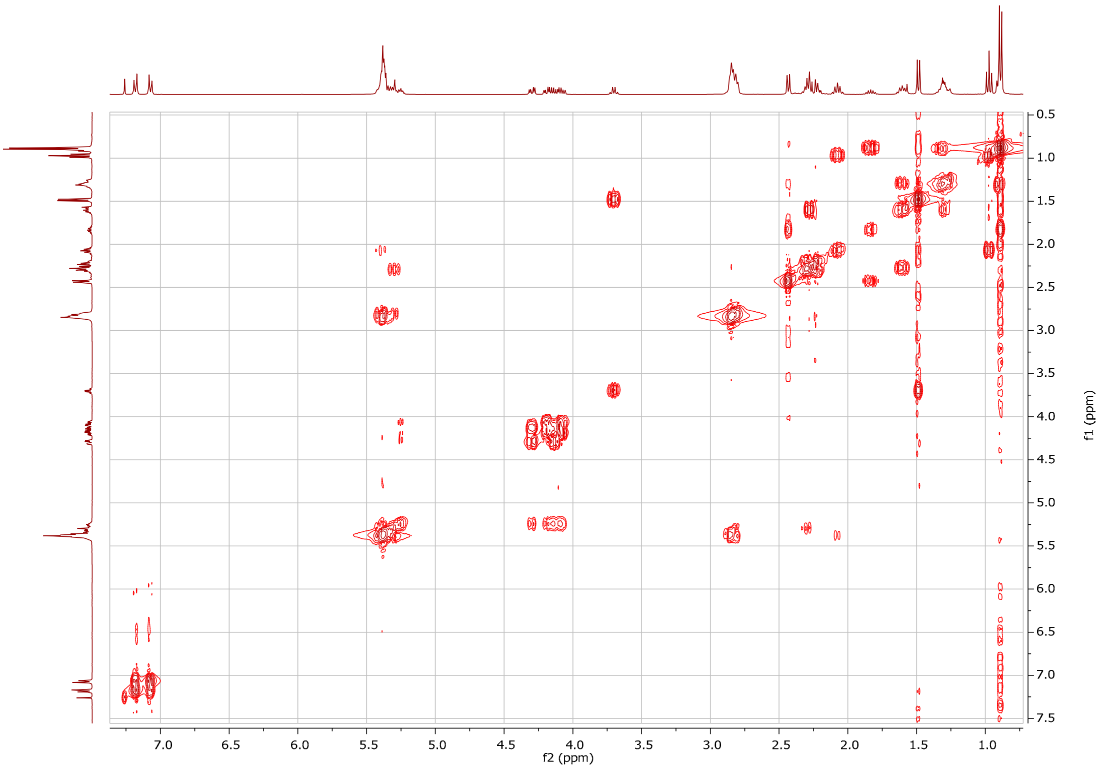

^{13}C-^{1}H HSQC spectrum of compound (*S,S′*)-**12a**

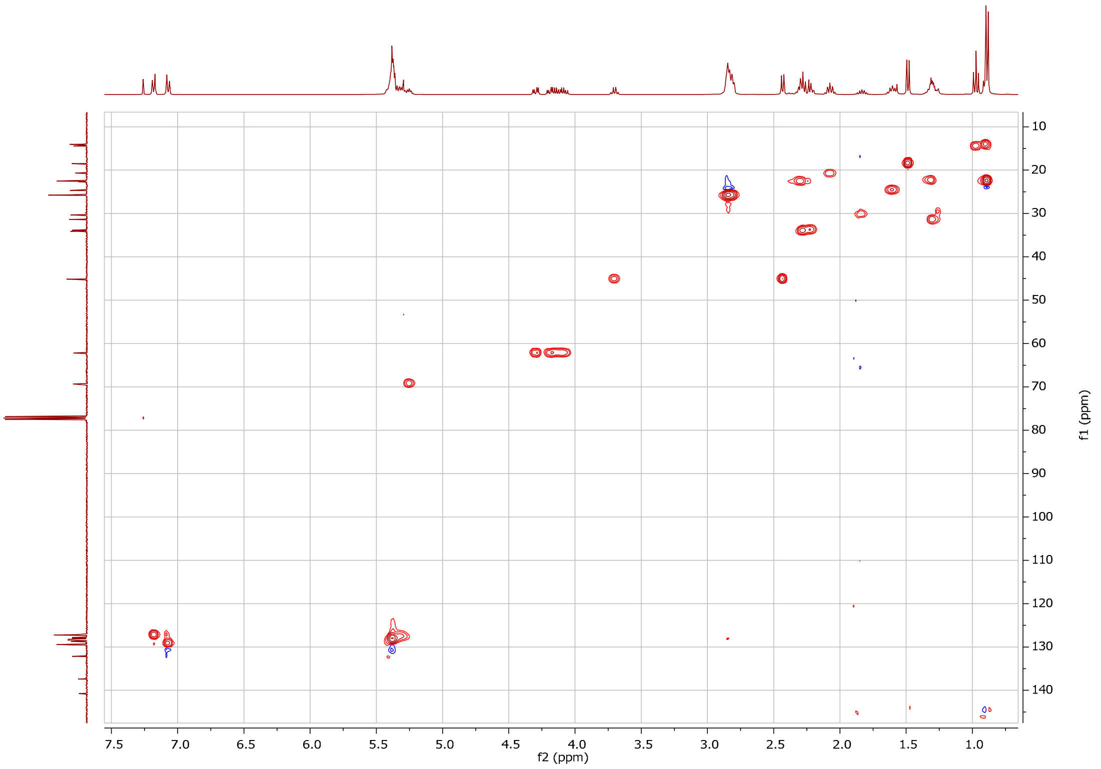

^{1}H NMR (400 MHz, CDCl3) of compound (*R*,*S*′)-**12a**

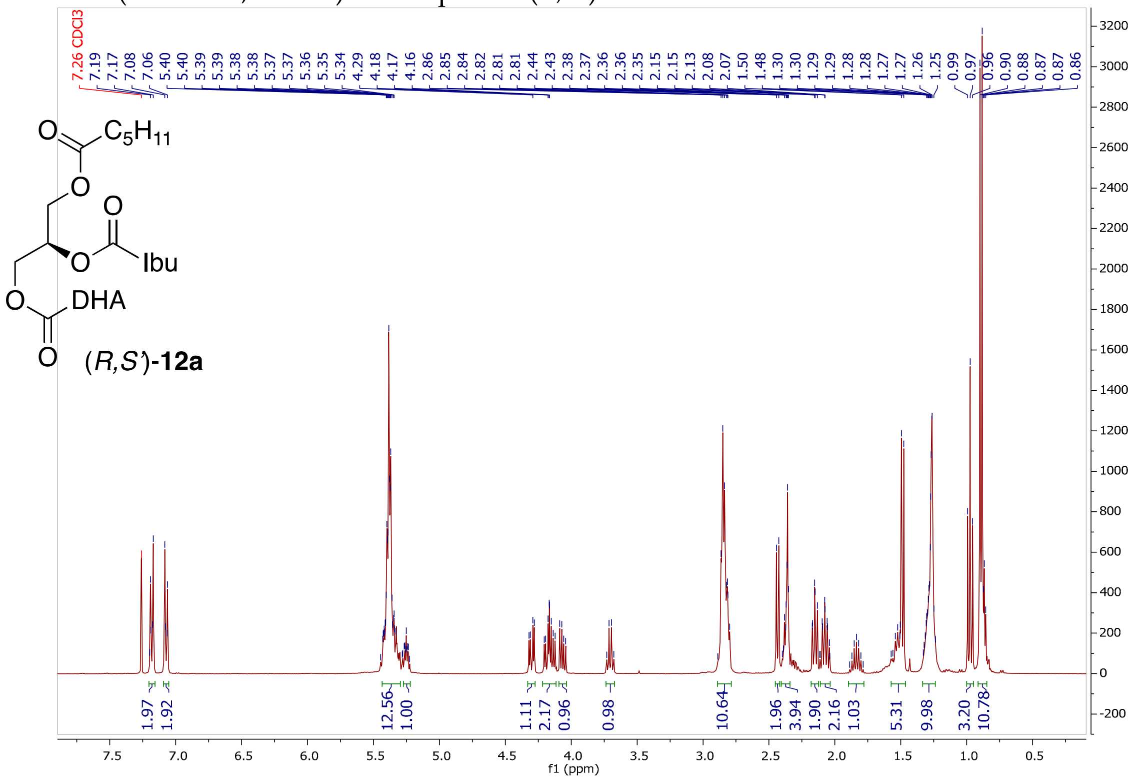

^{13}C{H} NMR (101 MHz, CDCl3) of compound (*R*,*S*′)-**12a**

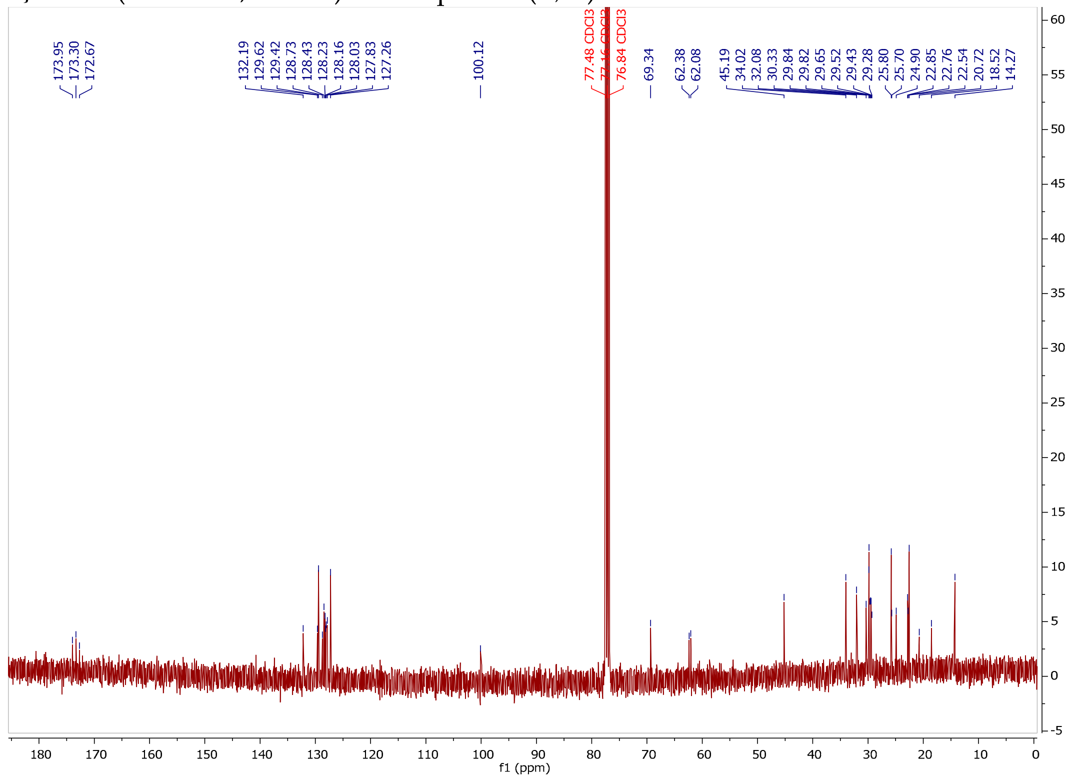

^{1}H-^{1}H COSY spectrum of compound (*R*,*S'*)-**12a**

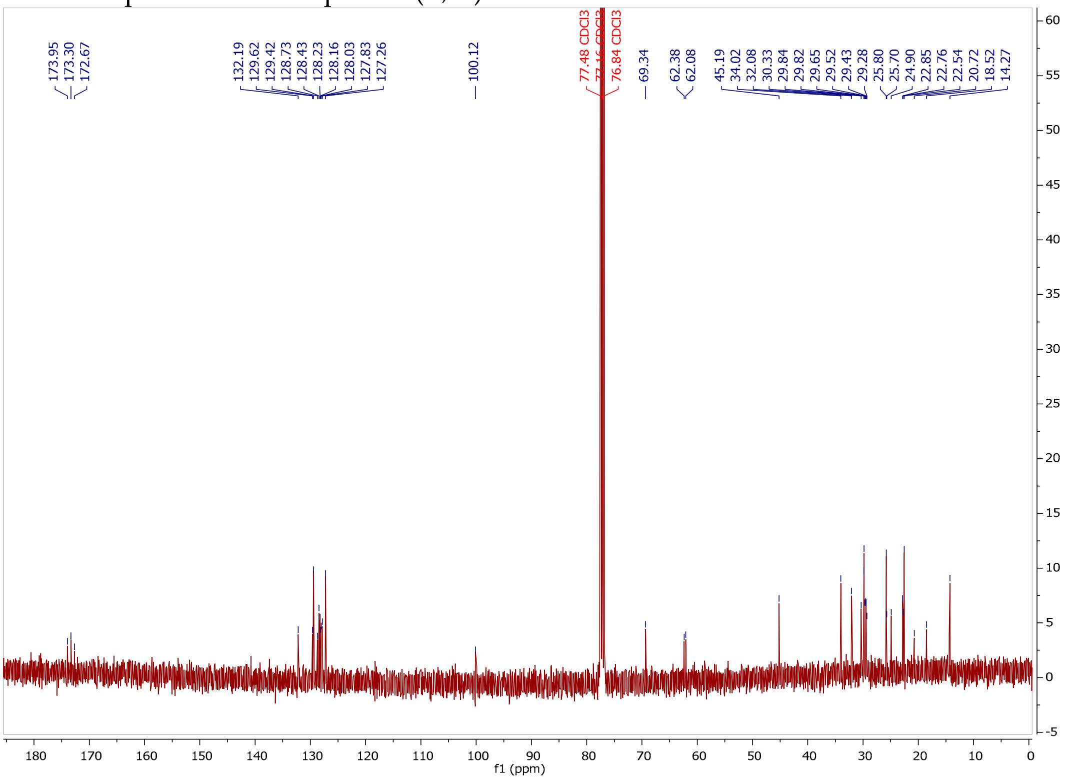

^{13}C-^{1}H HSQC spectrum of compound (*R*,*S'*)-**12a**

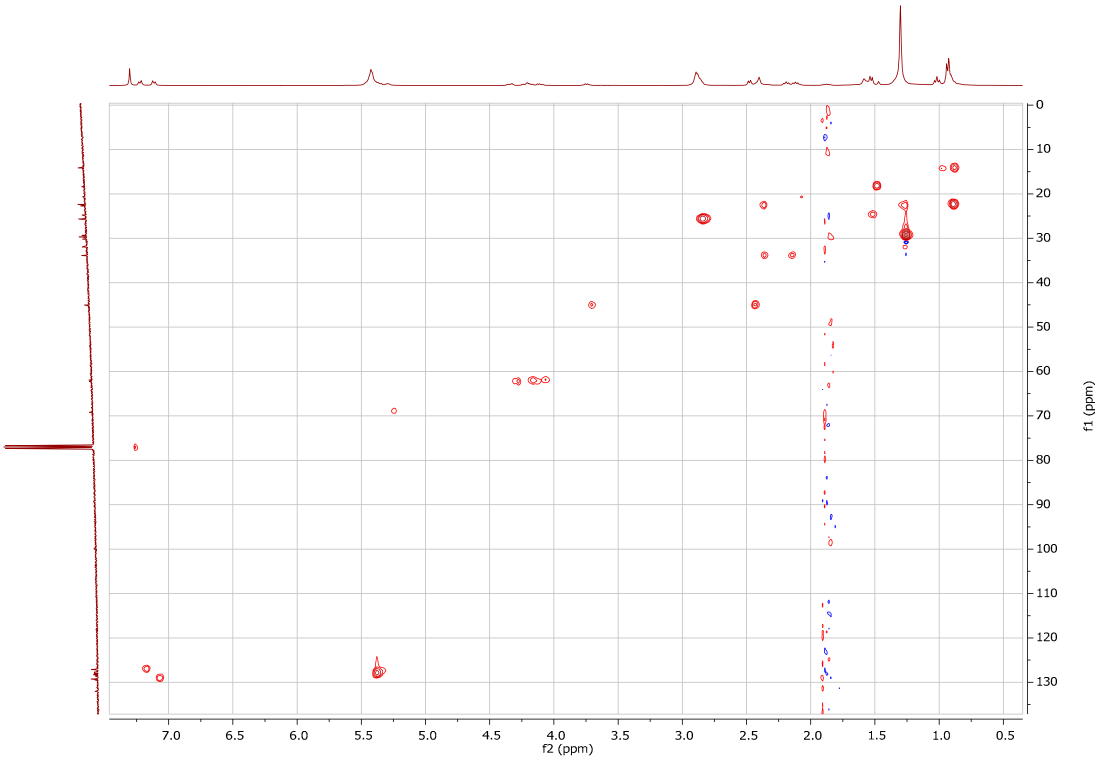

^{1}H NMR (400 MHz, CDCl$_3$) of compound (*S*,*S′*)-**13a**

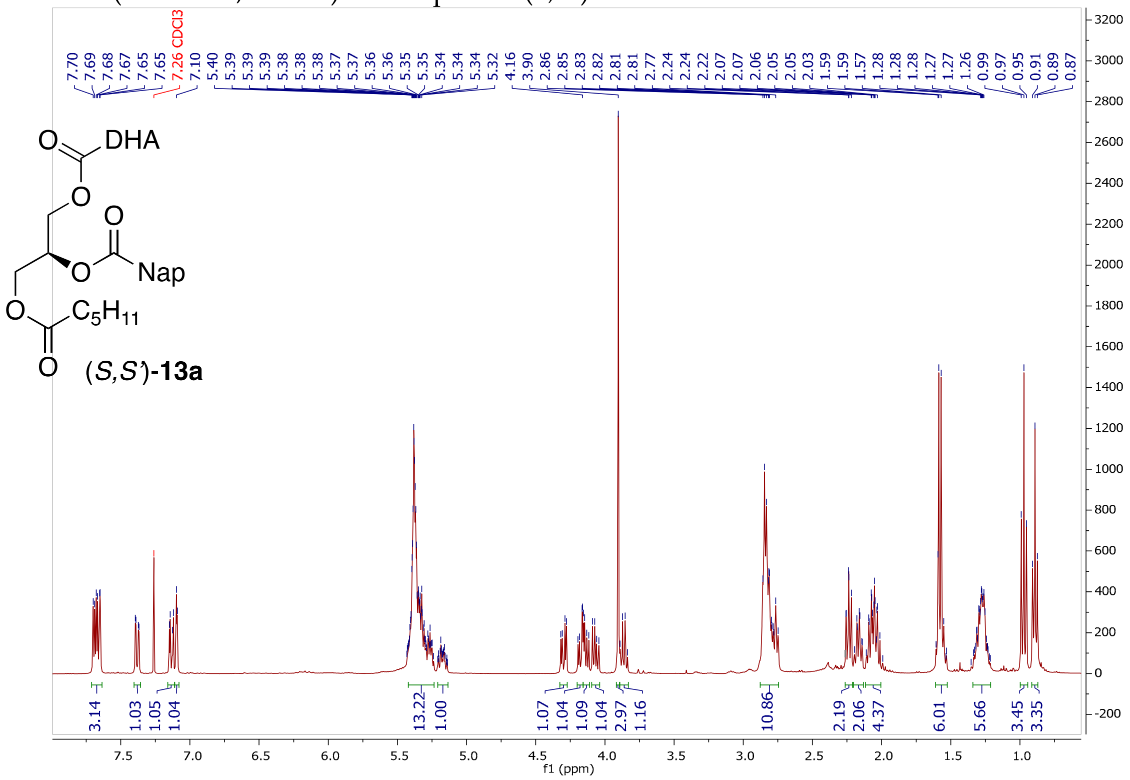

^{13}C{H} NMR (101 MHz, CDCl$_3$) of compound (*S*,*S′*)-**13a**

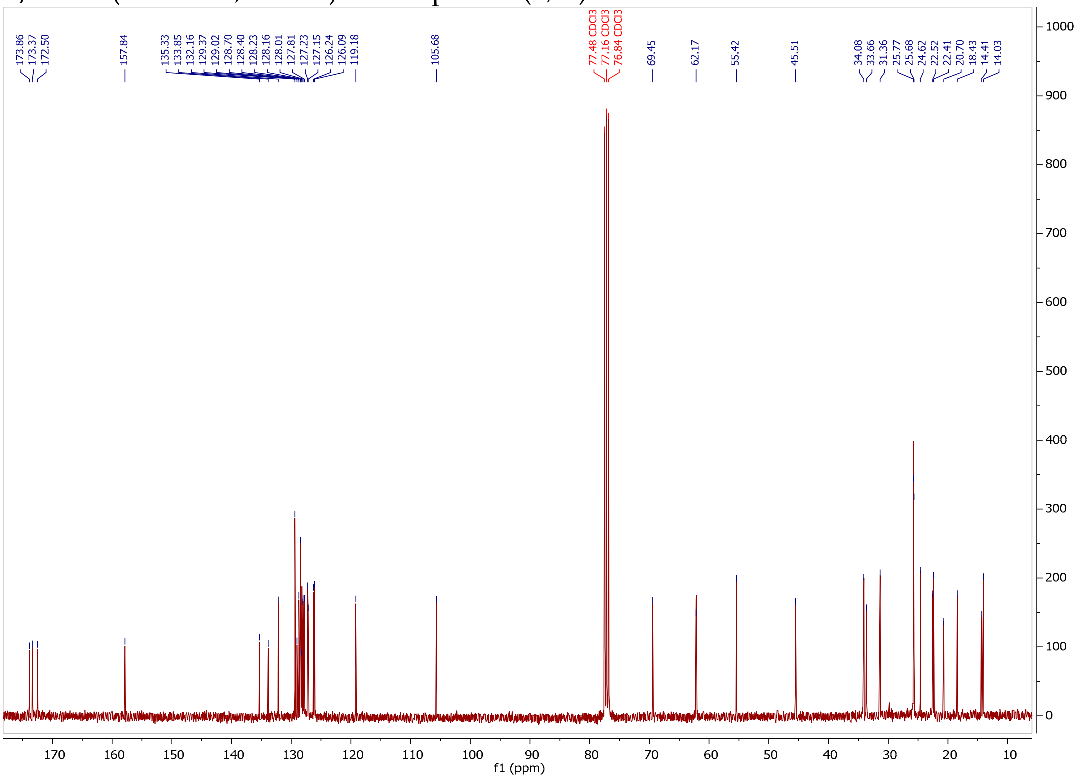

^{1}H-^{1}H COSY spectrum of compound (*S*,*S′*)-**13a**

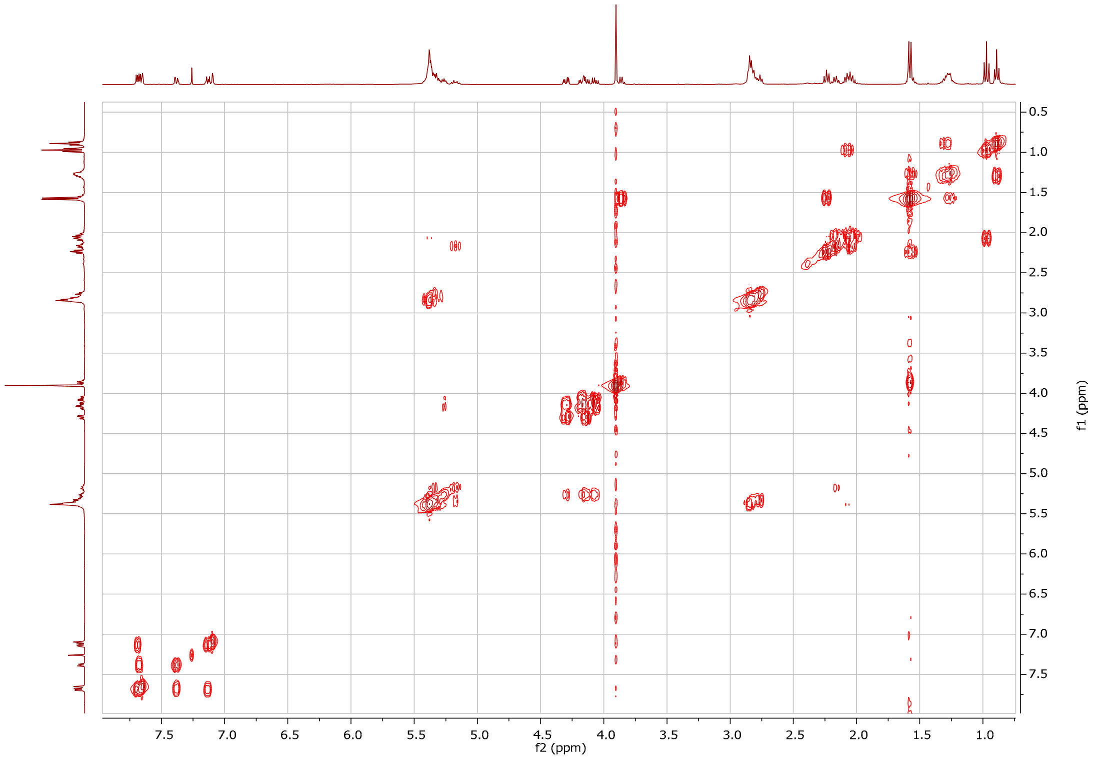

^{13}C-^{1}H HSQC spectrum of compound (*S*,*S′*)-**13a**

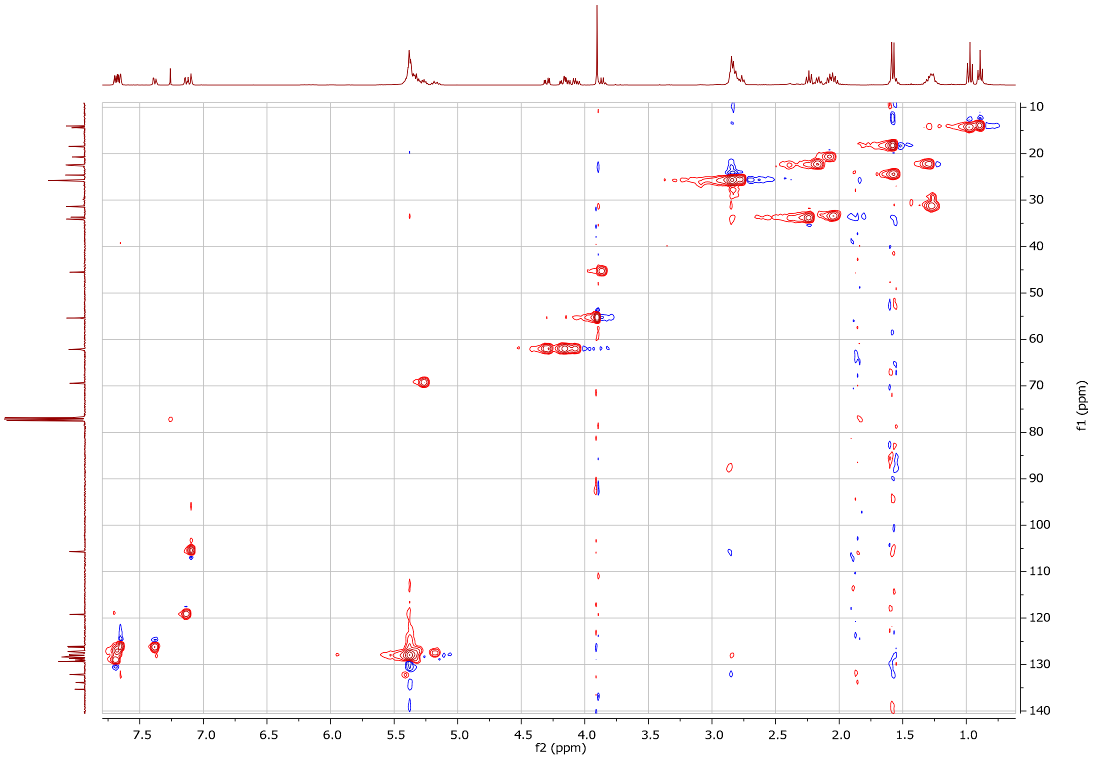

^{1}H NMR (400 MHz, CDCl3) of compound (*R*,*S'*)-**13a**

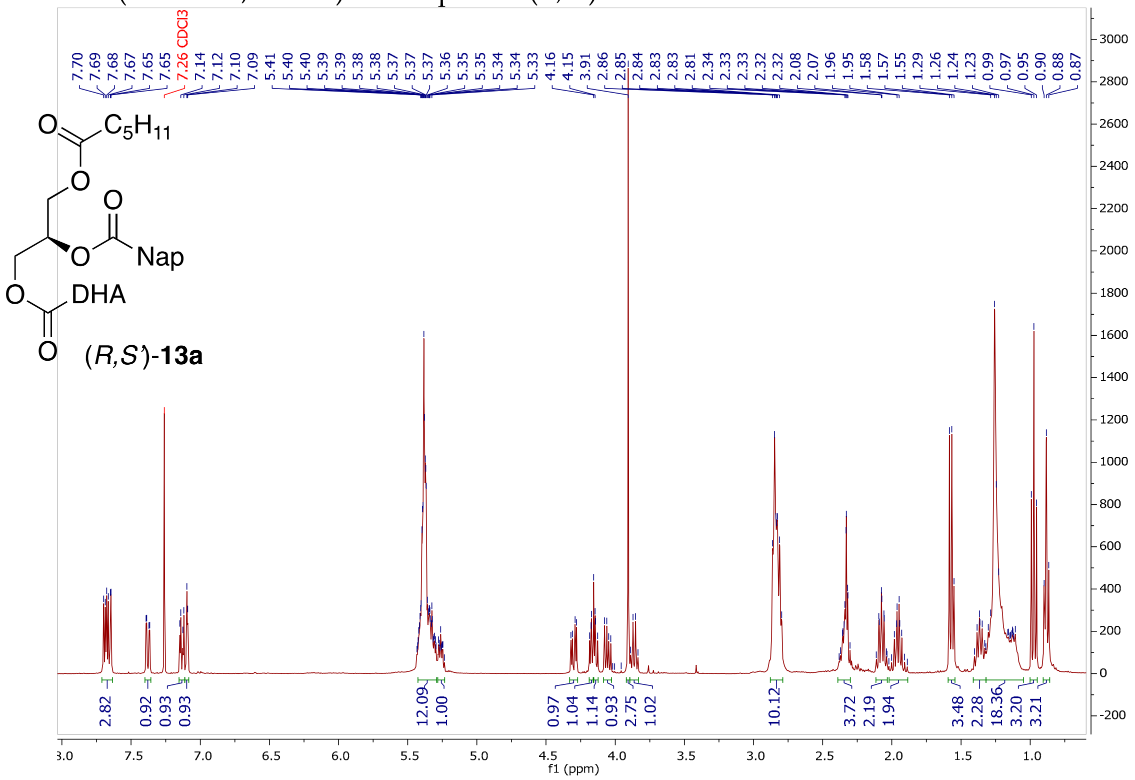

^{13}C{H} NMR (101 MHz, CDCl3) of compound (*R*,*S'*)-**13a**

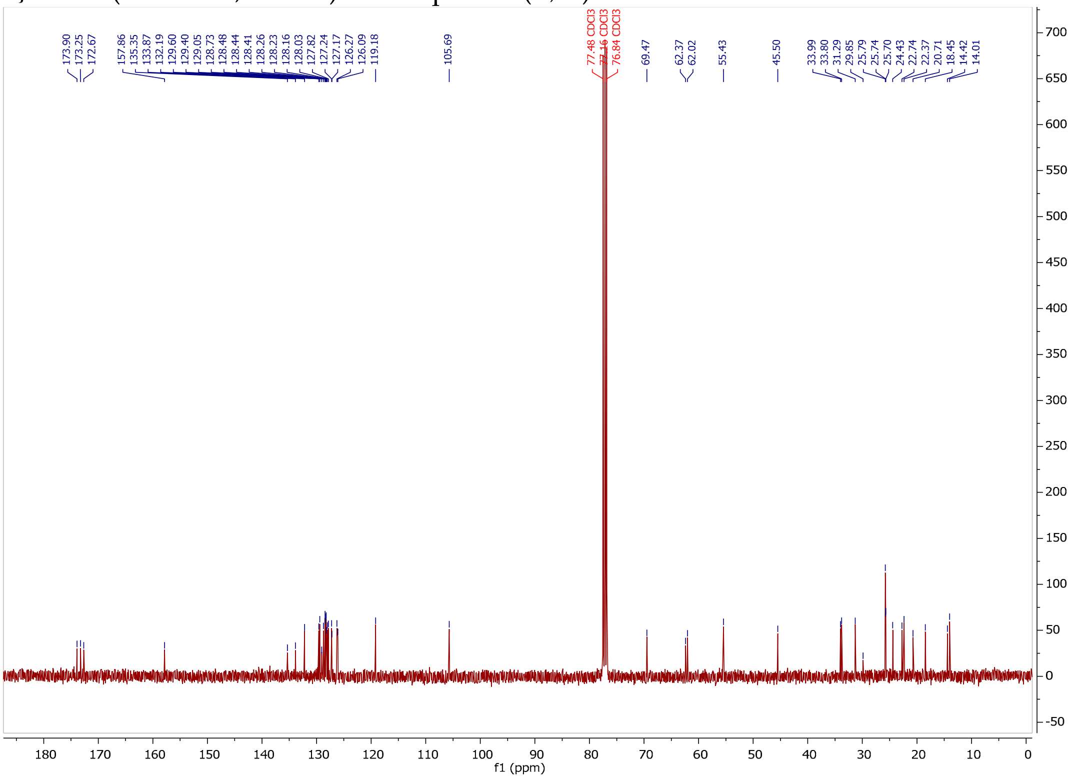

^{1}H-^{1}H COSY spectrum of compound (*R*,*S′*)-**13a**

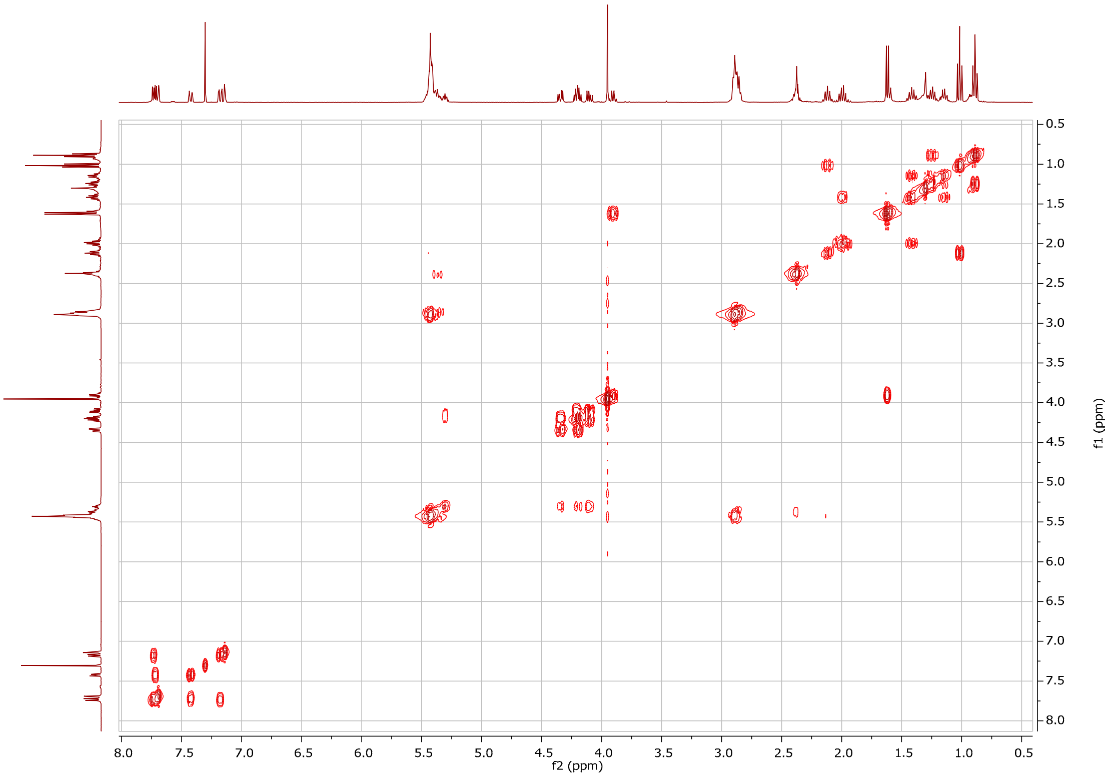

^{13}C-^{1}H HSQC spectrum of compound (*R*,*S′*)-**13a**

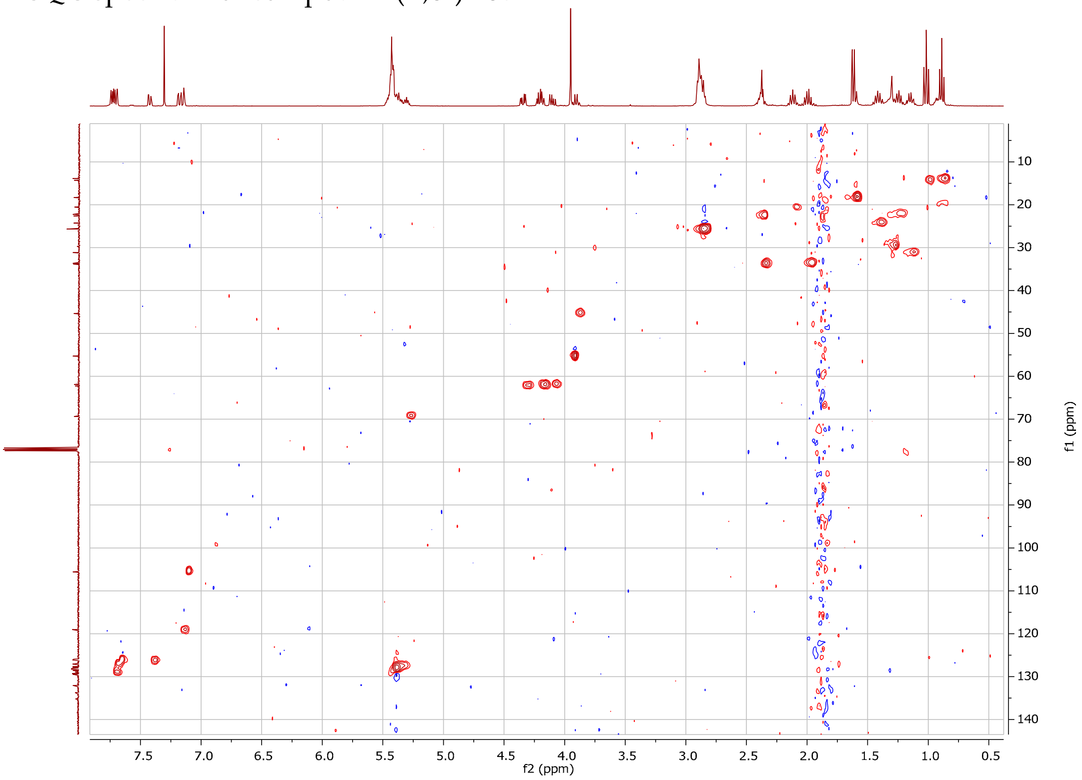

Supplement: Supplementary file 1 [file molecules-29-05745-s001.zip › molecules-3287683-supplementary.pdf]
